# Generation of glucosylated *sn*-1-glycerolphosphate teichoic acids: glycerol stereochemistry affects synthesis and antibody interaction†

**DOI:** 10.1039/d0cb00206b

**Published:** 2020-12-23

**Authors:** Francesca Berni, Liming Wang, Ermioni Kalfopoulou, D. Linh Nguyen, Daan van der Es, Johannes Huebner, Herman S. Overkleeft, Cornelis H. Hokke, Gijsbert A. van der Marel, Angela van Diepen, Jeroen D. C. Codée

**Affiliations:** Leiden Institute of Chemistry, Leiden University Einsteinweg 55 2333 CC Leiden The Netherlands jcodee@chem.leidenuniv.nl; Division of Pediatric Infectious Diseases, Dr von Hauner Children's Hospital, Ludwig-Maximilians-University Munich Germany; Department of Parasitology, Leiden University Medical Center Albinusdreef 2 2333 ZA Leiden The Netherlands

## Abstract

Lipoteichoic acids (LTAs) have been addressed as possible antigen candidates for vaccine development against several opportunistic Gram-positive pathogens. The study of structure-immunogenicity relationship represents a challenge due to the heterogenicity of LTA extracted from native sources. LTAs are built up from glycerol phosphate (GroP) repeating units and they can be substituted at the C-2-OH with carbohydrate appendages or d-alanine residues. The substitution pattern, but also the absolute chirality of the GroP residues can impact the interaction with chiral biomolecules including antibodies and biosynthesis enzymes. We have generated a set of diastereomeric GroP hexamers bearing a glucosyl modification at one of the residues. The chirality of the glycerol building block had an important impact on the stereoselectivity of the glycosylation reaction between the glycosyl donor and the glycerol C-2-OH acceptor. The GroP C-2-chirality also played an important role in the interaction with TA recognizing antibodies. These findings have important implications for the design and synthesis of synthetic TA fragments for diagnostic and therapeutic applications.

## Abstract 2

Glycerol stereochemistry is key for teichoic acid synthesis and antibodies binding.

## Graphical abstract

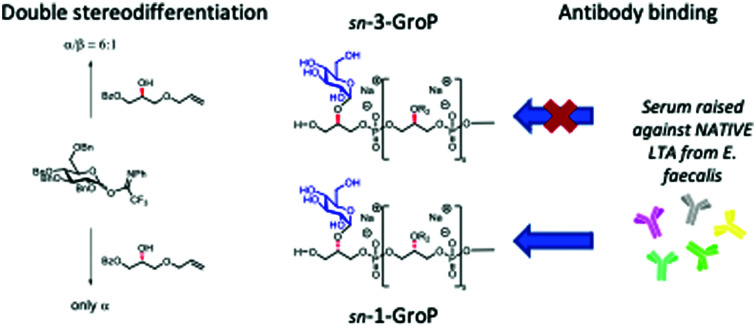

Teichoic acids (TAs) are anionic polymeric structures that compose the cell-wall of many Gram-positive bacterial species with a wide structural variability.^1^ As prime components of the bacterial cell-wall, TAs have several important biological functions. These structures are highly distinctive for Gram-positive bacteria, are antigenic and therefore considered as good antigen candidates for vaccine development against opportunistic pathogens, including important nosocomial enterococci and staphylococci.^2^ Most enterococci and staphylococci bear type I lipoteichoic acids (LTA) on their cell wall, which are composed of an *sn*-glycerol-1-phosphate (*sn*-Gro-1-P) backbone with either d-alanine or carbohydrate substituents at the C-2 position (see Fig. 1).^3^ These appendages, distended to the outside of the bacterial cell-wall, play an important role in the communication with the surrounding environment.^4^ The glycerol stereochemistry of the GroP chain has been established based on the nature of the biosynthesis precursor, as well as metabolism of the TAs. Type I GroP LTA differs from the structurally closely related GroP wall teichoic acid (WTA) as these biopolymers are built up from enantiomeric GroP monomers. While LTA is built up from *sn*-glycerol-1-phosphate, WTA is composed of *sn*-glycerol-3-phosphate residues (see Fig. 1).^5^ LTA is assembled by bacteria using phosphatidyl *sn*-1-glycerol, while WTA is constructed using cytidine diphosphate *sn*-3-glycerol (CDP-glycerol). Mayer and co-workers recently reported that an exo-acting *sn*-3-glycerol phosphodiesterase (GlpQ) from *B. subtilis* was only capable of cleaving *sn*-glycerol-3-phosphate unit from the exposed end of WTA, while this enzyme was not active on an LTA substrate, having the opposite stereochemistry.^6^

**Fig. 1:**
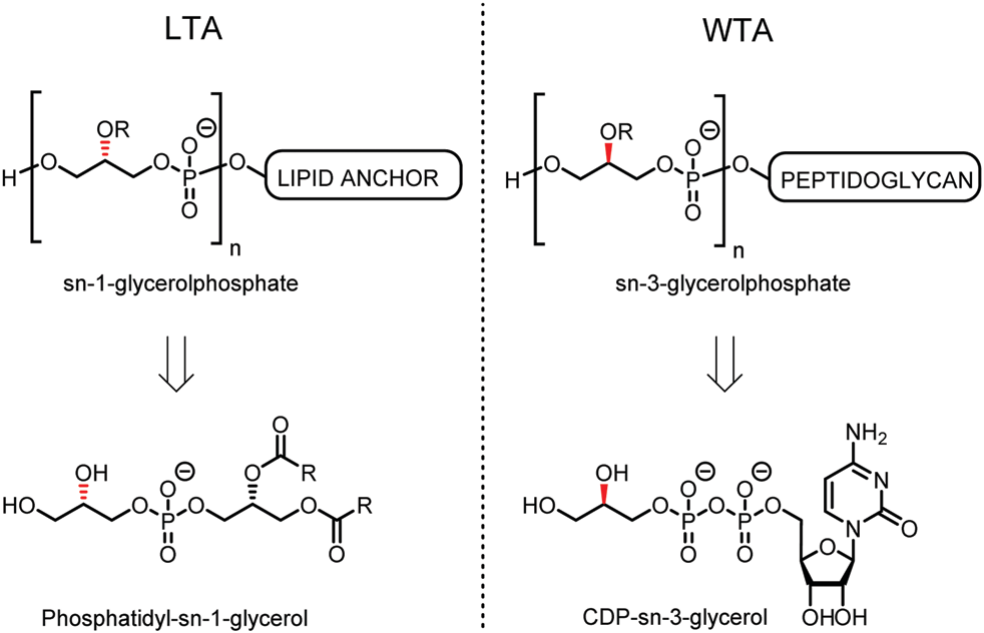
General structure of *sn*-Gro-1-P LTA and *sn*-Gro-3-P WTA and their biosynthesis precursors phosphatidyl-*sn*-1-glycerol and CDP-*sn*-3-glycerol.

Because of the microheterogenicity of TAs, resulting from the different glycosylation and d-alanylation patterns, it has been difficult to determine the precise antigenic elements at the molecular level using isolated TAs.^7^ Synthetic chemistry can provide well-defined structures to establish structure-immunogenicity relationship,^8^ and several groups have reported on strategies to assemble LTA and WTA fragments.^9^ We have previously described different approaches to assemble LTA fragments with well-defined glycosylation patterns. We have equipped these fragments with a linker to attach them to either carrier proteins, fluorescent labels or affinity tags as well as microarray surfaces.^10^ The linker in the molecules we previously generated was attached to the side of the oligomers, formally generating *sn*-Gro-3-P LTAs. From the pool of synthetic LTA oligomers, a glucosylated fragment was selected as a lead antigen, and this structure, WH7 (see Fig. 2A), was attached to a carrier protein (bovine serum albumin, BSA) to provide a model TA-conjugate vaccine.^11^ Realizing that the chirality of the GroP chains may play a role in the interaction with antibodies, we here describe the generation of a set of glucosylated *sn*-Gro-1-P LTA-hexamers **1–6**, which differ in the position of the α-glucose substituent (Fig. 2B). Using a TA-microarray we show that the serum previously generated against the *sn*-Gro-3-P LTA-hexamer does not recognize the fragments with the “natural” chirality well. In contrast, serum raised against isolated LTA is capable of binding to the *sn*-Gro-1-P LTA-hexamers and the interactions depend on the position of the glucosyl substituent.

**Fig. 2:**
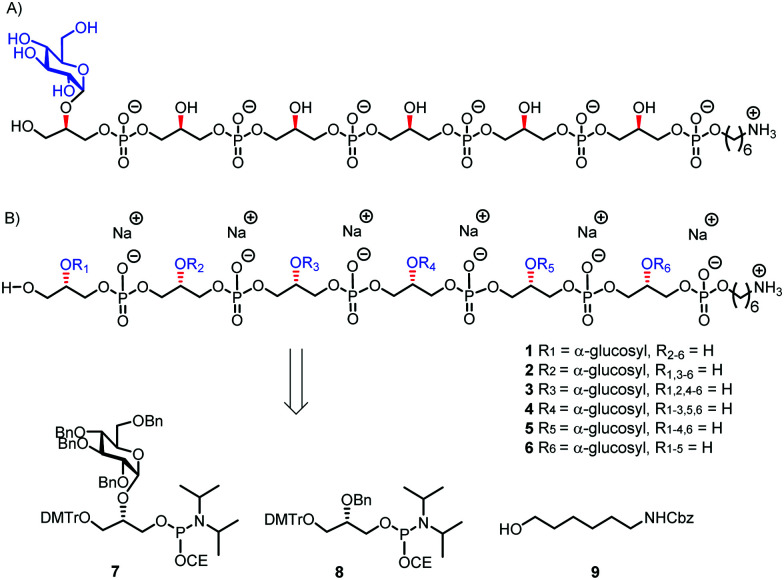
(A) Lead compound WH7; (B) the new set of TA hexamers and the building blocks used for their synthesis.

The required LTA-hexamers were assembled using phosphoramidite building blocks **7** and **8** and linker **9** (Scheme 1B). The glycerol phosphoramidite building blocks **7** and **8** carry a temporary dimethoxytrityl (DMTr) protecting group to enable the assembly of the target TA hexamers using well-established and highly efficient nucleic acid chemistry.^12–15^ The crucial step in the synthesis of building block **7** is the introduction of the 1,2-*cis* glycosidic linkage. To deliver the desired α-glucosyl glycerol intermediate with good stereoselectivity, we previously used a glucosyl imidate donor building block carrying a bulky fluorenylmethoxycarbonyl protecting group at the C-6 position.^11^ The use of a glucosyl donor, carrying solely benzyl ether protecting groups, would reduce the number of required protecting group manipulations. We therefore set out to explore the use of an additive-mediated glycosylation strategy to assemble building block **7** (Table 1).^16^ We have recently described that a combination of trimethylsilyl iodide (TMSI) and an excess of triphenylphosphine oxide (Ph_3_PO) can be used to glycosylate nucleophilic alcohols with a perbenzylated glucosyl imidate donor in a highly stereoselective manner. This strategy was applied here in the coupling of donor **10** and glycerol acceptor **11**, providing compound **17** in 72% yield. Unfortunately, the stereoselectivity was relatively poor (see Table 1, entry 1, *α*/*β* = 1.3/1). We therefore explored the use of acceptor **12** having the same protecting groups but opposite chirality. As shown in entry 2, the stereoselectivity significantly improved, indicating double stereodifferentiation^17^ to play an important role in the union of donor **10** and acceptor **11**/**12**. This finding is quite unexpected as the acceptor used is relatively flexible and small (as compared to other carbohydrate acceptors, for which this phenomenon has been observed). Upon scale up of the reaction, the yield of the glycosylation dropped to 45%, because of loss of the silyl group, and we therefore probed different protecting groups at this position. Since the stereochemistry of the glycerol acceptor had a strong impact on the stereoselectivity of the glycosylation reactions, we examined both enantiomers of the glycerol acceptor bearing either a *para*-methoxybenzyl (PMB) ether or a benzoyl (Bz) ester (**13–16**). The results of the glycosylations are summarized in Table 1, showing that the stereoselectivity is affected by both chirality at C2 and type of substituent at C1. Both enantiomers of the PMB protected alcohols provided the desired *cis*-linked product (entries 3 and 4). The glycosylations of the benzoylated acceptors **15**/**16** also proceeded stereoselectively with the R-isomer **15** providing solely the desired α-anomeric product (entries 5 and 6).

**Scheme 1:**
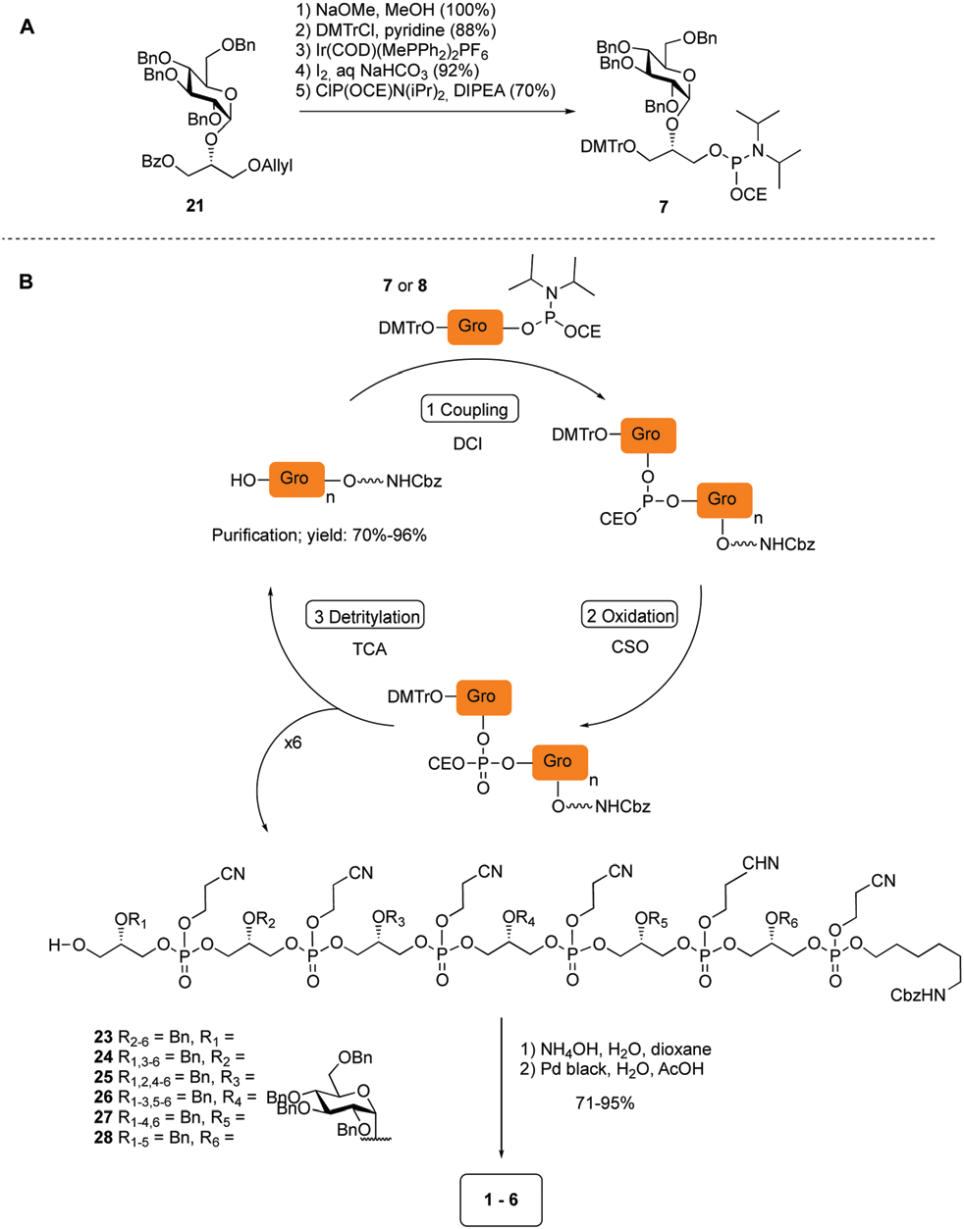
(A) Synthesis of building block **7**. (B) Assembly of hexamers **1–6**.

**Table 1:** Glycosylations of donor **10** and glycerol acceptors **11–16**^a^

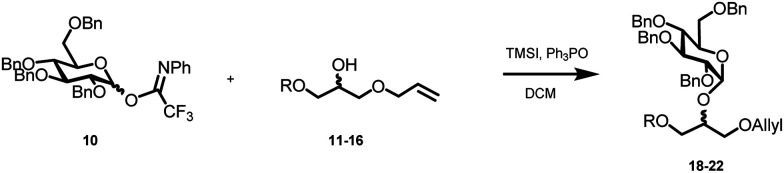						
Entry	R	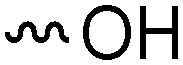	Acc.	Prod.	Yield (%)	*α* : *β*
1	TBDPS	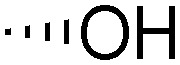	**11**	**17**	72	1.3 : 1
2	TBDPS	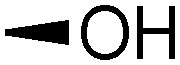	**12**	**18**	68	>10 : 1
3	PMB	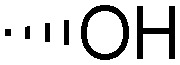	**13**	**19**	65	>10 : 1
4	PMB	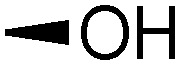	**14**	**20**	66	>10 : 1
5	Bz	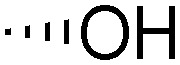	**15**	**21**	68	>10 : 1
6	Bz	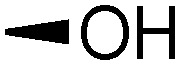	**16**	**22** ^18^	70	6 : 1
7	Bz	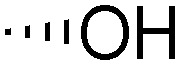	**15**	**21**	86^b^	>10 : 1

a Donor (1 eq.), acceptor (0.7 eq.), TMSI (1 eq.), Ph_3_PO (6 eq.), DCM (0.1 M), r.t., 24 h.

b Reaction time 36 h (15 mmol scale).

The desired α-product (**21**) could be isolated in 68% yield and by extending the reaction time (36 h) the yield was further improved to 86%, which was also reproducible on a large scale (up to 15 mmol, Table 1, entry 7). The results in Table 1 show that double stereodifferentiation in the glycosylations is most prominent when acceptors are used with sterically demanding protecting groups.

Having straightforward access to **21**, we next transformed this building block into the required phosphoramidite **7** as shown in Scheme 1A. Briefly, the benzoate ester in **21** was exchanged for the required DMTr-ether, after which the allyl ether was removed and the cyanoethyl-protected phosphoramidite installed. With building block **7**, **8** (see ESI†) and **9** in hand, the assembly of the GroP hexamers was performed using repetitive coupling cycles in solution. The alcohols, *i.e.* alcohol spacer **9** or the oligomer intermediates, were coupled with phosphoramidite building block **7** or **8** using DCI (4,5-dicyanoimidazole) as activating agent, followed by CSO [(1*S*)-(+)-(10-camphorsulfonyl)-oxaziridine] mediated oxidation of the so-formed phosphite triester. After aqueous work up, the DMTr was removed under mild acidic conditions (0.18 M trichloroacetic acid in DCM). The generated alcohol was then purified and used for the subsequent coupling. All coupling–deprotection cycles proceeded uneventfully delivering the elongated structures in 60–96% yield. After construction of the fully protected hexamers **23–28**, they were deprotected by first removing the cyanoethyl protecting groups under basic conditions, followed by Pd(C) catalyzed hydrogenolysis of all benzyl groups and the Cbz carbamate.

After the generation of the target hexamers, we evaluated them for the binding by anti-LTA antibodies. Recently we have reported the development of a TA-microarray, which allowed the screening of a library of synthetic TA-fragments for binding with mono- or polyclonal antibodies raised against whole bacteria, isolated LTA or a synthetic TA-BSA conjugate vaccine.^10*a*^ It was shown that a commercially available mouse anti *S. epidermidis* monoclonal antibody specifically recognized the glycerol phosphate backbone, while sera obtained by immunization with native LTA from *E. faecalis* 12030^19^ showed preferential binding to glycosylated TA-fragments. The serum raised against the WH7-BSA glycoconjugate very specifically recognized TA-fragments encompassing the WH7 structure.^10*b*^

Thus, the six glucosyl hexamers **1–6**, the lead antigen WH7 and an unsubstituted hexamer (**29**, Fig. 3A), previously generated, were immobilized on epoxy-silane functionalized glass-slides at three different concentrations (30 μM, 10 μM and 3 μM). The microarrays were then used to probe binding of the serum raised against the native LTA from *E. faecalis* 12303 and the anti-WH7-BSA serum. IgG binding was visualized using a fluorescently labelled (DyLight550) goat anti-Rabbit IgG antibody. Fig. 3B and C show the fluorescence read-out as average of three datapoints of these experiments. It becomes immediately apparent that IgG binding is influenced not only by the presence of the glucose substituent and its position, but also by the stereochemistry of the Gro-P backbone. The anti-LTA 12030 serum did not recognize the bare *sn*-Gro-3-P-backbone nor the WH7 antigen. In contrast, it bound well to the *sn*-Gro-1-P-hexamers bearing an α-glucosyl moiety. The antibodies seem to show a slightly better binding to fragments that display the glucosyl moiety further away from the linker. Perhaps the display of the glycosylated antigen close to the microarray surface prohibits binding of the antibody. The IgG antibodies present in the serum raised against WH7-BSA strongly recognized the *sn*-Gro-3-P-antigen WH7, but not its *sn*-Gro-1-P-counterpart **1**, nor any of the other *sn*-Gro-1-P-hexamers. These results clearly reveal that the stereochemistry of the LTA GroP-backbone is a crucial determinant for antibody binding. From the array results it can be concluded that glycosylated GroP-fragments represent important natural epitopes and anti-LTA antibodies can discriminate between glycosylated *sn*-1 and *sn*-3-glycerol fragments. This exquisite recognition implies that the position of the linker in the synthetic antigens is an important element in the design and construction of synthetic LTA-conjugate vaccines. Also, the position of the glucose appendage plays a major role in recognition by the antibodies, which need sufficient space for binding. The results highlight that a very specific antibody response can be elicited through the use of conjugate vaccines carrying single well-defined synthetic LTA-fragment epitopes.

**Fig. 3:**
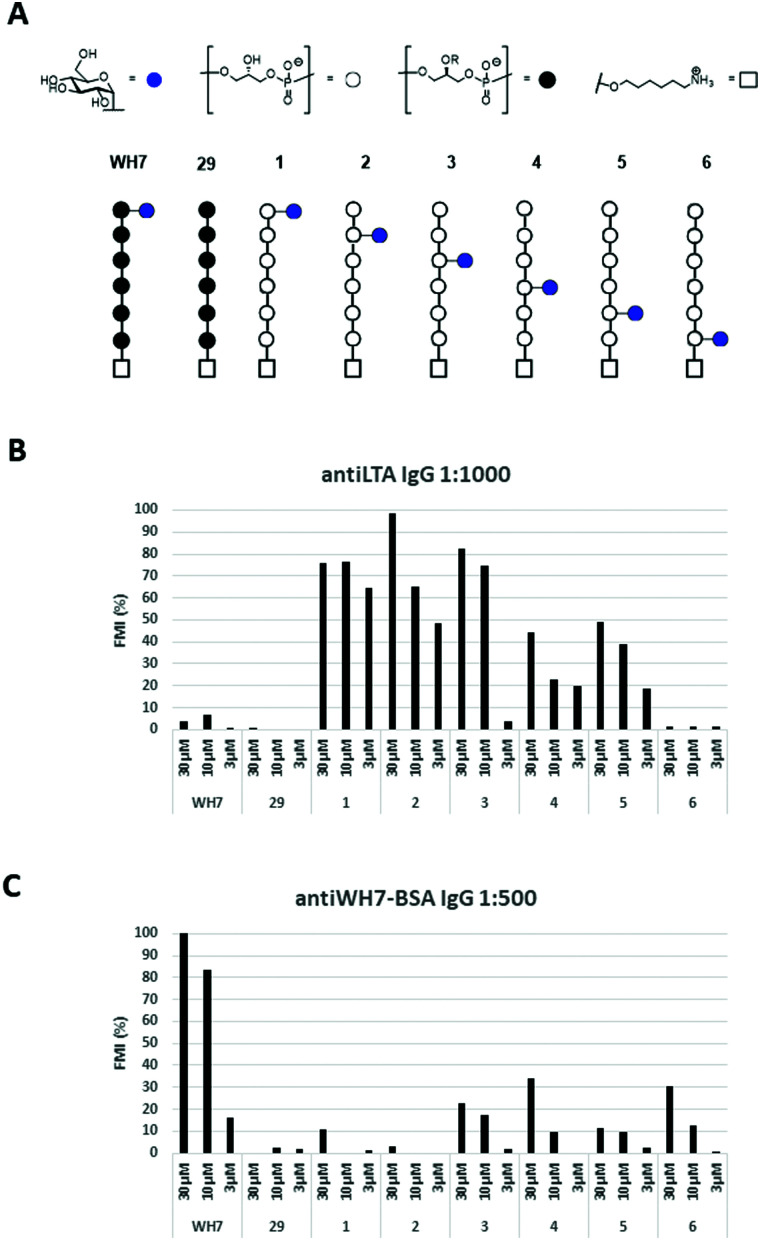
(A) Overview of the TA-fragments tested; (B) IgG binding in rabbit serum raised against native LTA from E. *faecalis* 12030 (1 : 1000 dilution); (C) IgG binding in rabbit serum raised against WH7-BSA (1 : 500 dilution). FMI (%): median florescent intensity normalized to the highest peak.

In conclusion, we have reported the synthesis of a new set of glucosylated GroP-LTA-fragments, featuring a *sn*-Gro-1-P backbone with an α-glucosyl substituent at different positions on the chain. The synthesis of the pivotal building block **7** was achieved by employing an additive-mediated glycosylation strategy. The stereochemistry of the glycerol acceptor proved to be important for the stereochemistry of the glycosylation reaction linking the glucose moiety to the glycerol alcohol. Evaluation of the set of glucosylated *sn*-Gro-1-P hexamers alongside an unsubstituted *sn*-Gro-3-P LTA hexamer and a glucosylated *sn*-Gro-3-P hexamer (WH7) for interactions with anti-LTA antibodies showed that the stereochemistry of the Gro-P backbone plays a decisive role. The position of the α-glucosyl substituent also influenced binding of the antibodies. In the design of conjugate vaccines or diagnostic tools using synthetic TA-fragments, it is therefore important to position the linker connecting the TA fragments to its carrier at the site of the fragment that mimics the natural linkage to the bacterial cell wall.

## Conflicts of interest

There are no conflicts to declare.

## Supplementary Material

CB-002-D0CB00206B-s001
