# Supplementary material for: Generation of glucosylated sn-1-glycerolphosphate teichoic acids: glycerol stereochemistry affects synthesis and antibody interaction
Source: RSC Chem Biol. 2020 Dec 23;2(1):187–91. doi: 10.1039/d0cb00206b (PMC8341164; doi:10.1039/d0cb00206b)
Supplement: CB-002-D0CB00206B-s001 [file CB-002-D0CB00206B-s001.pdf]

Electronic Supplementary Material (ESI) for RSC Chemical Biology.
This journal is © The Royal Society of Chemistry 2020

Generation of sn-1-glucosyl-glycerolphosphate hexamers: an influence of the glycerol stereochemistry from synthesis to antibody interaction.

Francesca Berni[a], Liming Wang[a], Ermioni Kalfopoulou[b], D. Linh Nguyen[c], Daan van der Es[a], Herman S. Overkleeft[a], Cornelis H. Hokke[c], Johannes Huebner[b], Gijsbert A. van der Marel[a], Angela van Diepen[c] and Jeroen D. C. Codée[a]*

[a]Leiden Institute of Chemistry, Leiden University, Einsteinweg 55, 2333 CC Leiden, The Netherlands

[b]Division of Pediatric Infectious Diseases, Dr. von Hauner Children's Hospital, Ludwig-Maximilians-University, Munich, Germany

[c]Department of Parasitology, Leiden University Medical Center, Albinusdreef 2, 2333 ZA Leiden, The Netherlands

Supporting information

Table of Contents

Experimental 2

Synthesis of acceptors 11-16 4

Glycosylation using TMSI/Ph_3PO. 6

Synthesis of building block 7 from 21 9

Phospharamidite couplings 12

Final deprotections 27

Generation and serum analysis of microarray 30

^1H, ^{13}C and ^{31}P NMR spectra 31

Experimental

General

All chemicals (Acros, Fluka, Merck, Sigma-Aldrich, etc.) were used as received and reactions were carried out dry, under an argon atmosphere, at ambient temperature, unless stated otherwise. Column chromatography was performed on Screening Devices silica gel 60 (0.040-0.063 mm). TLC analysis was conducted on HPTLC aluminium sheets (Merck, silica gel 60, F245). Compounds were visualized by UV absorption (245 nm), by spraying with 20% H_2SO_4 in ethanol or with a solution of $(NH_4)_6Mo_7O_{24}\cdot4H_2O$ 25 g/l and $(NH_4)_4Ce(SO_4)_4\cdot2H_2O$ 10 g/l, in 10% aqueous H_2SO_4 or with a solution of $KMnO_4$ (2%) and K_2CO_3 (1%) in water followed by charring at +/- 140 ^{o}C. Optical rotation measurements ($[\alpha]_D^{20}$) were performed on a Propol automated polarimeter (Sodium D-line, λ = 589 nm) with a concentration of 10 mg/ml (c = 1), unless stated otherwise and the reported value was calculated as the mean of 10 measurements. Infrared spectra were recorded on a Shimadzu FT-IR 8300. ^{1}H, ^{13}C and ^{31}P NMR spectra were recorded with a Bruker AV 400 (400, 101 and 162 MHz respectively), a Bruker AV 500 (500, 125 and 202 MHz respectively) or a Bruker DMX 850 (850, 214 and 344 MHz respectively). NMR spectra were recorded in $CDCl_3$ with chemical shift (δ) relative to tetramethylsilane for both ^{1}H and ^{13}C. When D_2O or CD_3CN were used, ^{1}H-NMR were recorded with chemical shift (δ) relative to the proton of residual solvent (4.75 ppm and 1.94 ppm respectevely). ^{13}C-NMR spectra were recorded with chemical shift (δ) relative to TMS (external standard) in case of D_2O and 1.32 ppm as residual solvent in CD_3CN.The ^{31}P- NMR spectra were recorded with chemical shift (δ) relative to H_3PO_4. (external standard). High resolution mass spectra were recorded by direct injection (2 µl of a 2 µM solution in water/acetonitrile; 50/50; v/v and 0.1 % formic acid) on a mass spectrometer (Thermo Finnigan LTQ Orbitrap) equipped with an electrospray ion source in positive mode (source voltage 3.5 kV, sheath gas flow 10, capillary temperature 250 °C) with resolution R = 60000 at *m/z* 400 (mass range *m/z* = 150-2000) and dioctylphthalate (*m/z* = 391.28428) as a lock mass. High resolution mass spectrometer was calibrated prior to measurements with a calibration mixture (Thermo Finnigan).

Synthesis of acceptors 11-16

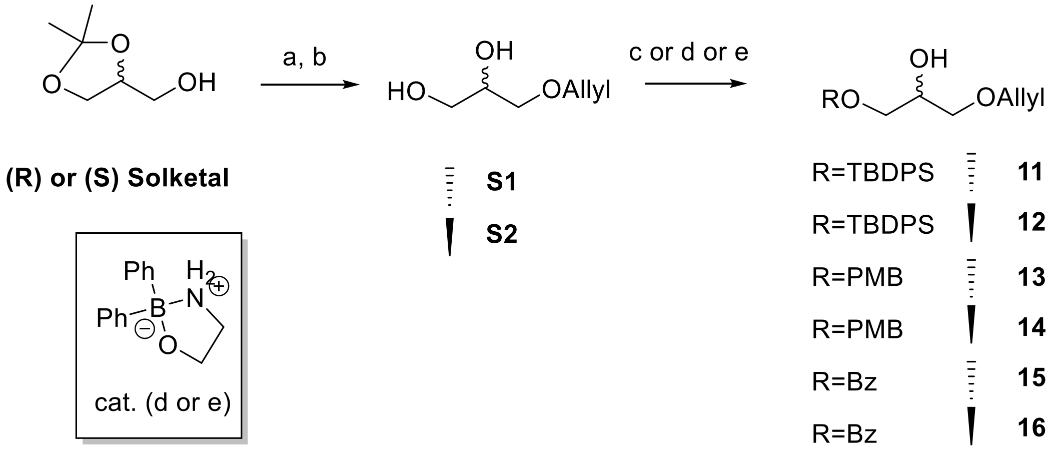

Scheme S 1: Synthetic strategy for synthesis of acceptors ***11-16***. a) AllylBr, NaH, DMF 96%; b) AcOH, H_2O, 50 °C, 300mbar, quant; c) TBDPSCl, Imidazole, DMF, 82% (**11**), 80% (**12**); d) cat., PMBCl, KI, K_2CO_3, ACN, 60 °C, quant (**13**), 96% (**14**); e) cat., BzCl, DIPEA, CAN, 98% (**15**), quant. (**16**).

Note: for experimental procedure and data analysis of steps (a) and (b) see J. Shin, D. H. Thompson, *JOC*, **2003**, 68, 17, 6760-6766

(S)-1-*O*-allyl-3-*O*-(*tert*-butyldiphenylsilyl)-*sn*-glycerol (11)

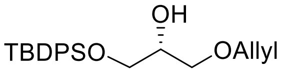

Diol **S1** (0.86 mmol) was diluted in DMF (8.6 ml, 0.1 M) and Imidazole (1 mmol, 1.15 eq) and TBDSCl (0.86 mmol, 1 eq) were added. After two hours stirring at room temperature, TLC analysis (DCM:MeOH, 95:5) showed complete conversion of the starting material. The reaction mixture was diluted with Et_2O (10 mL) and washed with H_2O (10mL x 3). The aqueous phase was reextracted with Et_2O and the combined organic layers were washed once with brine, dried over $Na_2S_2O_4$, filtered and concentrated *in vacuo*. Compound **11** was isolated by column chromatography (Pentane:EtOAc, 9:1; R_f: 0.31) as transparent oil in 82% yield (0.71 mmol).

^{1}H-NMR (400 MHz, $CDCl_3$), δ: 7.70-7.62 (4H, H_{arom}, m), 7.47-7.35 (6H, H_{arom}, m), 5.95-5.82 (1H, H_{allyl}, m), 5.29-5.15 (2H, H_{2_allyl}, m), 4.03-3.97 (2H, CH_{2_allyl}, m), 3.95-3.86 (1H, $CH_{glycerol}$, m), 3.71 (2H, $CH_{2\_glycerol}$, J_{CH2-CH}=5.4 Hz, d), 3.58-3.44 (2H, $CH_{2\_glycerol,}$ m), 2.49 (1H, OH, J_{OH-CH}=5.1Hz, d), 1.06 (9H, tBu, s).

^{13}C-NMR (101 MHz, $CDCl_3$), δ: 135.7 (CH_{arom}), 134.7 (CH_{allyl}), 133.1 (C_q), 129.9 (CH_{arom}), 127.9 (CH_{arom}), 117.3 (CH_{allyl}), 72.5 (CH_{2_allyl}), 71.0 ($CH_{2\_glycerol}$), 70.9 ($CH_{glycerol}$), 64.9 ($CH_{2\_glycerol}$), 27.0 (CH_{3_tBu}), 18.9 (C_{q_tBu}).

($[\alpha]_D^{20}$($CHCl_3$): -4.1

HRMS: $C_{22}H_{30}O_3Si + Na^+$ required 359.1856, found 359.1901.

(R)-1-*O*-(*tert*-butyldiphenylsilyl)-3-*O*-allyl-*sn*-glycerol (12)

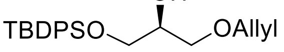

Starting with diol **S2** (1.00 mmol), compound **12** was obtained following the procedure described for **11** in 80% yield (0.80 mmol).

^{1}H-NMR (400 MHz, $CDCl_3$), δ: 7.70-7.62 (4H, H_{arom}, m), 7.47-7.35 (6H, H_{arom}, m), 5.95-5.82 (1H, H_{allyl}, m), 5.29-5.15 (2H, 2 x H_{allyl}, m), 4.03-3.97 (2H, CH_{2_allyl}, m), 3.95-3.86 (1H, $CH_{glycerol}$, m), 3.71 (2H, $CH_{2\_glycerol}$, J_{CH2-CH}=5.4 Hz, d), 3.58-3.44 (2H, $CH_{2glycerol,}$ m), 2.49 (1H, OH, J_{OH-CH}=5.1Hz, d), 1.06 (9H, tBu, s).

^{13}C-NMR (101 MHz, $CDCl_3$), δ: 135.7 (CH_{arom}), 134.7 (CH_{allyl}), 133.1 (C_q), 129.9 (CH_{arom}), 127.9 (CH_{arom}), 117.3 (CH_{allyl}), 72.5 (CH_{2_allyl}), 71.0 ($CH_{2\_glycerol}$), 70.9 ($CH_{glycerol}$), 64.9 ($CH_{2\_glycerol}$), 27.0 (CH_{3_tBu}), 18.9 (C_{q_tBu})

$[\alpha]_D^{20}$($CHCl_3$): +3.5

HRMS: $C_{22}H_{30}O_3Si + Na^+$ required 359.1856, found 359.1901.

(R)-1-*O*-allyl-3-*O*-(4-methoxybenzyl)-*sn*-glycerol (13)

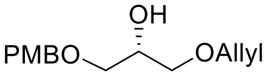

Diol **S1** (1.00 mmol) was coevaporated three times with toluene and dissolved under inert atmosphere in dry ACN (2.5 mL, 0.4 M) and the flask was wrapped in aluminium foil. After 10 minutes stirring, PMBCl (1.10 mmol, 1.1 eq) was added followed by K_2CO_3 (1.10 mmol, 1.1 eq) and KI (1 mmol, 1 eq). The reaction was heated to 60 °C and after stirring overnight TLC analysis (DCM:MeOH; 95:5) showed complete consumption of starting material. The reaction mixture was cooled to r.t., diluted with EtOAc and washed with H_2O. The water layer was extract with EtOAc and the combined organic layers were washed with Brine, dried over MgSO4 and concentrated *in vacuo*. The resulting crude was purified by column chromatography (8:2→7:3 Pentane:EtOAc) yielding **13** as a colorless oil in quantitative yield (1.00 mmol).

TLC analysis: R_f= 0.35 (Pentane:EtOAc; 7:3)

^{1}H-NMR (400 MHz, $CDCl_3$), δ: 7.28-7.23 (2H, H_{arom}, m), 6.91-6.86 (2H, H_{arom}, m), 5.96-5.84 (1H, H_{allyl}, m), 5.31-5.15 (2H, H_{2_allyl}, m), 4.49 (2H, CH_{2_PMB}, s), 4.04-3.95 (3H, CH_{2_allyl}, $CH_{glycerol}$, m), 3.80 (2H, CH_{3_OMe}, s), 3.57-3.43 (4H, $CH_{2\_glycerol}$, m), 2.46 (1H, OH, J_{OH-CH} =4.2 Hz, d).

^{13}C-NMR(101 MHz, $CDCl_3$), δ: 134.5 (CH_{allyl}), 130.1 (C_q), 129.4 (CH_{arom}), 117.3 (CH_{allyl}), 113.9 (CH_{arom}), 73.1 (CH_{2_PMB}), 72.3 (CH_{2_allyl}), 71.3 ($CH_{2\_glycerol}$), 71.04 ($CH_{2\_glycerol}$), 69.6 ($CH_{2\_glycerol}$), 55.3 (CH_{3_OMe}).

$[\alpha]_D^{20}$($CHCl_3$): -7.1

HRMS: $C_{14}H_{20}O_4 + Na^+$ required 275.1254, found 275.1259

(S)-1-*O*-(4-methoxybenzyl)-3-*O*-allyl-*sn*-glycerol (14)

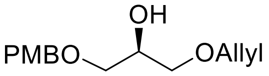

Starting from diol **S2** (1.00), compound **14** was obtained as colorless oil in 96% yield (0.96 mmol) following the procedure described for compound **13**.

TLC analysis: R_f= 0.35 (Pentane:EtOAc; 7:3)

^{1}H-NMR (400 MHz, $CDCl_3$), δ: 7.28-7.23 (2H, H_{arom}, m), 6.91-6.86 (2H, H_{arom}, m), 5.96-5.84 (1H, H_{allyl}, m), 5.31-5.15 (2H, 2 x H_{allyl}, m), 4.49 (2H, CH_{2_PMB}, s), 4.04-3.95 (3H, CH_{2_allyl}, $CH_{glycerol}$, m), 3.80 (2H, CH_{3_OMe}, s), 3.57-3.43 (4H, $CH_{2\_glycerol}$, m), 2.46 (1H, OH, J=4.2 Hz, d).

^{13}C-NMR(101 MHz, $CDCl_3$), δ: 134.5 (CH_{allyl}), 130.1 (C_q), 129.4 (CH_{arom}), 117.3 (CH_{allyl}), 113.9 (CH_{arom}), 73.1 (CH_{2_PMB}), 72.3 (CH_{2_allyl}), 71.3 ($CH_{2\_glycerol}$), 71.04 ($CH_{2\_glycerol}$), 69.6 ($CH_{2\_glycerol}$), 55.3 (CH_{3_OMe}).

$[\alpha]_D^{20}$($CHCl_3$): +7.5

HRMS: $C_{14}H_{20}O_4 + Na^+$ required 275.1254, found 275.1259

(R)-1-*O*-allyl-3-*O*-benzoyl-*sn*-glycerol (15)

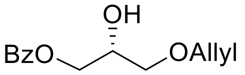

Diol **S1** (10 mmol) was coevaporated with toluene three times and dissolved under inert atmosphere in dry ACN (25 mL, 0.4M). The flask was wrapped in aluminium foil and after ten minutes stirring, BzCl (11 mmol, 1.1 eq), DiPEA (12 mmol, 1.2 eq) and 2-Aminoethyl diphenylborinate (0.1 mmol, 0.01 eq) were subsequently added. The reaction was left to stir at room temperature and after 2h TLC analysis (DCM:MeOH; 95:5) showed complete consumption of starting material. The reaction mixture was diluted with EtOAc and washed with H_2O. The water layer was reextracted with EtOAc and the combined organic layers were washed with brine, dried over $MgSO_4$ and concentrated *in vacuo*. The resulting crude was purified by column chromatography (85:15→7:3, pentane:EtOAc) yielding **15** in 98% yield (9.8 mmol).

TLC analysis: R_f= 0.35 (Pentane:EtOAc; 75:25)

^{1}H-NMR (400 MHz, $CDCl_3$), δ: 8.09-8.03 (2H, H_{arom}, m), 7.61-7.55 (1H, H_{arom}, m), 7.49-7.41 (2H, H_{arom}, m), 5.97-5.85 (1H, H_{allyl}, m), 5.33-5.18 (2H, H_{2_allyl}, m), 4.48-4.36 (2H, $CH_{2\_glycerol,}$ m), 4.22-4.11 (1H, $CH_{glycerol}$, m), 4.08-4.03 (2H, CH_{2_allyl}, m), 3.65-3.53 (2H, $CH_{2\_glycerol}$, m), 2.64-2.56 (1H, OH, bs).

^{13}C-NMR(101 MHz, $CDCl_3$), δ: 166.7 (C_q), 134.2 (CH_{allyl}), 133.2 (CH_{arom}), 129.9 (C_q), 129.7 (CH_{arom}), 128.4 (CH_{arom}), 117.6 (CH_{allyl}), 72.5 (CH_{2_allyl}), 710.9 ($CH_{2\_glycerol}$), 69.0 ($CH_{glycerol}$), 66.0 ($CH_{2\_glycerol}$).

$[\alpha]_D^{20}$ ($CHCl_3$) : -5.6

HRMS:$C_{13}H_{16}O_4$ + Na^+ required 259.0941, found 259.1002

(S)-1-*O*-benzoyl-3-*O*-allyl-*sn*-glycerol (16)

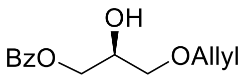

Starting from diol **S2** (1 mmol), compound **16** was obtained as colourless oil in quantitative yield (1.00 mmol), following the procedure described for compound **15**.

TLC analysis: R_f= 0.35 (Pentane:EtOAc; 75:25)

^{1}H-NMR (400 MHz, $CDCl_3$), δ: 8.09-8.03 (2H, H_{arom}, m), 7.61-7.55 (1H, H_{arom}, m), 7.49-7.41 (2H, H_{arom}, m), 5.97-5.85 (1H, H_{allyl}, m), 5.33-5.18 (2H, 2 x H_{allyl}, m), 4.48-4.36 (2H, $CH_{2glycerol,}$ m), 4.22-4.11 (1H, $CH_{glycerol}$, m), 4.08-4.03 (2H, CH_{2_allyl}, m), 3.65-3.53 (2H, $CH_{2\_glycerol}$, m), 2.64-2.56 (1H, OH, bs).

^{13}C-NMR(101 MHz, $CDCl_3$), δ: 166.7 (C_q), 134.2 (CH_{allyl}), 133.2 (CH_{arom}), 129.9 (C_{q_arom}), 129.7 (CH_{arom}), 128.4 (CH_{arom}), 117.6 (CH_{allyl}), 72.5 (CH_{2_allyl}), 710.9 ($CH_{2\_glycerol}$), 69.0 ($CH_{glycerol}$), 66.0 ($CH_{2\_glycerol}$).

$[\alpha]_D^{20}$($CHCl_3$): +4.7

HRMS:$C_{13}H_{16}O_4$ + Na^+ required 259.0941, found 259.0993

Glycosylation using TMSI/Ph_3PO.

General procedure

Donor (1 eq) and acceptor (0.75 eq) were co-evaporated three times with toluene. Under argon atmosphere, they were dissolved in dry DCM (0.1M) and after 10 minutes stirring Ph_3PO (6 eq) was added, followed by slow addition of TMSI (1 eq). The reaction mixture was allowed to stir at r.t. overnight. The reaction mixture was diluted with DCM, washed with $Na_2S_2O_3$, H_2O and brine. The organic layer was dried over $MgSO_4$, filtered and concentrated *in vacuo*. The crude was subjected to size exclusion gel chromatography (DCM:MeOH=1:1, for purification of the final product obtained as mixture of anomers (unless otherwise stated). The ratio α/β was calculated by ^{1}H-NMR.

(S)-1-*O*-allyl-2-*O*-(2,3,4,6-*O*-benzyl-α-D-glucopyranosyl)-3-*O*-(*tert*-butyldiphenylsilyl-*sn*-glycerol (17)

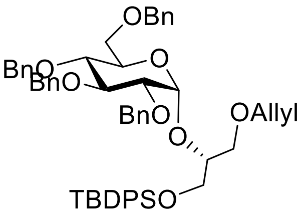

On a scale of 0.10 mmol of donor **10**, following the general procedure, compound **17** was obtained in 72% yield (0.072 mmol) as colourless syrup in a α/β mixture (1.5:1).
TLC analysis: R_f= 0.48 (Pentane:EtOAc; 9:1)
^{1}H-NMR (400 MHz, $CDCl_3$), δ(α): 7.68-7.64 (4H, H_{arom}, m), 7.47-7.07 (26H, H_{arom}, m), 5.94-5.81 (1H, H_{allyl}, m), 5.33-5.07 (3H, 2 x H_{allyl}, H_1 m), 5.02-4.90 (1H, CHH_{Bn}, m), 4.84-4.62 (2H, 3 x CHH_{Bn}, m), 4.68-4.43 (4H, 4 x CHH_{Bn}, m), 4.12-4.05 (1H, H_5, m), 4.01-3.86 (4H, H_3, $CH_{glycerol}$, CH_{2_allyl}, m), 3.82-3.50 (8H, 2 x $CH_{2\_glycerol}$, 2 x H_6, H_4, H_2, m), 1.08 (9H, tBu, s).
^{13}C-NMR(101 MHz, $CDCl_3$), δ(α): 139.1, 138.6, 138.2 (C_q), 135.7, 135.7 (CH_{arom}), 134.9 (CH_{allyl}), 133.1 (C_q), 129.9, 128.5, 128.1, 127.9, 127.8, 127.7 (CH_{arom}), 116.7 (CH_{2_allyl}), 96.2 (C_1), 82.1 (C_3), 79.9 (C_2), 77.8 (C_4), 77.3 ($CH_{glycerol}$), 75.8, 75.1, 73.6, 73.0 (CH_{2_Bn}), 72.2 (CH_{2_Allyl}), 70.4 (C_6), 70.2 (C_5), 68.8, 63.2 ($CH_{2\_glycerol}$), 27.0 (CH_{3_tBu}), 19.4 (C_q).
$[\alpha]_D^{20}$($CHCl_3$): +26.3
HRMS: $C_{56}H_{64}O_8Si + Na^+$ required 915.4263, found 915.4265

(R)-1-*O*-(*tert*-butyldiphenylsilyl)-2-*O*-(2,3,4,6-*O*-benzyl-α-D-glucopyranosyl)-3-*O*-allyl-*sn*-glycerol (18)

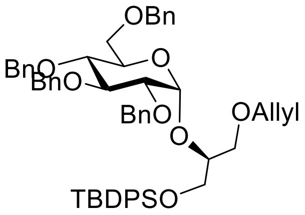

On a scale of 0.1 mmol of donor **10**, following the general procedure described above, compound **18** was isolated as colourless oil in a mixture of α/β anomers (>10:1) in 68% yield (0.068 mmol).
Analytical data in accordance with the one reported in: W. F. J. Hogendorf, L. J. van den Bos, H. S. Overkleeft, J. D. C. Codee, G. A. van der Marel, *Bioorg, Med. Chem.,* **2010**, 18, 3668-3678.

(R)-1-*O*-allyl-2-*O*-(2,3,4,6-*O*-benzyl-α-D-glucopyranosyl)-3-*O*-(4-methoxybenzyl)-*sn*-glycerol (19)

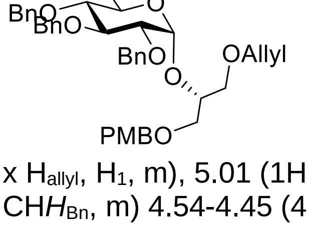

On a scale of 0.1 mmol of donor **10**, following the general procedure, compound **19** was obtained in 65% yield (0.065 mmol) as colourless syrup in a α/β mixture (>10:1).
TLC analysis: R_f= 0.34 (Pentane:EtOAc; 8:2)
^{1}H-NMR (101 MHz, $CDCl_3$), δ(α): 7.42-7.22 (20H, H_{arom}, m), 7.203-7.12 (2H, H_{arom}, m), 6.90-6.84 (2H, H_{arom}, m), 5.94-5.81 (1H, H_{allyl}, m), 5.30-5.12 (3H, 2 x H_{allyl}, H_1, m), 5.01 (1H, CHH_{Bn}, J=10.9 Hz, d), 4.88-4.78 (2H, 2 x CHH_{Bn}, m), 4.71-4.61 (3H, 2 x CHH_{Bn}, m) 4.54-4.45 (4H, 4 x CHH_{Bn}, m), 4.17-4.08 (1H, $CH_{glycerol}$, m), 4.07-3.93 (4H, H_5, H_3, CH_{2_allyl}, m), 3.81 (3H, CH_{3_OMe}, s), 3.76 (1H, $CHH_{glycerol}$, $J_{CHH\text{-}CHH}$=10.7 Hz, $J_{CHH\text{-}CH}$=3.4 Hz, dd), 3.72-3.51 (7H, $CHH_{glycerol}$, $CH_{2\_glycerol}$, 2 x H_6, H_4, H_2, m).

^{13}C-NMR(400 MHz, CDCl$_3$), δ(α): 139.0, 138.5, 138.2, 138.0 (C$_q$), 134.7 (CH$_{allyl}$), 130.3 (C$_q$), 129.2, 128.3, 128.0 x 2, 127.9 x 2, 127.7, 127.6 x 2, 127.5 (CH$_{arom}$), 117.0 (CH$_{2_allyl}$), 96.2 (C$_1$), 81.9 (C$_3$), 79.5 (C$_2$), 77.7 (C$_4$), 75.7, 75.0 (CH$_{2_Bn}$), 74.7 (CH$_{glycerol}$), 73.5, 73.0, 72.3 (CH$_{2_Bn}$), 72.2 x 2 (CH$_{2_Allyl}$, CH$_{2_Bn}$), 70.4 (CH$_{2_glycerol}$), 70.2 (C$_5$), 69.7 (C$_6$), 55.3 (CH$_{3_OMe}$)
$[\alpha]_D^{20}$(CHCl$_3$): +31.2
HRMS: $C_{48}H_{54}O_9$ + Na$^+$ required 9797.3660, found 797.3667

(S)-1-*O*-(4-methoxybenzyl)-2-*O*-(2,3,4,6-*O*-benzyl-α-D-glucopyranosyl)-3-*O*-allyl-*sn*-glycerol (20)

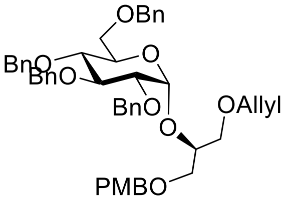

On a scale of 0.1 mmol of donor **10**, following the general procedure, compound **20** was obtained in 66% (0.066 mmol) yield as colourless syrup in a α/β mixture (9:1).
TLC analysis: R$_f$= 0.34 (Pentane:EtOAc; 8:2)
^{1}H-NMR (400 MHz, CDCl$_3$), δ(α): 7.39-7.16 (20H, H$_{arom}$, m), 7.15-7.08 (2H, H$_{arom}$, m), 6.80-6.73 (2H, H$_{arom}$, m), 5.93-5.81 (1H, H$_{allyl}$, m), 5.29-5.11 (3H, 2 x H$_{allyl}$, H$_1$, m), 4.98 (1H, CH*H*$_{Bn}$, J=10.8 Hz, d), 4.84-4.77 (2H, 2 x CH*H*$_{Bn}$, m), 4.74 (1H, CH*H*$_{Bn}$, J=12.0 Hz, d), 4.69 (1H, CH*H*$_{Bn}$, J=12.0 Hz, d), 4.57 (1H, CH*H*$_{Bn}$, J=12.1 Hz, d), 4.48-4.34 (4H, 4 x CH*H*$_{Bn}$, m), 4.12-3.94 (5H, CH$_{glycerol}$, H$_5$, H$_3$, CH$_{2_allyl}$, m), 3.79-3.71 (4H, CH*H*$_{glycerol}$, CH$_{3_OMe}$, s), 3.67-3.51 (6H, 2 x CH*H*$_{glycerol}$, 2 x H$_6$, H$_4$, H$_2$, m), 3,45 (1H, CH*H*$_{glycerol}$, J$_{CHH\text{-}CHH}$=10.6 Hz, J$_{CHH\text{-}CH}$=2,1 Hz, dd),
^{13}C-NMR(101 MHz, CDCl$_3$), δ(α): 139.1, 138.7, 138.5, 138.2 (C$_q$), 134.8 (CH$_{allyl}$), 130.4 (C$_q$), 129.6, 128.5 x 3, 128.2, 128.1, 128.0, 127.8 x 2, 127.7, 127.6 (CH$_{arom}$), 117.1 (CH$_{2_allyl}$), 113.8 (CH$_{arom}$), 96.3 (C$_1$), 82.1 (C$_3$), 79.8 (C$_2$), 77.8 (C$_4$), 75.8, 75.1 (CH$_{2_Bn}$), 74.8 (CH$_{glycerol}$), 73.6, 73.1, 72.6 (CH$_{2_Bn}$), 72.5 (CH$_{2_Allyl}$), 70.8, 70.3 (CH$_{2_glycerol}$), 70.2 (C$_5$), 69.6 (C$_6$), 55.4 (CH$_{3_OMe}$)
$[\alpha]_D^{20}$(CHCl$_3$): +19.4
HRMS: $C_{48}H_{54}O_9$ + Na$^+$ required 797.3660, found 797.3664

(R)-1-*O*-allyl-2-*O*-(2,3,4,6-*O*-benzyl-α-D-glucopyranosyl)-3-*O*-benzoyl-*sn*-glycerol (21)

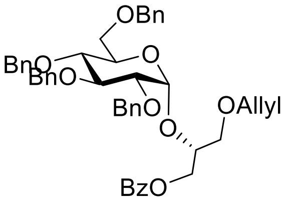

On a scale of 15 mmol of donor **10**, following the general procedure and leaving the reaction stirring for 3 days, compound **21** was obtained in 86% yield (12.9 mmol) as colourless syrup (no presence of β anomer was detected).
TLC analysis: R$_f$= 0.31 (Pentane:EtOAc; 8:2)
^{1}H-NMR (400 MHz, CDCl$_3$), δ(α): 8.05-8.00 (2H, H$_{arom}$, m), 7.57-7.52 (1H, H$_{arom}$, m), 7.43-7.23 (15H, H$_{arom}$, m), 7.18 (5H, H$_{arom}$, s), 7.15-7.10 (2H, H$_{arom}$, m), 5.90-5.79 (1H, H$_{allyl}$, m), 5.28-5.12 (3H, 2 x H$_{allyl}$, H$_1$, m), 4.95 (1H, CH*H*$_{Bn}$, J=10.8 Hz, d), 4.86-4.76 (2H, 2 x CH*H*$_{Bn}$, m), 4.65-4.58 (3H, 3 x CH*H*$_{Bn}$, m), 4.54 (1H, CH*H*$_{glycerol}$, J$_{CHH\text{-}CHH}$=10.8 Hz, J$_{CHH\text{-}CH}$=4.03 Hz, dd), 4.50-4.40 (3H, 2 x CH*H*$_{Bn}$, CH*H*$_{glycerol}$, m), 4.28-4.19 (1H, CH$_{glycerol}$, m), 4.04-3.94 (4H, H$_5$, H$_3$, CH$_{2_allyl}$, m), 3.78-3.55 (6H, CH$_{2_glycerol}$, 2 x H$_6$, H$_4$, H$_2$, m).
^{13}C-NMR(101 MHz, CDCl$_3$), δ(α): 166.5 (C$_q$), 139.0, 138.5, 138.1, 138.0 (C$_q$), 134.5 (CH$_{allyl}$), 133.2 (CH$_{arom}$), 130.0 (C$_q$), 129.8, 128.7, 128.6, 128.5, 128.4, 128.0 x 2, 127.9, 127.8 x 2, 127.7, 127.1 (CH$_{arom}$, 117.4 (CH$_{2_allyl}$), 96.4 (C$_1$), 82.0 (C$_3$), 79.8 (C$_2$), 77.7 (C$_4$), 75.7, 75.2 (CH$_{2_Bn}$), 73.9 (CH$_{glycerol}$), 73.7, 72.9 (CH$_{2_Bn}$), 72.4 (CH$_{2_Allyl}$), 70.6 (C$_5$), 70.0 (C$_6$), 68.6, 64.7 (CH$_{2_glycerol}$).
$[\alpha]_D^{20}$(CHCl$_3$): +22.4
HRMS: $C_{47}H_{50}O_9$ + Na$^+$ required 781.3347, found 781.3354

(S)-1-*O*-benzoyl-2-*O*-(2,3,4,6-*O*-benzyl-α-D-glucopyranosyl)-3-*O*-allyl-*sn*-glycerol (22)

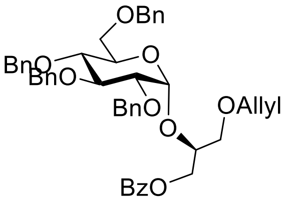

On a scale of 0.1 mmol of donor **10**, following the general procedure, compound **22** was obtained in 70% yield (0.070 mmol) as colourless syrup in a α/β mixture (6:1).

TLC analysis: R_f= 0.31 (Pentane:EtOAc; 8:2)

^{1}H-NMR (400 MHz, $CDCl_3$), δ(α): 8.02-7.97 (2H, H_{arom}, m), 7.53-7.47 (1H, H_{arom}, m), 7.39-7.17 (20H, H_{arom}, m), 7.11-7.03 (2H, H_{arom}, m), 5.94-5.81 (1H, H_{allyl}, m), 5.31-5.15 (3H, 2 x H_{allyl}, H_1, m), 4.97 (1H, CHH_{Bn}, J=10.8 Hz, d), 4.84-4.76 (2H, 2 x CHH_{Bn}, m), 4.73 (2H, CH_{2_Bn}, J=2.9 Hz, d) 4.58-4.49 (2H, CHH_{Bn}, $CHH_{glycerol}$, m), 4.44-4.34 (2H, CHH_{Bn}, $CHH_{glycerol}$, m), 4.28 (1H, CHH_{Bn}, J=12.1 Hz, d), 4.26-4.20 (1H, $CH_{glycerol}$, m), 4.05-3.94 (4H, H_5, H_3, CH_{2_allyl}, m), 3.71-3.53 (4H, 2 x H_6, H_4, H_2, m). 3.49 (1H, $CHH_{glycerol}$, $J_{CHH\text{-}CHH}$ =10.6 Hz, $J_{CHH\text{-}CH}$=3.1 Hz, dd), 3.34 (1H, $CHH_{glycerol}$, $J_{CHH\text{-}CH}$=2.1 Hz, dd).

^{13}C-NMR(101 MHz, $CDCl_3$), δ(α): 166.4 (C_q), 139.0, 138.5, 138.4, 137.9 (C_q), 134.5 (CH_{allyl}), 133.1 ()CH_{arom}), 130.0 (C_q), 129.8, 128.6 x 2, 128.5 x 2, 128.4, 128.2, 128.1, 128.0 x 2, 127.8, 127.7 x 2, (CH_{arom}), 117.4 (CH_{2_allyl}), 96.3 (C_1), 82.0 (C_3), 79.7 (C_2), 77.6 (C_4), 75.8, 75.1 (CH_{2_Bn}), 73.8 ($CH_{glycerol}$), 73.6, 72.8 (CH_{2_Bn}), 72.5 (CH_{2_Allyl}), 70.5 (C_5), 69.8 (C_6), 68.2, 65.2 ($CH_{2\_glycerol}$).

$[\alpha]_D^{20}$($CHCl_3$): +35.6

HRMS: $C_{47}H_{50}O_9$ + Na^+ requires 781.3347, found 781.3357

Synthesis of building block 7 from 21

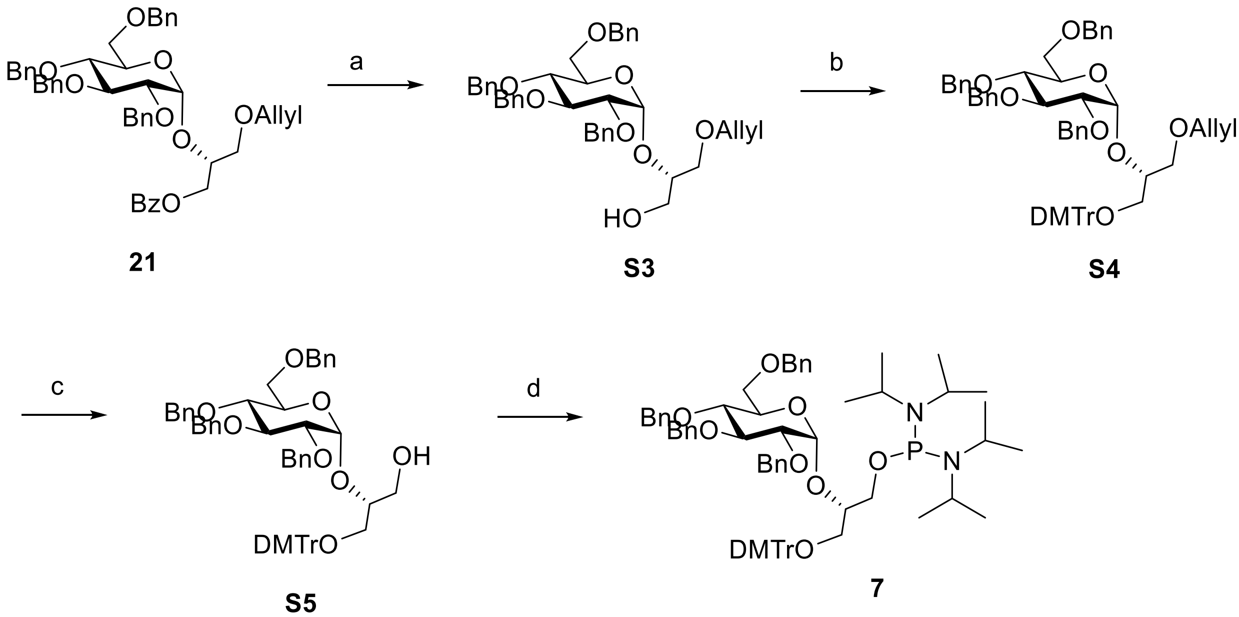

*Scheme S2: Synthetic strategy towards compound **7**.* a) $Na_{(s)}$, MeOH, quant.; b) DMTrCl, TEA, DCM, 88%; c) (i) $Ir(COD)(PPh_2Me)_2PF_6$, H_2, THF; (ii) $NaHCO_{3(aq)}$, I_2, THF, 92%; d) 2-cianoethyl-*N,N*-diisopropylchlorophosphoramidite, TEA, DCM, 70%.

(R)-1-*O*-allyl-2-*O*-(2,3,4,6-*O*-benzyl-α-D-glucopyranosyl)-*sn*-glycerol (S3)

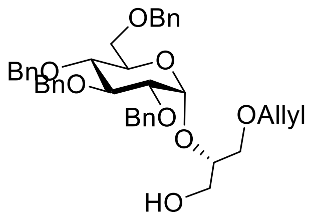

Compound **21** (12 mmol) was dissolved in dry MeOH (60 mL, 0,2 M) and a piece of $Na_{(s)}$ was added. The reaction was stirred for 1 hour, until TLC analysis (Pentane:EtOAc, 8:2) showed complete consumption of the starting material. The reaction mixture was neutralized by addition of Amberlite IR-120 (H^+ form), filtered and concentrated *in vacuo*. The product was obtained quantitatively (12 mmol) and used directly in the subsequent step without further purification.

TLC analysis: R_f= 0.32 (Pentane:EtOAc; 7:3)

^{1}H-NMR (400 MHz, $CDCl_3$), δ: 7.41-7.20 (18H, H_{arom}, m), 7.19-7.07 (2H, H_{arom}, m), 5.90-5.75 (1H, H_{allyl}, m), 5.22 (1H, CHH_{allyl}, J = 17.3, 1.7 Hz, dd), 5.14 (1H, CHH_{allyl}, J = 10.4, 1.8 Hz, dd), 4.98-4.89 (2H, H_1, CHH_{Bn}, m), 4.89-4.75 (3H, 3 x CHH_{Bn}, m), 4.67 (1H, CHH_{Bn}, J=11.6 Hz, d), 4.60 (1H, CHH_{Bn}, J=12.1 Hz, d), 4.52-4.41 (2H,2 x CHH_{Bn}, m), 4.05-3.87 (4H, H_3, H_5, $CH_{2\text{-Allyl}}$, m), 3.87-3.78 (1H, $CH_{glycerol}$, m), 3.77-3.38 (8H, 2 x H_6, H_4, H_2, 2 x $CH_{2\_glycerol}$ m), 3.17-3.04 (1H, OH, bs).

^{13}C-NMR(101 MHz, $CDCl_3$), δ: 138.8, 138.3, 138.0, 137.7 (C_q), 134.6 (CH_{allyl}), 128.7, 128.5 x 3, 128.4 x 2, 128.3, 128.2, 128.1 x 2, 128.0 x 2, 127.9, 127.8 x 2, 127.7 (CH_{arom}), 117.3 (CH_{2_allyl}), 98.8 (C_1), 82.4 (C_3), 79.9 (C_2, $CH_{glycerol}$), 77.9 (C_4), 75.7, 75.2, 74.2, 73.6 (CH_{2_Bn}), 72.4 (CH_{2_Allyl}), 70.8 (C_5), 70.3 (C_6), 68.5, 63.0 ($CH_{2\_glycerol}$).

$[\alpha]_D^{20}$($CHCl_3$): +26.7

HRMS: $C_{40}H_{46}O_8 + H^+$ required 655.3265, found 655.3271

(R)-1-*O*-allyl-2-*O*-(2,3,4,6-*O*-benzyl-α-D-glucopyranosyl)-3-*O*(4,4'-dimethoxytrityl)-*sn*-glycerol (S4)

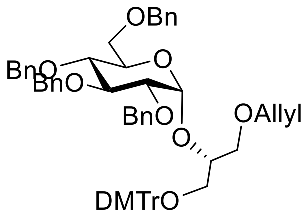

Compound **S3** (12 mmol) was dissolved in dry DCM (60mL, 0.2M) and under inert atmosphere Et_3N (18 mmol, 1.5eq) and DMTrCl (13.8 mmol, 1.15 eq) were added. The reaction mixture stirred for 3 hours until TLC analysis (Pentane:EtOAc:Et_3N, 7:3:0.1) showed complete consumption of starting material. The reaction was quenched by addition of MeOH (1 mL), diluted with DCM and washed with a 1:1 mixture of $NaHCO_3$ and brine. The aqueous layer was extracted with DCM twice and the combined organic layer were dried with $Na_2S_2O_4$, filtered and concentrated *in vacuo*. Compound **S4** was isolated in 88% yield (10.6 mmol) after column chromatography (Pentane:EtOAc:Et_3N, 97:2:1→80:19:1).

TLC analysis: R_f= 0.31 (Pentane:EtOAc; 8:2)

^{1}H-NMR (400 MHz, $CDCl_3$), δ: 7.47-7.38 (2H, H_{arom}, m), 7.37-7.04 (27H, H_{arom}, m), 6.84-6.73 (4H, H_{arom}, m), 5.90-5.73 (1H, H_{allyl}, m), 5.36-5.06 (3H, 2 x H_{allyl}, H_1, m), 4.95 (1H, CHH_{Bn}, J=10.7 Hz, d), 4.86-4.73 (2H, 2 x CHH_{Bn}, m), 4.64 (1H, CH*H*, J=12.0 Hz, d) 4.58-4.53 (2H, CHH_{Bn}, m), 4.51-4.41 (2H, 2 x CHH_{Bn}, m), 4.19-4.01 (2H, $CH_{glycerol}$, H_5, m), 4.01-3.86 (3H, H_3, CH_{2_allyl}, m), 3.81-3.43 (12H, 2 x CH_{3_OMe}, 2 x H_6, H_4, H_2, $CH_{2\_glycerol}$, m). 3.25 (2H, $CH_{2\_glycerol}$, $J_{CHH\text{-}CH}$=5.7 Hz, dd).

^{13}C-NMR(101 MHz, CD_3CN), δ: 159.6, 146.2, 140.1, 139.8, 139.6, 139.5, 137.0 x 2 (C_q), 136.1 (CH_{allyl}), 131.0, 130.0, 129.3 x 2, 129.2 x 3, 129.0, 128.9 x 2, 128.8 x 4, 128.4 x 2, 127.8 (CH_{arom}), 118.3 (CH_{2_allyl}), 114.0 (CH_{arom}), 97.0 (C_1), 82.5 (C_3), 81.0 (C_2), 78.9 (C_4), 76.8 ($CH_{glycerol}$), 76.09, 75.5, 73.9, 72.9 (CH_{2_Bn}), 72.6 (CH_{2_Allyl}), 71.4 (C_5), 71.2 (C_6), 70.1, 64.5 ($CH_{2\_glycerol}$), 55.9 (2 x CH_{3_OMe}).

$[\alpha]_D^{20}$($CHCl_3$): +21.8

HRMS: $C_{61}H_{60}O_{10}$ + Na^+ required 979.4392, found 979.4401

(R)-2-*O*-(2,3,4,6-*O*-benzyl-α-D-glucopyranosyl)-3-*O*(4,4'-dimethoxytrityl)-*sn*-glycerol (S5)

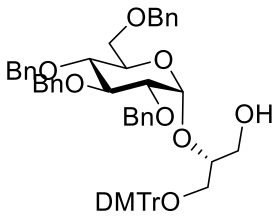

Compound **S4** (10.2 mmol) was dissolved in freshly distilled dry THF (68 mL, 0.15 M). After bubbling $Ar_{(g)}$ for 20 minutes, Ir(COD)(PPh_2Me)PF_6 (0,1 mmol, 0.01 eq) was added to the reaction mixture. $Ar_{(g)}$was bubbled for 10 minutes, followed by $H_{2(g)}$ purge for not more than 10 seconds, after which a change in the catalyst colour was observed from red to yellow. After 1 hour TLC analysis (Pentane:Toluene:EtOAc, 85:5:10) showed complete conversion of the starting material to the isomerized intermediate. The reaction mixture was diluted with THF (20 mL) and a sat. aq. solution of $NaHCO_3$ (20 mL) was added together with I_2 (15.9 mmol, 1.6 eq). TLC analysis showed complete consumption of the isomerized intermediate after 18 hours of stirring and the reaction mixture was diluted with EtOAc and washed with $NaS_2O_{3(sat)(aq)}$, $NaHCO_{3(sat.)(aq)}$, H_2O and brine. The organic layer was dried over $Na_2S_2O_4$, filtered and concentrated *in vacuo*. The desired product **S5** was isolated after purification with column chromatography (Pentane:EtOAc:Et_3N, 70:25:5) in 92% yield (9.4 mmol) as colourless syrup.

TLC analysis: R_f= 0.31 (Pentane:EtOAc; 8:2)

^{1}H-NMR (400 MHz, CD_3CN), δ: 7.50-7.44 (2H, H_{arom}, m), 7.38-7.13 (27H, H_{arom}, m), 6.84-6.74 (4H, H_{arom}, m), 5.17 (1H, H_1, J=3.6 Hz, d), 4.92 (1H, CHH_{Bn}, J=11.0 Hz, d), 4.83-4.74 (2H, 2 x CHH_{Bn}, m), 4.64-4.47 (5H, CHH_{Bn}, m), 4.05-3.98 (1H, H_5, m), 3.94-3.82 (2H, H_3, $CH_{glycerol}$, m), 3.75-3.63 (9H, 2 x CH_{3_OMe}, 2 x H_6, CH$H_{glycerol}$, m). 3.62-3.53 (1H, CH$H_{glycerol}$, m), 3.53-3.44 (2H, H_4, H_2, m), 3.25-3.13 (2H, $CH_{2\_glycerol}$, m), 3.04-2.97 (1H, OH, m).

^{13}C-NMR(101 MHz, CD_3CN), δ: 158.6, 145.3, 139.2, 138.7, 138.5 x 2, 136.1, 136.0 (C_q), 130.1 x 2, 128.4, 128.3 x 2, 128.1, 128.0, 127.9 x 2, 127.8, 127.7, 127.6 x 2, 127.5, 126.8 (CH_{arom}), 96.3 (C_1), 81.6 (C_3), 80.1 (C_2), 78.9 ($CH_{glycerol}$), 78.1 (C_4), 75.1, 74.7, 72.9, 72.0 (CH_{2_Bn}), 70.6 (C_5), 69.1 (C_6), 63.6, 62.5 ($CH_{2\_glycerol}$), 54.9 (2 x CH_3).

$[\alpha]_D^{20}$($CHCl_3$): +27.3

HRMS: $C_{58}H_{60}O_{10}$ + Na^+ required 939.4079, found 939.4090

(S)-1-*O*-([*N,N*-diisopropyl]-2-cyanoethyl-phosphoramidite)-2-*O*-(2,3,4,6-*O*-benzyl-α-D-glucopyranosyl)-3-*O*(4,4'-dimethoxytrityl)-*sn*-glycerol (7)

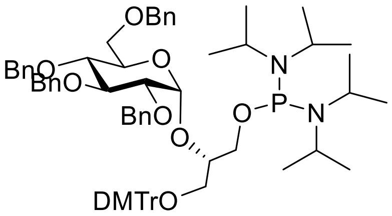

Compound **S5** (8.5 mmol) was dissolved in dry DCM (85 mL, 0.1 M) and Et_3N (12.75 mmol, 1.5 eq) was added. At 0 °C 2-cyanoethyl-*N,N*-diisopropylchlorophosphoramidite (10.2 mmol, 1.2 eq) was added and the reaction was left for 2 hours after which TLC analysis (Pentane:EtOAc:Et_3N, 7:3:0,1) showed complete consumption of the starting material. After diluting the reaction mixture with DCM, a wash with a mixture of $NaHCO_3$ and brine (1:1) was performed and the organic layer was dried over $Na_2S_2O_4$, filtered and concentrated *in vacuo*. The desired product was purified by column chromatography (Pentane:EtOAc:Et_3N, 90:19:1 → 75:25:0), affording compound **7** in 70% (5.95 mmol) as a colourless oil.

Analytical data in accordance with the one reported in W. F. J. Hogendorf, L. J. van den Bos, H. S. Overkleeft, J. D. C. Codee, G. A. van der Marel, *Bioorg, Med. Chem.,* **2010**, 18, 3668-3678.

Phosphoramidite couplings

General procedure

The starting material alcohol is co-evaporated three times with dry ACN. Once dissolved in dry ACN (0.1M), a solution of DCI in ACN (0.25 M, 1.5-2.5 eq) is added together with 3Å MS and the reaction mixture is stirred for 15 min at room temperature. A solution of phosphoramidite **7** or **8** (0.176 M in ACN) is added (1.2-2.0 eq) under inert atmosphere. After TLC analysis shows complete consumption of starting material, a solution of CSO (0.5 M in ACN) is added (2.0-3.0 eq) and the reaction is allowed to stir at r.t. for 15 min, after which the reaction is diluted with EtOAc and washed once with a mixture of $NaHCO_3$ and brine (1:1). The organic layer is dried over $Na_2S_2O_4$, filtered and concentrated *in vacuo*. The crude is then dissolved in DCM (0.1 M) and a solution of TCA (0.18 M in DCM) is added (5 eq). Once TLC analysis show complete conversion to a lower running spot, the reaction mixture is diluted in DCM and washed with a solution of $NaHCO_3$ and brine (1:1), dried over $Na_2S_2O_4$, filtered and concentrated *in vacuo*. The desired product is isolated by column chromatography.

List of intermediates from phoshoramidite couling

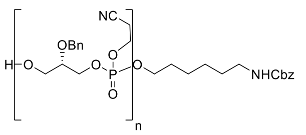

n=1, **S6**
n=2, **S7**
n=3, **S8**
n=4, **S9**
n=5, **S10**

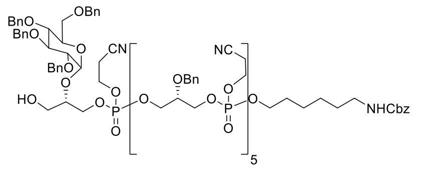

23

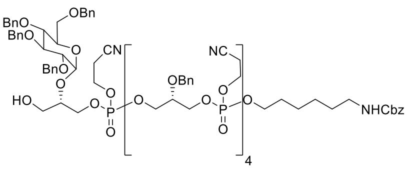

S11

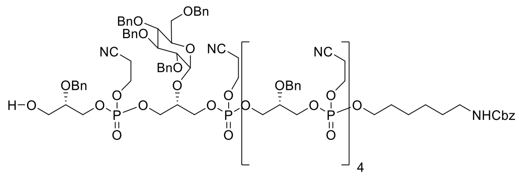

24

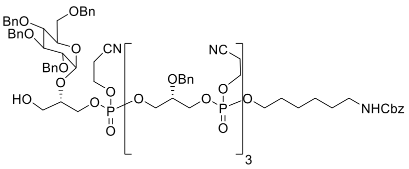

S12

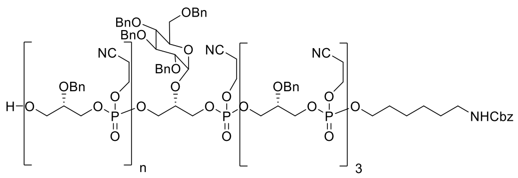

n=1, **S13**
n=2, **25**

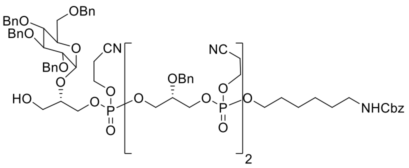

S14

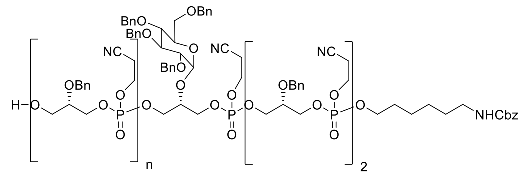

n=1, **S15**
n=2, **S16**
n=3, **26**

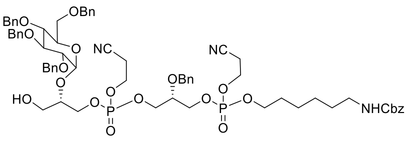

S17

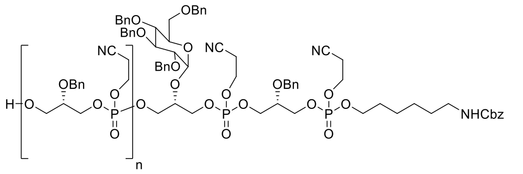

n=1, **S18**
n=2, **S19**
n=3, **S20**
n=4, **27**

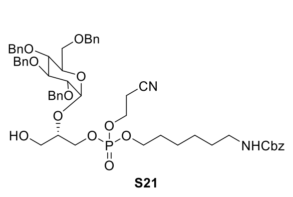

S21

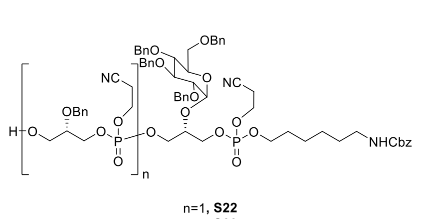

n=1, **S22**
n=2, **S23**
n=3, **S24**
n=4, **S25**
n=5, **28**

(Protected) (GroP)-Spacer or Monomer S6

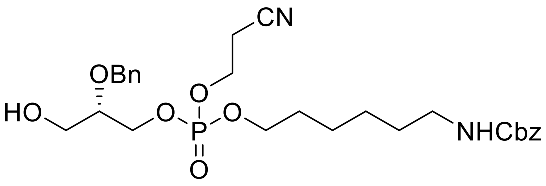

Alchol spacer **9** (1.1 mmol) was coupled with phospharamidite **8** (1.67 mmol, 1.5 eq) following the general procedure. Compound **S6** was obtained after column chromatography (DCM:Acetone, 7.5:2.5) in 90% yield (0.99 mmol).

TLC analysis, R_f:0.48 (DCM:Acetone, 7:3)

^{1}H-NMR (400 MHz, CD_3CN), δ: 7.42-7.23 (10H, H_{arom}, m), 5.68-5.54 (1H, NH, b), 5.03 (2H, CH_{2_Cbz}, s), 4.63 (2H, CH_{2_Bn}, s), 4.25-3.97 (6H, CH_{2_OCE}, $CH_{2\_glycerol}$, $CH_{2\_Ospacer}$, m), 3.71-3.54 (3H, $CH_{glycerol}$, $CH_{2\_glycerol}$, m), 3.07 (2H, $CH_{2\_Nspacer}$, J=6.6 Hz, q), 3.02-2.92 (1H, OH, b), 2.78-2.68 (2H, CH_{2_OCE}, m), 1.68-1.57 (2H, CH_{2_spacer}, m), 1.51-1.23 (6H, 3 x CH_{2_spacer}, m).

^{13}C-NMR(101 MHz, CD_3CN), δ: 157.4, 139.6, 138.4 (C_q), 129.4, 129.2, 128.8, 128.7, 128.6, 128.5 (CH_{arom}), 118.6 (C_q), 79.2-79.1 ($CH_{glycerol}$), 72.4 (CH_{2_Bn}), 69.0 ($CH_{2\_Ospacer}$), 67.6-67.5 ($CH_{2\_glycerol}$), 66.7 (CH_{2_Cbz}), 63.2-63.1 (CH_{2_OCE}), 61.1 ($CH_{2\_glycerol}$), 41.4 ($CH_{2\_Nspacer}$), 30.7, 30.4, 26.8, 25.7 (CH_{2_spacer}), 20.2-20.1 (CH_{2_OCE}).

^{31}P-NMR(162 MHz, CD_3CN), δ: -1.32, -1.29

HRMS: $C_{27}H_{37}N_2O_8P + H^+$ required 549.2360, found 549.2361

(Protected) $(GroP)_2$-Spacer or Dimer S7

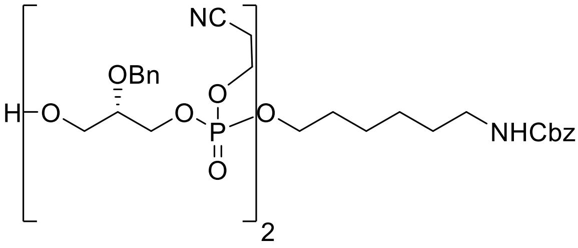

Alchol **S6** (0.75 mmol) was coupled with phospharamidite **8** (1.1 mmol, 1.5 eq) following the general procedure. Compound **S7** was obtained after column chromatography (DCM:Acetone, 6.5:3.5) in quantitave yield (0.75 mmol).

TLC analysis, R_f: 0.43 (DCM:Acetone, 6:4)

^{1}H-NMR (400 MHz, CD_3CN), δ: 7.47-7.23 (15H, H_{arom}, m), 5.95-5.86 (1H, NH, b), 5.06 (2H, CH_{2_Cbz}, s), 4.71-4.58 (4H, CH_{2_Bn}, m), 4.34-3.98 (12H, 2 x CH_{2_OCE}, 3 x $CH_{2\_glycerol}$, $CH_{2\_Ospacer}$, m), 3.93-3.81 (1H, $CH_{glycerol}$, m), 3.72-3.56 (3H, $CH_{glycerol}$, $CH_{2\_glycerol}$, m), 3.50-3.35 (1H, OH, b), 3.09 (2H, $CH_{2\_Nspacer}$, J=6.6 Hz, q), 2.77-2.67 (2H, 2 x CH_{2_OCE}, m), 1.71-1.55 (2H, CH_{2_spacer}, m), 1.52-1.22 (6H, 3 x CH_{2_spacer}, m).

^{13}C-NMR(101 MHz, CD_3CN), δ: 157.3, 139.6, 139.1 (C_q), 129.4, 129.3, 128.9, 128.8, 128.7, 128.5 (CH_{arom}), 118.6 (C_q), 79.1-79.0 ($CH_{glycerol}$), 76.8-76.7 ($CH_{glycerol}$), 72.7, 72.4 (CH_{2_Bn}), 69.1-69.0 ($CH_{2\_Ospacer}$), 67.0-66.6 ($CH_{2\_glycerol}$, CH_{2_Cbz}), 63.5-63.2 (CH_{2_OCE}), 61.1 ($CH_{2\_glycerol}$), 41.4 ($CH_{2\_Nspacer}$), 30.7, 30.4, 26.8, 25.7 (CH_{2_spacer}), 20.2-20.1 (CH_{2_OCE}).

^{31}P-NMR(162 MHz, CD_3CN), δ: -0.34, -0.33, -0.31, -0.13, -0.10.

HRMS $C_{40}H_{53}N_3O_{13}P_2 + H^+$ required 846.3126, found 846.3119

(Protected) $(GroP)_3$-Spacer or Trimer S8

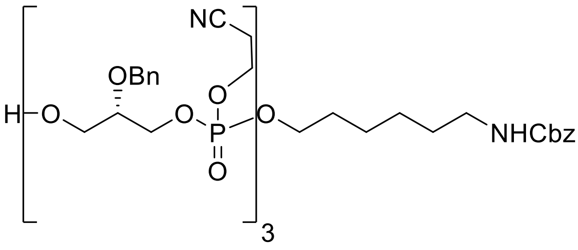

Alchol **S7** (0.83 mmol) was coupled with phospharamidite **8** (1.4 mmol, 1.7 eq) following the general procedure. Compound **S8** was obtained after column chromatography (DCM:Acetone, 6:4) in 97% yield (0.80 mmol).

TLC analysis, R_f: 0.39 (DCM:Acetone, 6.5:3.5)

^{1}H-NMR (400 MHz, CD_3CN), δ: 7.47-7.23 (20H, H_{arom}, m), 5.95-5.86 (1H, NH, b), 5.06 (2H, CH_{2_Cbz}, s), 4.71-4.58 (6H, CH_{2_Bn}, m), 4.34-3.98 (18H, 3 x CH_{2_OCE}, 5 x $CH_{2\_glycerol}$, $CH_{2\_Ospacer}$, m), 3.93-3.81 (2H, 2 x $CH_{glycerol}$, m), 3.72-3.56 (3H, $CH_{glycerol}$, $CH_{2\_glycerol}$, m), 3.09 (2H, $CH_{2\_Nspacer}$, J=6.6 Hz, q), 3.10-2.94 (1H, OH, b), 2.77-2.67 (6H, 3 x CH_{2_OCE}, m), 1.71-1.55 (2H, CH_{2_spacer}, m), 1.52-1.22 (6H, 3 x CH_{2_spacer}, m).
^{13}C-NMR(101 MHz, CD_3CN), δ: 157.3, 139.6, 139.1 (C_q), 129.4, 129.3, 128.9, 128.8, 128.7, 128.5 (CH_{arom}), 118.6 (C_q), 79.1-79.0 ($CH_{glycerol}$), 76.8-76.7 ($CH_{glycerol}$), 72.7, 72.4 (CH_{2_Bn}), 69.1-69.0 ($CH_{2\_Ospacer}$), 67.0-66.6 ($CH_{2\_glycerol}$, CH_{2_Cbz}), 63.5-63.2 (CH_{2_OCE}), 61.1 ($CH_{2\_glycerol}$), 41.4 ($CH_{2\_Nspacer}$), 30.7, 30.4, 26.8, 25.7 (CH_{2_spacer}), 20.2-20.1 (CH_{2_OCE}).
^{31}P-NMR(162 MHz, CD_3CN), δ: -1.48, -1.47, -1.43, -1.42, -1.41, -1.23, -1.21, -1.19.
HRMS: $C_{53}H_{69}N_4O_{18}P_3 + H^+$ required 1143.3893, found 1143.3900

(Protected) (GroP)$_4$-Spacer or Tetramer S9

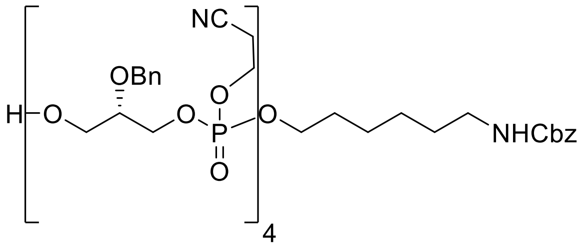

Alchol **S8** (0.12 mmol) was coupled with phospharamidite **8** (0.24 mmol, 2 eq) following the general procedure. Compound **S9** was obtained after column chromatography (DCM:Acetone, 1:1) in 83% yield (0.1 mmol).
TLC analysis, R_f:0.32 (DCM:Acetone, 1:1)
^{1}H-NMR (400 MHz, CD_3CN), δ: 7.47-7.23 (25H, H_{arom}, m), 5.82-5.69 (1H, NH, b), 5.03 (2H, CH_{2_Cbz}, s), 4.67-4.54 (8H, CH_{2_Bn}, m), 4.34-3.98 (24H, 4 x CH_{2_OCE}, 7 x $CH_{2\_glycerol}$, $CH_{2\_Ospacer}$, m), 3.93-3.81 (3H, 3 x $CH_{glycerol}$, m), 3.72-3.56 (3H, $CH_{glycerol}$, $CH_{2\_glycerol}$, m), 3.23-3.13 (1H, OH, b), 3.07 (2H, $CH_{2\_Nspacer}$, J=6.6 Hz, q), 2.77-2.67 (8H, 4 x CH_{2_OCE}, m), 1.71-1.55 (2H, CH_{2_spacer}, m), 1.52-1.22 (6H, 3 x CH_{2_spacer}, m).
^{13}C-NMR(101 MHz, CD_3CN), δ: 157.3, 139.6, 139.1 (C_q), 129.4 x 2, 129.3, 128.9 x 2, 128.8 x 2, 128.7, 128.5 (CH_{arom}), 118.6 (C_q), 79.1-79.0 ($CH_{glycerol}$), 76.8-76.7 ($CH_{glycerol}$), 72.7, 72.4 (CH_{2_Bn}), 69.1-69.0 ($CH_{2\_Ospacer}$), 67.0-66.6 ($CH_{2\_glycerol}$, CH_{2_Cbz}), 63.5-63.2 (CH_{2_OCE}), 61.1 ($CH_{2\_glycerol}$), 41.4 ($CH_{2\_Nspacer}$), 30.7, 30.4, 26.8, 25.7 (CH_{2_spacer}), 20.2-20.1 (CH_{2_OCE}).
^{31}P-NMR(162 MHz, CD_3CN), δ: -1.66, -1.64, -1.62, -1.60, -1.58, -1.40, -1.37.
HRMS: $C_{66}H_{85}N_5O_{23}P_4 + H^+$ required 1440.4659, found 1440.4656

(Protected) (GroP)$_5$-Spacer or Pentamer S10

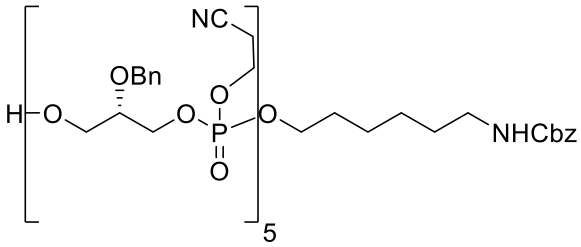

Alchol **S9** (35 μmol) was coupled with phospharamidite **8** (86 μmol, 2.5 eq) following the general procedure. Compound **S10** was obtained after column chromatography (DCM:Acetone, 1:1) in 65% yield (23 μmol).
TLC analysis, R_f: 0.27 (DCM:Acetone, 6:4)
^{1}H-NMR (400 MHz, CD_3CN), δ: 7.47-7.23 (30H, H_{arom}, m), 5.82-5.69 (1H, NH, b), 5.03 (2H, CH_{2_Cbz}, s), 4.67-4.54 (10H, CH_{2_Bn}, m), 4.34-3.98 (30H, 5 x CH_{2_OCE}, 9 x $CH_{2\_glycerol}$, $CH_{2\_Ospacer}$, m), 3.93-3.81 (4H, 4 x $CH_{glycerol}$, m), 3.72-3.56 (3H, $CH_{glycerol}$, $CH_{2\_glycerol}$, m), 3.20-3.02 (3H, OH, $CH_{2\_Nspacer}$, m), 2.77-2.67 (10H, 5 x CH_{2_OCE}, m), 1.71-1.55 (2H, CH_{2_spacer}, m), 1.52-1.22 (6H, 3 x CH_{2_spacer}, m).
^{13}C-NMR(101 MHz, CD_3CN), δ: 157.3, 139.6, 139.1 (C_q), 129.4 x 2, 129.3, 128.9 x 2, 128.8 x 2, 128.7, 128.5 (CH_{arom}), 118.6 (C_q), 79.1-79.0 ($CH_{glycerol}$), 76.8-76.7 ($CH_{glycerol}$), 72.7, 72.4 (CH_{2_Bn}), 69.1-69.0 ($CH_{2\_Ospacer}$), 67.0-66.6 ($CH_{2\_glycerol}$, CH_{2_Cbz}), 63.5-63.2 (CH_{2_OCE}), 61.1 ($CH_{2\_glycerol}$), 41.4 ($CH_{2\_Nspacer}$), 30.7, 30.4, 26.8, 25.7 (CH_{2_spacer}), 20.2-20.1 (CH_{2_OCE}).
^{31}P-NMR(162 MHz, CD_3CN), δ: -1.67, -1.64, -1.63, -1.61, -1.58, -1.44, -1.40, -1.37.

HRMS: $C_{79}H_{101}N_6O_{28}P_5 + H^+$ required 1737.5425, found 1737.5428

(Protected) (GlcGroP)(GroP)$_5$-Spacer or Hexamer 23

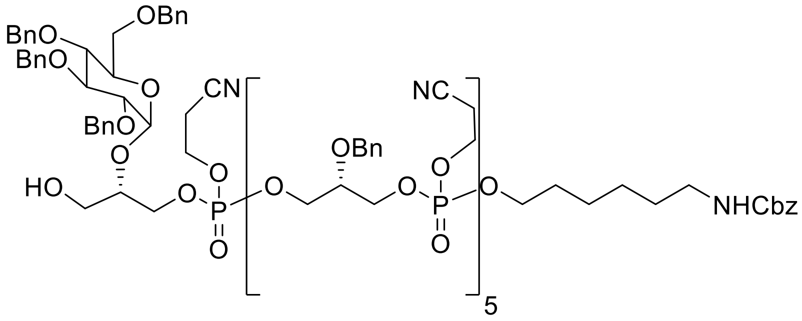

Alchol **S10** (22 μmol) was coupled with phospharamidite **7** (32 μmol, 1.5 eq) following the general procedure. Compound **23** was obtained after column chromatography (DCM:Acetone, 1:1) in 65% yield (14 μmol).

TLC analysis, R_f: 0.31 (DCM:Acetone, 1:1)

^{1}H-NMR (400 MHz, CD_3CN), δ: 7.45-7.16 (50H, H_{arom}, m), 5.79-5.69 (1H, NH, b), 5.21-5.14 (1H, H_1, m), 5.06 (2H, CH_{2_Cbz}), 4.91-4.45 (18H, CH_{2_Bn}, m), 4.31-3.99 (36H, 6 x CH_{2_OCE}, 11 x $CH_{2\_glycerol}$, $CH_{2\_Ospacer}$, m), 3.99-3.78 (8H, 6 x $CH_{\_glycerol}$, H_5, H_3, m), 3.78-3.48 (6H, 2 x H_6, H_4, H_2, $CH_{2\_glycerol}$), 3.28-3.27 (1H, OH, b), 3.09 (2H, $CH_{2\_Nspacer}$, J=6.6 Hz, q), 2.78-2.58 (12H, 6 x CH_{2_OCE}, m), 1.71-1.55 (2H, CH_{2_spacer}, m), 1.52-1.22 (6H, 3 x CH_{2_spacer}, m).

^{13}C-NMR(101 MHz, CD_3CN), δ: 157.3, 140.1, 139.8, 139.6, 139.1 (C_q), 129.4 x 2, 129.3 X 2, 129.1 x 2, 129.0, 128.9, 128.8 x 2, 128.7, 128.6, 128.4 (CH_{arom}), 118.6 (C_q), 98.4 (C_1), 82.5 (C_3), 81.0 (C_2), 78.7 (C_4), 77.9 ($CH_{glycerol}$), 77.9 ($CH_{glycerol}$), 76.0, 75.6, 73.8, 73.5, 72.7 (CH_{2_Bn}), 71.6 (C_5), 69.7 (C_6), 69.2 ($CH_{2\_Ospacer}$), 66.8-66.6 ($CH_{2\_glycerol}$, CH_{2_Cbz}), 63.6-63.5 (CH_{2_OCE}), 61.1 ($CH_{2\_glycerol}$), 41.4 ($CH_{2\_Nspacer}$), 30.9-30.8, 30.4, 26.8, 25.7 (CH_{2_spacer}), 20.2-20.1 (CH_{2_OCE}).

^{31}P-NMR(162 MHz, CD_3CN), δ: -1.66, -1.63, -1.62, -1.60, -1.58, -1.44, -1.38.

HRMS: $C_{119}H_{145}N_7O_{38}P_6 + H^+$ required 2466.8128, found 2466.8129

(Protected) (GlcGroP)(GroP)$_4$-Spacer or Pentamer S11

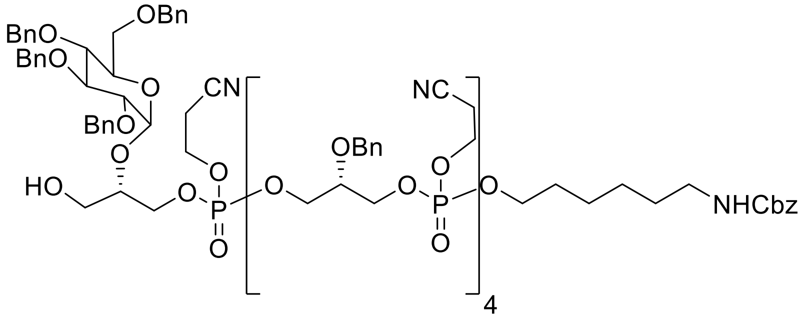

Alchol **S9** (35 μmol) was coupled with phospharamidite **7** (53 μmol, 1.5 eq) following the general procedure. Compound **S11** was obtained after column chromatography (DCM:Acetone, 1:1) in 67% yield (24 μmol).

TLC analysis, R_f: 0.33 (DCM:Acetone, 6:4)

^{1}H-NMR (400 MHz, CD_3CN), δ: 7.45-7.11 (45H, H_{arom}, m), 5.71-5.58 (1H, NH, b), 5.16 (1H, H_1, J=3.6 Hz, d), 5.01 (2H, CH_{2_Cbz}, s), 4.89-4.81 (1H, CH_{2_Bn}, m), 4.80-4.66 (3H, CH_{2_Bn}, m), 4.66-4.43 (12H, CH_{2_Bn}, m), 4.31-3.94 (30H, 5 x CH_{2_OCE}, 9 x $CH_{2\_glycerol}$, $CH_{2\_Ospacer}$, m), 3.94-3.74 (6H, 4 x $CH_{\_glycerol}$, H_5, H_3, m), 3.74-3.42 (7H, $CH_{glycerol}$, 2 x H_6, H_4, H_2, $CH_{2\_glycerol}$), 3.13-2.94 (3H, OH, $CH_{2\_Nspacer}$, m), 2.74-2.54 (10H, 5 x CH_{2_OCE}, m), 1.65-1.49 (2H, CH_{2_spacer}, m), 1.45-1.14 (6H, 3 x CH_{2_spacer}, m).

^{13}C-NMR(101 MHz, CD_3CN), δ: 157.2, 140.0, 139.6, 139.4, 139.0 (C_q), 129.4 x 2, 129.3 X 2, 129.2 x 2, 129.1, 129.0 x 2, 128.9 x 2, 128.8 x 3, 128.7 x 2, 128.6, 128.5, 128.4 (CH_{arom}), 118.6 (C_q), 97.0 (C_1), 82.3 (C_3), 80.7 (C_2), 79.0 (C_4), 78.6 ($CH_{glycerol}$), 76.7 ($CH_{glycerol}$), 76.0, 75.6, 73.8, 73.5, 73.0, 72.7, 72.4 (CH_{2_Bn}), 71.6 (C_5), 69.7 (C_6), 69.2 ($CH_{2\_Ospacer}$), 67.8-66.0 ($CH_{2\_glycerol}$, CH_{2_Cbz}), 63.5-63.3 (CH_{2_OCE}), 61.1 ($CH_{2\_glycerol}$), 41.4 ($CH_{2\_Nspacer}$), 30.7-30.4, 26.8, 25,7 (CH_{2_spacer}), 20.2-20.1 (CH_{2_OCE}).

^{31}P-NMR(162 MHz, CD_3CN), δ: -0.41, -0.36, -0.34, -0.32, -0.30, -0.29, -0.14, -0.11

HRMS: $C_{106}H_{129}N_6O_{33}P_5 + H^+$ required 2169.7361, found 2169.7368

(Protected) (GroP)(GlcGroP)(GroP)$_4$-Spacer or Hexamer 24

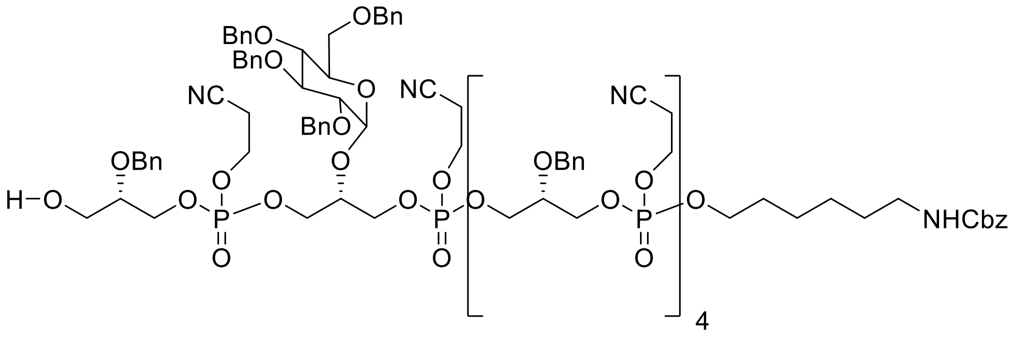

Alchol **S11** (20 μmol) was coupled with phospharamidite **8** (50 μmol, 2.5 eq) following the general procedure. Compound **24** was obtained after column chromatography (DCM:Acetone, 1:1) in 77% yield (15 μmol).

TLC analysis, R_f: 0.31 (DCM:Acetone, 1:1)

^{1}H-NMR (400 MHz, CD_3CN), δ: 7.47-7.16 (50H, H_{arom}, m), 5.71-5.58 (1H, NH, b), 5.16 (1H, H_1, J=3.6 Hz, d), 5.01 (2H, CH_{2_Cbz}, s), 4.89-4.66 (4H, CH_{2_Bn}, m), 4.66-4.43 (14H, CH_{2_Bn}, m), 4.30-3.94 (36H, 6 x CH_{2_OCE}, 11 x $CH_{2\_glycerol}$, $CH_{2\_Ospacer}$, m), 3.94-3.74 (7H, 5 x $CH_{\_glycerol}$, H_5, H_3, m), 3.74-3.42 (7H, $CH_{glycerol}$, 2 x H_6, H_4, H_2, $CH_{2\_glycerol}$), 3.13-2.94 (3H, OH, $CH_{2\_Nspacer}$, m), 2.74-2.54 (12H, 6 x CH_{2_OCE}, m), 1.65-1.49 (2H, CH_{2_spacer}, m), 1.45-1.14 (6H, 3 x CH_{2_spacer}, m).

^{13}C-NMR(101 MHz, CD_3CN), δ: 157.2, 140.1, 139.6 x 2, 139.5, 139.0 (C_q), 129.4 x 2, 129.3 X 2, 129.2 x 2, 129.1, 129.0 x 4, 128.9 x 2, 128.8 x 3, 128.7 x 2, 128.6, 128.5 x 2, 128.4 (CH_{arom}), 118.6 (C_q), 97.0 (C_1), 82.3 (C_3), 80.7 (C_2), 79.1-79.0 ($CH_{glycerol}$), 78.6 (C_4), 76.7 ($CH_{glycerol}$), 76.0, 75.6, 73.8, 73.0, 72.7, 72.4 (CH_{2_Bn}), 71.7 (C_5), 69.7 (C_6), 69.2 ($CH_{2\_Ospacer}$), 67.8-66.6 ($CH_{2\_glycerol}$, CH_{2_Cbz}), 63.6-63.3 (CH_{2_OCE}), 61.1 ($CH_{2\_glycerol}$), 41.4 ($CH_{2\_Nspacer}$), 30.7-30.4, 26.8, 25,7 (CH_{2_spacer}), 20.2-20.1 (CH_{2_OCE}).

^{31}P-NMR(162 MHz, CD_3CN), δ: -0.44, -0.43, -0.40, -0.38, -0.34, -0.32, -0.18, -0.15.

HRMS: $C_{119}H_{145}N_7O_{38}P_6 + H^+$ required 2466.8128, found 2466.8133

(Protected) (GlcGroP)(GroP)$_3$-Spacer or Tetramer S12

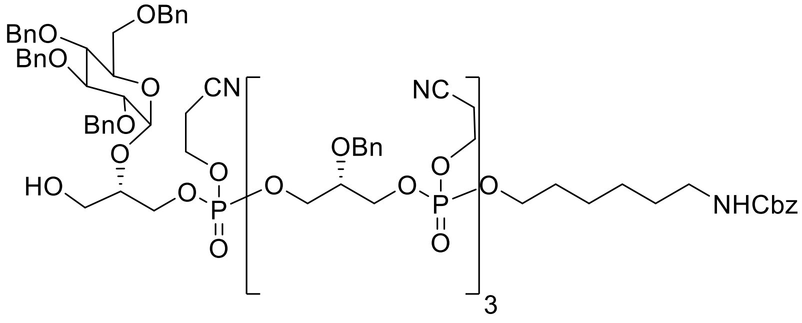

Alchol **S8** (59 μmol) was coupled with phospharamidite **7** (88 μmol, 1.5 eq) following the general procedure. Compound **S12** was obtained after column chromatography (DCM:Acetone, 5.5:4.5) in 86% yield (51 μmol).

TLC analysis, R_f: 0.31 (DCM:Acetone, 1:1)

^{1}H-NMR (400 MHz, CD_3CN), δ: 7.50-7.08 (40H, H_{arom}, m), 5.77-5.64 (1H, NH, b), 5.18-5.12 (1H, H_1, m), 5.03 (2H, CH_{2_Cbz}, s), 4.92-4.39 (14H, CH_{2_Bn}, m), 4.31-3.97 (34H, 4 x CH_{2_OCE}, 7 x $CH_{2\_glycerol}$, $CH_{2\_Ospacer}$, m), 3.94-3.75 (6H, 4 x $CH_{\_glycerol}$, H_5, H_3, m), 3.74-3.45 (6H, 2 x H_6, H_4, H_2, $CH_{2\_glycerol}$), 3.26-3.16 (1H, OH, b), 3.06 (2H, $CH_{2\_Nspacer}$, J=6.6 Hz, q), 2.78-2.50 (8H, 4 x CH_{2_OCE}, m), 1.65-1.49 (2H, CH_{2_spacer}, m), 1.45-1.14 (6H, 3 x CH_{2_spacer}, m).

^{13}C-NMR(101 MHz, CD_3CN), δ: 157.3, 140.0, 139.6, 139.5, 139.4, 139.1 (C_q), 129.4 x 2, 129.3 X 2, 129.2, 129.1 x 2, 129.0, 128.9 x 3, 128.8 x 3, 128.7 x 2, 128.6, 128.5, 128.4 (CH_{arom}), 118.6 (C_q), 97.4 (C_1), 82.5 (C_3), 80.9 (C_2), 78.9 (C_4), 78.0-77.8 ($CH_{glycerol}$), 76.8-76.7 ($CH_{glycerol}$), 75.9, 75.6, 73.8, 73.5, 72.7 (CH_{2_Bn}), 71.6 (C_5), 69.7 (C_6), 69.1 ($CH_{2\_Ospacer}$), 68.8-66.06($CH_{2\_glycerol}$, CH_{2_Cbz}), 63.5-63.2 (CH_{2_OCE}), 61.1 ($CH_{2\_glycerol}$), 41.4 ($CH_{2\_Nspacer}$), 30.8-30.4, 26.8, 25,7 (CH_{2_spacer}), 20.3-20.1 (CH_{2_OCE}).

^{31}P-NMR(162 MHz, CD_3CN), δ: -0.39, -0.37, -0.35, -0.33, -0.31, -0.17, -0.10.

HRMS: $C_{93}H_{113}N_5O_{28}P_4 + H^+$ required 1872.6595, found 1872.6603

(Protected) (GroP)(GlcGroP)(GroP)$_3$-Spacer or Pentamer S13

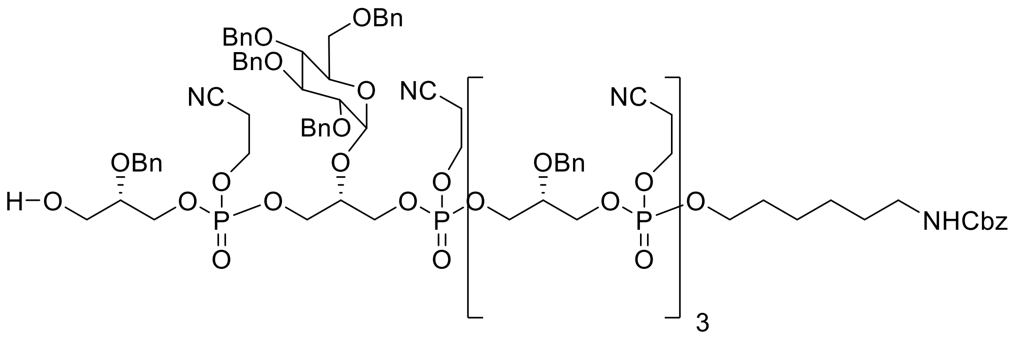

Alchol **S12** (40 µmol) was coupled with phospharamidite **8** (100 µmol, 2.5 eq) following the general procedure. Compound **S13** was obtained after column chromatography (DCM:Acetone, 1:1) in 76% yield (30 µmol).

TLC analysis, R_f: 0.38 (DCM:Acetone, 4:6)

^{1}H-NMR (400 MHz, CD_3CN), δ: 7.47-7.16 (45H, H_{arom}, m), 5.74-5.61 (1H, NH, b), 5.16 (1H, H_1, J=3.6 Hz, d), 5.01 (2H, CH_{2_Cbz}, s), 4.89-4.41 (18H, CH_{2_Bn}, m), 4.30-3.93 (30H, 5 x CH_{2_OCE}, 9 x $CH_{2\_glycerol}$, $CH_{2\_Ospacer}$, m), 3.94-3.74 (5H, 3 x $CH_{\_glycerol}$, H_5, H_3, m), 3.74-3.42 (7H, $CH_{glycerol}$, 2 x H_6, H_4, H_2, $CH_{2\_glycerol}$), 3.22-3.10 (1H, OH, m), 3.08-2.94 (2H, $CH_{2\_Nspacer}$, m), 2.74-2.53 (10H, 5 x CH_{2_OCE}, m), 1.65-1.49 (2H, CH_{2_spacer}, m), 1.45-1.14 (6H, 3 x CH_{2_spacer}, m).

^{13}C-NMR(101 MHz, CD_3CN), δ: 157.2, 140.0, 139.6, 139.4, 139.0 (C_q), 129.4 x 2, 129.3 X 2, 129.2 x 2, 129.1, 129.0 x 2, 128.9 x 2, 128.8 x 3, 128.7 x 2, 128.6, 128.5, 128.4 (CH_{arom}), 118.6 (C_q), 97.0 (C_1), 82.3 (C_3), 80.7 (C_2), 79.0 (C_4), 78.6 ($CH_{glycerol}$), 76.7 ($CH_{glycerol}$), 76.0, 75.6, 73.8, 73.5, 73.0, 72.7, 72.4 (CH_{2_Bn}), 71.6 (C_5), 69.7 (C_6), 69.2 ($CH_{2\_Ospacer}$), 67.8-66.0 ($CH_{2\_glycerol}$, CH_{2_Cbz}), 63.5-63.3 (CH_{2_OCE}), 61.1 ($CH_{2\_glycerol}$), 41.4 ($CH_{2\_Nspacer}$), 30.7-30.4, 26.8, 25,7 (CH_{2_spacer}), 20.2-20.1 (CH_{2_OCE}).

^{31}P-NMR(162 MHz, CD_3CN), δ: -0.41, -0.36, -0.34, -0.32, -0.30, -0.29, -0.14, -0.11

HRMS: $C_{106}H_{129}N_6O_{33}P_5 + H^+$ required 2169.7361, found 2169.7355

(Protected) (GroP)$_2$(GlcGroP)(GroP)$_3$-Spacer or Hexamer 25

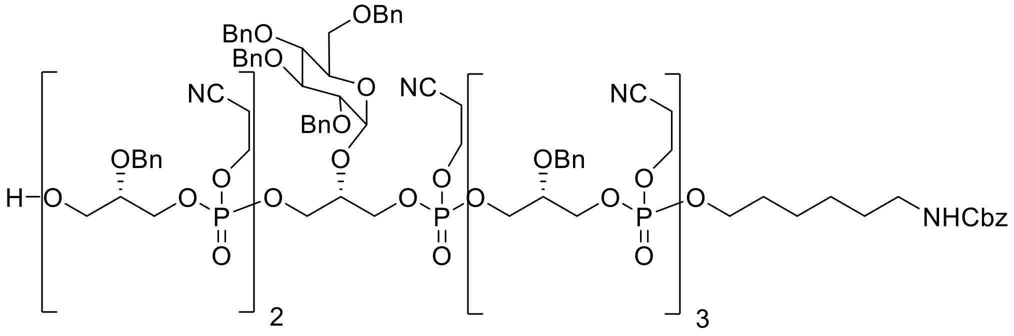

Alchol **S13** (10 µmol) was coupled with phospharamidite **8** (25 µmol, 2.5 eq) following the general procedure. Compound **25** was obtained after column chromatography (DCM:Acetone, 1:1) in 72% yield (7.2 µmol).

TLC analysis, R_f: 0.31 (DCM:Acetone, 1:1)

^{1}H-NMR (400 MHz, CD_3CN), δ: 7.50-7.16 (50H, H_{arom}, m), 5.76-5.65 (1H, NH, b), 5.20-5.12 (1H, H_1, m), 5.02 (2H, CH_{2_Cbz}, s), 4.89-4.39 (18H, CH_{2_Bn}, m), 4.30-3.94 (36H, 6 x CH_{2_OCE}, 11 x $CH_{2\_glycerol}$, $CH_{2\_Ospacer}$, m), 3.94-3.74 (7H, 5 x $CH_{\_glycerol}$, H_5, H_3, m), 3.74-3.42 (7H, $CH_{glycerol}$, 2 x H_6, H_4, H_2, $CH_{2\_glycerol}$), 3.17-2.98 (3H, OH, $CH_{2\_Nspacer}$, m), 2.77-2.48 (12H, 6 x CH_{2_OCE}, m), 1.65-1.49 (2H, CH_{2_spacer}, m), 1.45-1.14 (6H, 3 x CH_{2_spacer}, m).

^{13}C-NMR(101 MHz, CD_3CN), δ: 157.2, 140.0, 139.6, 139.5, 139.1 (C_q), 129.4 x 3, 129.3, 129.2 x 2, 129.1, 129.0, 128.9, 128.8 x 2,128.7, 128.6, 128.4 (CH_{arom}), 118.6 (C_q), 98.1 (C_1), 82.3 (C_3), 80.7 (C_2), 79.1 ($CH_{glycerol}$), 78.6 (C_4), 76.8 ($CH_{glycerol}$), 76.0, 75.7, 73.8, 73.1, 72.7, 72.4 (CH_{2_Bn}), 71.7 (C_5), 69.7 (C_6), 69.1 ($CH_{2\_Ospacer}$), 67.8-66.9 ($CH_{2\_glycerol}$, CH_{2_Cbz}), 63.5-63.2 (CH_{2_OCE}), 61.1 ($CH_{2\_glycerol}$), 41.4 ($CH_{2\_Nspacer}$), 30.7-30.4, 26.8, 25,7 (CH_{2_spacer}), 20.2-20.1 (CH_{2_OCE}).

^{31}P-NMR(162 MHz, CD_3CN), δ: -1.77, -1.71, -1.68, -1.63, -1.59, -1.56, -1.43, -1.13, -1.11, -1.03, -1.01.

HRMS: $C_{119}H_{145}N_7O_{38}P_6 + H^+$ required 2466.8128, found 2466.8137

(Protected) (GlcGroP)(GroP)$_2$-Spacer or Trimer S14

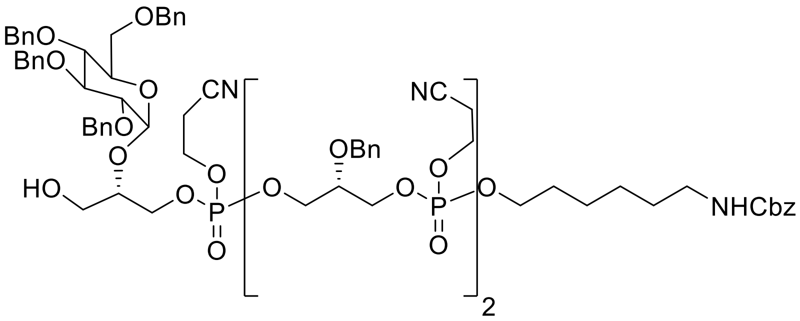

Alchol **S7** (98 μmol) was coupled with phospharamidite **7** (147 μmol, 1.5 eq) following the general procedure. Compound **S14** was obtained after column chromatography (DCM:Acetone, 5.5:4.5) in 86% yield (84 μmol).
TLC analysis, R_f: 0.35 (DCM:Acetone, 6:4)
^{1}H-NMR (400 MHz, CD_3CN), δ: 7.44-7.11 (35H, H_{arom}, m), 5.77-5.62 (1H, NH, b), 5.17-5.11 (1H, H_1, m), 5.03 (2H, CH_{2_Cbz}, s), 4.90-4.41 (12H, 6 x CH_{2_Bn}, m), 4.29-3.96 (18H, 3 x CH_{2_OCE}, 5 x $CH_{2\_glycerol}$, $CH_{2\_Ospacer}$, m), 3.96-3.75 (5H, 3 x $CH_{\_glycerol}$, H_5, H_3, m), 3.74-3.42 (6H, 2 x H_6, H_4, H_2, $CH_{2\_glycerol}$), 3.26-3.14 (1H, OH, b), 3.05 (2H, $CH_{2\_Nspacer}$, J=6.6 Hz, q), 2.77-2.53 (6H, 3 x CH_{2_OCE}, m), 1.65-1.49 (2H, CH_{2_spacer}, m), 1.45-1.14 (6H, 3 x CH_{2_spacer}, m).
^{13}C-NMR(101 MHz, CD_3CN), δ: 157.4, 140.1, 139.6, 139.5, 139.4, 139.1, 139.0, 138.6 (C_q), 129.4 x 3, 129.3 x 2, 129.2, 129.1, 129.0, 128.9, 128.8 x 2,128.7, 128.6, 128.4 (CH_{arom}), 118.6 (C_q), 97.4 (C_1), 82.5 (C_3), 81.0 (C_2), 78.7 (C_4), 78.0, 76.0 ($CH_{glycerol}$), 76.0, 75.6 x 2, 73.8, 73.6, 72.7, 72.4 (CH_{2_Bn}), 71.6 (C_5), 69.7 (C_6), 69.1 ($CH_{2\_Ospacer}$), 68.3, 67.8-66.9 ($CH_{2\_glycerol}$, CH_{2_Cbz}), 63.5-63.2 (CH_{2_OCE}), 61.1 ($CH_{2\_glycerol}$), 41.4 ($CH_{2\_Nspacer}$), 30.7-30.4, 26.8, 25,7 (CH_{2_spacer}), 20.2-20.1 (CH_{2_OCE}).
^{31}P-NMR(162 MHz, CD_3CN), δ: -1.67, -1.65, -1.64, -1.62, -1.61, -1.58, -1.45, -1.42, -1.39, -1.38
HRMS: $C_{80}H_{97}N_4O_{23}P_3 + H^+$ required 1575.5829, found 1575.5833

(Protected) (GroP)(GlcGroP)(GroP)$_2$-Spacer or Tetramer S15

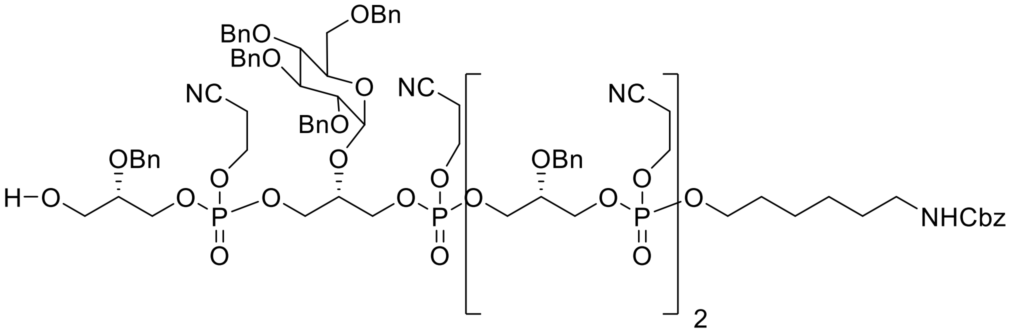

Alchol **S14** (11 μmol) was coupled with phospharamidite **8** (28 μmol, 2.5 eq) following the general procedure. Compound **S15** was obtained after column chromatography (DCM:Acetone, 1:1) in 83% yield (9.1 μmol).
TLC analysis, R_f: 0.31 (DCM:Acetone, 1:1)
^{1}H-NMR (400 MHz, CD_3CN), δ: 7.48-7.11 (40H, H_{arom}, m), 5.73-5.62 (1H, NH, b), 5.19-5.13 (1H, H_1, m), 5.03 (2H, CH_{2_Cbz}, s), 4.89-4.42 (14H, 7 x CH_{2_Bn}, m), 4.30-3.96 (24H, 4 x CH_{2_OCE}, 7 x $CH_{2\_glycerol}$, $CH_{2\_Ospacer}$, m), 3.96-3.75 (5H, 3 x $CH_{\_glycerol}$, H_5, H_3, m), 3.74-3.42 (7H, $CH_{glycerol}$, 2 x H_6, H_4, H_2, $CH_{2\_glycerol}$), 3.15-3.00 (3H, OH, $CH_{2\_Nspacer}$, m), 2.77-2.53 (8H, 4 x CH_{2_OCE}, m), 1.65-1.49 (2H, CH_{2_spacer}, m), 1.45-1.14 (6H, 3 x CH_{2_spacer}, m).
^{13}C-NMR(101 MHz, CD_3CN), δ: 157.3, 139.7, 139.6, 139.5, 139.1 (C_q), 129.4 x 3, 129.3 x 2, 129.2, 129.1 x 2, 129.0 x 4, 128.9 x 2, 128.8 x 3,128.7 x 2, 128.6 x 2, 128.4 (CH_{arom}), 118.6 (C_q), 97.1 (C_1), 82.3 (C_3), 80.8 (C_2), 79.2-79.1 ($CH_{gltcerol}$), 78.6 (C_4), 76.9-76.8 ($CH_{glycerol}$), 76.0, 75.6 x 2, 73.9, 73.1, 72.4 (CH_{2_Bn}), 71.8 (C_5), 69.7 (C_6), 69.1 ($CH_{2\_Ospacer}$), 67.9, 67.8, 67.0, 66.7, 66.6, 66.5 ($CH_{2\_glycerol}$, CH_{2_Cbz}), 63.6-63.3 (CH_{2_OCE}), 61.2 ($CH_{2\_glycerol}$), 41.4 ($CH_{2\_Nspacer}$), 30.7-30.4, 26.8, 25,7 (CH_{2_spacer}), 20.2-20.1 (CH_{2_OCE}).

^{31}P-NMR(162 MHz, CD_3CN), δ: -1.71, -1.69, -1.65, -1.63, -1.61, -1.59, -1.55, -1.53, -1.43, -1.42, -1.39, -1.36
HRMS: $C_{93}H_{113}N_5O_{28}P_4$ + H^+ required 1872.6595, found 1872.6594

(Protected) (GroP)$_2$(GlcGroP)(GroP)$_2$-Spacer or Pentamer S16

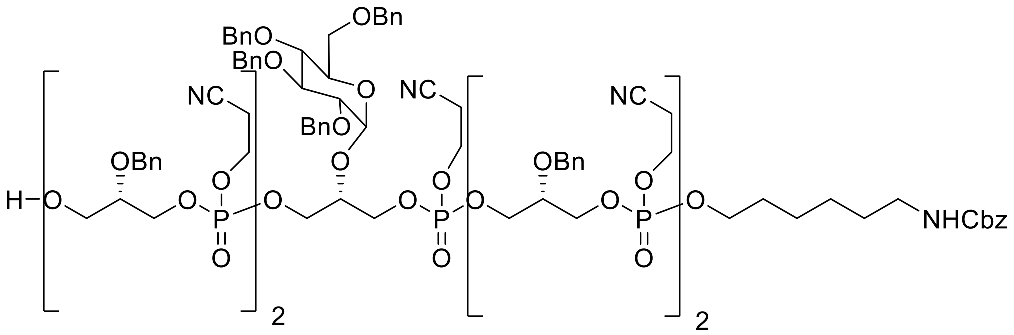

Alchol **S15** (24 μmol) was coupled with phospharamidite **8** (60 μmol, 2.5 eq) following the general procedure. Compound **S16** was obtained after column chromatography (DCM:Acetone, 1:1) in 82% yield (19 μmol).
TLC analysis, R_f: 0.38 (DCM:Acetone, 4:6)
^{1}H-NMR (400 MHz, CD_3CN), δ: 7.48-7.12 (45H, H_{arom}, m), 5.77-5.64 (1H, NH, b), 5.20-5.13 (1H, H_1, m), 5.02 (2H, CH_{2_Cbz}, s), 4.87-4.40 (16H, 7 x CH_{2_Bn}, m), 4.30-3.94 (30H, 5 x CH_{2_OCE}, 9 x $CH_{2\_glycerol}$, $CH_{2\_Ospacer}$, m), 3.94-3.74 (5H, 3 x $CH_{\_glycerol}$, H_5, H_3, m), 3.74-3.42 (7H, $CH_{glycerol}$, 2 x H_6, H_4, H_2, $CH_{2\_glycerol}$), 3.19-2.99 (3H, OH, $CH_{2\_Nspacer}$, m), 2.77-2.53 (10H, 5 x CH_{2_OCE}, m), 1.65-1.49 (2H, CH_{2_spacer}, m), 1.45-1.14 (6H, 3 x CH_{2_spacer}, m).
^{13}C-NMR(101 MHz, CD_3CN), δ: 157.3, 140.1, 139.6 x 2, 139.5, 139.0 (C_q), 129.4 x 2, 129.3 x 2, 129.2 x 2, 129.1, 129.0 x 4, 128.9 x 2, 128.8 x 3,128.7 x 3, 128.6, 128.5 x 2, 128.4 (CH_{arom}), 118.6 (C_q), 97.0 (C_1), 82.3 (C_3), 80.7 (C_2), 79.1-79.0 ($CH_{gltcerol}$), 78.6 (C_4), 76.7 ($CH_{glycerol}$), 76.0, 75.6, 73.8, 73.1, 72.7, 72.4 (CH_{2_Bn}), 71.7 (C_5), 69.7 (C_6), 69.2 ($CH_{2\_Ospacer}$), 67.8-66.6 ($CH_{2\_glycerol}$, CH_{2_Cbz}), 63.6-63.3 (CH_{2_OCE}), 61.1 ($CH_{2\_glycerol}$), 41.4 ($CH_{2\_Nspacer}$), 30.7-30.4, 26.8, 25,7 (CH_{2_spacer}), 20.2-20.1 (CH_{2_OCE}).
^{31}P-NMR(162 MHz, CD_3CN), δ: -1.68, -1.65, -1.62, -1.60, -1.42, -1.39.
HRMS: $C_{106}H_{129}N_6O_{33}P_5$ + H^+ required 2169.7361, found 2169.7365

(Protected) (GroP)$_3$(GlcGroP)(GroP)$_2$-Spacer or Hexamer 26

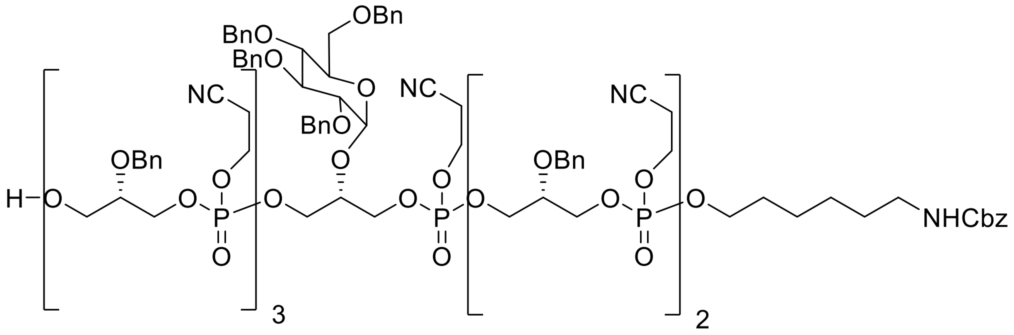

Alchol **S16** (35 μmol) was coupled with phospharamidite **8** (86 μmol, 2.5 eq) following the general procedure. Compound **26** was obtained after column chromatography (DCM:Acetone, 1:1) in 65% yield (23 μmol).
TLC analysis, R_f: 0.41 (DCM:Acetone, 7:3)
^{1}H-NMR (400 MHz, CD_3CN), δ: 7.43-7.12 (50H, H_{arom}, m), 5.75-5.64 (1H, NH, b), 5.20-5.13 (1H, H_1, m), 5.03 (2H, CH_{2_Cbz}, s), 4.88-4.41 (18H, 7 x CH_{2_Bn}, m), 4.30-3.94 (36H, 6 x CH_{2_OCE}, 11 x $CH_{2\_glycerol}$, $CH_{2\_Ospacer}$, m), 3.94-3.74 (6H, 4 x $CH_{\_glycerol}$, H_5, H_3, m), 3.74-3.42 (8H, 2 x $CH_{glycerol}$, 2 x H_6, H_4, H_2, $CH_{2\_glycerol}$), 3.17-2.99 (3H, OH, $CH_{2\_Nspacer}$, m), 2.77-2.53 (12H, 6 x CH_{2_OCE}, m), 1.65-1.49 (2H, CH_{2_spacer}, m), 1.45-1.14 (6H, 3 x CH_{2_spacer}, m).

^{13}C-NMR(101 MHz, CD_3CN), δ: 157.3, 140.1, 139.6 x 2, 139.5, 139.0 (C_q), 129.4 x 2, 129.3 x 3, 129.2, 129.1, 129.0 x 2, 128.9 x 2, 128.8 x 3,128.7 x 2, 128.6, 128.5, 128.4 (CH_{arom}), 118.6 (C_q), 97.0 (C_1), 82.3 (C_3), 80.7 (C_2), 79.1-79.0 ($CH_{gltcerol}$), 78.6 (C_4), 76.7 ($CH_{glycerol}$), 76.0, 75.6, 73.8, 73.1, 72.7, 72.4 (CH_{2_Bn}), 71.7 (C_5), 69.7 (C_6), 69.2 ($CH_{2\_Ospacer}$), 67.8-66.6 ($CH_{2\_glycerol}$, CH_{2_Cbz}), 63.6-63.3 (CH_{2_OCE}), 61.1 ($CH_{2\_glycerol}$), 41.4 ($CH_{2\_Nspacer}$), 30.7-30.4, 26.8, 25,7 (CH_{2_spacer}), 20.2-20.1 (CH_{2_OCE}).
^{31}P-NMR(162 MHz, CD_3CN), δ: -1.69, -1.67, -1.64, -1.63, -1.61, -1.58, -1.44, -1.40, 1.37.
HRMS: $C_{119}H_{145}N_7O_{38}P_6 + H^+$ required 2466.8128, found 2466.8125

(Protected) (GlcGroP)(GroP)-Spacer or Dimer S17

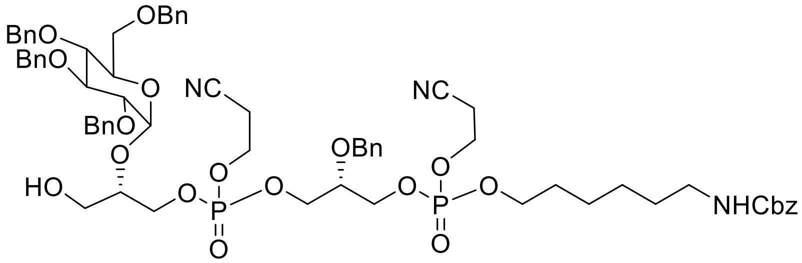

Alchol **S6** (160 μmol) was coupled with phospharamidite **7** (200 μmol, 1.3 eq) following the general procedure. Compound **S17** was obtained after column chromatography (DCM:Acetone, 7:3) in 64% yield (102 μmol).
TLC analysis, R_f: 0.38 (DCM:Acetone, 7:3)
^{1}H-NMR (400 MHz, CD_3CN), δ: 7.48-7.11 (30H, H_{arom}, m), 5.73-5.62 (1H, NH, b), 5.19-5.13 (1H, H_1, m), 5.03 (2H, CH_{2_Cbz}, s), 4.89-4.42 (10H, 5 x CH_{2_Bn}, m), 4.30-3.96 (12H, 2 x CH_{2_OCE}, 3 x $CH_{2\_glycerol}$, $CH_{2\_Ospacer}$, m), 3.96-3.75 (4H, 2 x $CH_{\_glycerol}$, H_5, H_3, m), 3.74-3.42 (6H, 2 x H_6, H_4, H_2, $CH_{2\_glycerol}$), 3.15-3.00 (3H, OH, $CH_{2\_Nspacer}$, m), 2.77-2.53 (4H, 2 x CH_{2_OCE}, m), 1.65-1.49 (2H, CH_{2_spacer}, m), 1.45-1.14 (6H, 3 x CH_{2_spacer}, m).
^{13}C-NMR(101 MHz, CD_3CN), δ: 157.3, 139.7, 139.6, 139.5, 139.4 (C_q), 129.4 x 2, 129.3 x 2, 129.2, 129.1 x 2, 129.0, 128.9 x 2, 128.8 x 2,128.7, 128.5, 128.4 (CH_{arom}), 118.6 (C_q), 97.4 (C_1), 82.5 (C_3), 81.0 (C_2), 79.2 ($CH_{gltcerol}$), 78.7 (C_4), 78.0-77.8 ($CH_{glycerol}$), 76.0, 75.6, 73.8, 73.5, 72.7, 72.4 (CH_{2_Bn}), 71.6 (C_5), 69.7 (C_6), 69.0 ($CH_{2\_Ospacer}$), 68.3-66.6 ($CH_{2\_glycerol}$, CH_{2_Cbz}), 63.4-63.1 (CH_{2_OCE}), 61.1 ($CH_{2\_glycerol}$), 41.4 ($CH_{2\_Nspacer}$), 30.7-30.4, 26.8, 25,7 (CH_{2_spacer}), 20.2-20.1 (CH_{2_OCE}).
^{31}P-NMR(162 MHz, CD_3CN), δ: -1.64, -1.61, -1.60, -1.48, -1.46, -1.44, -1.43, -1.39, -1.32, -1.28.
HRMS: $C_{67}H_{81}N_3O_{18}P_2 + H^+$ required 1278.5063, found 1278.5064

(Protected) (GroP)(GlcGroP)(GroP) -Spacer or Trimer S18

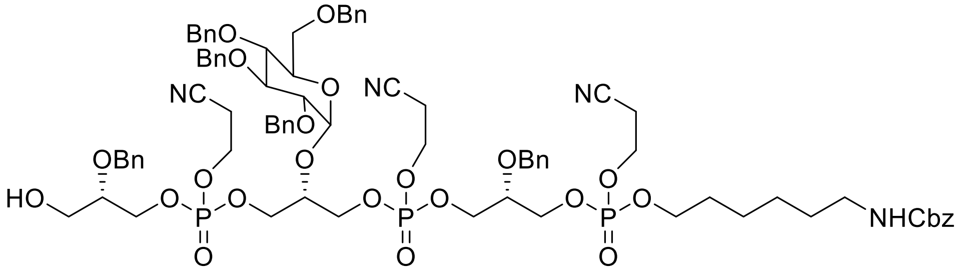

Alchol **S17** (86 μmol) was coupled with phospharamidite **8** (215 μmol, 2.5 eq) following the general procedure. Compound **S18** was obtained after column chromatography (DCM:Acetone, 6:4) in 68% yield (58 μmol).
TLC analysis, R_f: 0.35 (DCM:Acetone, 6:4)
^{1}H-NMR (400 MHz, CD_3CN), δ: 7.48-7.11 (35H, H_{arom}, m), 5.73-5.62 (1H, NH, b), 5.19-5.13 (1H, H_1, m), 5.03 (2H, CH_{2_Cbz}, s), 4.89-4.42 (12H, 6 x CH_{2_Bn}, m), 4.30-3.96 (18H, 3 x CH_{2_OCE}, 5 x $CH_{2\_glycerol}$, $CH_{2\_Ospacer}$, m), 3.96-3.75 (4H, 2 x $CH_{\_glycerol}$, H_5, H_3, m), 3.74-3.42 (7H, $CH_{glycerol}$, 2 x H_6, H_4, H_2, $CH_{2\_glycerol}$), 3.15-3.00 (3H, OH, $CH_{2\_Nspacer}$, m), 2.77-2.53 (6H, 3 x CH_{2_OCE}, m), 1.65-1.49 (2H, CH_{2_spacer}, m), 1.45-1.14 (6H, 3 x CH_{2_spacer}, m).

^{13}C-NMR(101 MHz, CD_3CN), δ: 157.3, 140.0, 139.6 x 2, 139.5, 139.1 (C_q), 129.4 x 2, 129.3 x 3, 129.2, 129.1 x 2, 129.0 x 3, 128.9 x 3, 128.8 x 3,128.7 x 3, 128.6 x 2, 128.4 (CH_{arom}), 118.6 (C_q), 97.1 (C_1), 82.3 (C_3), 80.7 (C_2), 79.1-79.0 ($CH_{gltcerol}$), 78.6 (C_4), 76.8 ($CH_{glycerol}$), 76.0, 75.6 x 2, 73.9, 73.1, 72.4 (CH_{2_Bn}), 71.7 (C_5), 69.7 (C_6), 69.1 ($CH_{2\_Ospacer}$), 67.9-66.4 ($CH_{2\_glycerol}$, CH_{2_Cbz}), 63.6-63.2 (CH_{2_OCE}), 61.1 ($CH_{2\_glycerol}$), 41.4 ($CH_{2\_Nspacer}$), 30.7-30.4, 26.8, 25,7 (CH_{2_spacer}), 20.2-20.1 (CH_{2_OCE}).
^{31}P-NMR(162 MHz, CD_3CN), δ: -0.43, -0.42, -0.40, -0.39, -0.37, -0.36, -0.35, -0.12, -0.09, -0.07
HRMS: $C_{80}H_{97}N_4O_{23}P_3 + H^+$ required 1575.5829, found 1575.5832

(Protected) (GroP)$_2$(GlcGroP)(GroP) -Spacer or Tetramer S19

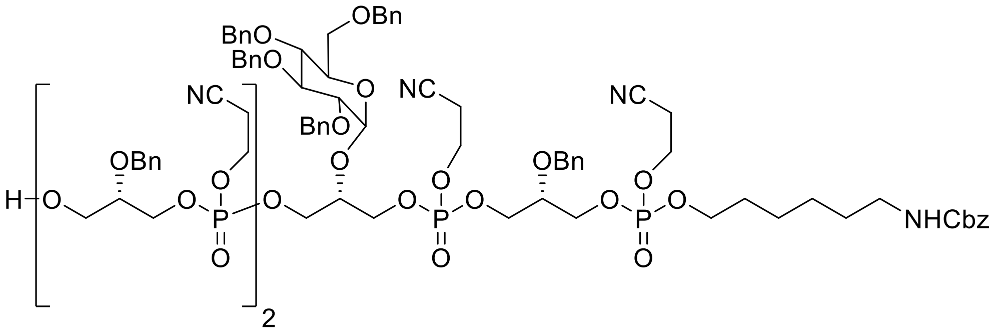

Alchol **S18** (41 μmol) was coupled with phospharamidite **8** (102 μmol, 2.5 eq) following the general procedure. Compound **S19** was obtained after column chromatography (DCM:Acetone, 1:1) in 65% yield (27 μmol).
TLC analysis, R_f: 0.31 (DCM:Acetone, 1:1)
^{1}H-NMR (400 MHz, CD_3CN), δ: 7.42-7.11 (40H, H_{arom}, m), 5.72-5.61 (1H, NH, b), 5.20-5.13 (1H, H_1, m), 5.02 (2H, CH_{2_Cbz}, s), 4.89-4.42 (14H, 7 x CH_{2_Bn}, m), 4.30-3.96 (24H, 4 x CH_{2_OCE}, 7 x $CH_{2\_glycerol}$, $CH_{2\_Ospacer}$, m), 3.96-3.75 (4H, 2 x $CH_{\_glycerol}$, H_5, H_3, m), 3.74-3.42 (7H, $CH_{glycerol}$, 2 x H_6, H_4, H_2, $CH_{2\_glycerol}$), 3.15-3.00 (3H, OH, $CH_{2\_Nspacer}$, m), 2.77-2.53 (8H, 4 x CH_{2_OCE}, m), 1.65-1.49 (2H, CH_{2_spacer}, m), 1.45-1.14 (6H, 3 x CH_{2_spacer}, m).
^{13}C-NMR(101 MHz, CD_3CN), δ: 157.3, 139.7, 139.6, 139.5, 139.4 (C_q), 129.4 x 2, 129.3 x 2, 129.2, 129.1 x 2, 129.0, 128.9 x 2, 128.8 x 2,128.7, 128.5, 128.4 (CH_{arom}), 118.6 (C_q), 97.4 (C_1), 82.5 (C_3), 81.0 (C_2), 79.2 ($CH_{gltcerol}$), 78.7 (C_4), 78.0-77.8 ($CH_{glycerol}$), 76.0, 75.6, 73.8, 73.5, 72.7, 72.4 (CH_{2_Bn}), 71.6 (C_5), 69.7 (C_6), 69.0 ($CH_{2\_Ospacer}$), 68.3-66.6 ($CH_{2\_glycerol}$, CH_{2_Cbz}), 63.4-63.1 (CH_{2_OCE}), 61.1 ($CH_{2\_glycerol}$), 41.4 ($CH_{2\_Nspacer}$), 30.7-30.4, 26.8, 25,7 (CH_{2_spacer}), 20.2-20.1 (CH_{2_OCE}).
^{31}P-NMR(162 MHz, CD_3CN), δ: -0.42, -0.40, -0.36, -0.34, -0.32, -0.14, -0.12.
HRMS: $C_{93}H_{113}N_5O_{28}P_4 + H^+$ required 1872.6595, found 1872.6598

(Protected) (GroP)$_3$(GlcGroP)(GroP) -Spacer or Pentamer S20

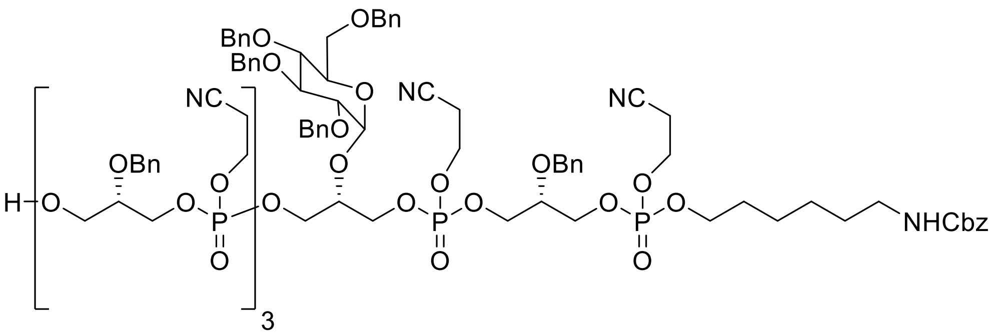

Alchol **S19** (20 μmol) was coupled with phospharamidite **8** (50 μmol, 2.5 eq) following the general procedure. Compound **S20** was obtained after column chromatography (DCM:Acetone, 1:1) in 77% yield (15 μmol).
TLC analysis, R_f: 0.38 (DCM:Acetone, 4:6)

^{1}H-NMR (400 MHz, CD_3CN), δ: 7.48-7.11 (45H, H_{arom}, m), 5.73-5.62 (1H, NH, b), 5.19-5.13 (1H, H_1, m), 5.03 (2H, CH_{2_Cbz}, s), 4.89-4.42 (16H, 8 x CH_{2_Bn}, m), 4.30-3.96 (30H, 5 x CH_{2_OCE}, 9 x $CH_{2\_glycerol}$, $CH_{2\_Ospacer}$, m), 3.96-3.75 (4H, 3 x $CH_{\_glycerol}$, H_5, H_3, m), 3.74-3.42 (6H, 2 x H_6, H_4, H_2, $CH_{2\_glycerol}$), 3.15-3.00 (3H, OH, $CH_{2\_Nspacer}$, m), 2.77-2.53 (4H, 2 x CH_{2_OCE}, m), 1.65-1.49 (2H, CH_{2_spacer}, m), 1.45-1.14 (6H, 3 x CH_{2_spacer}, m).
^{13}C-NMR(101 MHz, CD_3CN), δ: 157.3, 139.7, 139.6, 139.5, 139.4 (C_q), 129.4 x 2, 129.3 x 2, 129.2, 129.1 x 2, 129.0, 128.9 x 2, 128.8 x 2,128.7, 128.5, 128.4 (CH_{arom}), 118.6 (C_q), 97.4 (C_1), 82.5 (C_3), 81.0 (C_2), 79.2 ($CH_{gltcerol}$), 78.7 (C_4), 78.0-77.8 ($CH_{glycerol}$), 76.0, 75.6, 73.8, 73.5, 72.7, 72.4 (CH_{2_Bn}), 71.6 (C_5), 69.7 (C_6), 69.0 ($CH_{2\_Ospacer}$), 68.3-66.6 ($CH_{2\_glycerol}$, CH_{2_Cbz}), 63.4-63.1 (CH_{2_OCE}), 61.1 ($CH_{2\_glycerol}$), 41.4 ($CH_{2\_Nspacer}$), 30.7-30.4, 26.8, 25,7 (CH_{2_spacer}), 20.2-20.1 (CH_{2_OCE}).
^{31}P-NMR(162 MHz, CD_3CN), δ: -0.41, -0.36, -0.34, -0.32, -0.30, -0.29, -0.14, -0.11.
HRMS: $C_{106}H_{129}N_6O_{33}P_5 + H^+$ required 2169.7361, found 2169.7363

(Protected) $(GroP)_4$(GlcGroP)(GroP) -Spacer or Hexamer 27

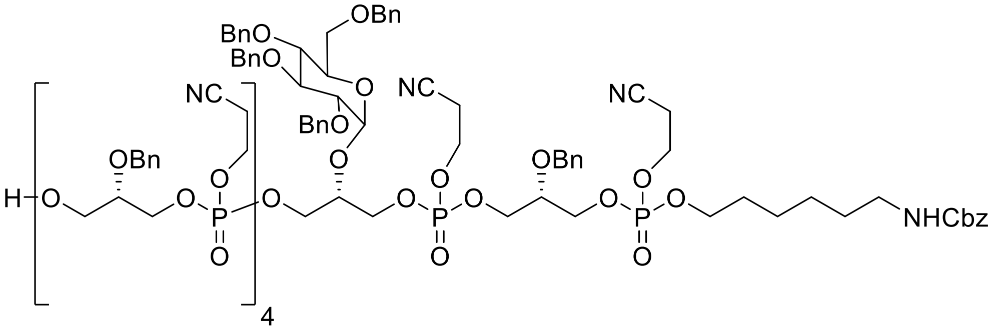

Alchol **S20** (13 μmol) was coupled with phospharamidite **8** (40 μmol, 2.5 eq) following the general procedure. Compound **27** was obtained after column chromatography (DCM:Acetone, 1:1) in 72% yield (9.4 μmol).
TLC analysis, R_f: 0.31 (DCM:Acetone, 4:6)
^{1}H-NMR (400 MHz, CD_3CN), δ: 7.48-7.11 (50H, H_{arom}, m), 5.73-5.62 (1H, NH, b), 5.19-5.13 (1H, H_1, m), 5.03 (2H, CH_{2_Cbz}, s), 4.89-4.42 (18H, 9 x CH_{2_Bn}, m), 4.30-3.96 (36H, 6 x CH_{2_OCE}, 11 x $CH_{2\_glycerol}$, $CH_{2\_Ospacer}$, m), 3.96-3.75 (6H, 4 x $CH_{\_glycerol}$, H_5, H_3, m), 3.74-3.42 (7H, $CH_{glycerol}$, 2 x H_6, H_4, H_2, $CH_{2\_glycerol}$), 3.15-3.00 (3H, OH, $CH_{2\_Nspacer}$, m), 2.77-2.53 (4H, 2 x CH_{2_OCE}, m), 1.65-1.49 (2H, CH_{2_spacer}, m), 1.45-1.14 (6H, 3 x CH_{2_spacer}, m).
^{13}C-NMR(101 MHz, CD_3CN), δ: 157.3, 139.7, 139.6, 139.5, 139.4 (C_q), 129.4 x 2, 129.3 x 2, 129.2, 129.1 x 2, 129.0, 128.9 x 2, 128.8 x 2,128.7, 128.5, 128.4 (CH_{arom}), 118.6 (C_q), 97.4 (C_1), 82.5 (C_3), 81.0 (C_2), 79.2 ($CH_{gltcerol}$), 78.7 (C_4), 78.0-77.8 ($CH_{glycerol}$), 76.0, 75.6, 73.8, 73.5, 72.7, 72.4 (CH_{2_Bn}), 71.6 (C_5), 69.7 (C_6), 69.0 ($CH_{2\_Ospacer}$), 68.3-66.6 ($CH_{2\_glycerol}$, CH_{2_Cbz}), 63.4-63.1 (CH_{2_OCE}), 61.1 ($CH_{2\_glycerol}$), 41.4 ($CH_{2\_Nspacer}$), 30.7-30.4, 26.8, 25,7 (CH_{2_spacer}), 20.2-20.1 (CH_{2_OCE}).
^{31}P-NMR(162 MHz, CD_3CN), δ: -0.41, -0.36, -0.34, -0.32, -0.30, -0.29, -0.14, -0.11.
HRMS: $C_{119}H_{145}N_7O_{38}P_6 + H^+$ required 2466.8128, found 2466.8130

(Protected) GlcGroP-Spacer or Monomer S21

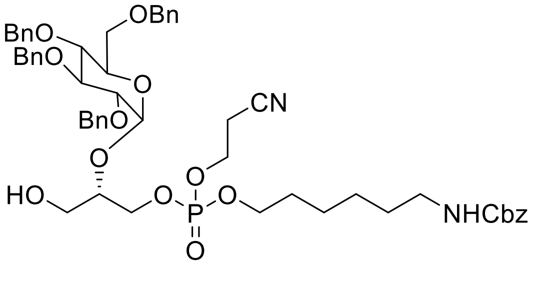

Alchol spacer **9** (0.26 mmol) was coupled with phospharamidite **7** (0.35 mmol, 1.3 eq) following the general procedure. Compound **S21** was obtained after column chromatography (DCM:Acetone, 7.5:2.5) in 81% yield (0.21 mmol).
TLC analysis, R_f: 0.45 (DCM:Acetone, 7:3

^{1}H-NMR (400 MHz, CD_3CN), δ: 7.47-7.12 (25H, H_{arom}, m), 5.68-5.54 (1H, NH, b), 5.16 (1H, H_1, J=3.6 Hz, d), 5.03 (2H, CH_{2_Cbz}, s), 4.88 (1H, CH$H_{_Bn}$, J=10.6 Hz, d), 4.82-4.61 (4H, 2 x CH_{2_Bn}, m), 4.58-4.43 (3H, CH$H_{_Bn}$), 4.22-3.06 (4H, CH_{2_OCE}, $CH_{2\_glycerol}$), 4.05-3.80 (5H, $CH_{2\_Ospacer}$, H_5, $CH_{glycerol}$, H_3, m), 3.75-3.46 (6H, 2 x H_6, H_4, H_2, $CH_{2\_glycerol}$), 3.15-2.98 (3H, OH, $CH_{2\_Nspacer}$, m), 2.68-2.57 (2H,CH_{2_OCE}, m), 1.65-1.49 (2H, CH_{2_spacer}, m), 1.45-1.14 (6H, 3 x CH_{2_spacer}, m).
^{13}C-NMR(101 MHz, CD_3CN), δ: 157.3, 140.1, 139.6, 139.5, 139.4 (C_q), 129.4, 129.3 x 2, 129.2 x 2, 129.1, 128.9 x 2, 128.8 x 2,128.7 x 2, 128.5, 128.4 (CH_{arom}), 118.6 (C_q), 97.3 (C_1), 82.5 (C_3), 81.0 (C_2), 78.7 (C_4), 78.0-77.8 ($CH_{glycerol}$), 76.0, 75.6, 73.8, 73.5 (CH_{2_Bn}), 71.5 (C_5), 69.7 (C_6), 69.0 ($CH_{2\_Ospacer}$), 68.1-68.0 ($CH_{2\_glycerol}$), 66.6 CH_{2_Cbz}), 63.2 (CH_{2_OCE}), 61.1 ($CH_{2\_glycerol}$), 41.4 ($CH_{2\_Nspacer}$), 30.7-30.4, 26.8, 25.7 (CH_{2_spacer}), 20.2-20.1 (CH_{2_OCE}).
^{31}P-NMR(162 MHz, CD_3CN), δ: -1.44, -1.37
HRMS: $C_{54}H_{65}N_2O_{13}P + H^+$ required 981.4297, found 981.4296

(Protected) (GroP)(GlcGroP)-Spacer or Dimer S22

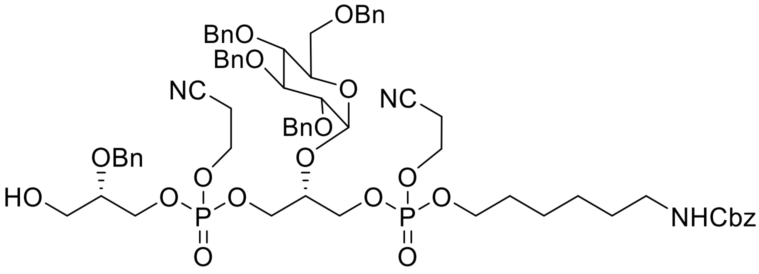

Alchol **S21** (0.17 mmol) was coupled with phospharamidite **8** (0.26 mmol, 1.5 eq) following the general procedure. Compound **S22** was obtained after column chromatography (DCM:Acetone, 6:4) in 82% yield (0.14 mmol).
TLC analysis, R_f: 0.32 (DCM:Acetone, 1:1).
^{1}H-NMR (400 MHz, CD_3CN), δ: 7.48-7.10 (30H, H_{arom}, m), 5.71-5.55 (1H, NH, b), 5.16 (1H, H_1, J=3.6 Hz, d), 5.02 (2H, CH_{2_Cbz}, s), 4.90-4.83 (1H, CH$H_{_Bn}$, m), 4.80-4.69 (3H, 3 x CH$H_{_Bn}$, m), 4.66-4.56 (3H, 3 x CH$H_{_Bn}$, m), 4.58-4.43 (3H, CH$H_{_Bn}$), 4.30-4.04 (11H,2 x CH_{2_OCE}, 3 x $CH_{2\_glycerol}$, $CH_{glycerol}$), 4.05-3.96 (2H, $CH_{2\_Ospacer}$, m), 3.95-3.86 (H_5), 3.86-3.76 (1H, H_3, m), 3.75-3.46 (7H, $CH_{gòycerol}$, 2 x H_6, H_4, H_2, $CH_{2\_glycerol}$), 3.09-2.97 (3H, OH, $CH_{2\_Nspacer}$, m), 2.78-2.52 (4H,2 x CH_{2_OCE}, m), 1.65-1.49 (2H, CH_{2_spacer}, m), 1.45-1.14 (6H, 3 x CH_{2_spacer}, m).
^{13}C-NMR(101 MHz, CD_3CN), δ: 157.3, 140.1, 139.7, 139.6, 139.5 (C_q), 129.4 x3, 129.3 x 2, 129.2, 129.1 x 2, 129.0, 128.9 x 2, 128.8 x 3,128.7 x 2, 128.6 x 2, 128.4 (CH_{arom}), 118.6 (C_q), 97.1-97.0 (C_1), 82.3 (C_3), 80.8 (C_2), 79.2-79.1 ($CH_{glycerol}$), 78.6 (C_4), 76.0, 75.6 (CH_{2_Bn}), 75.1-74.2 ($CH_{glycerol}$), 73.9, 73.1, 72.5 (CH_{2_Bn}), 71.7 (C_5), 69.7 (C_6), 69.2 ($CH_{2\_Ospacer}$), 67.8-66.4 ($CH_{2\_glycerol}$, CH_{2_Cbz}), 63.5-63.3 (CH_{2_OCE}), 61.1 ($CH_{2\_glycerol}$), 41.4 ($CH_{2\_Nspacer}$), 30.7-30.4, 26.8, 25.7 (CH_{2_spacer}), 20.2-20.1 (CH_{2_OCE}).
^{31}P-NMR(162 MHz, CD_3CN), δ: -1.72, -1.70, -1.67, -1.66 -1.47, -1.41
HRMS: $C_{67}H_{81}N_3O_{18}P_2 + H^+$ required 1278.5063, found 1278.5067

(Protected) (GroP)$_2$(GlcGroP)-Spacer or Trimer S23

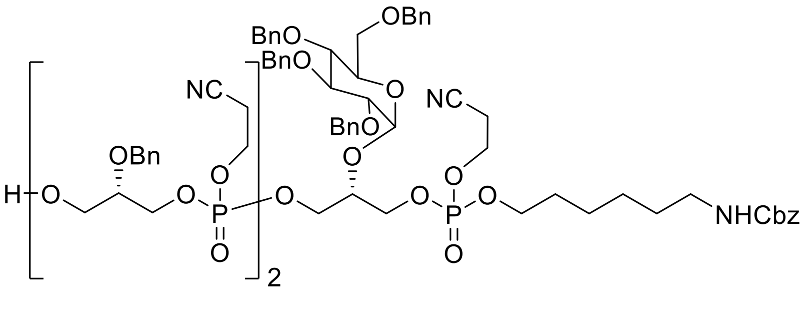

Alchol **S22** (0.12 mmol) was coupled with phospharamidite **8** (0.24 mmol, 2.0 eq) following the general procedure. Compound **S23** was obtained after column chromatography (DCM:Acetone, 1:1) in 77% yield (0.92 mmol).
TLC analysis, R_f: 0.38 (DCM:Acetone, 4:6)

^{1}H-NMR (400 MHz, CD_3CN), δ: 7.48-7.10 (35H, H_{arom}, m), 5.71-5.56 (1H, NH, b), 5.18-5.15 (1H, H_1, m), 5.02 (2H, CH_{2_Cbz}, s), 4.90-4.81 (1H, $CHH_{\_Bn}$, m), 4.80-4.69 (3H, 3 x $CHH_{\_Bn}$, m), 4.65-4.45 (8H, 8 x $CHH_{\_Bn}$, m), 4.28-3.94 (17H,3 x CH_{2_OCE}, 5 x $CH_{2\_glycerol}$, $CH_{glycerol}$), 3.94-3.75 (5H, $CH_{2\_Ospacer}$, H_5, H_3, $CH_{glycerol}$, m), 3.75-3.46 (7H, $CH_{gòycerol}$, 2 x H_6, H_4, H_2, $CH_{2\_glycerol}$), 3.13-2.97 (3H, OH, $CH_{2\_Nspacer}$, m), 2.73-2.58 (6H,3 x CH_{2_OCE}, m), 1.65-1.49 (2H, CH_{2_spacer}, m), 1.45-1.14 (6H, 3 x CH_{2_spacer}, m).
^{13}C-NMR(101 MHz, CD_3CN), δ: 157.3, 140.1, 139.7, 139.6, 139.5 (C_q), 129.4 x3, 129.3 x 2, 129.2, 129.1 x 2, 129.0, 128.9 x 2, 128.8 x 3,128.7 x 2, 128.6 x 2, 128.4 (CH_{arom}), 118.6 (C_q), 97.1-97.0 (C_1), 82.3 (C_3), 80.8 (C_2), 79.2-79.1 ($CH_{glycerol}$), 78.6 (C_4), 76.0, 75.6 (CH_{2_Bn}), 75.1-74.2 ($CH_{glycerol}$), 73.9, 73.1, 72.5 (CH_{2_Bn}), 71.7 (C_5), 69.7 (C_6), 69.2 ($CH_{2\_Ospacer}$), 67.8-66.4 ($CH_{2\_glycerol}$, CH_{2_Cbz}), 63.5-63.3 (CH_{2_OCE}), 61.1 ($CH_{2\_glycerol}$), 41.4 ($CH_{2\_Nspacer}$), 30.7-30.4, 26.8, 25.7 (CH_{2_spacer}), 20.2-20.1 (CH_{2_OCE}).
^{31}P-NMR(162 MHz, CD_3CN), δ: -1.72, -1.71, -1.69, -1.68, -1.66, -1.65, -1.64, -1.61, -1.40, -1.38, -1.35
HRMS: $C_{80}H_{97}N_4O_{23}P_3 + H^+$ required 1575.5829, found 1575.5827

(Protected) (GroP)$_3$(GlcGroP)-Spacer or Tetramer S24

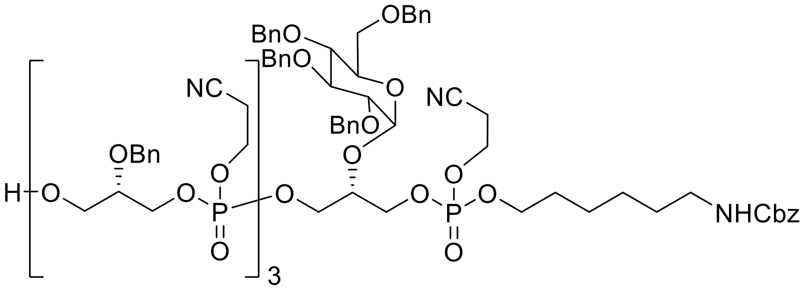

Alchol **S23** (80 μmol) was coupled with phospharamidite **8** (160 μmol, 2.0 eq) following the general procedure. Compound **S24** was obtained after column chromatography (DCM:Acetone, 1:1) in 81% yield (65 μmol).
TLC analysis, R_f: 0.33 (DCM:Acetone, 4:6)
^{1}H-NMR (400 MHz, CD_3CN), δ: 7.48-7.10 (35H, H_{arom}, m), 5.74-5.56 (1H, NH, b), 5.18-5.15 (1H, H_1, m), 5.02 (2H, CH_{2_Cbz}, s), 4.90-4.81 (1H, $CHH_{\_Bn}$, m), 4.80-4.69 (3H, 3 x $CHH_{\_Bn}$, m), 4.65-4.45 (10H, 10 x $CHH_{\_Bn}$, m), 4.28-3.94 (23H,4 x CH_{2_OCE}, 7 x $CH_{2\_glycerol}$, $CH_{glycerol}$), 3.94-3.75 (6H, $CH_{2\_Ospacer}$, H_5, H_3,2 x $CH_{glycerol}$, m), 3.75-3.46 (7H, $CH_{gòycerol}$, 2 x H_6, H_4, H_2, $CH_{2\_glycerol}$), 3.16-2.97 (3H, OH, $CH_{2\_Nspacer}$, m), 2.73-2.58 (8H,4 x CH_{2_OCE}, m), 1.65-1.49 (2H, CH_{2_spacer}, m), 1.45-1.14 (6H, 3 x CH_{2_spacer}, m).
^{13}C-NMR(101 MHz, CD_3CN), δ: 157.3, 140.1, 139.6 x 2, 139.5. 139.1 (C_q), 129.4 x3, 129.3 x 2, 129.2 x 2, 129.1 x 2, 129.0 x 3, 128.9 x 2, 128.8 x 3,128.7 x 2, 128.6 x 2, 128.4 (CH_{arom}), 118.6 (C_q), 97.0-96.9 (C_1), 82.3 (C_3), 80.8 (C_2), 79.2-79.1 ($CH_{glycerol}$), 78.6 (C_4), 76.0, 75.6 (CH_{2_Bn}), 75.1-74.2 ($CH_{glycerol}$), 73.9, 73.1, 72.5 (CH_{2_Bn}), 71.7 (C_5), 69.7 (C_6), 69.2 ($CH_{2\_Ospacer}$), 67.8-66.4 ($CH_{2\_glycerol}$, CH_{2_Cbz}), 63.5-63.3 (CH_{2_OCE}), 61.1 ($CH_{2\_glycerol}$), 41.4 ($CH_{2\_Nspacer}$), 30.7-30.4, 26.8, 25.7 (CH_{2_spacer}), 20.2-20.1 (CH_{2_OCE}).
^{31}P-NMR(162 MHz, CD_3CN), δ: -1.67, -1.66, -1.64, -1.63, -1.61, -1.59, -1.57, -1.39, -1.37.
HRMS: $C_{93}H_{113}N_5O_{28}P_4 + H^+$ required 1872.6595, found 1872.6601

(Protected) (GroP)$_4$(GlcGroP)-Spacer or Pentamer S25

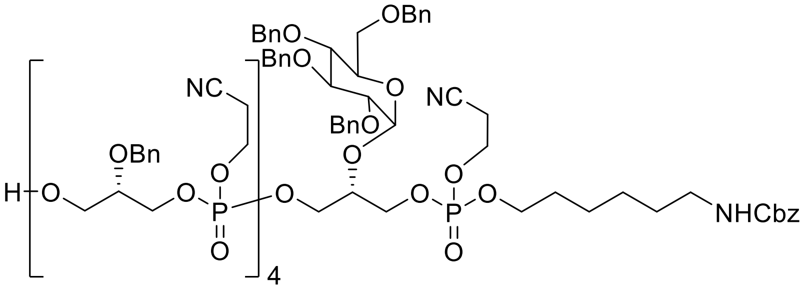

Alchol **S24** (48 μmol) was coupled with phospharamidite **8** (120 μmol, 2.5 eq) following the general procedure. Compound **S25** was obtained after column chromatography (DCM:Acetone, 1:1) in 76% yield (36 μmol).
TLC analysis, R_f: 0.31 (DCM:Acetone, 4:6)

^{1}H-NMR (400 MHz, CD_3CN), δ: 7.48-7.10 (35H, H_{arom}, m), 5.74-5.56 (1H, NH, b), 5.18-5.15 (1H, H_1, m), 5.02 (2H, CH_{2_Cbz}, s), 4.90-4.81 (1H, $CHH_{\_Bn}$, m), 4.80-4.69 (3H, 3 x $CHH_{\_Bn}$, m), 4.65-4.45 (12H, 12 x $CHH_{\_Bn}$, m), 4.28-3.94 (29H,5 x CH_{2_OCE}, 9 x $CH_{2\_glycerol}$, $CH_{glycerol}$), 3.94-3.75 (7H, $CH_{2\_Ospacer}$, H_5, H_3,3 x $CH_{glycerol}$, m), 3.75-3.46 (7H, $CH_{gòycerol}$, 2 x H_6, H_4, H_2, $CH_{2\_glycerol}$), 3.16-2.97 (3H, OH, $CH_{2\_Nspacer}$, m), 2.73-2.58 (8H,4 x CH_{2_OCE}, m), 1.65-1.49 (2H, CH_{2_spacer}, m), 1.45-1.14 (6H, 3 x CH_{2_spacer}, m).
^{13}C-NMR(101 MHz, CD_3CN), δ: 157.3, 140.1, 139.6 x 2, 139.5. 139.1 (C_q), 129.4 x3, 129.3 x 2, 129.2 x 2, 129.1 x 2, 129.0 x 3, 128.9 x 2, 128.8 x 3,128.7 x 2, 128.6 x 2, 128.4 (CH_{arom}), 118.6 (C_q), 97.0-96.9 (C_1), 82.3 (C_3), 80.8 (C_2), 79.2-79.1 ($CH_{glycerol}$), 78.6 (C_4), 76.0, 75.6 (CH_{2_Bn}), 75.1-74.2 ($CH_{glycerol}$), 73.9, 73.1, 72.5 (CH_{2_Bn}), 71.7 (C_5), 69.7 (C_6), 69.2 ($CH_{2\_Ospacer}$), 67.8-66.4 ($CH_{2\_glycerol}$, CH_{2_Cbz}), 63.5-63.3 (CH_{2_OCE}), 61.1 ($CH_{2\_glycerol}$), 41.4 ($CH_{2\_Nspacer}$), 30.7-30.4, 26.8, 25.7 (CH_{2_spacer}), 20.2-20.1 (CH_{2_OCE}).
^{31}P-NMR(162 MHz, CD_3CN), δ: -0.44, -0.43, -0.40, -0.38, -0.34, -0.32, -0.18, -0.15.
HRMS: $C_{106}H_{129}N_6O_{33}P_5 + H^+$ required 2169.7361, found 2169.7358

(Protected) $(GroP)_5$(GlcGroP)-Spacer or Hexamer 28

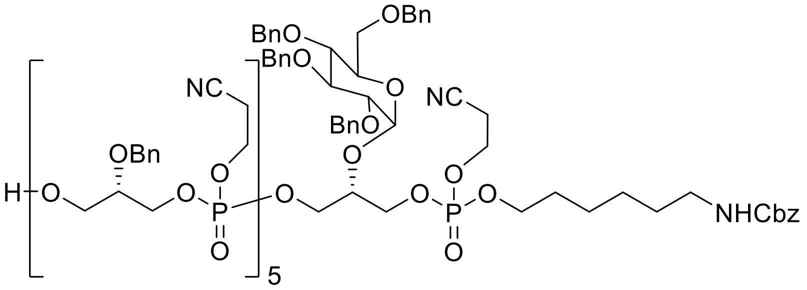

Alchol **S25** (23 μmol) was coupled with phospharamidite **8** (58 μmol, 2.5 eq) following the general procedure. Compound **28** was obtained after column chromatography (DCM:Acetone, 1:1) in 65% yield (15 μmol).
TLC analysis, R_f: 0.28 (DCM:Acetone, 4:6)
^{1}H-NMR (400 MHz, CD_3CN), δ: 7.48-7.10 (35H, H_{arom}, m), 5.74-5.56 (1H, NH, b), 5.18-5.15 (1H, H_1, m), 5.02 (2H, CH_{2_Cbz}, s), 4.90-4.81 (1H, $CHH_{\_Bn}$, m), 4.80-4.69 (3H, 3 x $CHH_{\_Bn}$, m), 4.65-4.45 (14H, 14 x $CHH_{\_Bn}$, m), 4.28-3.94 (35H, 6 x CH_{2_OCE}, 11 x $CH_{2\_glycerol}$, $CH_{glycerol}$), 3.94-3.75 (8H, $CH_{2\_Ospacer}$, H_5, H_3,4 x $CH_{glycerol}$, m), 3.75-3.46 (7H, $CH_{gòycerol}$, 2 x H_6, H_4, H_2, $CH_{2\_glycerol}$), 3.16-2.97 (3H, OH, $CH_{2\_Nspacer}$, m), 2.73-2.58 (8H,4 x CH_{2_OCE}, m), 1.65-1.49 (2H, CH_{2_spacer}, m), 1.45-1.14 (6H, 3 x CH_{2_spacer}, m).
^{13}C-NMR(101 MHz, CD_3CN), δ: 157.3, 140.1, 139.6 x 2, 139.5. 139.1 (C_q), 129.4 x3, 129.3 x 2, 129.2 x 2, 129.1 x 2, 129.0 x 3, 128.9 x 2, 128.8 x 3,128.7 x 2, 128.6 x 2, 128.4 (CH_{arom}), 118.6 (C_q), 97.0-96.9 (C_1), 82.3 (C_3), 80.8 (C_2), 79.2-79.1 ($CH_{glycerol}$), 78.6 (C_4), 76.0, 75.6 (CH_{2_Bn}), 75.1-74.2 ($CH_{glycerol}$), 73.9, 73.1, 72.5 (CH_{2_Bn}), 71.7 (C_5), 69.7 (C_6), 69.2 ($CH_{2\_Ospacer}$), 67.8-66.4 ($CH_{2\_glycerol}$, CH_{2_Cbz}), 63.5-63.3 (CH_{2_OCE}), 61.1 ($CH_{2\_glycerol}$), 41.4 ($CH_{2\_Nspacer}$), 30.7-30.4, 26.8, 25.7 (CH_{2_spacer}), 20.2-20.1 (CH_{2_OCE}).
^{31}P-NMR(162 MHz, CD_3CN), δ: -0.41, -0.36, -0.34, -0.32, -0.30, -0.29, -0.14, -0.11.
HRMS: $C_{119}H_{145}N_7O_{38}P_6 + H^+$ required 2466.8128, found 2466.8133

Final deprotections

The oligomer is dissolved in dioxane (2mM) and upon the addition of ammonia solution in H_2O (33%) the reaction mixture turns turbid. Once the solution becomes transparent (1-3 hours) the reaction mixture is concentrated *in vacuo*. After checking by ^{1}H-NMR the disappearing of the cianoehtyl group, the residue is flushed over Dowex Na^+ cation-exchange resin (type 50WX4-200, stored in 0.5M NaOH in MilliQ, flushed with MeOH and MilliQ before use) column. After evaporation, the residue is dissolved in MilliQ (2mM) and 2 drops of AcOH are added. $Ar_{(g)}$ is bubbled in the reaction mixture for 20 minutes while sonicating, Pd-black (≈10 mg) is added and after an additional 10 minutes of $Ar_{(g)}$ bubbling, the solution is left stirring under $H_{2(g)}$ atmosphere for 1 week. After filtration over Celite®, the reaction mixture is concentrated *in vacuo*. The final compound is purified by sixe-exclusion chromatography (HW40, dimensions: 16/60 mm, eluent: 0.15M NH_4OAc). After several co-evaporation with MilliQ, the product is eluted through a small column containing Dowex Na^+ cation-exchange resin (type 50WX4-200, stored in 0.5M NaOH in MilliQ, flushed with MeOH and MilliQ before use).

(GlcGroP)(GroP)$_5$-Spacer or Hexamer (1)

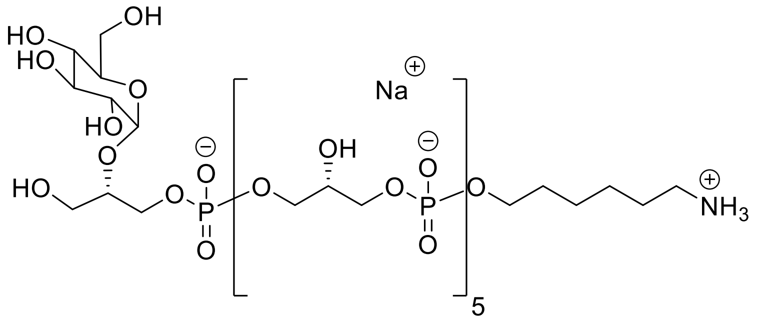

Compound **23** (6 μmol) was deprotected following the general procedure. The final product **1** was obtained in 78% yield (4.7 μmol).

^{1}H-NMR (850 MHz, CD_3CN), δ: 5.07 (1H, H_1, J=3.8 Hz, d), 4.05-3.95 (7H, 5 x $CH_{glycerol}$, $CH_{2\_glycerol}$, m), 3.95-3.78 (24H, 10 $CH_{2\_glycererol,}$ $CH\mathit{H}_{glycerol}$, $CH_{2\_Ospacer}$, H_5, m), 3.77-3.64 (4H, 2 x H_6, H_3, $CH\mathit{H}_{glycerol}$, m), 3.49 (1H, H_2, J=3.8 Hz, J=9.9 Hz, dd), 3.38-3.30 (1H, H_4, m), 3.00-2.91 (2H, $CH_{2\_Nspacer}$, m), 1.69-1.56 (4H, CH_{2_spacer}, m), 1.45-1.34 (4H, CH_{2_spacer}, m).

^{13}C-NMR(101 MHz, CD_3CN), δ: 100.4 (C_1), 79.7 ($CH_{glycerol}$), 75.6 (C_3), 74.5 (C_5), 74.2 (C_2), 72.3 (C_4), 72.2-72.1 ($CH_{glycerol}$), 68.9-68.6 ($CH_{2\_glycerol}$), 67.8-67.7 ($CH_{2\_glycerol}$), 63.1 (C_6), 62.8 ($CH_{2\_glycerol}$), 42.1 ($CH_{2\_Nspacer}$), 32.0, 29.2, 27.7, 27.1 ($CH_{2spacer}$).

^{31}P-NMR(162 MHz, CD_3CN), δ: 1.78, 1.89, 1.93, 2.04.

HRMS: $C_{30}H_{67}NO_{36}P_6$ + H^+ required 1204.1941, found 1204.1951

(GroP)(GlcGroP)(GroP)$_4$-Spacer or Hexamer (2)

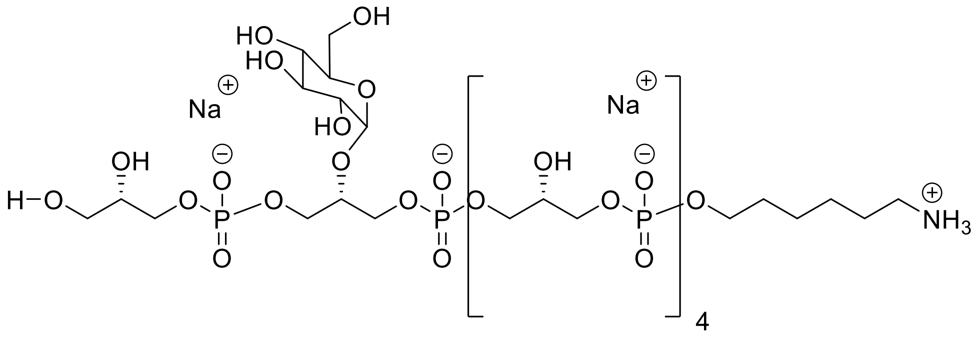

Compound **24** (11 μmol) was deprotected following the general procedure. The final product **2** was obtained in 73% yield (8 μmol).

^{1}H-NMR (850 MHz, CD_3CN), δ: 5.14 (1H, H_1, J=3.8 Hz, d), 4.11-4.04 (1H, $CH_{glycerol}$, m), 4.05-3.95 (8H, 4 x $CH_{glycerol}$, 2 x $CH_{2\_glycerol}$, m), 3.95-3.78 (23H, 9 x $CH_{2\_glycererol,}$ H_6, $CH_{2\_Ospacer}$, H_5, $CH_{glycerol}$, m), 3.76-3.68 (2H, H_6, H_3, m), 3.64 (1H, $CH\mathit{H}_{glycerol}$, J=4.3 Hz, J=11.8 Hz, dd), 3.56 (1H, $CH\mathit{H}_{glycerol}$, J=6.1 Hz, J=11.8 Hz, dd) 3.50 (1H, H_2, J=3.8 Hz, J=9.9 Hz, dd), 3.35 (1H, H_4, J=9.6 Hz, t), 2.96 (2H, $CH_{2\_Nspacer}$, J=7.5 Hz, t), 1.69-1.56 (4H, CH_{2_spacer}, m), 1.45-1.34 (4H, CH_{2_spacer}, m).

^{13}C-NMR(101 MHz, CD_3CN), δ: 98.6 (C_1), 76.2-76.1 ($CH_{glycerol}$), 73.8 (C_3), 72.7 (C_5), 72.4 (C_2), 71.7-71.6 ($CH_{glycerol}$), 70.6 (C_4), 70.5-70.3 ($CH_{glycerol}$), 67.3-66.9 ($CH_{2\_glycerol}$), 66.1 ($CH_{2\_glycerol}$), 65.3 ($CH_{2glycerol}$), 62.8 ($CH_{2\_glycerol}$), 61.4 (C_6), 42.1 ($CH_{2\_Nspacer}$), 32.0, 29.2, 27.7, 27.1 ($CH_{2spacer}$).
^{31}P-NMR(162 MHz, CD_3CN), δ: 1.62, 1.84, 1.94, 2.04.
HRMS: $C_{30}H_{67}NO_{36}P_6 + H^+$ required 1204.1941, found 1204.1956

(GroP)₂(GlcGroP)(GroP)₃-Spacer or Hexamer (3)

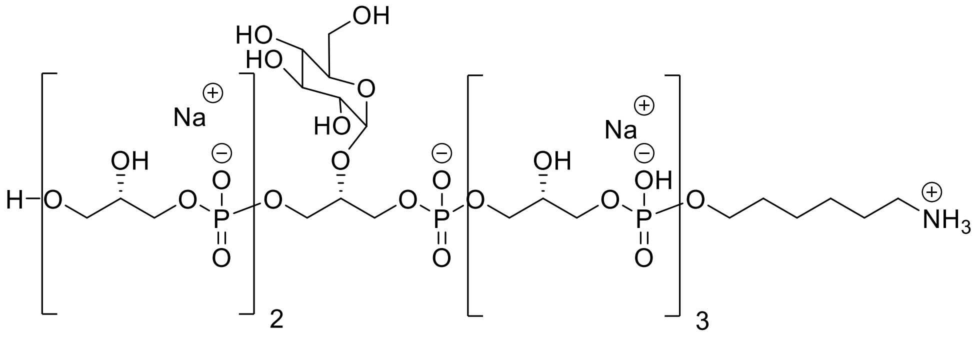

Compound **25** (6 μmol) was deprotected following the general procedure. The final product **3** was obtained in 62% yield (3.7 μmol).

^{1}H-NMR (850 MHz, CD_3CN), δ: 5.14 (1H, H_1, J=3.8 Hz, d), 4.11-4.04 (1H, $CH_{glycerol}$, m), 4.05-3.95 (8H, 4 x $CH_{glycerol}$, 2 x $CH_{2\_glycerol}$, m), 3.95-3.78 (23H, 9 x $CH_{2\_glycererol}$, H_6, $CH_{2\_Ospacer}$, H_5, $CH_{glycerol}$, m), 3.76-3.68 (2H, H_6, H_3, m), 3.64 (1H, $CHH_{glycerol}$, J=4.3 Hz, J=11.8 Hz, dd), 3.56 (1H, $CHH_{glycerol}$, J=6.1 Hz, J=11.8 Hz, dd) 3.50 (1H, H_2, J=3.8 Hz, J=9.9 Hz, dd), 3.35 (1H, H_4, J=9.6 Hz, t), 2.96 (2H, $CH_{2\_Nspacer}$, J=7.5 Hz, t), 1.69-1.56 (4H, CH_{2_spacer}, m), 1.45-1.34 (4H, CH_{2_spacer}, m).
^{13}C-NMR(101 MHz, CD_3CN), δ: 98.6 (C_1), 76.2-76.1 ($CH_{glycerol}$), 73.8 (C_3), 72.7 (C_5), 72.4 (C_2), 71.7-71.6 ($CH_{glycerol}$), 70.6 (C_4), 70.5-70.3 ($CH_{glycerol}$), 67.3-66.9 ($CH_{2\_glycerol}$), 66.1 ($CH_{2\_glycerol}$), 65.3 ($CH_{2glycerol}$), 62.8 ($CH_{2\_glycerol}$), 61.4 (C_6), 42.1 ($CH_{2\_Nspacer}$), 32.0, 29.2, 27.7, 27.1 ($CH_{2spacer}$).
^{31}P-NMR(162 MHz, CD_3CN), δ: 1.62, 1.84, 1.94, 2.04.
HRMS: $C_{30}H_{67}NO_{36}P_6 + H^+$ requires 1204.1941, found 1204.1951

(GroP)₃(GlcGroP)(GroP)₂-Spacer or Hexamer (4)

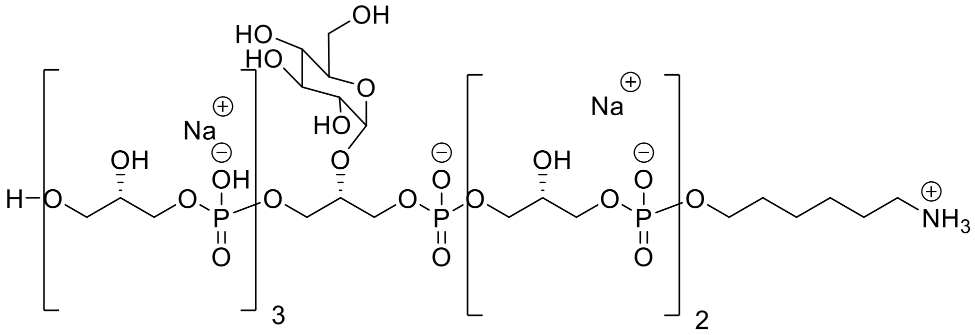

Compound **26** (9 μmol) was deprotected following the general procedure. The final product **4** was obtained in 81% yield (7.3 μmol).

^{1}H-NMR (850 MHz, CD_3CN), δ: 5.14 (1H, H_1, J=3.8 Hz, d), 4.11-4.04 (1H, $CH_{glycerol}$, m), 4.05-3.95 (8H, 4 x $CH_{glycerol}$, 2 x $CH_{2\_glycerol}$, m), 3.95-3.78 (23H, 9 x $CH_{2\_glycererol}$, H_6, $CH_{2\_Ospacer}$, H_5, $CH_{glycerol}$, m), 3.76-3.68 (2H, H_6, H_3, m), 3.64 (1H, $CHH_{glycerol}$, J=4.3 Hz, J=11.8 Hz, dd), 3.56 (1H, $CHH_{glycerol}$, J=6.1 Hz, J=11.8 Hz, dd) 3.50 (1H, H_2, J=3.8 Hz, J=9.9 Hz, dd), 3.35 (1H, H_4, J=9.6 Hz, t), 2.96 (2H, $CH_{2\_Nspacer}$, J=7.5 Hz, t), 1.69-1.56 (4H, CH_{2_spacer}, m), 1.45-1.34 (4H, CH_{2_spacer}, m).

^{13}C-NMR(101 MHz, CD_3CN), δ: 98.6 (C_1), 76.2-76.1 ($CH_{glycerol}$), 73.8 (C_3), 72.7 (C_5), 72.4 (C_2), 71.7-71.6 ($CH_{glycerol}$), 70.6 (C_4), 70.5-70.3 ($CH_{glycerol}$), 67.3-66.9 ($CH_{2\_glycerol}$), 66.1 ($CH_{2\_glycerol}$), 65.3 ($CH_{2glycerol}$), 62.8 ($CH_{2\_glycerol}$), 61.4 (C_6), 42.1 ($CH_{2\_Nspacer}$), 32.0, 29.2, 27.7, 27.1 ($CH_{2spacer}$).
^{31}P-NMR(162 MHz, CD_3CN), δ: 1.62, 1.84, 1.94, 2.04.
HRMS: $C_{30}H_{67}NO_{36}P_6 + H^+$ required 1204.1941, found 1204.1949

(GroP)$_4$(GlcGroP)(GroP) -Spacer or Hexamer (5)

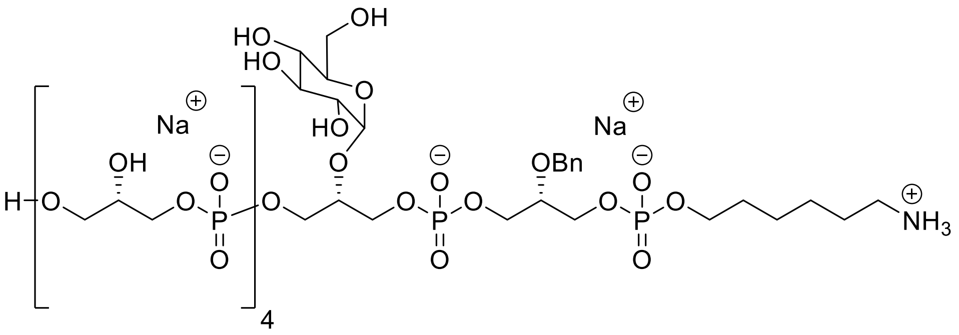

Compound **27** (16 μmol) was deprotected following the general procedure. The final product **5** was obtained in 78% yield (12 μmol).
^{1}H-NMR (850 MHz, CD_3CN), δ: 5.14 (1H, H_1, J=3.8 Hz, d), 4.11-4.04 (1H, $CH_{glycerol}$, m), 4.05-3.95 (8H, 4 x $CH_{glycerol}$, 2 x $CH_{2\_glycerol}$, m), 3.95-3.78 (23H, 9 x $CH_{2\_glycererol,}$ H_6, $CH_{2\_Ospacer}$, H_5, $CH_{glycerol}$, m), 3.76-3.68 (2H, H_6, H_3, m), 3.64 (1H, $CH\mathit{H}_{glycerol}$, J=4.3 Hz, J=11.8 Hz, dd), 3.56 (1H, $CH\mathit{H}_{glycerol}$, J=6.1 Hz, J=11.8 Hz, dd) 3.50 (1H, H_2, J=3.8 Hz, J=9.9 Hz, dd), 3.35 (1H, H_4, J=9.6 Hz, t), 2.96 (2H, $CH_{2\_Nspacer}$, J=7.5 Hz, t), 1.69-1.56 (4H, CH_{2_spacer}, m), 1.45-1.34 (4H, CH_{2_spacer}, m).
^{13}C-NMR(101 MHz, CD_3CN), δ: 98.6 (C_1), 76.2-76.1 ($CH_{glycerol}$), 73.8 (C_3), 72.7 (C_5), 72.4 (C_2), 71.7-71.6 ($CH_{glycerol}$), 70.6 (C_4), 70.5-70.3 ($CH_{glycerol}$), 67.3-66.9 ($CH_{2\_glycerol}$), 66.1 ($CH_{2\_glycerol}$), 65.3 ($CH_{2glycerol}$), 62.8 ($CH_{2\_glycerol}$), 61.4 (C_6), 42.1 ($CH_{2\_Nspacer}$), 32.0, 29.2, 27.7, 27.1 ($CH_{2spacer}$).
^{31}P-NMR(162 MHz, CD_3CN), δ: 1.62, 1.84, 1.94, 2.04.
HRMS: $C_{30}H_{67}NO_{36}P_6 + H^+$ required 1204.1941, found 1204.1957

(GroP)$_5$(GlcGroP)-Spacer or Hexamer (6)

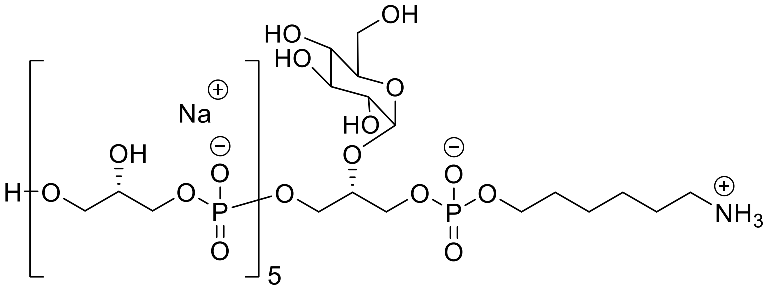

Compound **28** (21 μmol) was deprotected following the general procedure. The final product **6** was obtained in 68% yield (14 μmol).
^{1}H-NMR (850 MHz, CD_3CN), δ: 5.14 (1H, H_1, J=3.8 Hz, d), 4.11-4.04 (1H, $CH_{glycerol}$, m), 4.05-3.95 (8H, 4 x $CH_{glycerol}$, 2 x $CH_{2\_glycerol}$, m), 3.95-3.78 (23H, 9 x $CH_{2\_glycererol,}$ H_6, $CH_{2\_Ospacer}$, H_5, $CH_{glycerol}$, m), 3.76-3.68 (2H, H_6, H_3, m), 3.64 (1H, $CH\mathit{H}_{glycerol}$, J=4.3 Hz, J=11.8 Hz, dd), 3.56 (1H, $CH\mathit{H}_{glycerol}$, J=6.1 Hz, J=11.8 Hz, dd) 3.50 (1H, H_2, J=3.8 Hz, J=9.9 Hz, dd), 3.35 (1H, H_4, J=9.6 Hz, t), 2.96 (2H, $CH_{2\_Nspacer}$, J=7.5 Hz, t), 1.69-1.56 (4H, CH_{2_spacer}, m), 1.45-1.34 (4H, CH_{2_spacer}, m).
^{13}C-NMR(101 MHz, CD_3CN), δ: 98.6 (C_1), 76.2-76.1 ($CH_{glycerol}$), 73.8 (C_3), 72.7 (C_5), 72.4 (C_2), 71.7-71.6 ($CH_{glycerol}$), 70.6 (C_4), 70.5-70.3 ($CH_{glycerol}$), 67.3-66.9 ($CH_{2\_glycerol}$), 66.1 ($CH_{2\_glycerol}$), 65.3 ($CH_{2glycerol}$), 62.8 ($CH_{2\_glycerol}$), 61.4 (C_6), 42.1 ($CH_{2\_Nspacer}$), 32.0, 29.2, 27.7, 27.1 ($CH_{2spacer}$).
^{31}P-NMR(162 MHz, CD_3CN), δ: 1.62, 1.84, 1.94, 2.04.
HRMS: $C_{30}H_{67}NO_{36}P_6 + H^+$ requires 1204.1941, found 1204.1948

Generation and Serum analysis of microarrays

The amino-spacer equipped GTA-fragments were dissolved in spotting buffer (Nexterion Spot, Schott Nexterion) with 10% DMSO in 384-wells V-bottom plates (Genetix, New Milton, UK). The GTA-fragments were printed in three final concentrations (30µM, 10µM and 3µM) in triplicate on epoxysilane-coated glass slides (Slide E, Schott, Nexterion) by contact printing using the Omnigrid 100 microarrayer (Genomic Solutions, Ann Arbor, MI) equipped with SMP3 pins with uptake channels that deposit 0.7 nl at each contact. The slides were rested in a high humidity chamber for 18 hours and were stored in the dark until used. The slides were washed with PBS (3x) and subsequently all unreacted sites on the arrays were blocked by shaking the slides for 1 hour with ethanolamine (0.25 ml, 0.05M in PBS containing 20 mg/ml of BSA). The slides were flushed with PBS containing 5% of Tween® 20 and PBS containing 1% of Tween® 20 subsequently. After removal of the PBS containing 1% of Tween® 20, the arrays were shaken with the primary antibody dilutions (0.25 ml, diluted with PBS containing 1% of Tween® 20 and 10 mg/ml of BSA) for 60 minutes. Serum obtained from rabbits immunized with native LTA isolated from *E. faecalis* strain 12030 was used at a 1:1000 dilution, while rabbit serum raised against the previously reported BSA-WH7 at a 1:500 dilution. The slides were flushed with PBS containing 5% of Tween® 20 and PBS containing 1% of Tween® 20 subsequently. After removal of the PBS containing 1% of Tween® 20, slides were shaken with anti-rabbit-IgG secondary antibodies, labeled with DyLight 550 reporter groups (0.25 ml, 0.5 µg/ml final dilution in PBS containing 1% of Tween® 20 and 10 mg/ml of BSA) for 30 minutes in the dark. The slides were flushed with PBS containing 5% of Tween® 20, PBS and MilliQ subsequently. The slides were dried by centrifugation and were analyzed on fluorescence on 532 nm and 635 nm using a G2565BA scanner. Data and image analyses were performed with GenePix Pro 7.0 software (Molecular Devices, Sunnyvale, CA, USA) as described previously (J. Proteome Res., 8 (2009), pp. 4301–4310). Fluorescence intensities were quantified and corrected for background/non-specific antibody adhesion by subtracting the fluorescence at blank spots, where only spotting buffer was printed without GTA fragment. The average of the triplicate spots was normalized to the highest intensity on the array and visualized in bar graphs using Microsoft Excel.

^{1}H, ^{13}C and ^{31}P NMR spectra

(S)-1-*O*-allyl-3-*O*-(*tert*-butyldiphenylsilyl)-*sn*-glycerol (11)

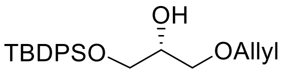

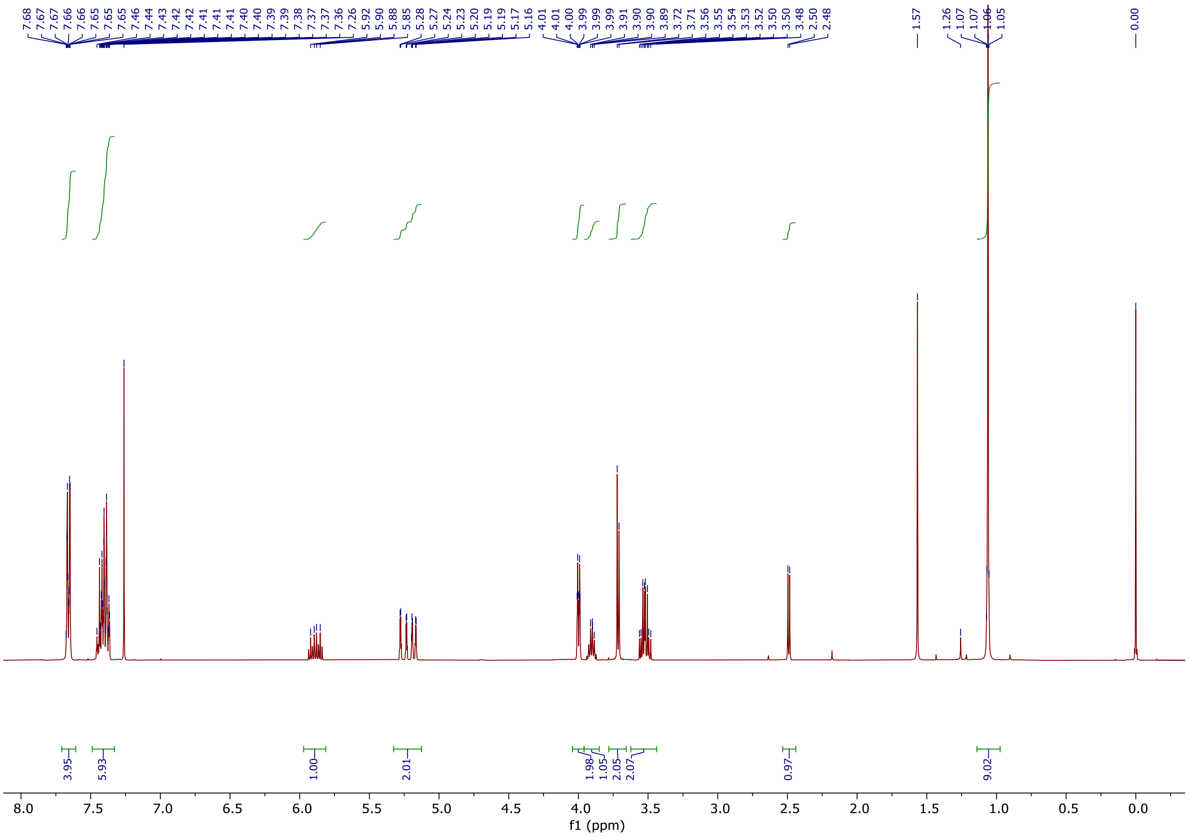

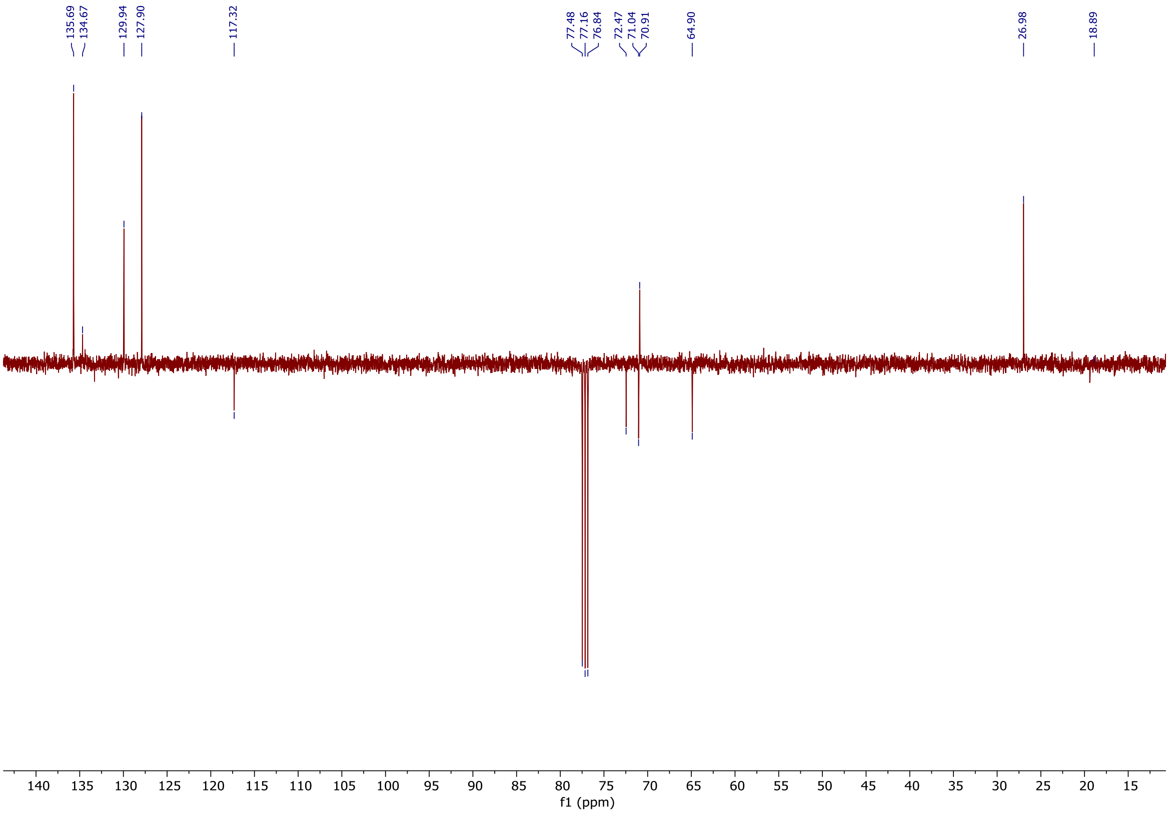

(R)-1-*O*-(*tert*-butyldiphenylsilyl)-3-*O*-allyl-*sn*-glycerol (12)

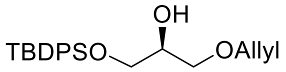

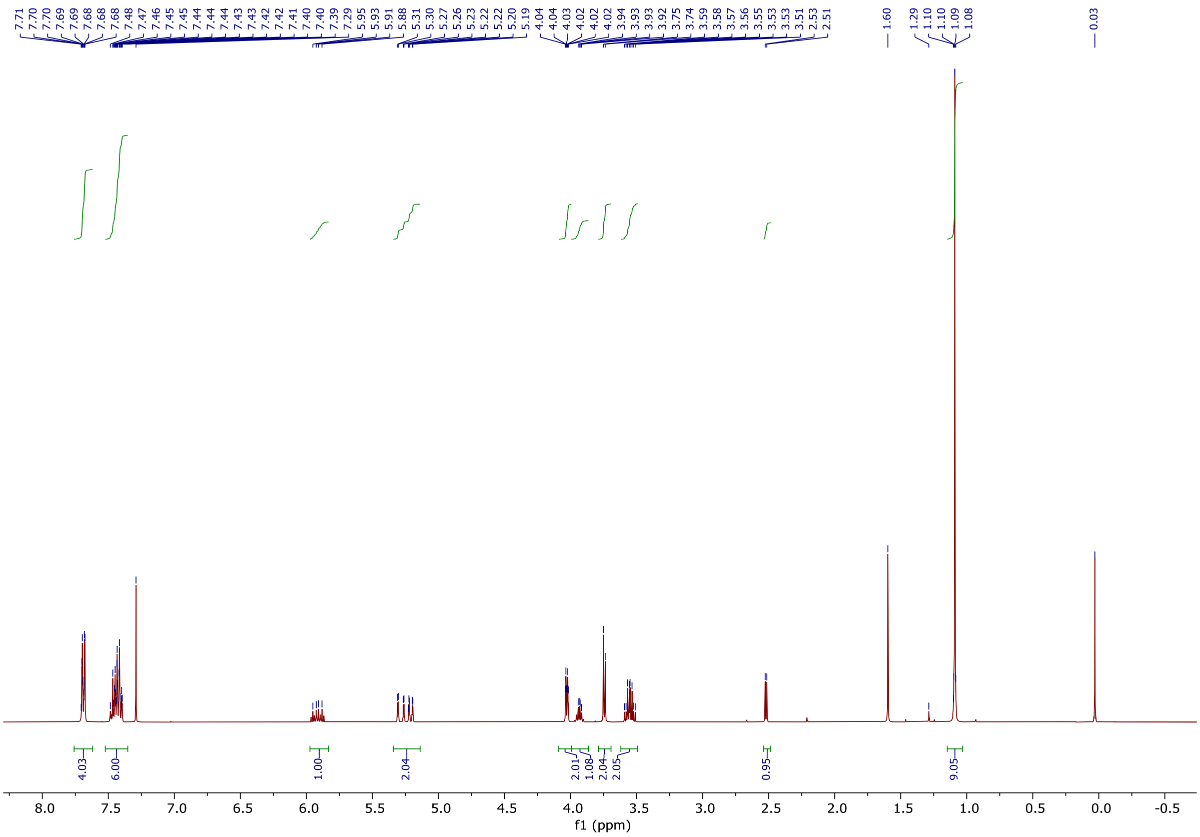

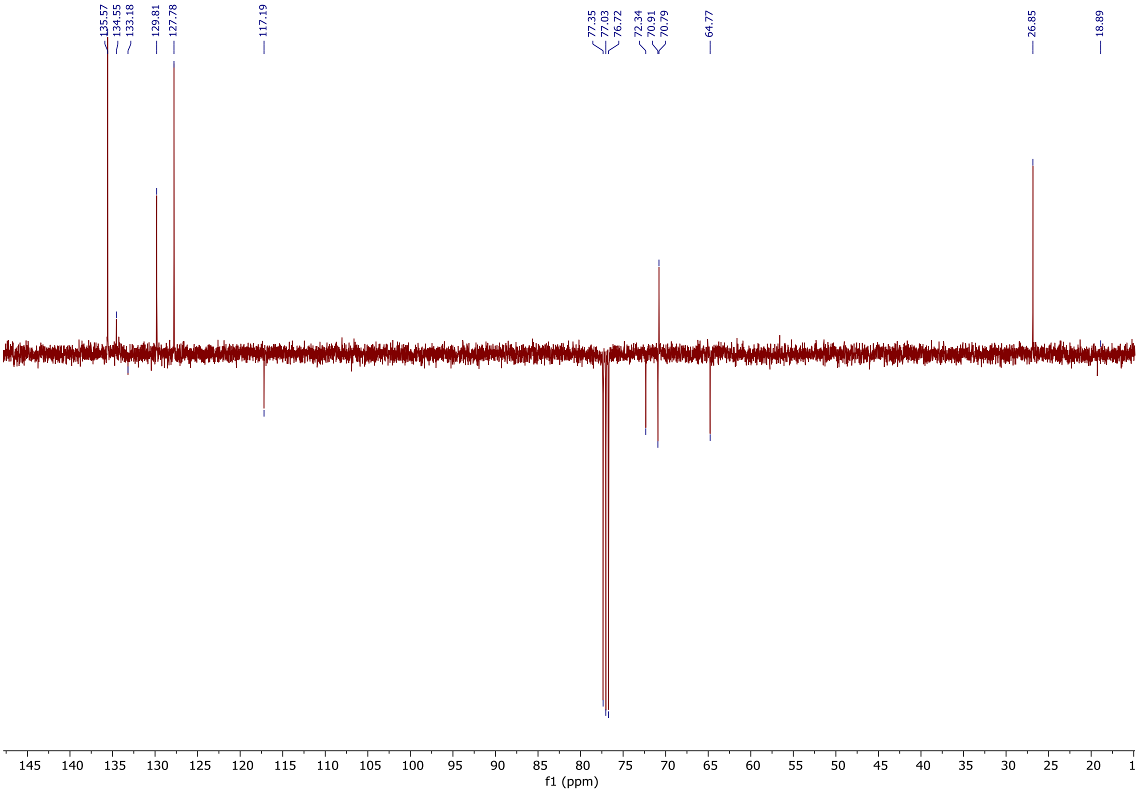

(R)-1-*O*-allyl-3-*O*-(4-methoxybenzyl) -*sn*-glycerol (13)

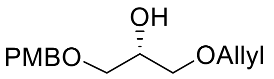

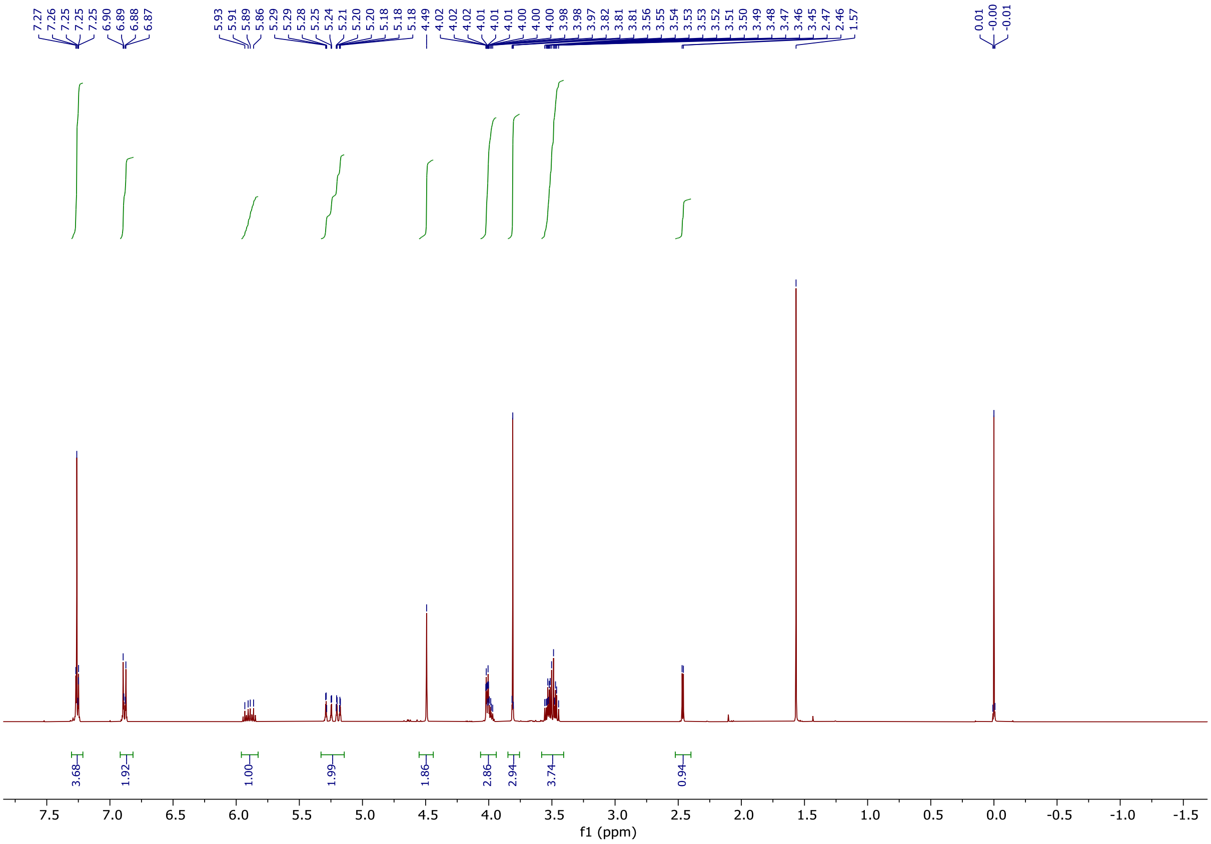

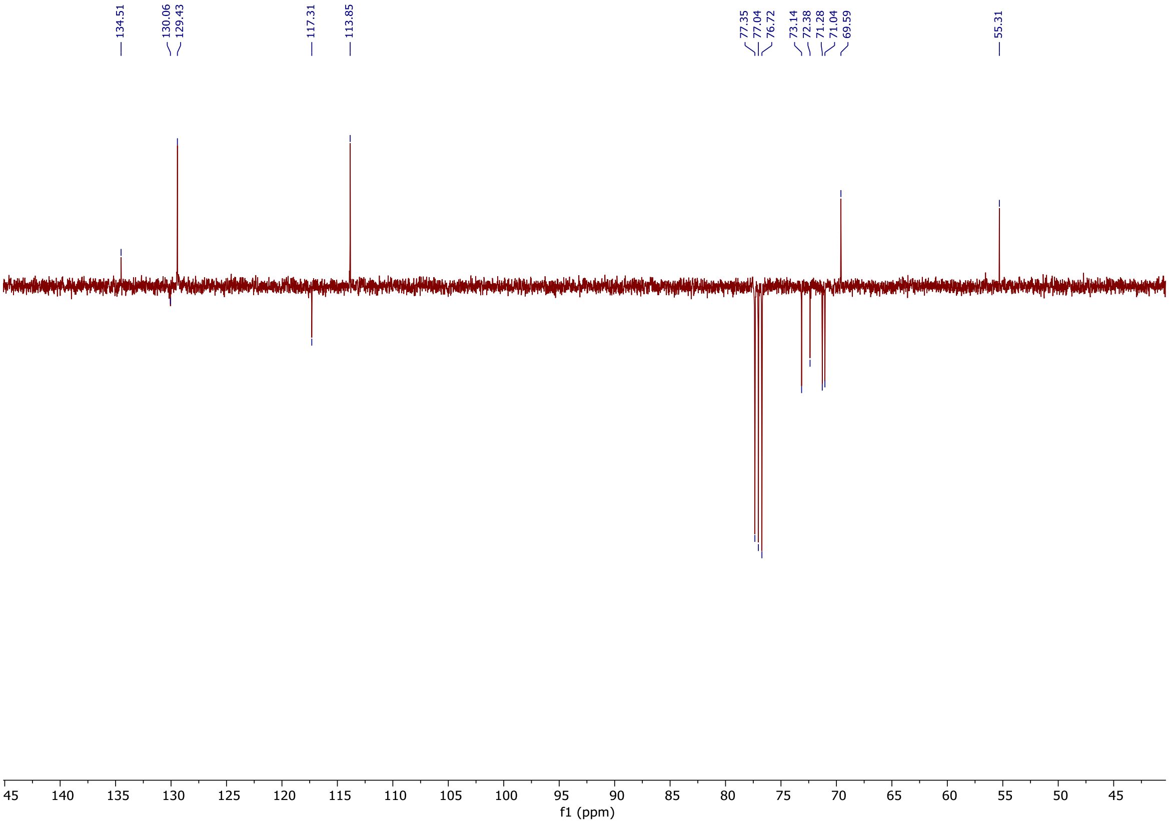

(S)-1-*O*-(4-methoxybenzyl)-3-*O*-allyl-*sn*-glycerol (14)

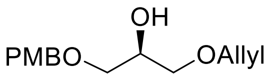

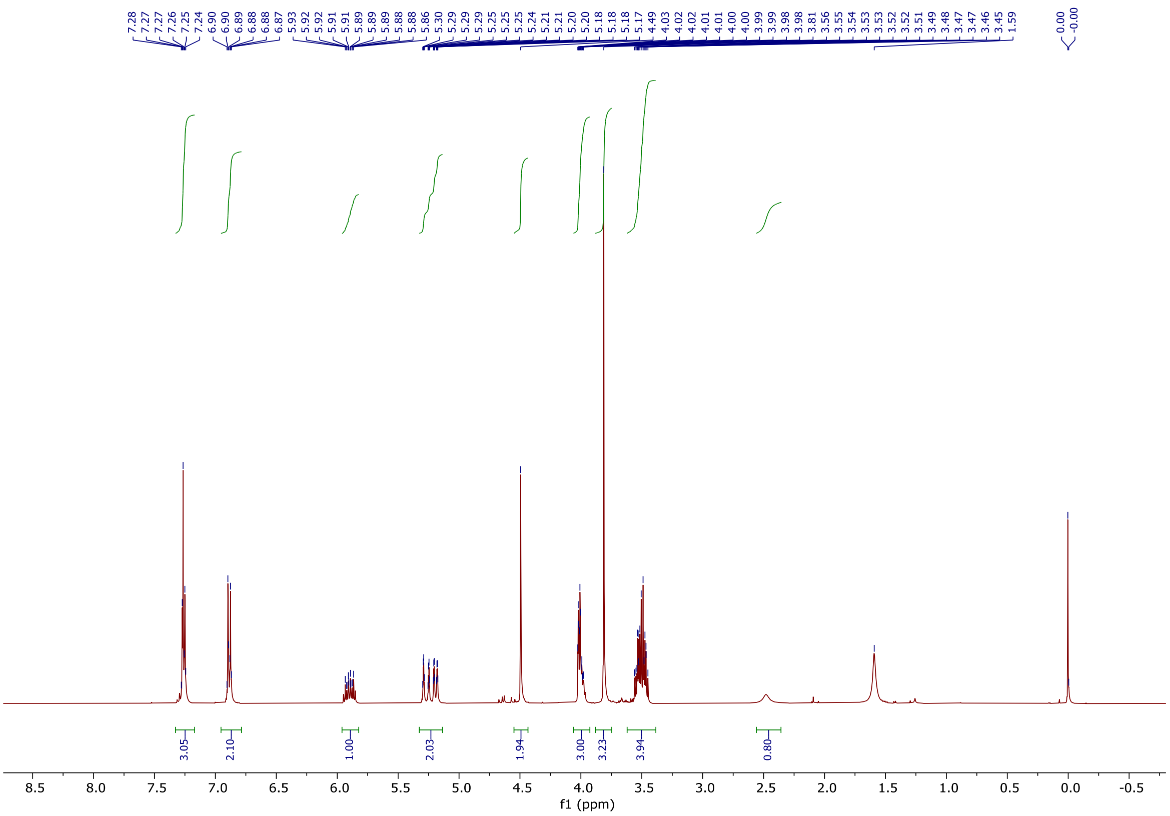

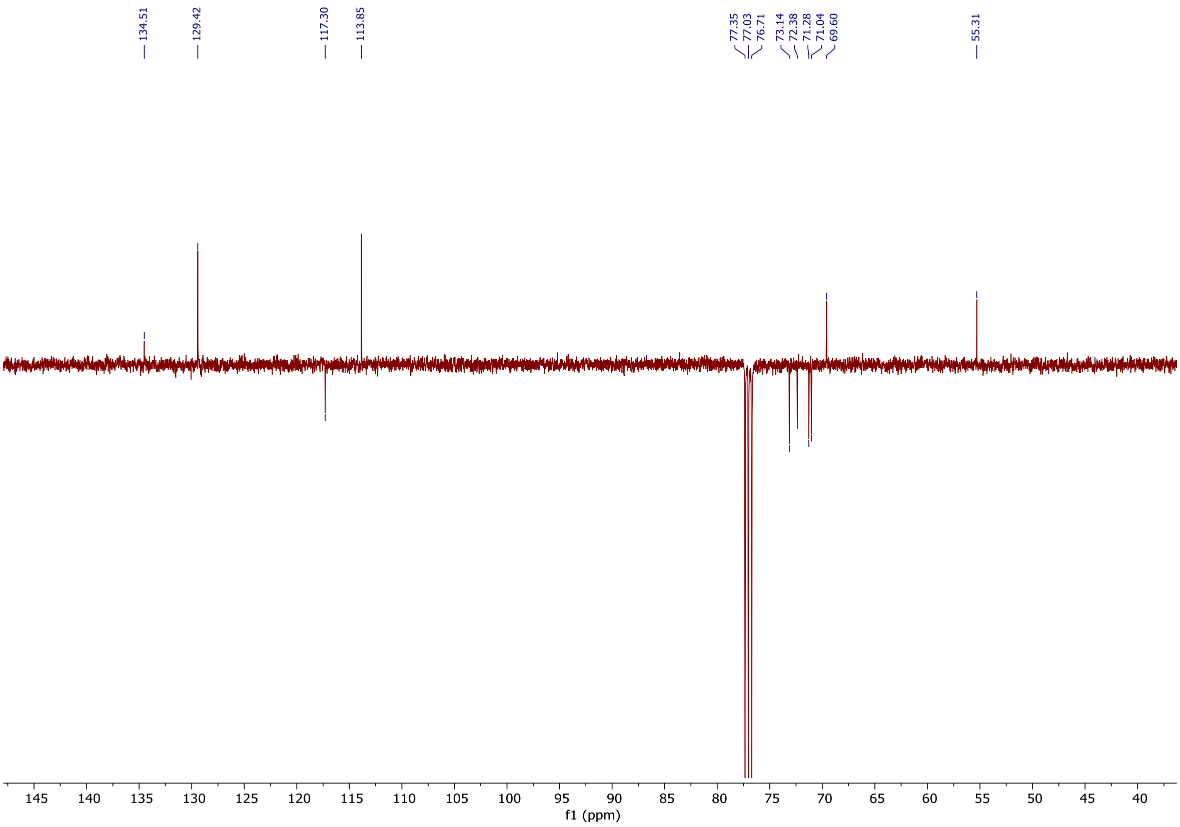

(R)-1-*O*-allyl-3-*O*-benzoyl-*sn*-glycerol (15)

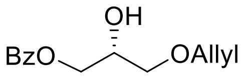

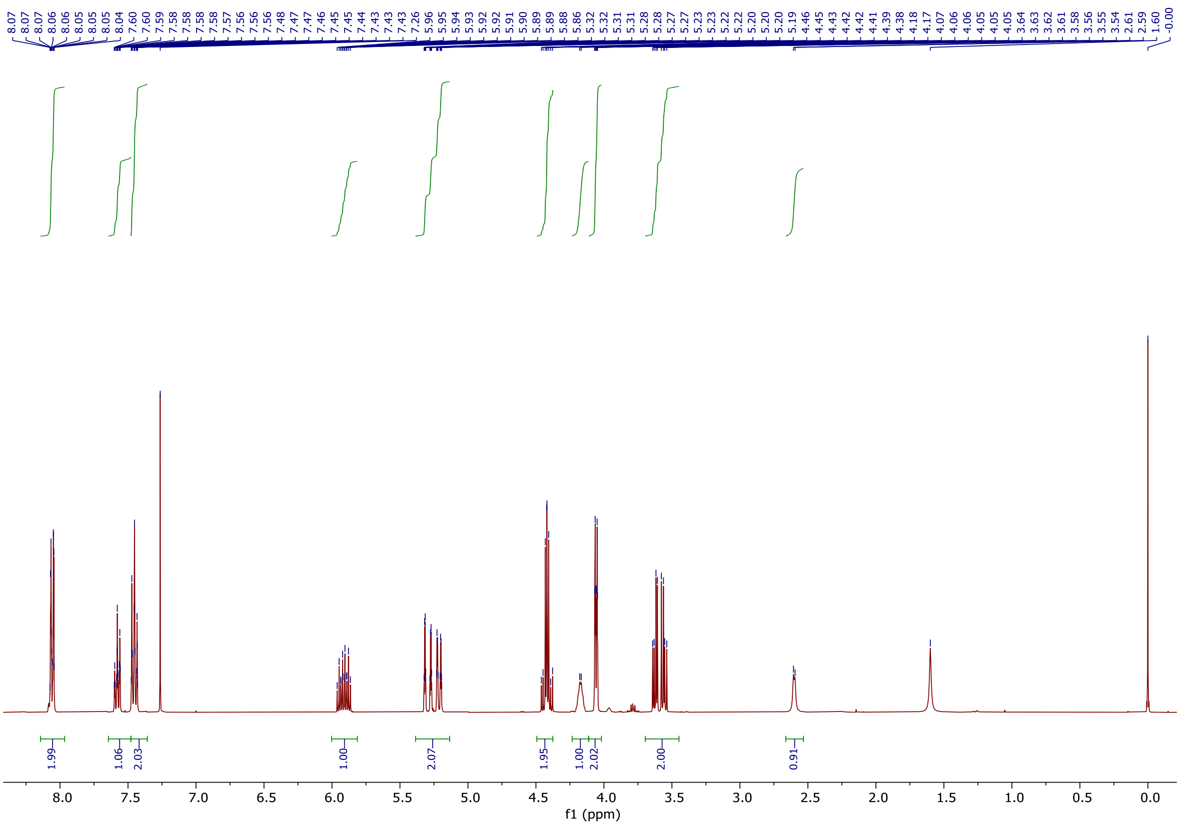

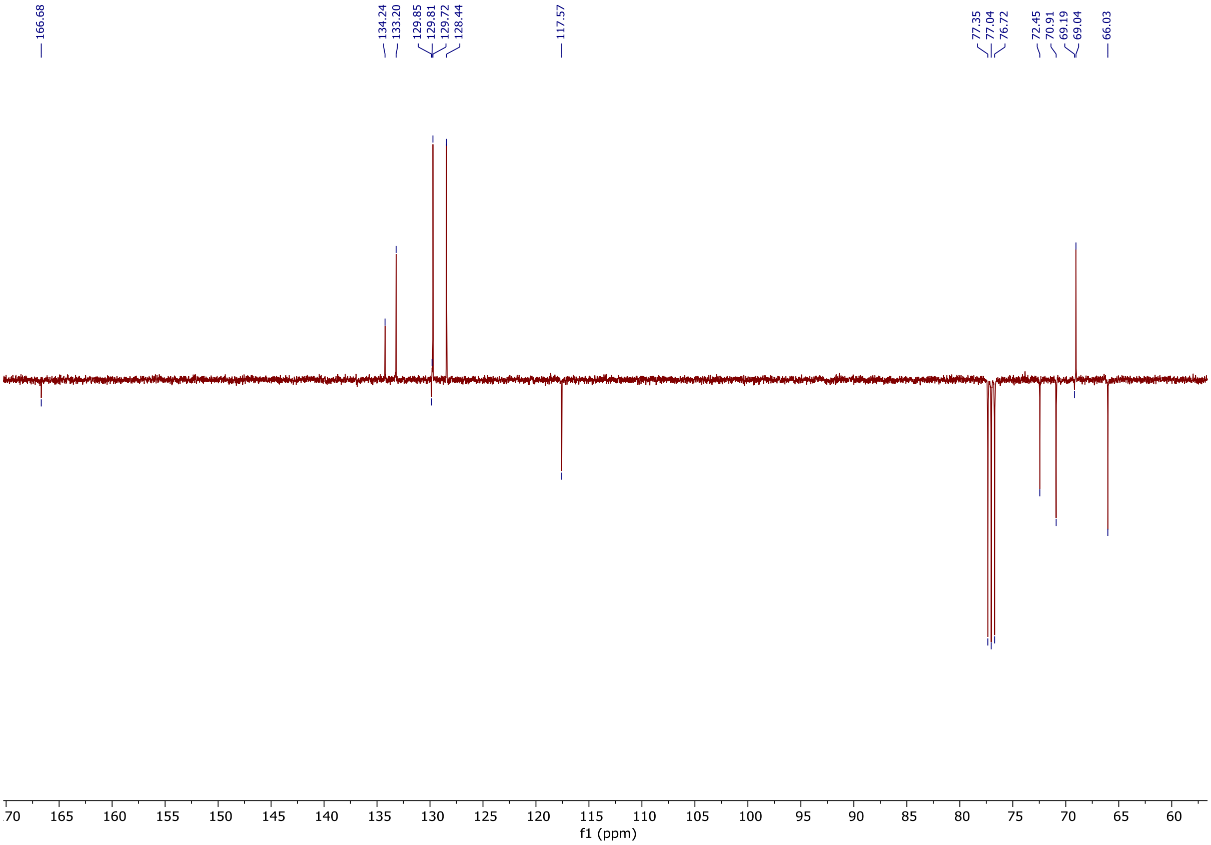

(S)-1-*O*-benzoyl-3-*O*-allyl-*sn*-glycerol (16)

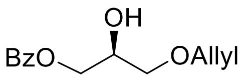

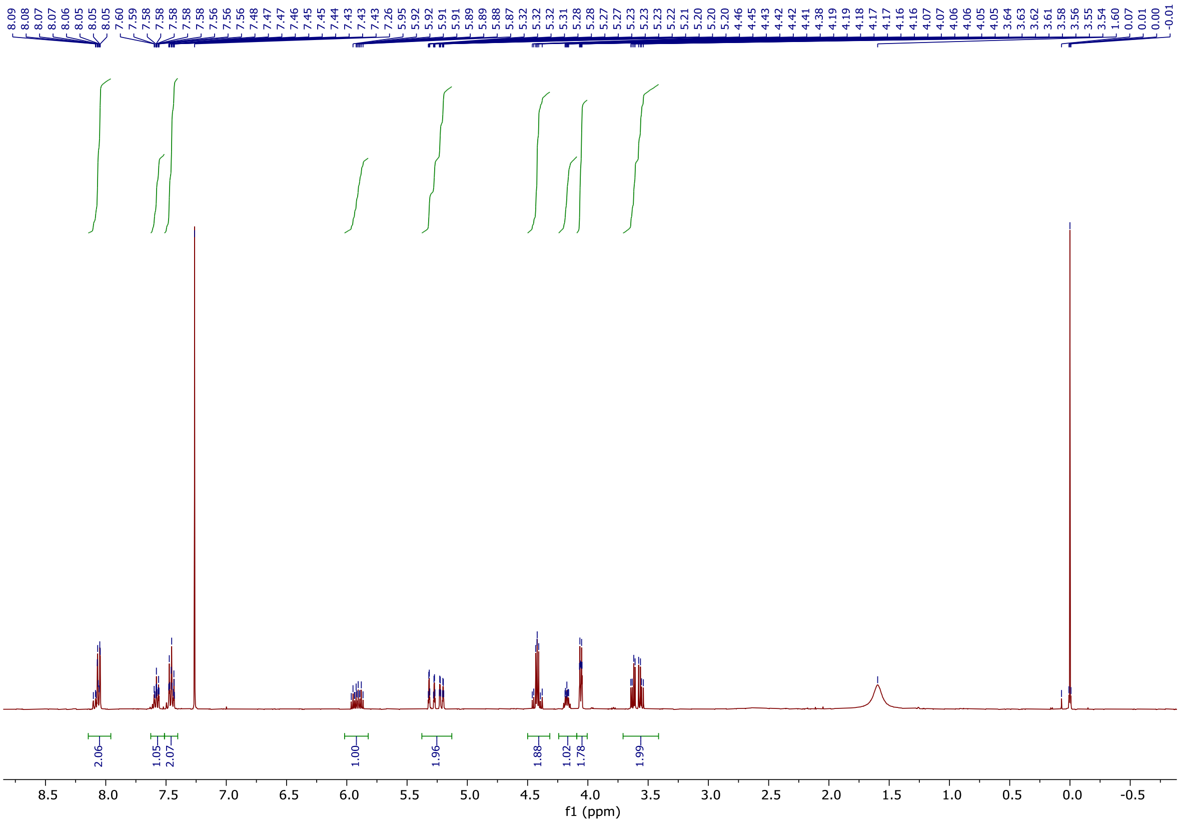

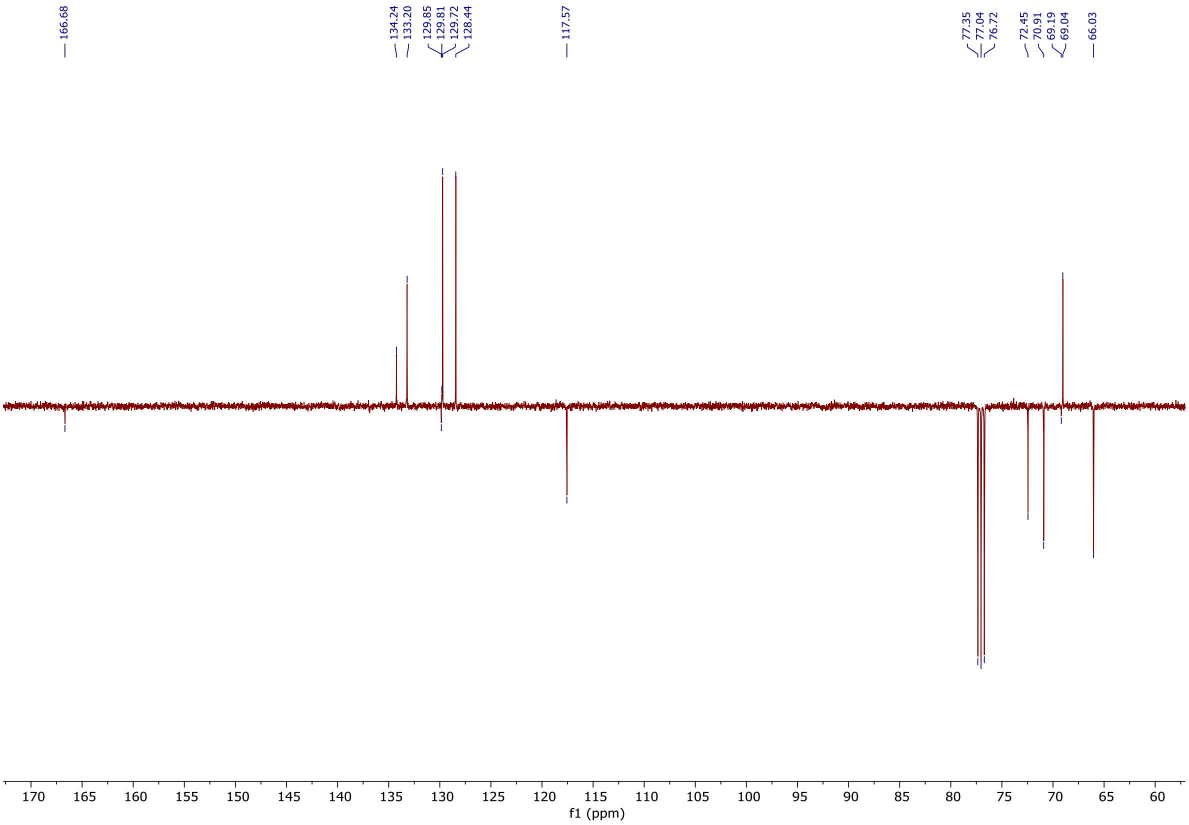

(S)-1-*O*-allyl-2-*O*-(2,3,4,6-*O*-benzyl-α-D-glucopyranosyl)-3-*O*-(*tert*-butyldiphenylsilyl-*sn*-glycerol (17)

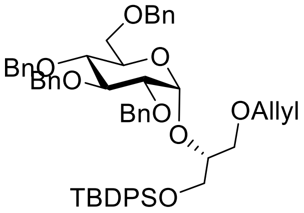

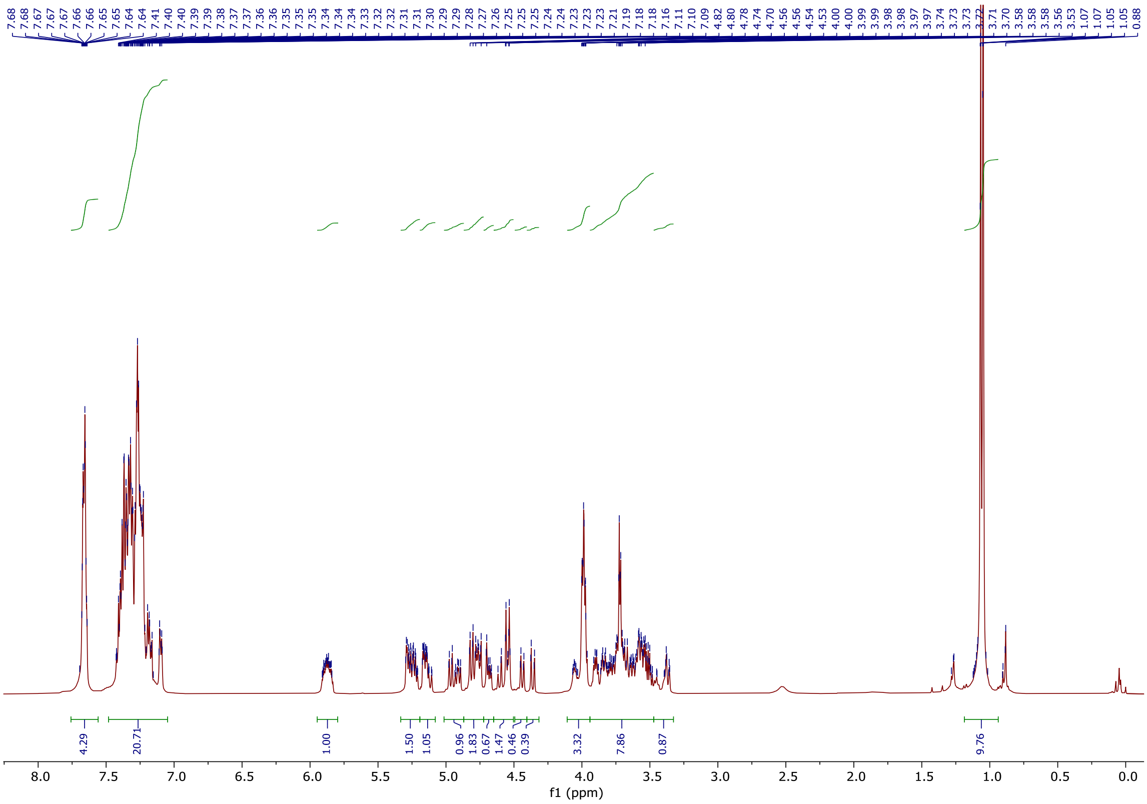

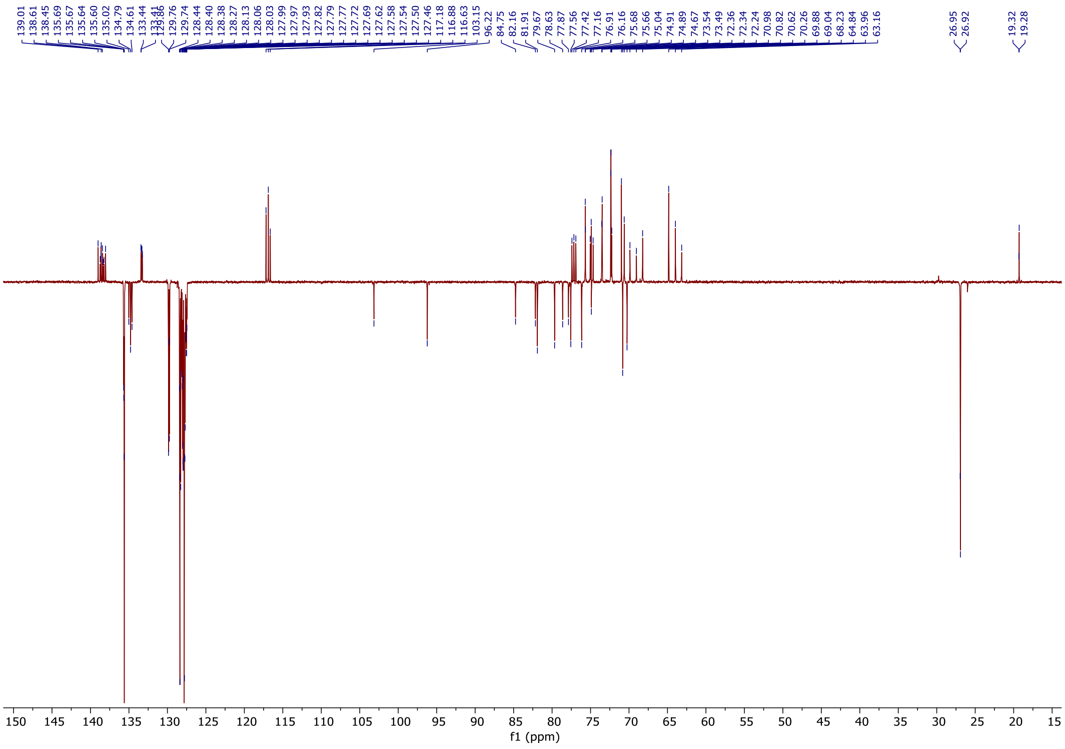

(R)-1-*O*-(*tert*-butyldiphenylsilyl)-2-*O*-(2,3,4,6-*O*-benzyl-α-D-glucopyranosyl)-3-*O*-allyl-*sn*-glycerol (18)

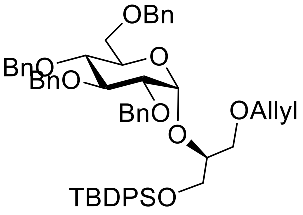

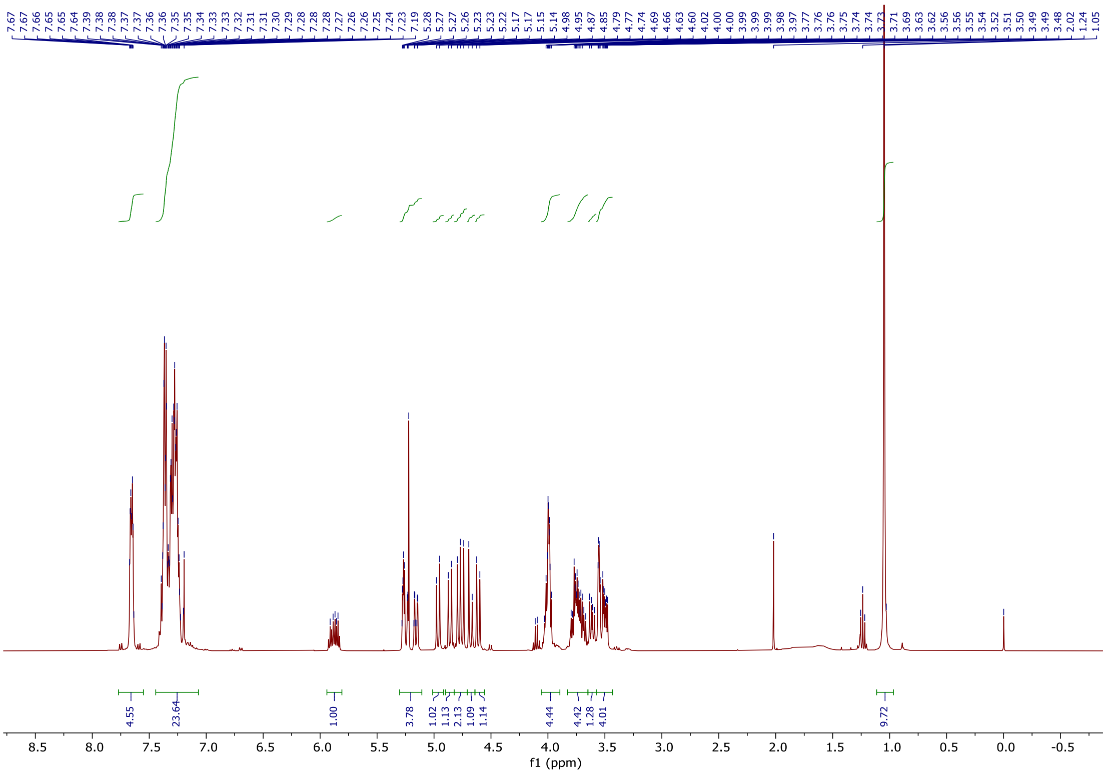

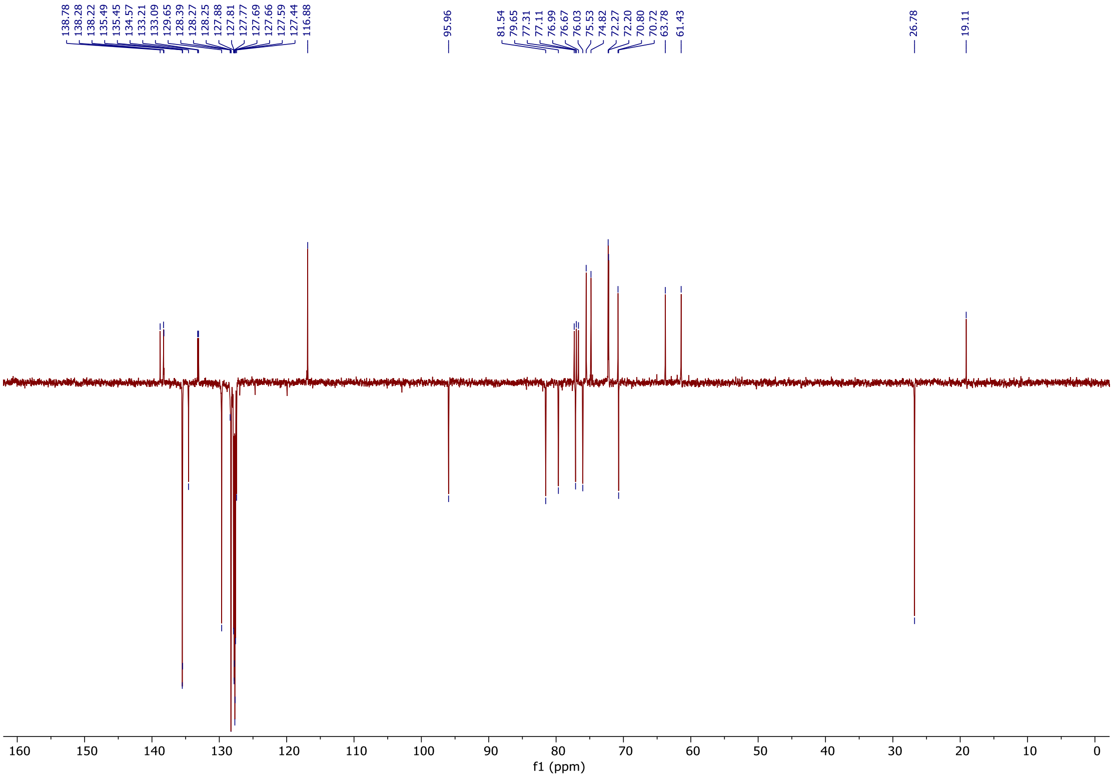

(R)-1-*O*-allyl-2-*O*-(2,3,4,6-*O*-benzyl-α-D-glucopyranosyl)-3-*O*-(4-methoxybenzyl)-*sn*-glycerol (19)

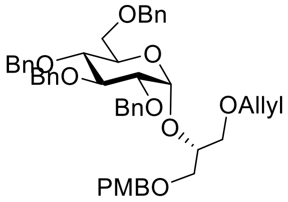

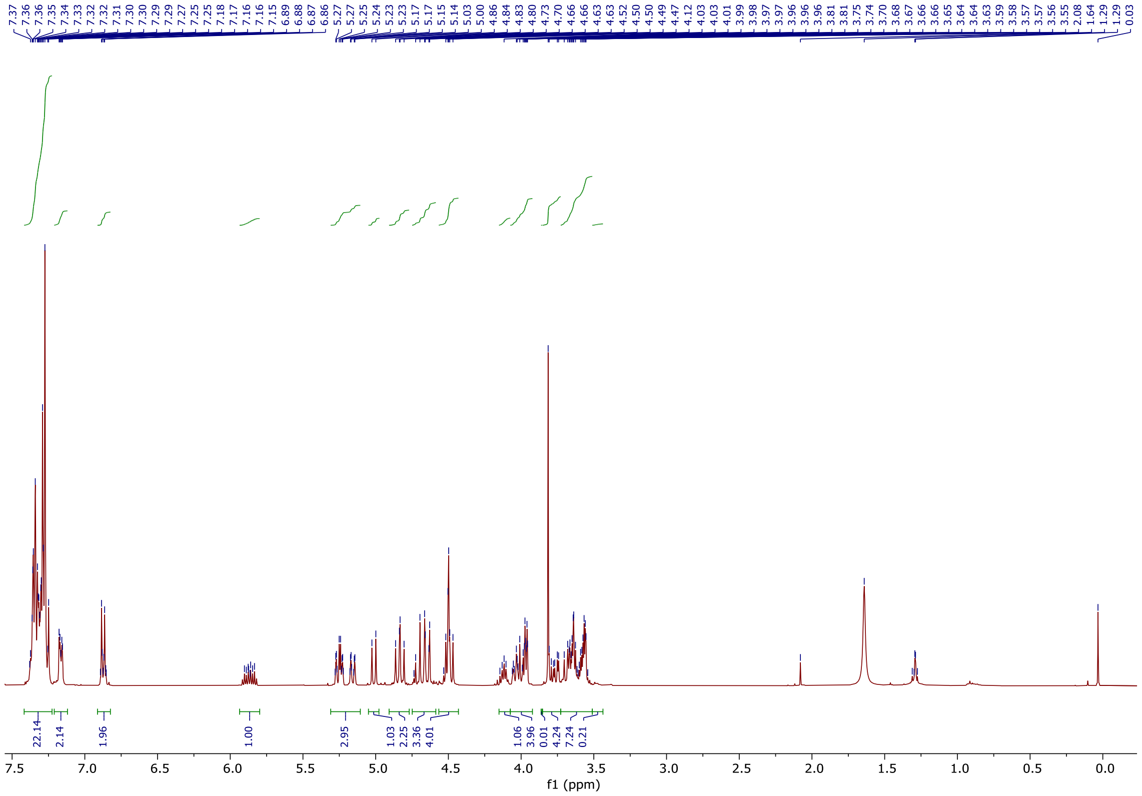

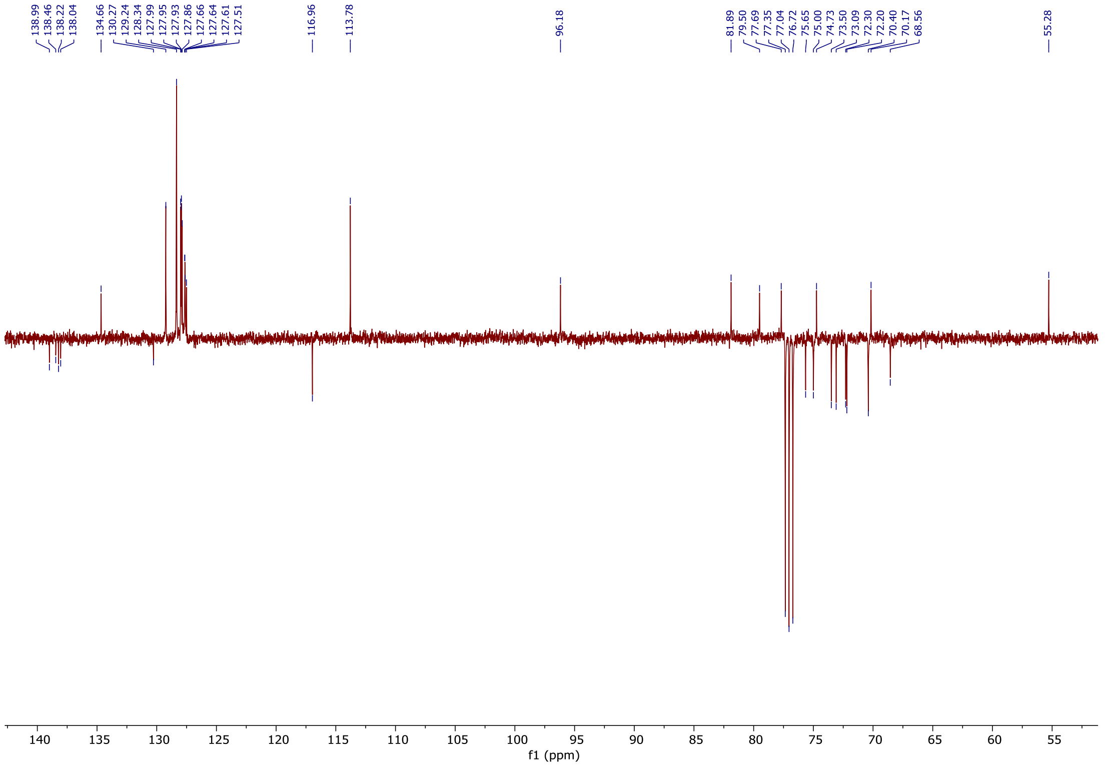

(S)-1-*O*-(4-methoxybenzyl)-2-*O*-(2,3,4,6-*O*-benzyl-α-D-glucopyranosyl)-3-*O*-allyl-*sn*-glycerol (20)

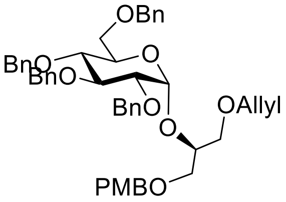

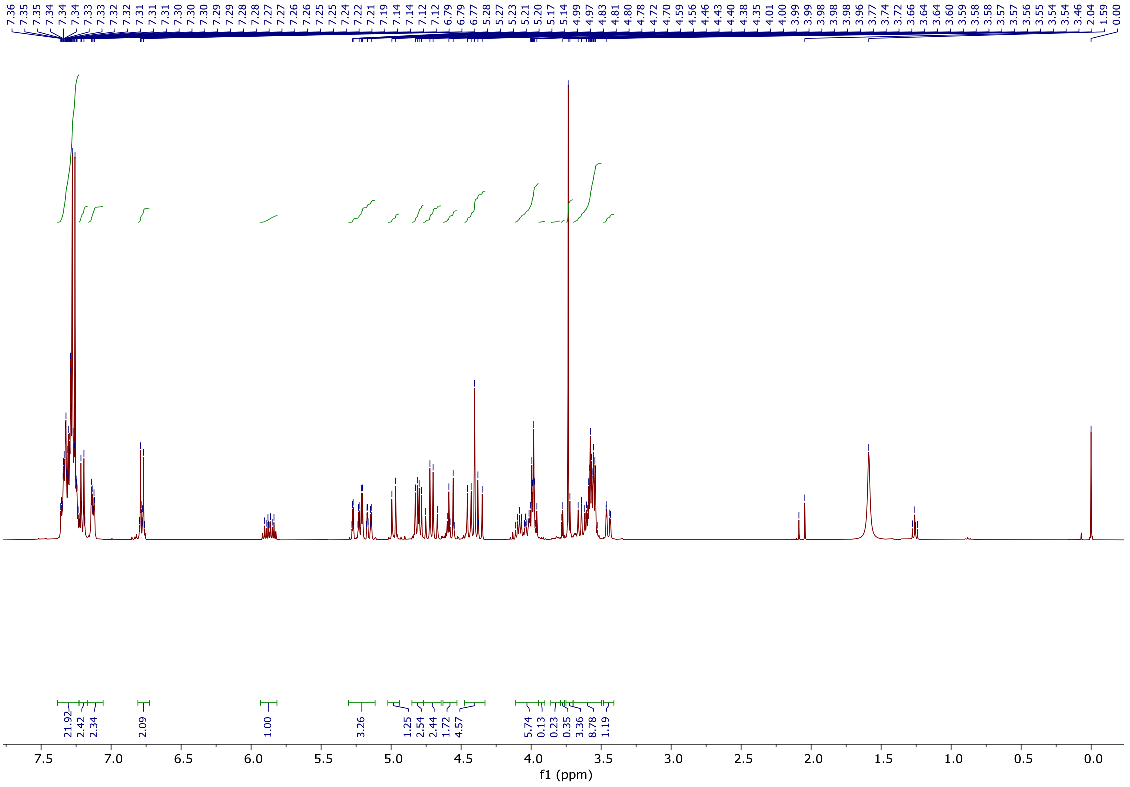

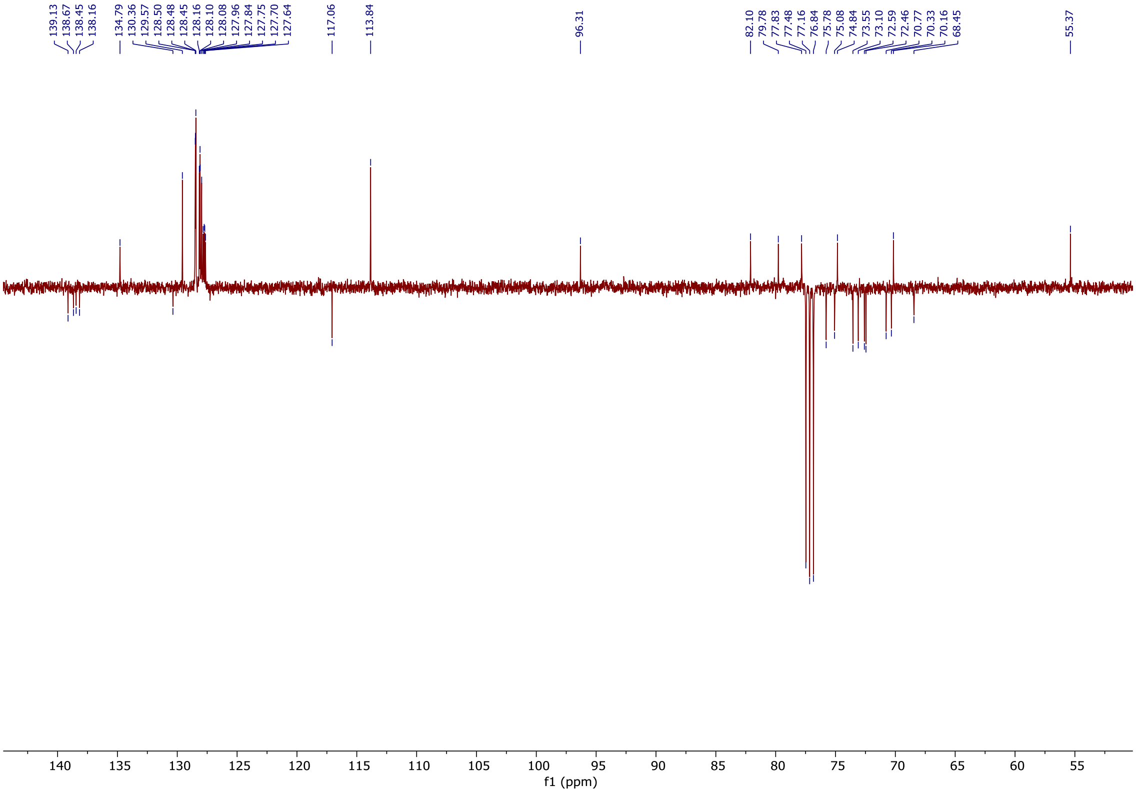

(R)-1-*O*-allyl-2-*O*-(2,3,4,6-*O*-benzyl-α-D-glucopyranosyl)-3-*O*-benzoyl-*sn*-glycerol (21)

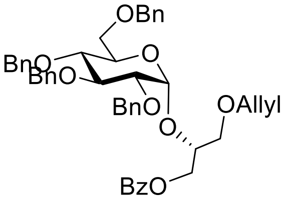

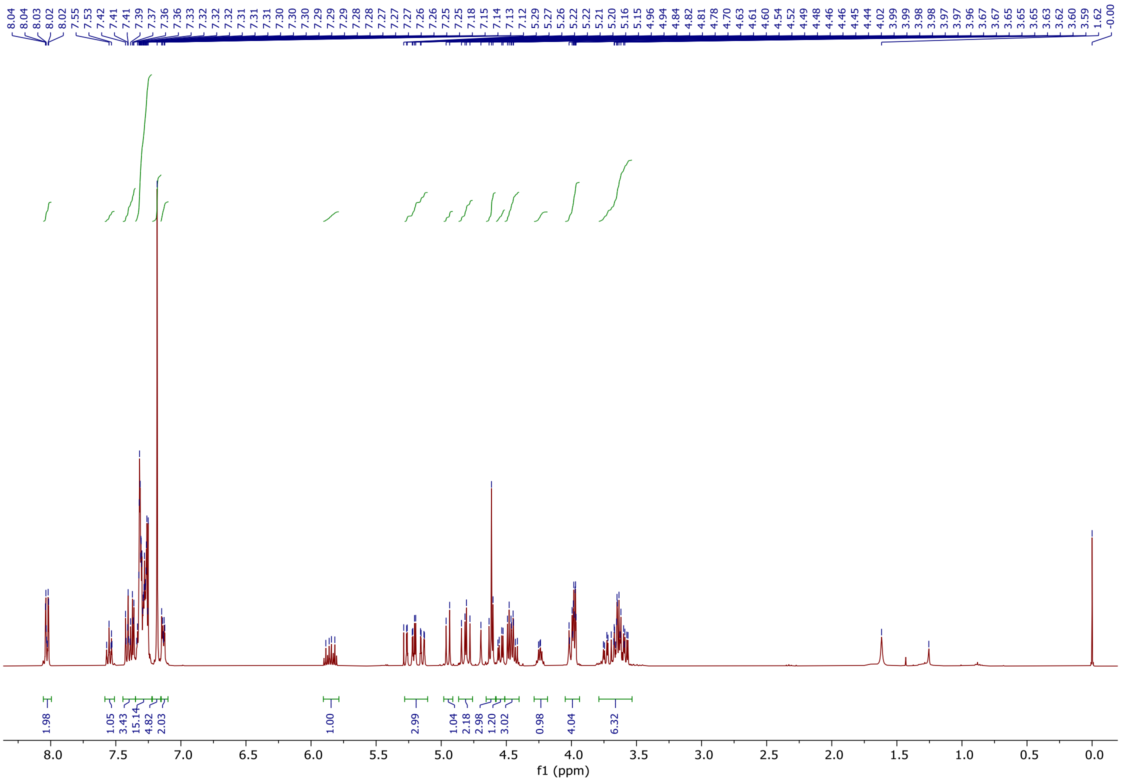

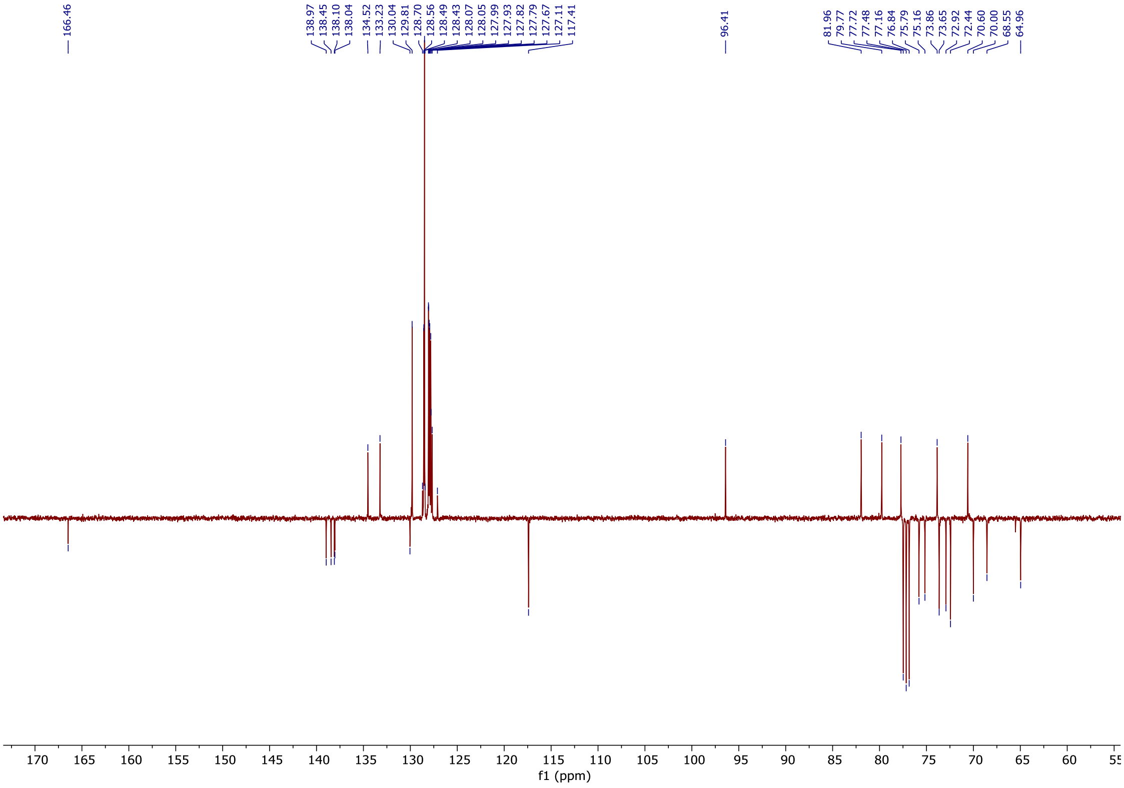

(S)-1-*O*-benzoyl-2-*O*-(2,3,4,6-*O*-benzyl-α-D-glucopyranosyl)-3-*O*-allyl-*sn*-glycerol (22)

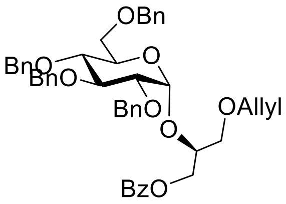

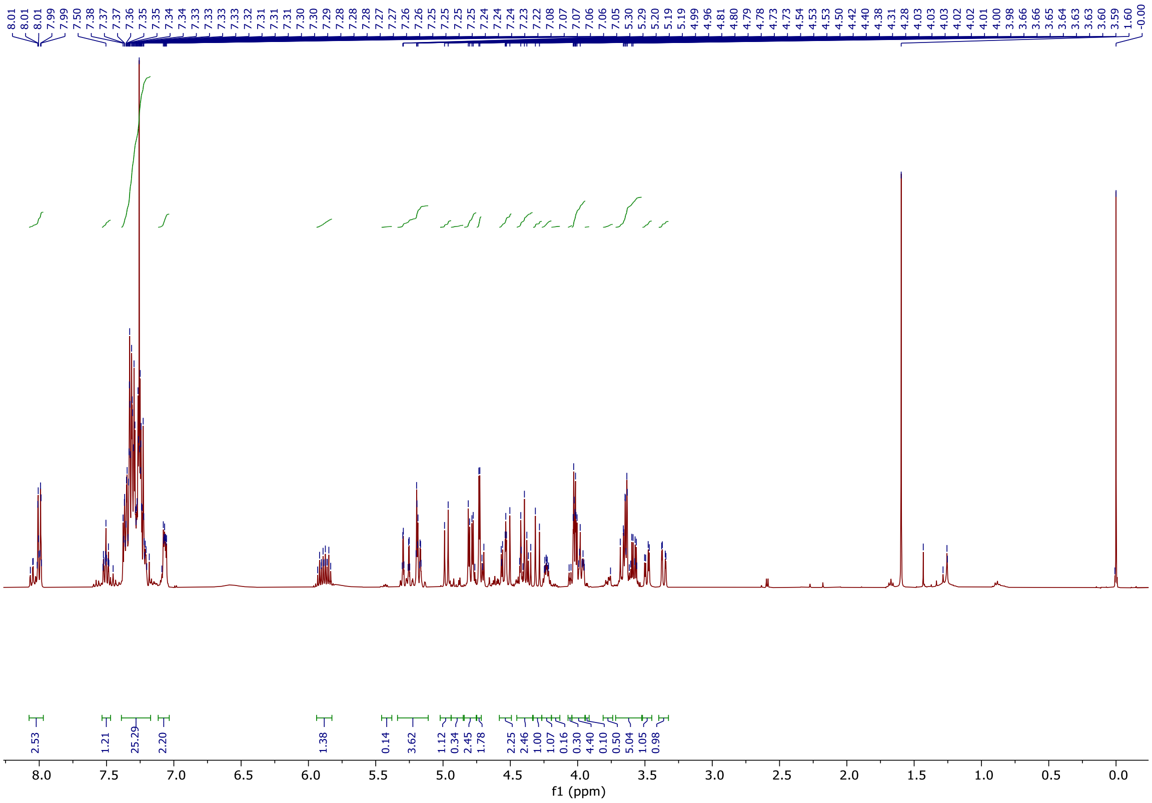

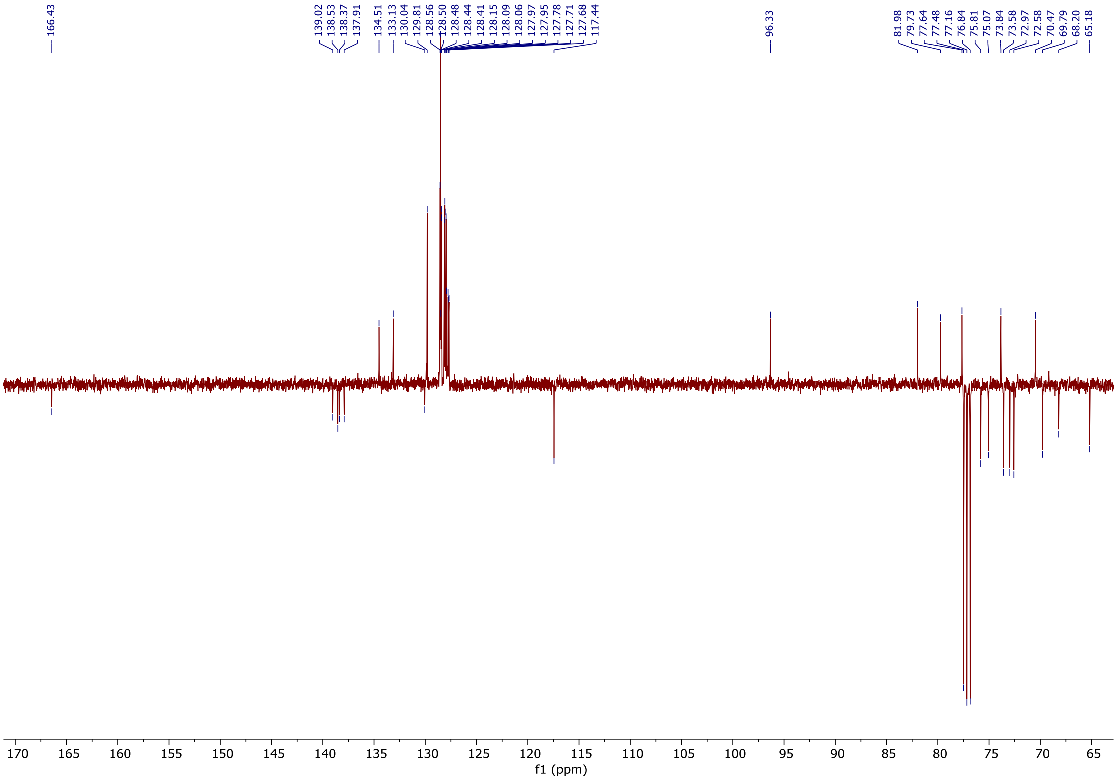

(R)-1-*O*-allyl-2-*O*-(2,3,4,6-*O*-benzyl-α-D-glucopyranosyl)-*sn*-glycerol (S3)

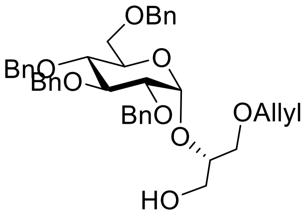

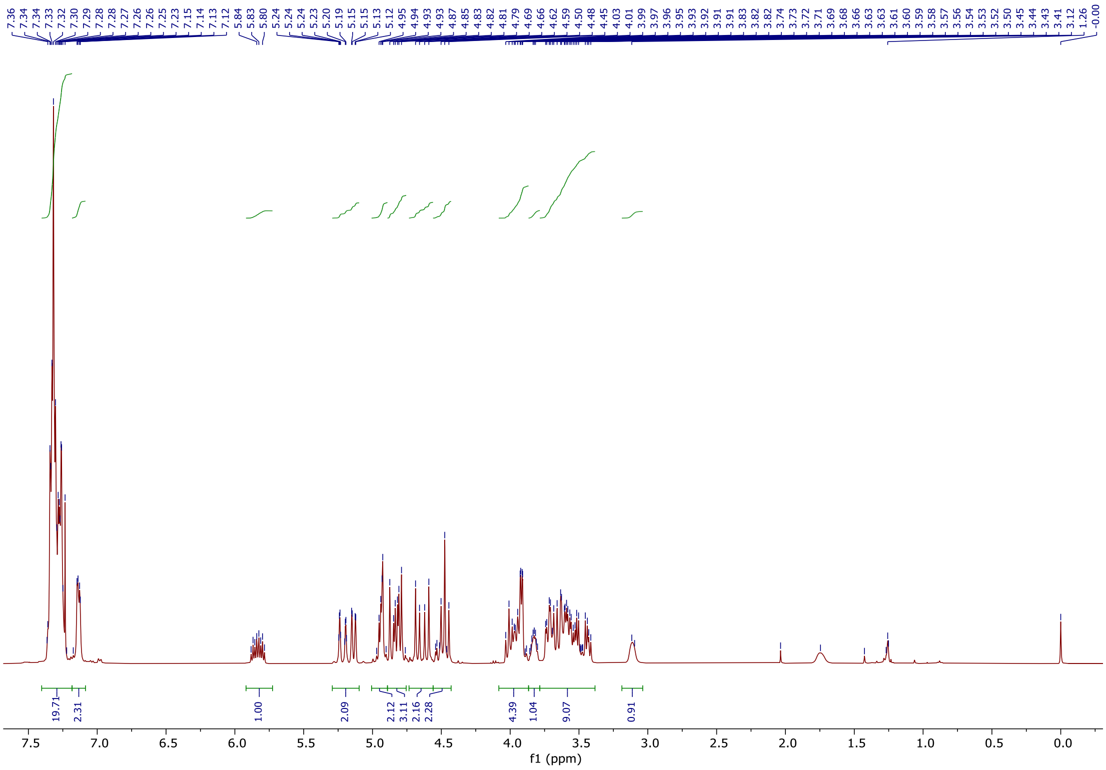

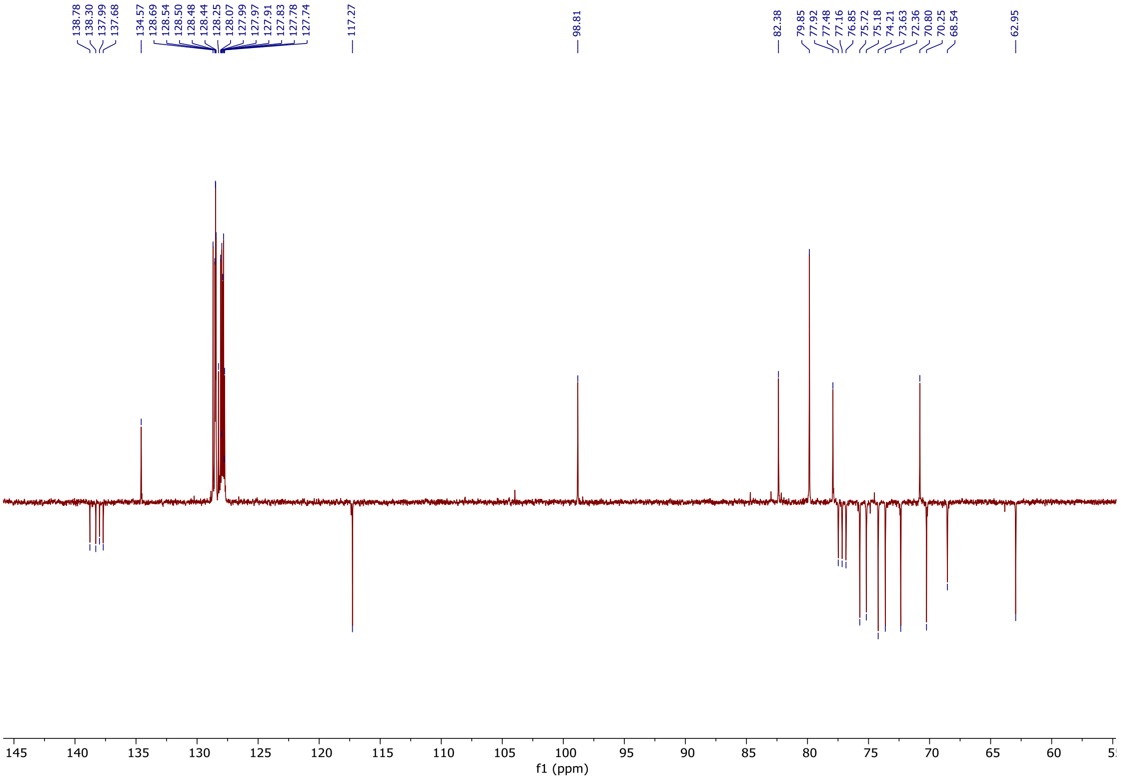

(R)-1-*O*-allyl-2-*O*-(2,3,4,6-*O*-benzyl-α-D-glucopyranosyl)-3-*O*(4,4'-dimethoxytrityl)-*sn*-glycerol (S4)

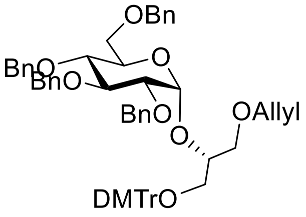

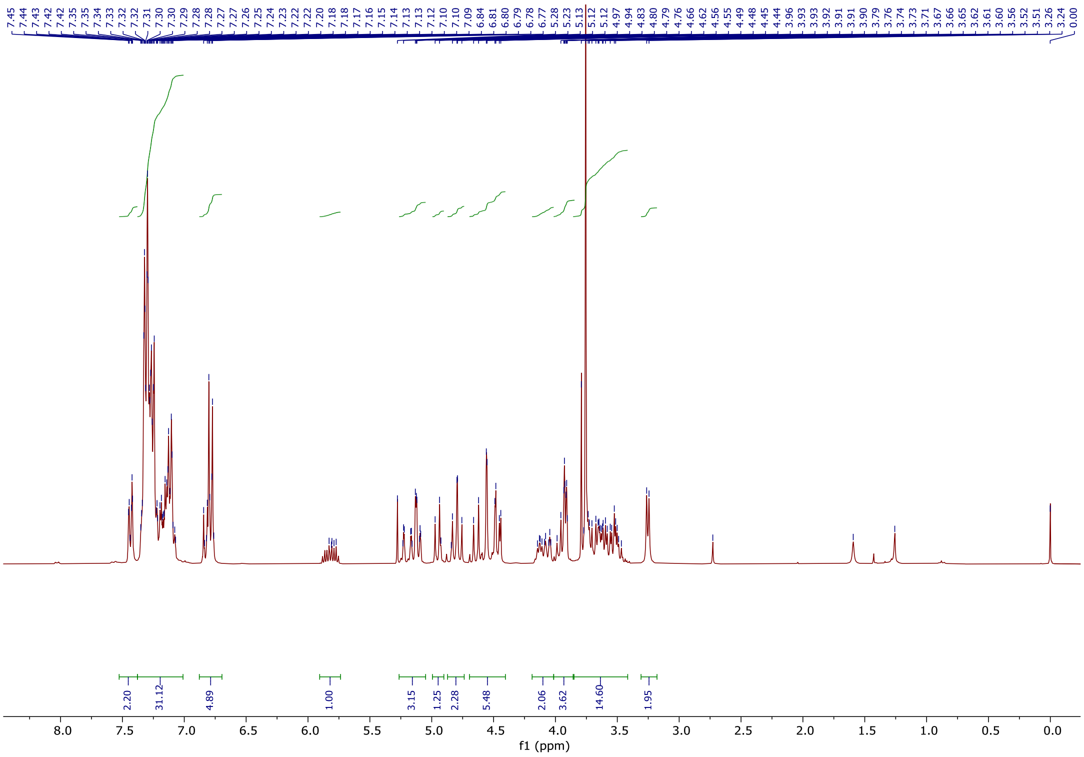

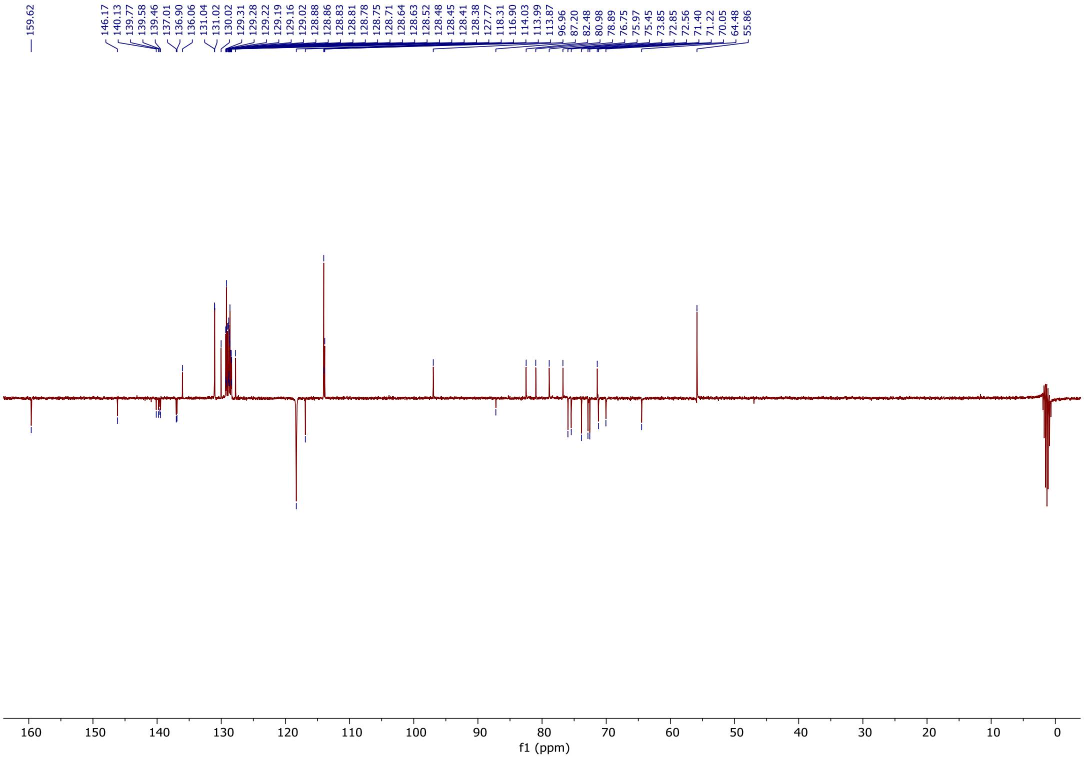

(R)-2-*O*-(2,3,4,6-*O*-benzyl-α-D-glucopyranosyl)-3-*O*(4,4'-dimethoxytrityl)-*sn*-glycerol (S5)

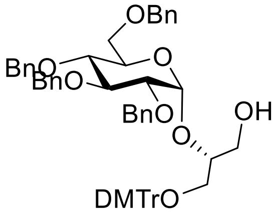

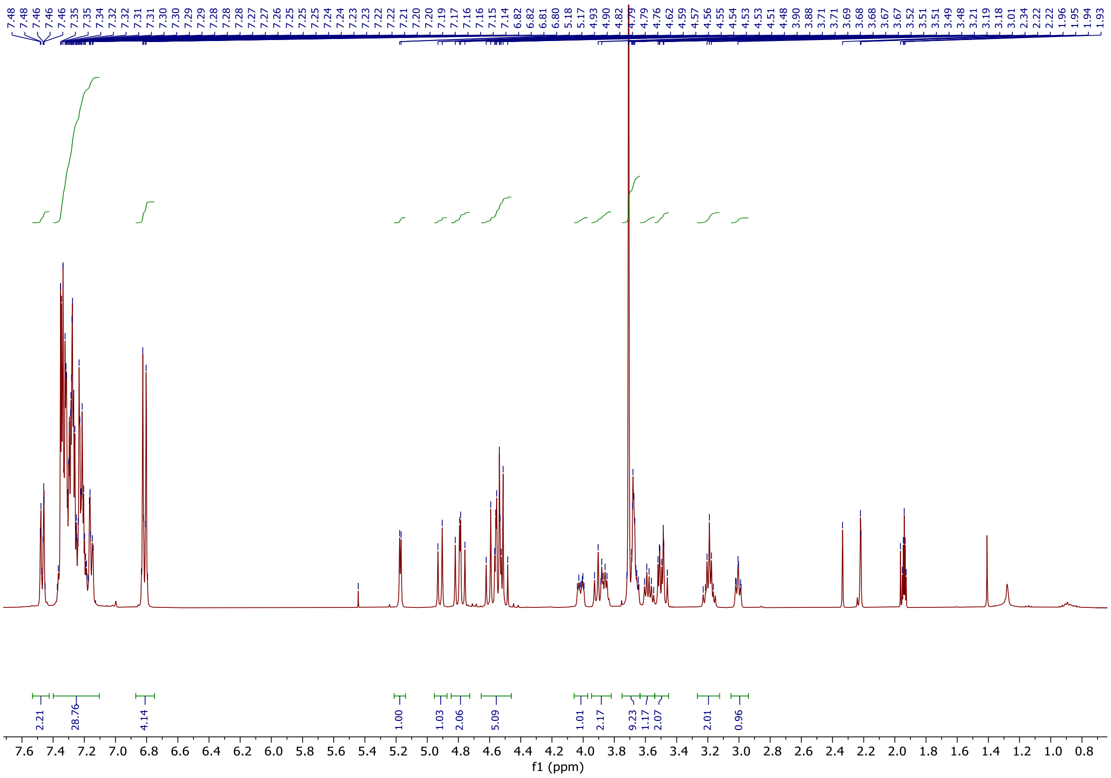

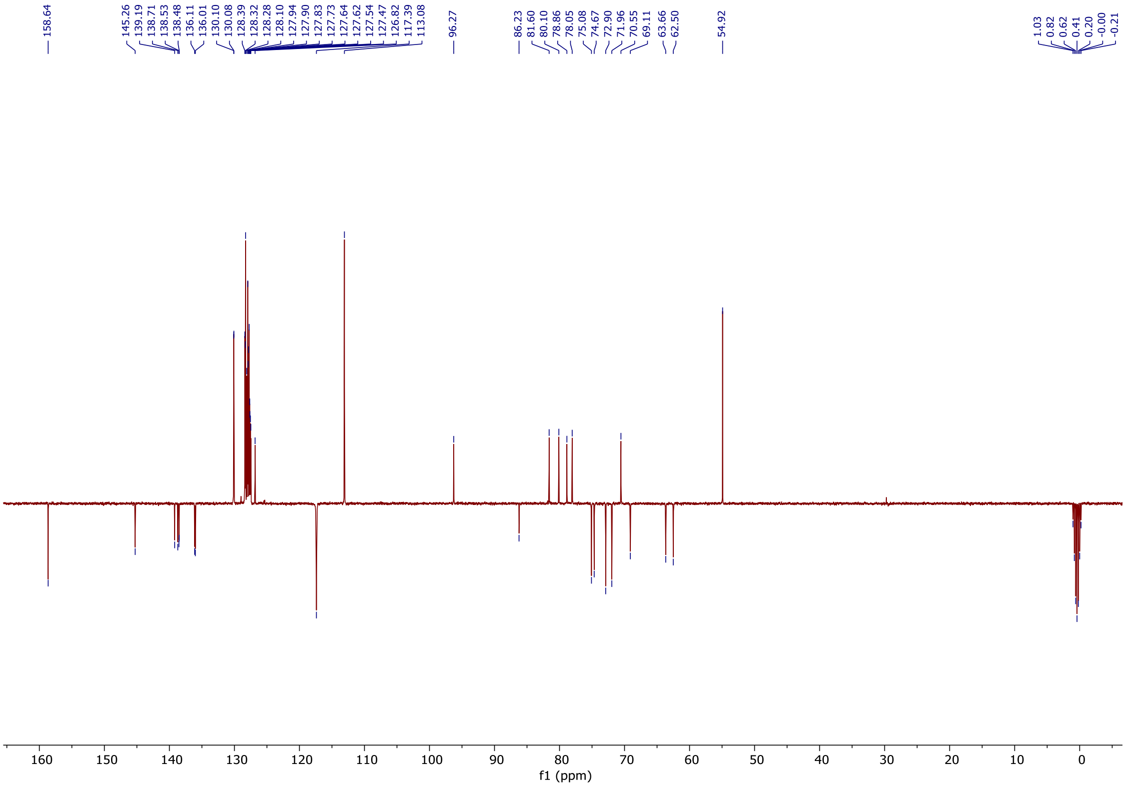

(GroP)-Spacer or Monomer S6

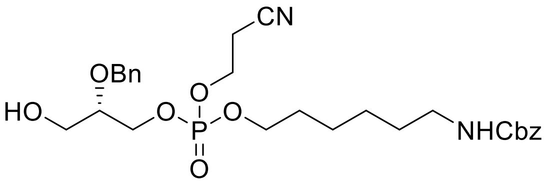

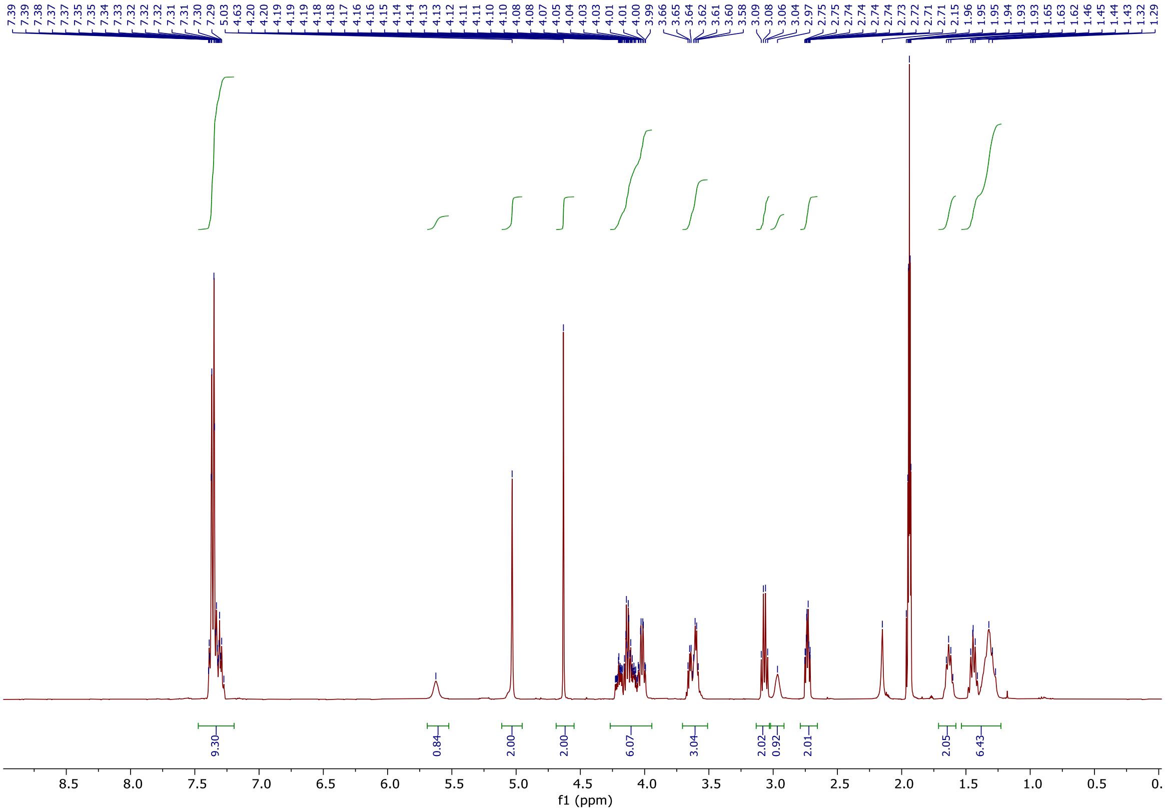

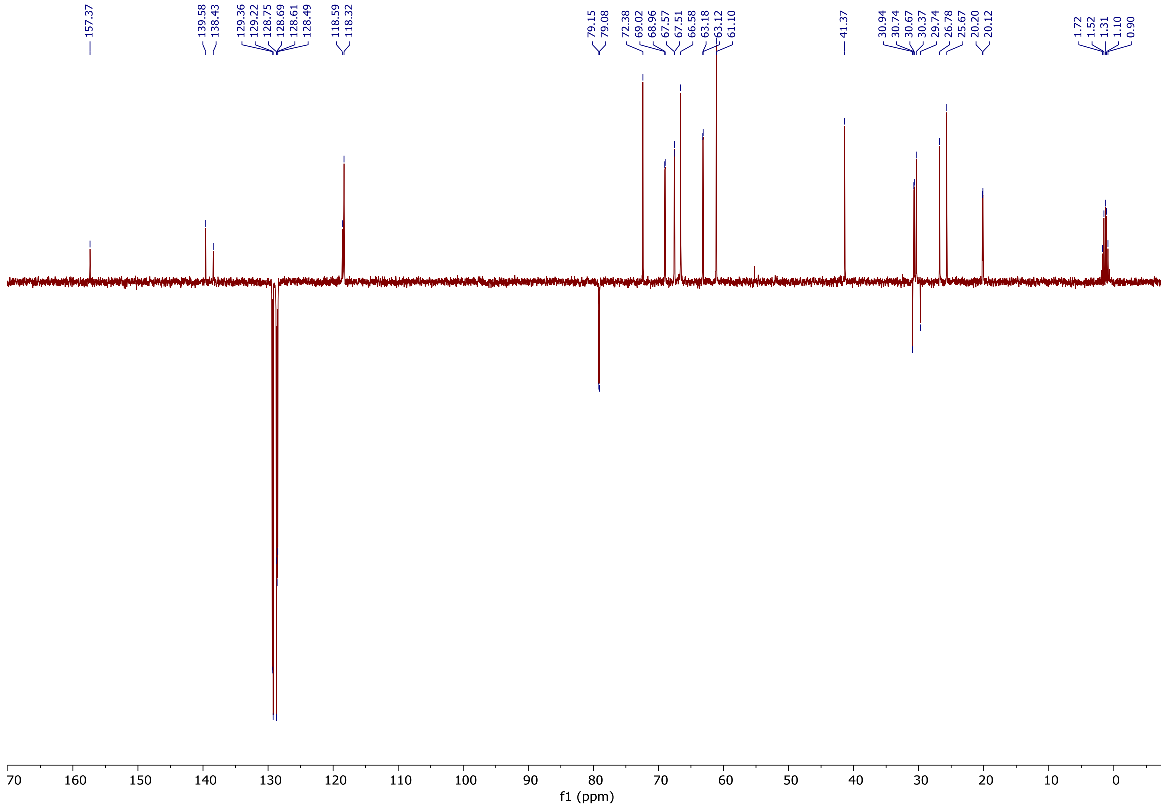
157.37
139.58
138.43
129.36
129.22
128.75
128.69
128.61
128.49
118.59
118.32
79.15
79.08
72.38
69.02
68.96
67.57
67.51
66.58
63.18
63.12
61.10
41.37
30.94
30.74
30.67
30.37
29.74
26.78
25.67
20.20
20.12
1.72
1.52
1.31
1.10
0.90
70 160 150 140 130 120 110 100 90 80 70 60 50 40 30 20 10 0
f1 (ppm)

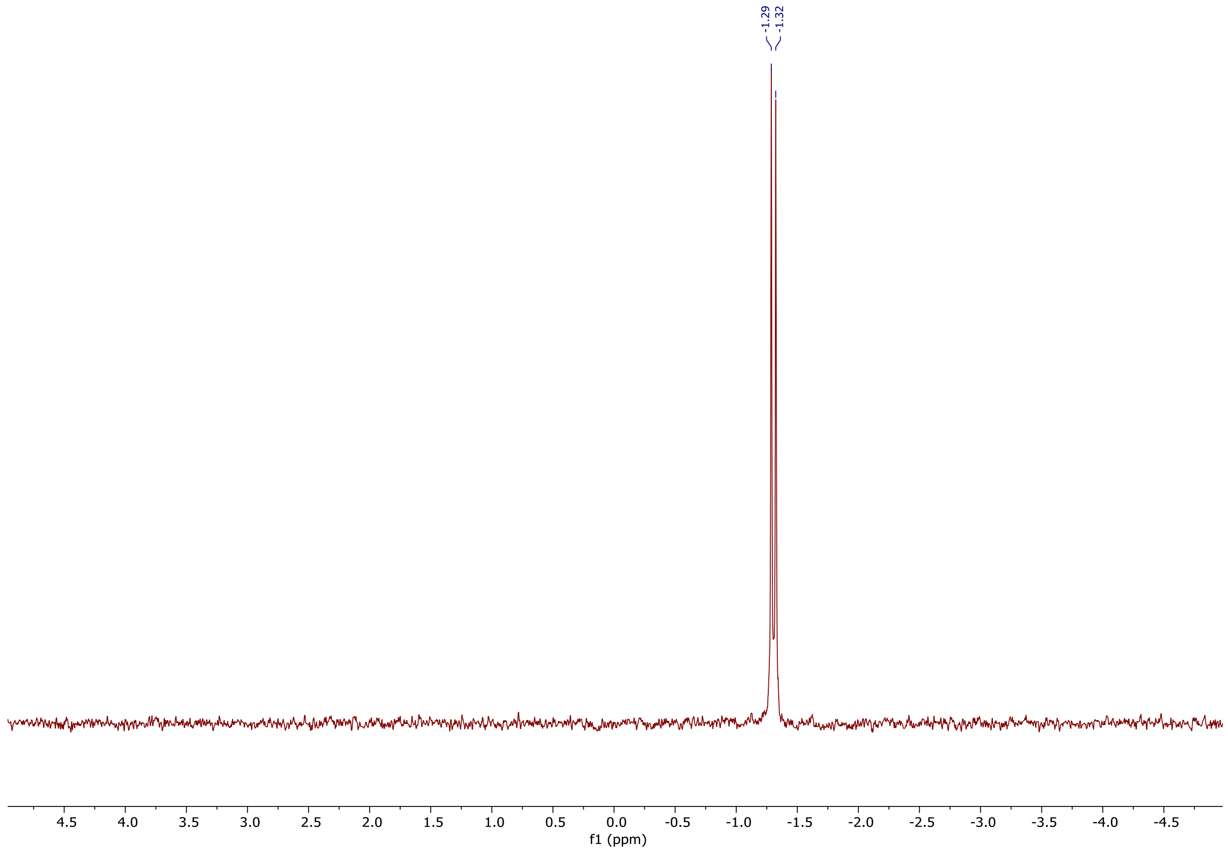
-1.29
-1.32
4.5 4.0 3.5 3.0 2.5 2.0 1.5 1.0 0.5 0.0 -0.5 -1.0 -1.5 -2.0 -2.5 -3.0 -3.5 -4.0 -4.5
f1 (ppm)

(GroP)$_2$-Spacer or Dimer S7

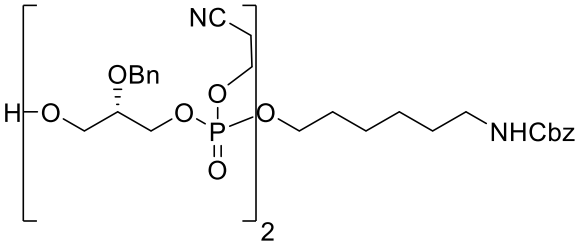

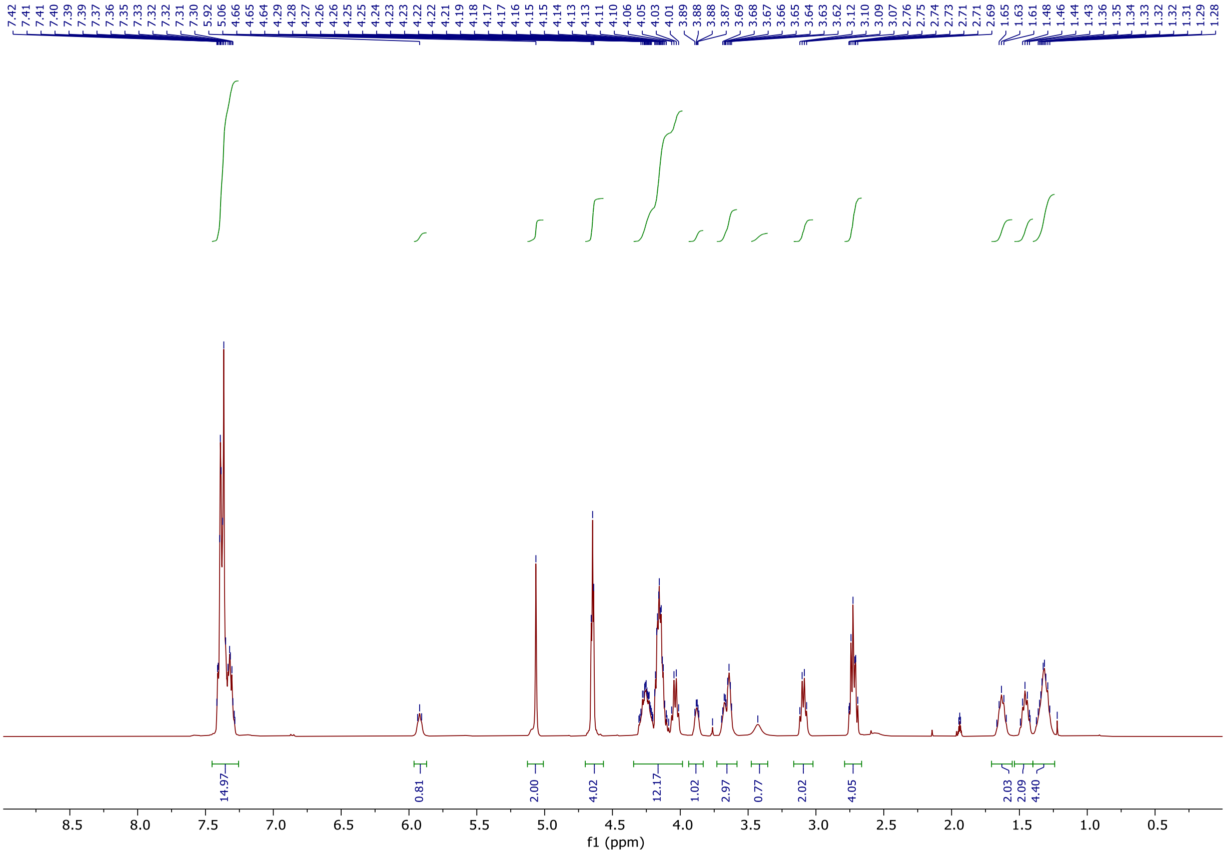

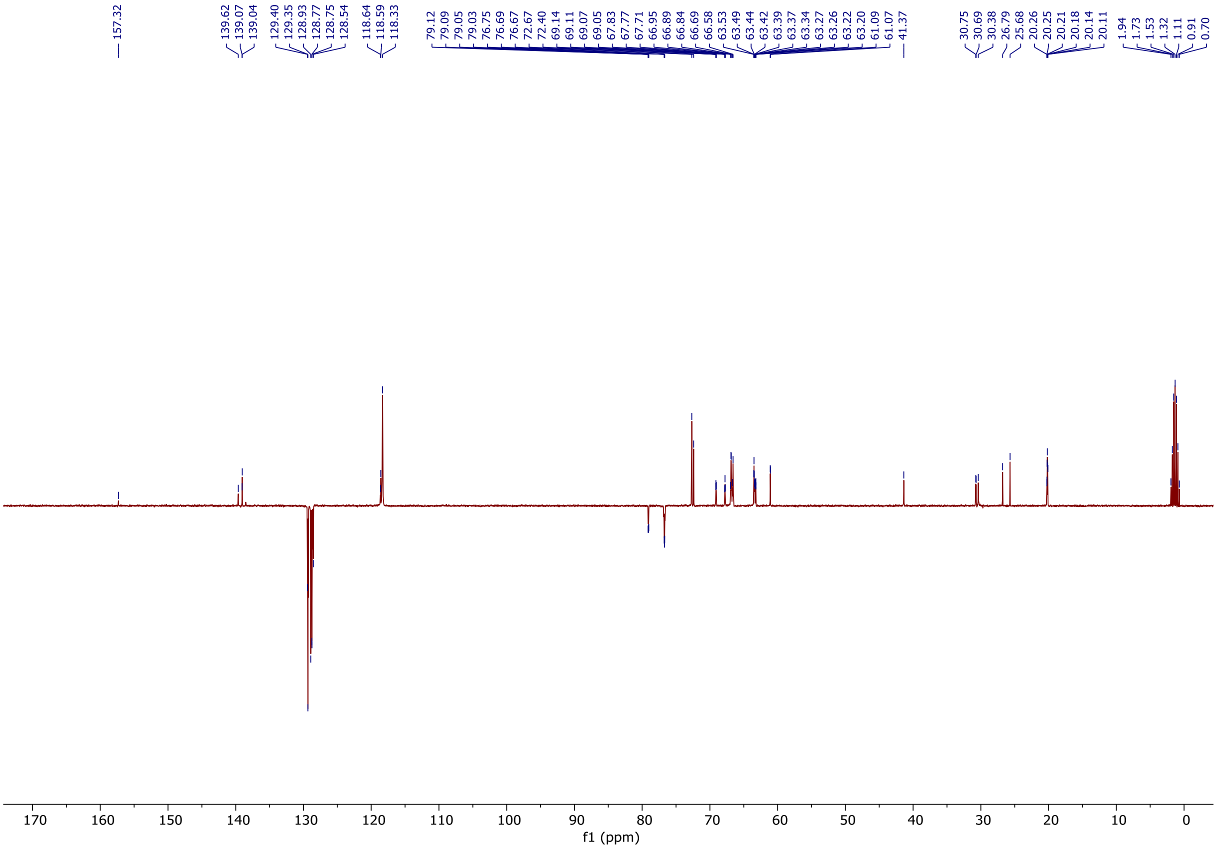
157.32
139.62
139.07
139.04
129.40
129.35
128.93
128.77
128.75
128.54
118.64
118.59
118.33
79.12
79.09
79.05
79.03
76.75
76.69
76.67
72.67
72.40
69.14
69.11
69.07
69.05
67.83
67.77
67.71
66.95
66.89
66.84
66.69
66.58
63.53
63.49
63.44
63.42
63.39
63.37
63.34
63.27
63.26
63.22
63.20
61.09
61.07
41.37
30.75
30.69
30.38
26.79
25.68
20.26
20.25
20.21
20.18
20.14
20.11
1.94
1.73
1.53
1.32
1.11
0.91
0.70
170 160 150 140 130 120 110 100 90 80 70 60 50 40 30 20 10 0
f1 (ppm)

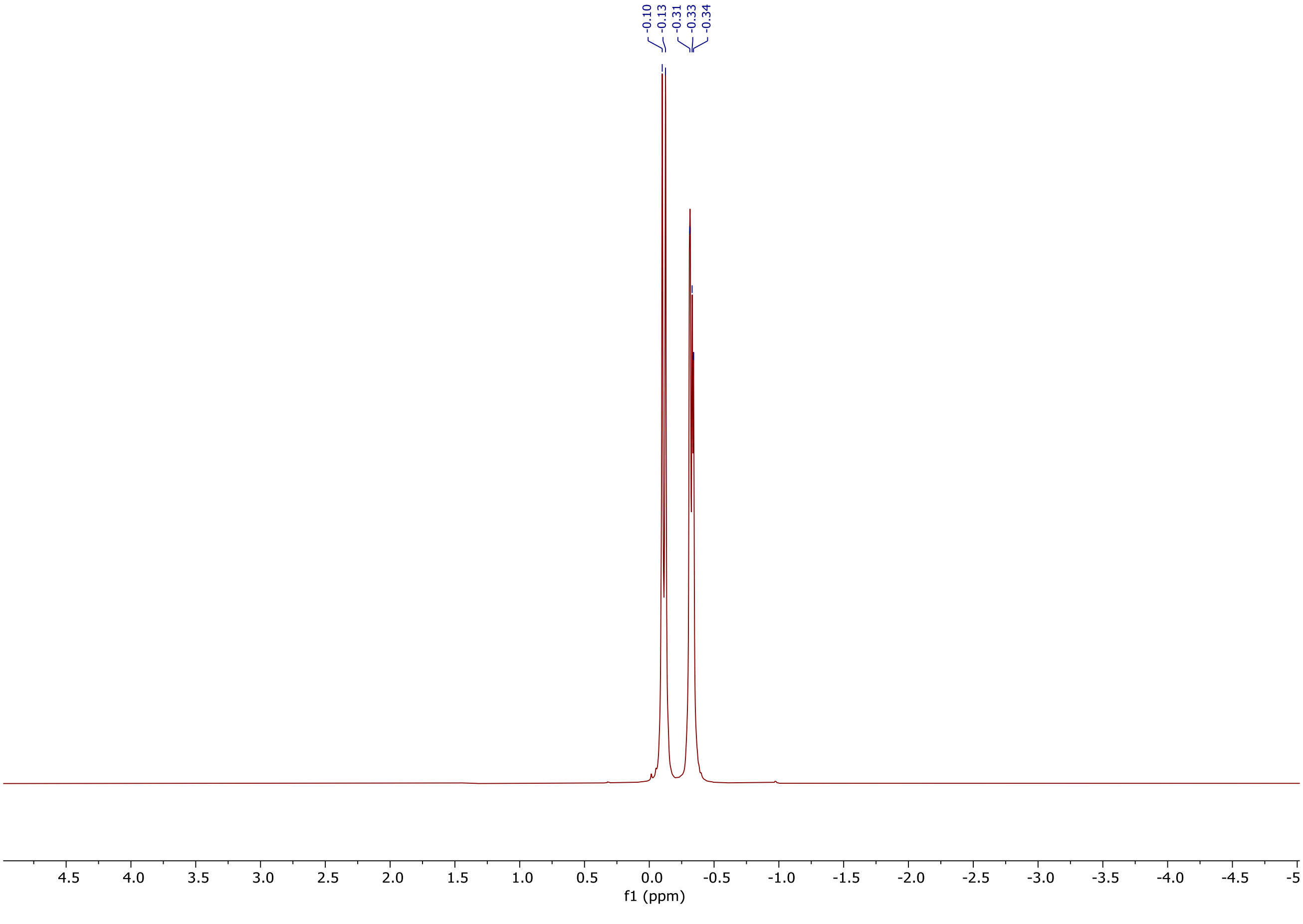
-0.10
-0.13
-0.31
-0.33
-0.34
4.5 4.0 3.5 3.0 2.5 2.0 1.5 1.0 0.5 0.0 -0.5 -1.0 -1.5 -2.0 -2.5 -3.0 -3.5 -4.0 -4.5 -5
f1 (ppm)

(GroP)$_3$-Spacer or Trimer S8

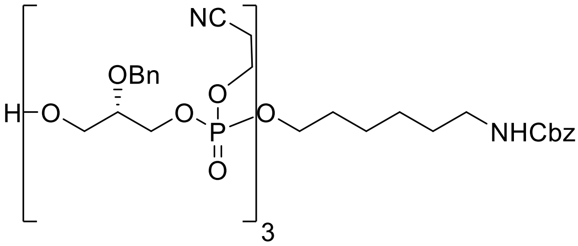

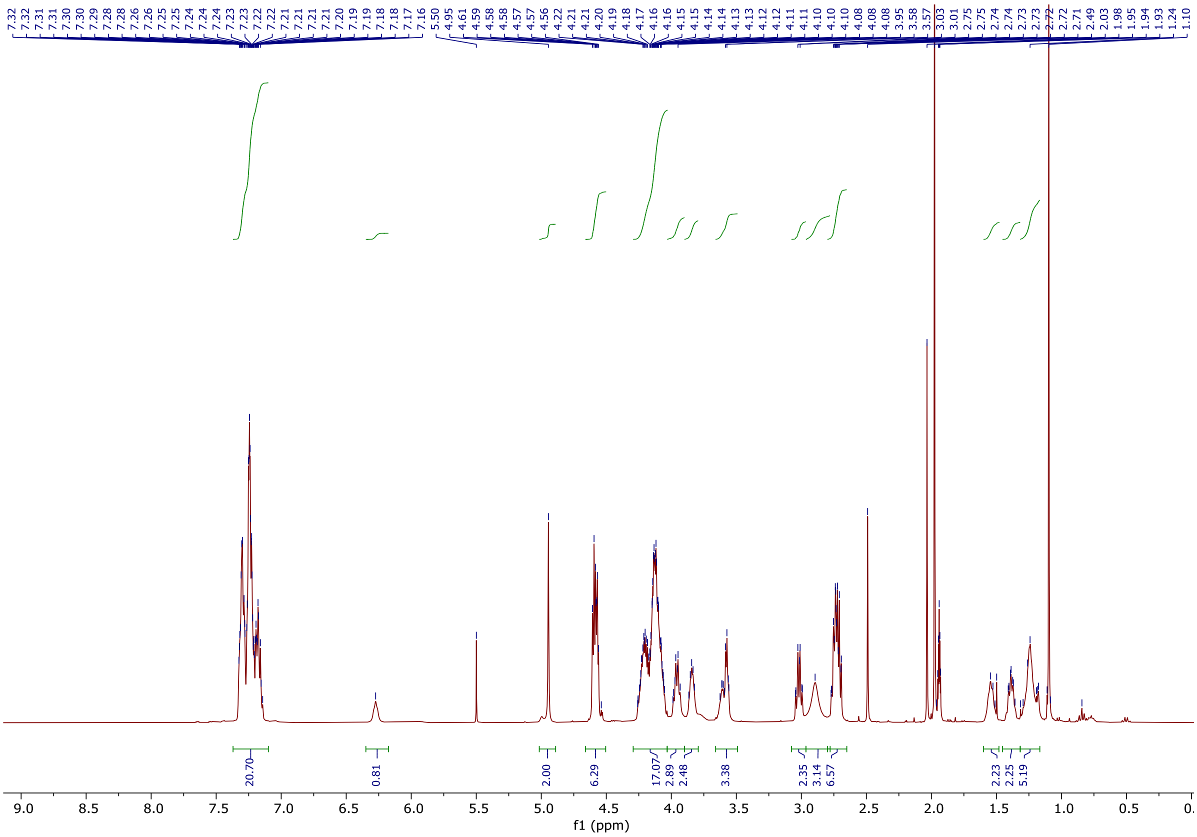

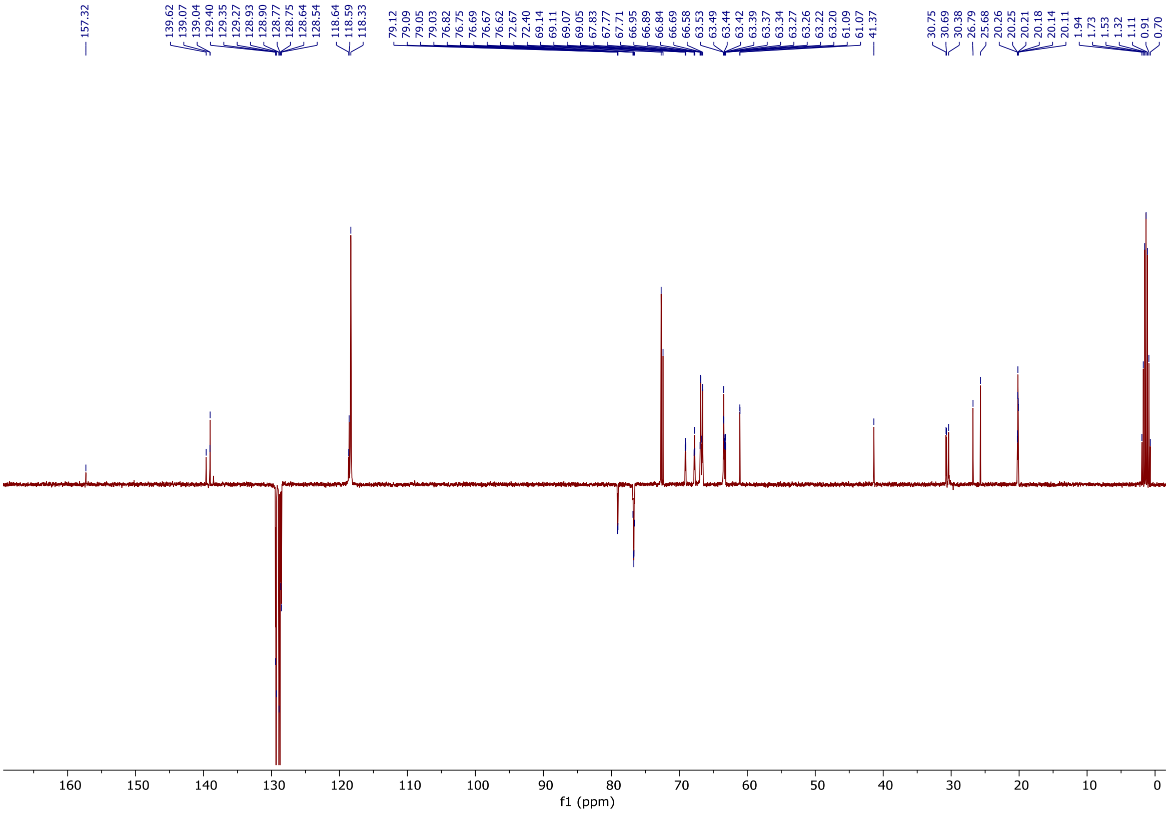
157.32
139.62
139.07
139.04
129.40
129.35
129.27
128.93
128.90
128.77
128.75
128.64
128.54
118.64
118.59
118.33
79.12
79.09
79.05
79.03
76.82
76.75
76.69
76.67
76.62
72.67
72.40
69.14
69.11
69.07
69.05
67.83
67.77
67.71
66.95
66.89
66.84
66.69
66.58
63.53
63.49
63.44
63.42
63.39
63.37
63.34
63.27
63.26
63.22
63.20
61.09
61.07
41.37
30.75
30.69
30.38
26.79
25.68
20.26
20.25
20.21
20.18
20.14
20.11
1.94
1.73
1.53
1.32
1.11
0.91
0.70
160 150 140 130 120 110 100 90 80 70 60 50 40 30 20 10 0
f1 (ppm)

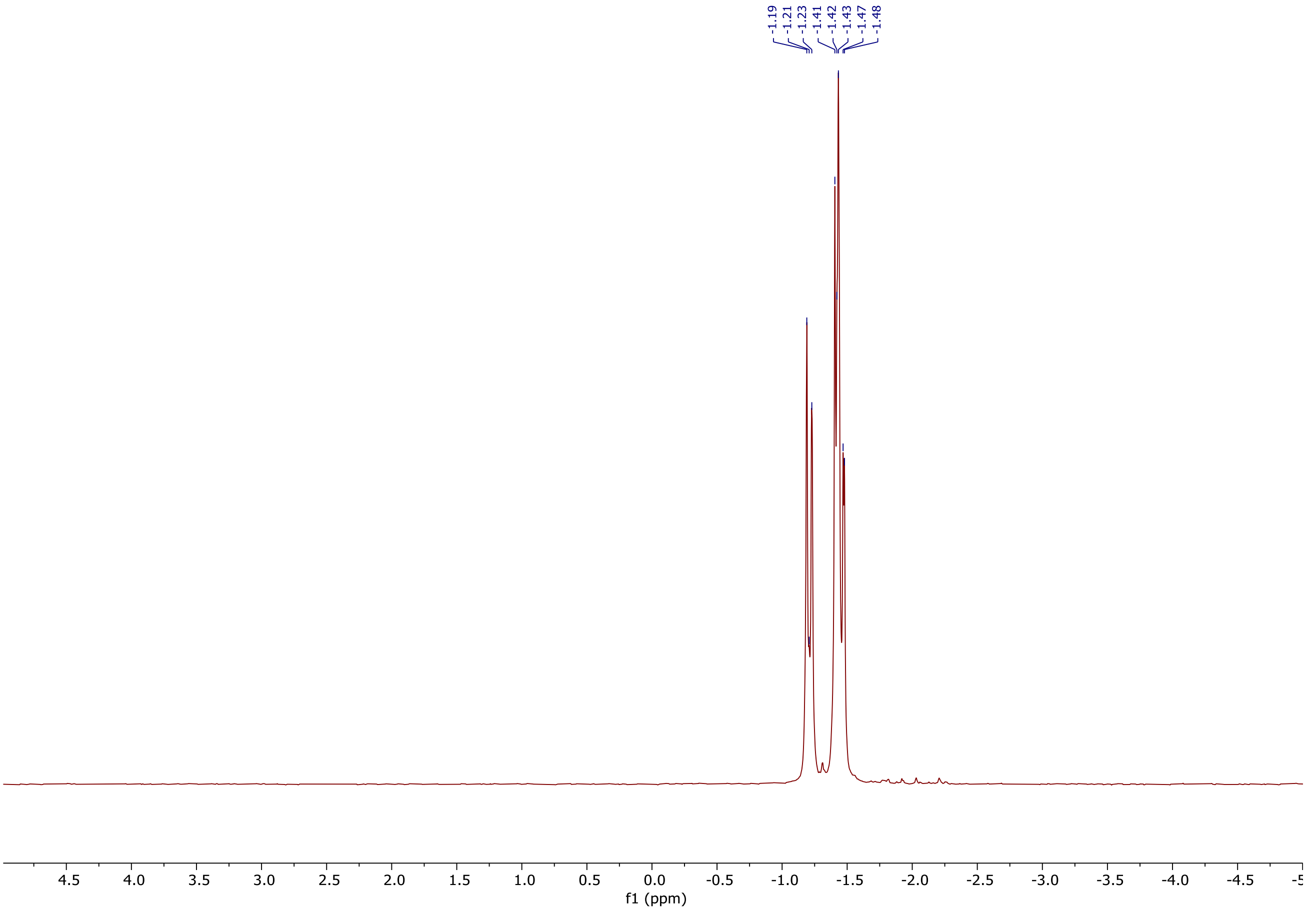
-1.19
-1.21
-1.23
-1.41
-1.42
-1.43
-1.47
-1.48
4.5 4.0 3.5 3.0 2.5 2.0 1.5 1.0 0.5 0.0 -0.5 -1.0 -1.5 -2.0 -2.5 -3.0 -3.5 -4.0 -4.5
f1 (ppm)

(GroP)$_4$-Spacer or Tetramer S9

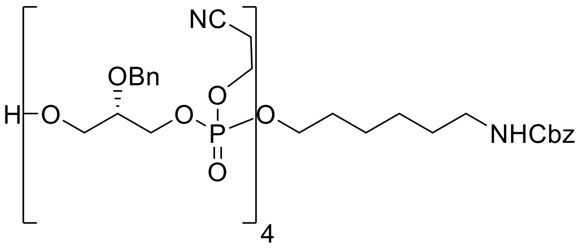

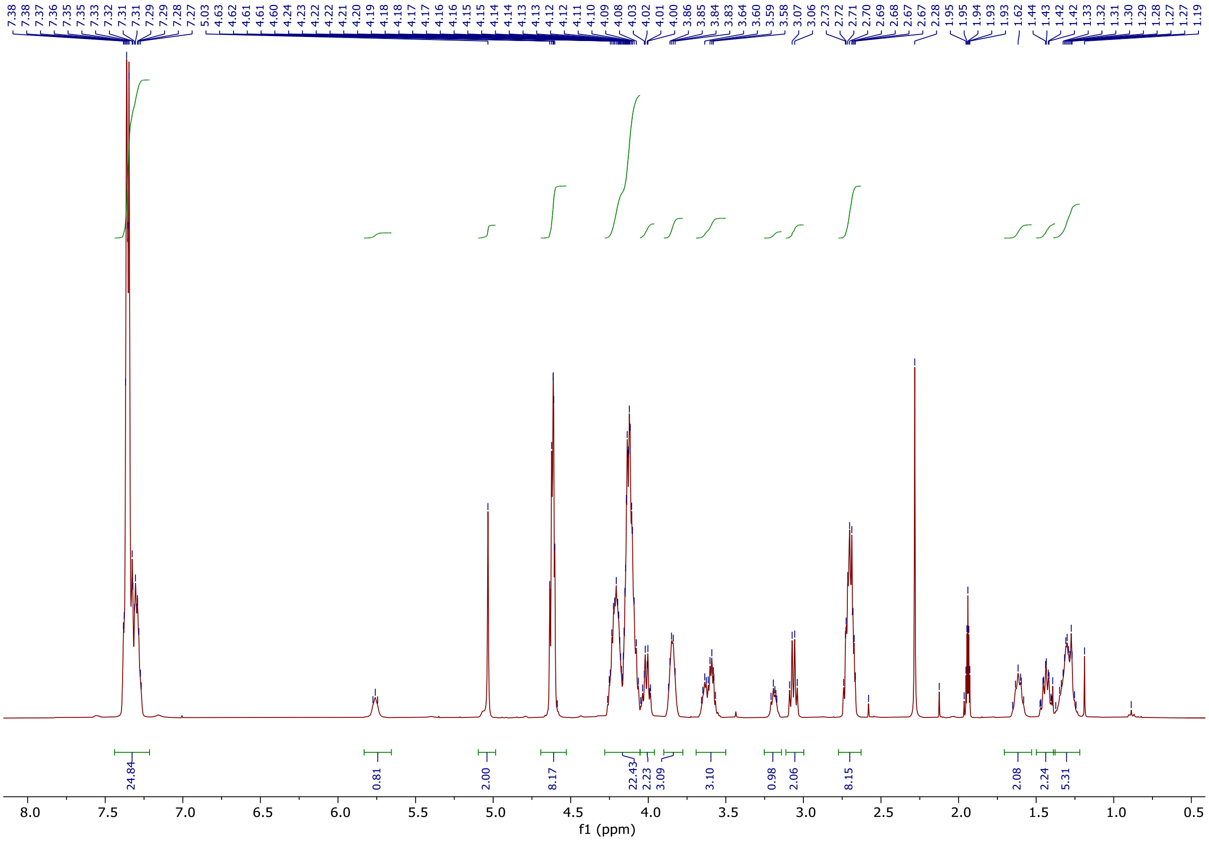

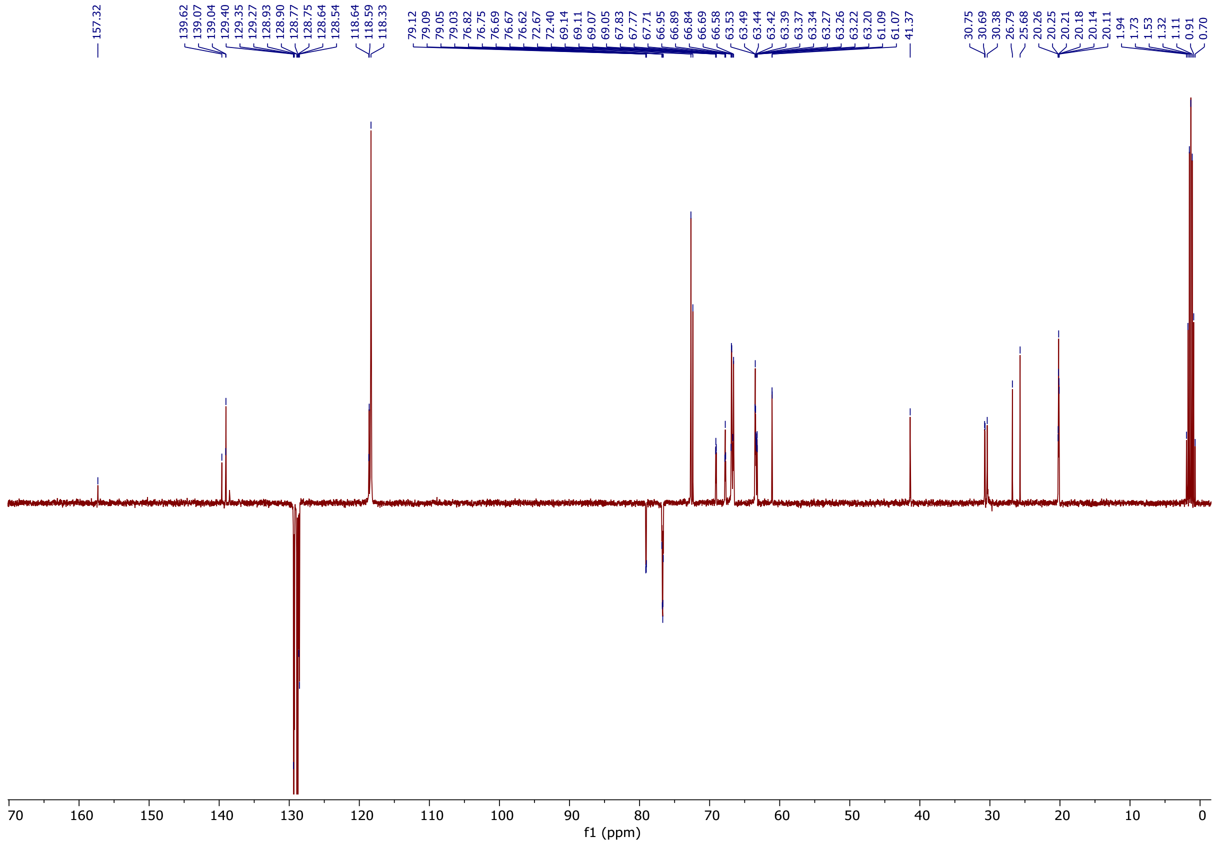
157.32
139.62
139.07
139.04
129.40
129.35
129.27
128.93
128.90
128.77
128.75
128.64
128.54
118.64
118.59
118.33
79.12
79.09
79.05
79.03
76.82
76.75
76.69
76.67
76.62
72.67
72.40
69.14
69.11
69.07
69.05
67.83
67.77
67.71
66.95
66.89
66.84
66.69
66.58
63.53
63.49
63.44
63.42
63.39
63.37
63.34
63.27
63.26
63.22
63.20
61.09
61.07
41.37
30.75
30.69
30.38
26.79
25.68
20.26
20.25
20.21
20.18
20.14
20.11
1.94
1.73
1.53
1.32
1.11
0.91
0.70
f1 (ppm)

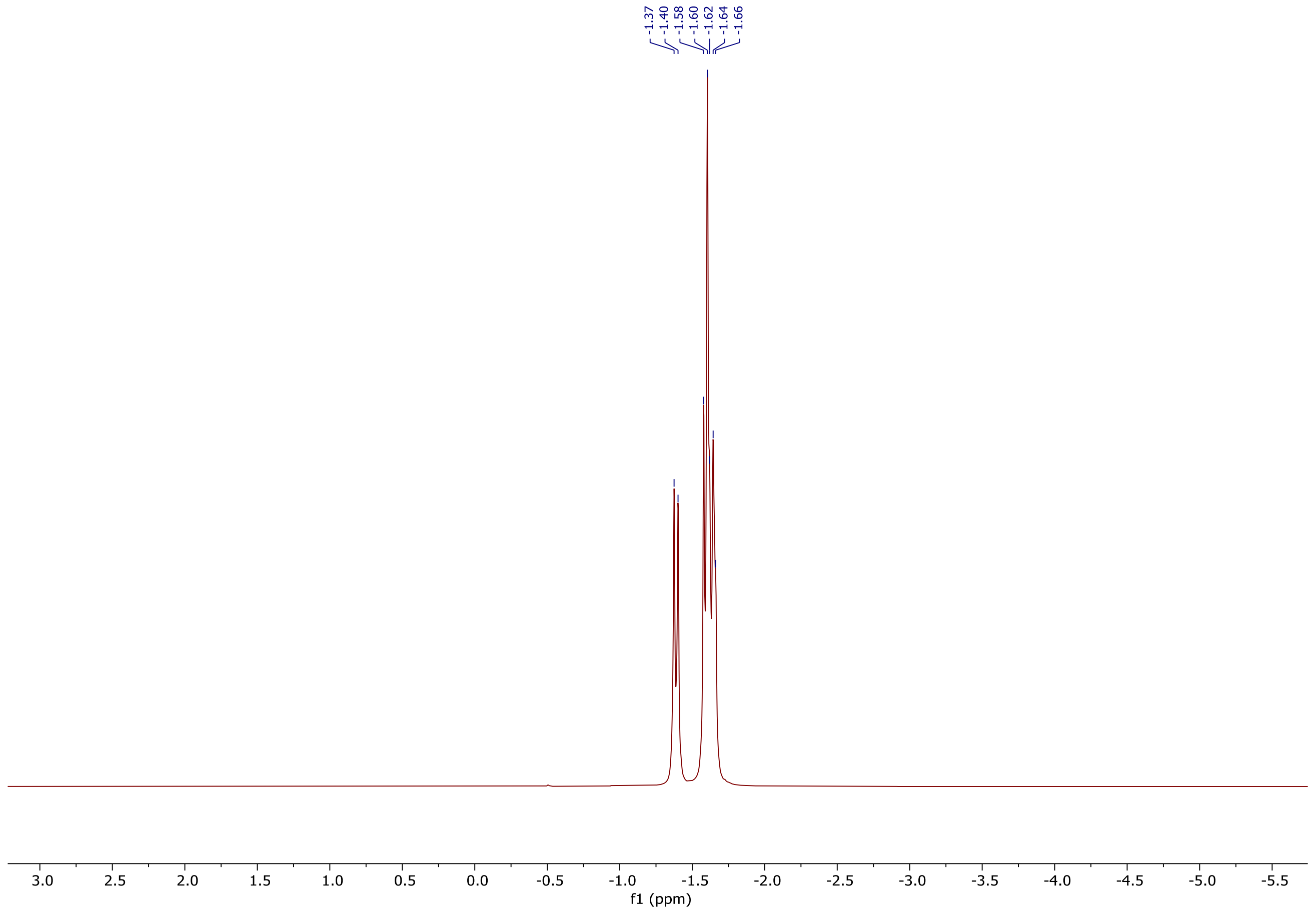
-1.37
-1.40
-1.58
-1.60
-1.62
-1.64
-1.66
f1 (ppm)

(Protected) (GroP)$_5$-Spacer or Pentamer S10

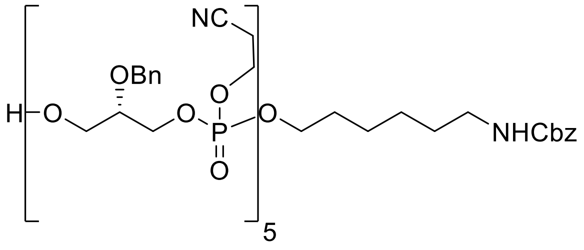

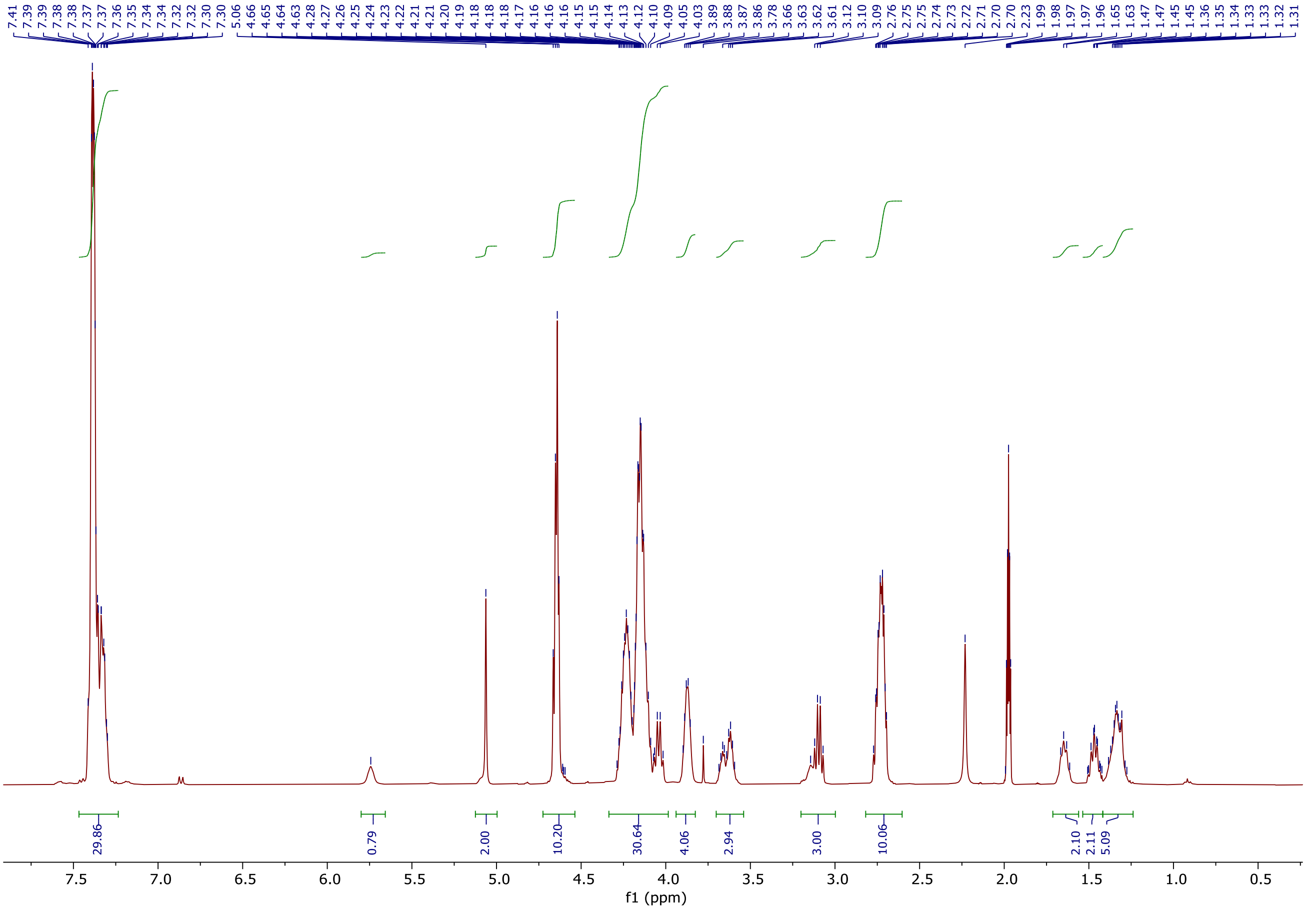

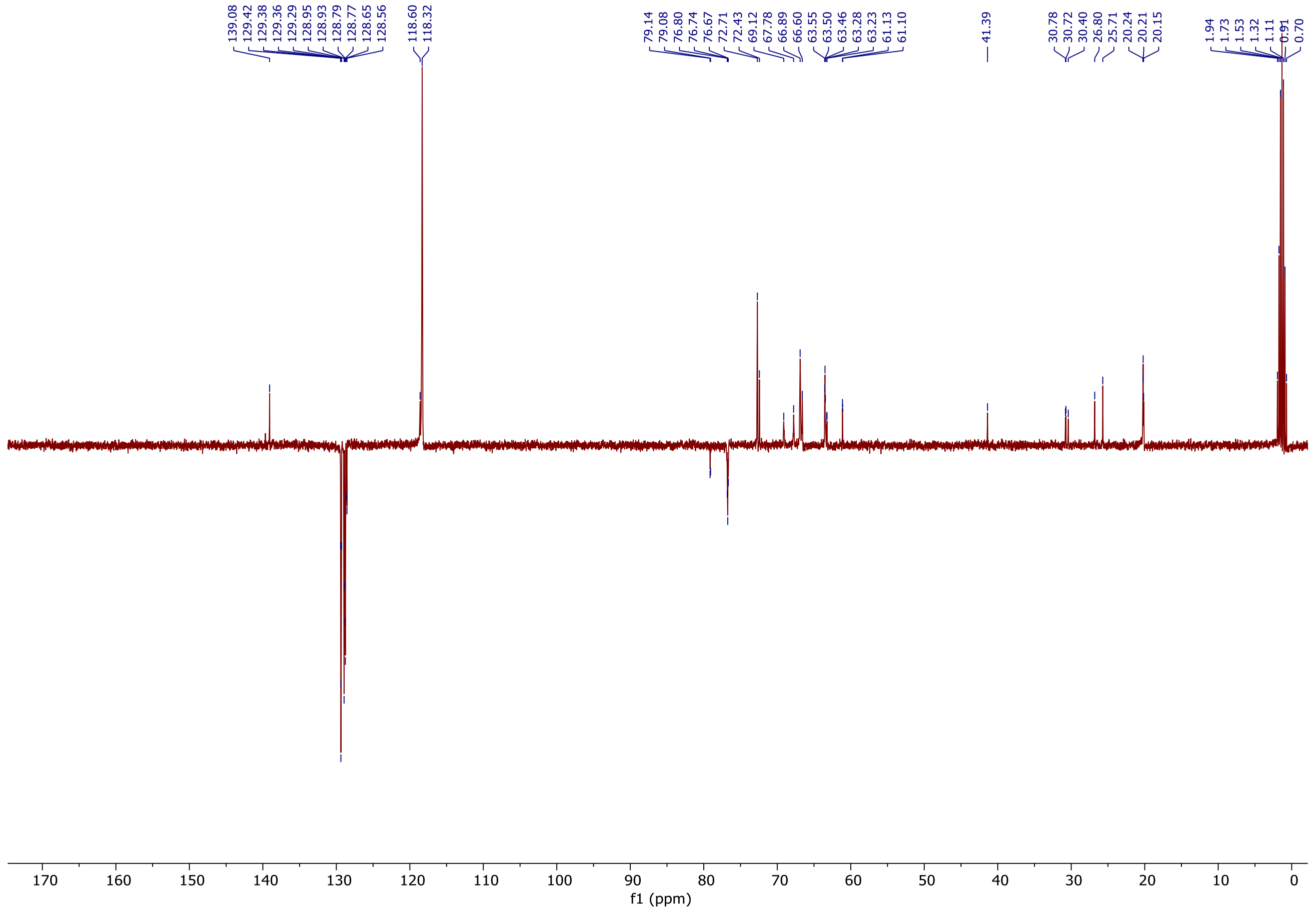
139.08
129.42
129.38
129.36
129.29
128.95
128.93
128.79
128.77
128.65
128.56
118.60
118.32
79.14
79.08
76.80
76.74
76.67
72.71
72.43
69.12
67.78
66.89
66.60
63.55
63.50
63.46
63.28
63.23
61.13
61.10
41.39
30.78
30.72
30.40
26.80
25.71
20.24
20.21
20.15
1.94
1.73
1.53
1.32
1.11
0.91
0.70
f1 (ppm)

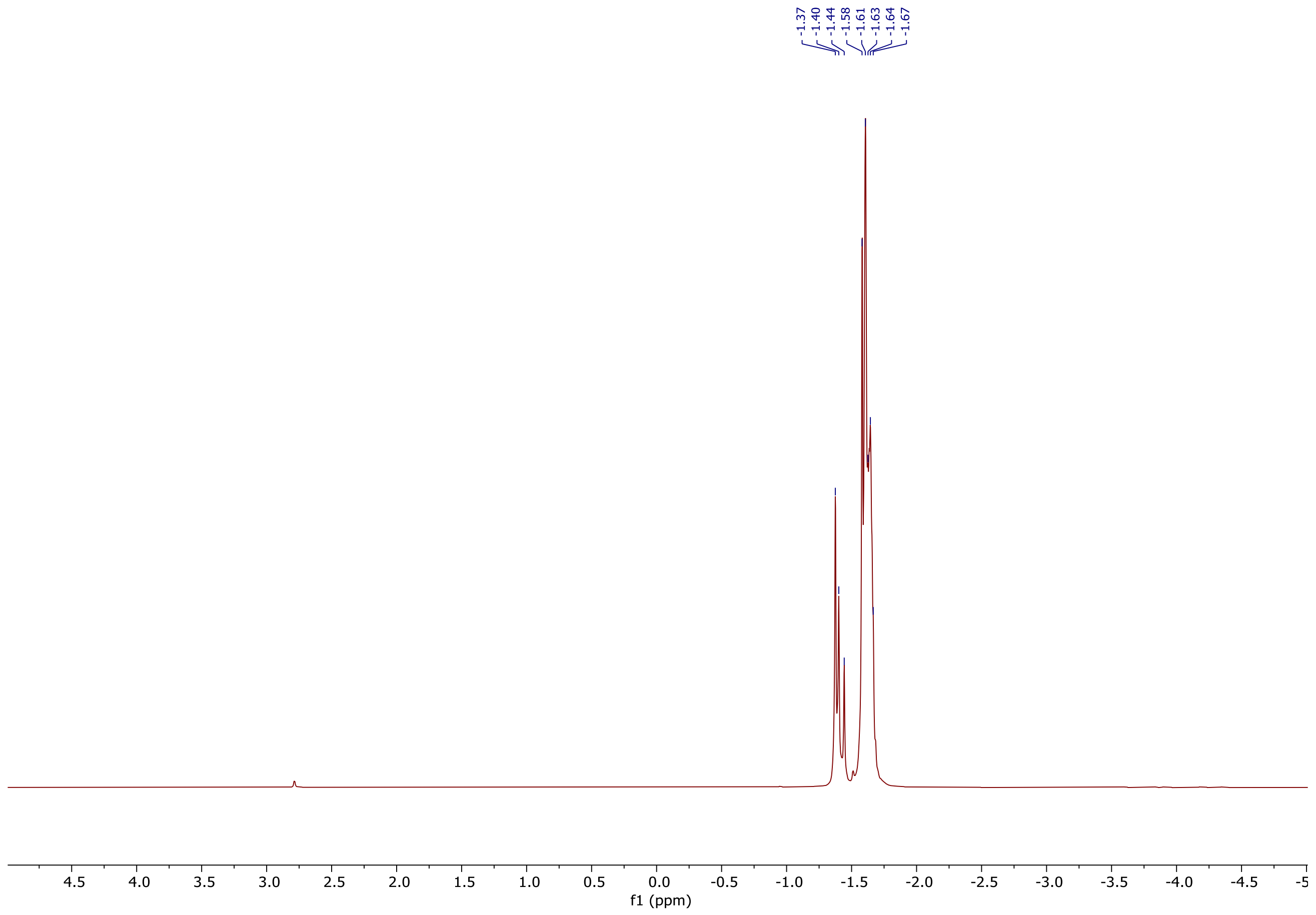
-1.37
-1.40
-1.44
-1.58
-1.61
-1.63
-1.64
-1.67
f1 (ppm)

(Protected) (GlcGroP)(GroP)$_5$-Spacer or Hexamer23

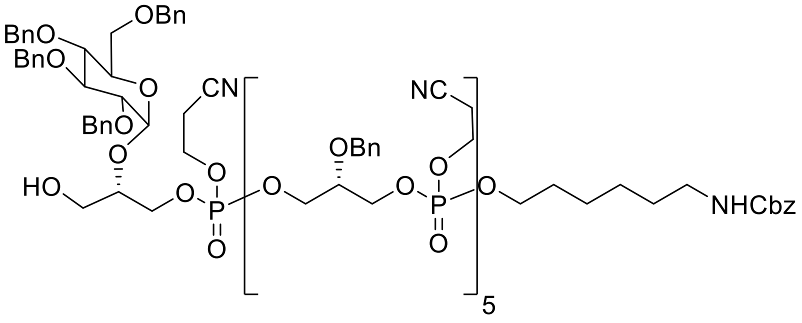

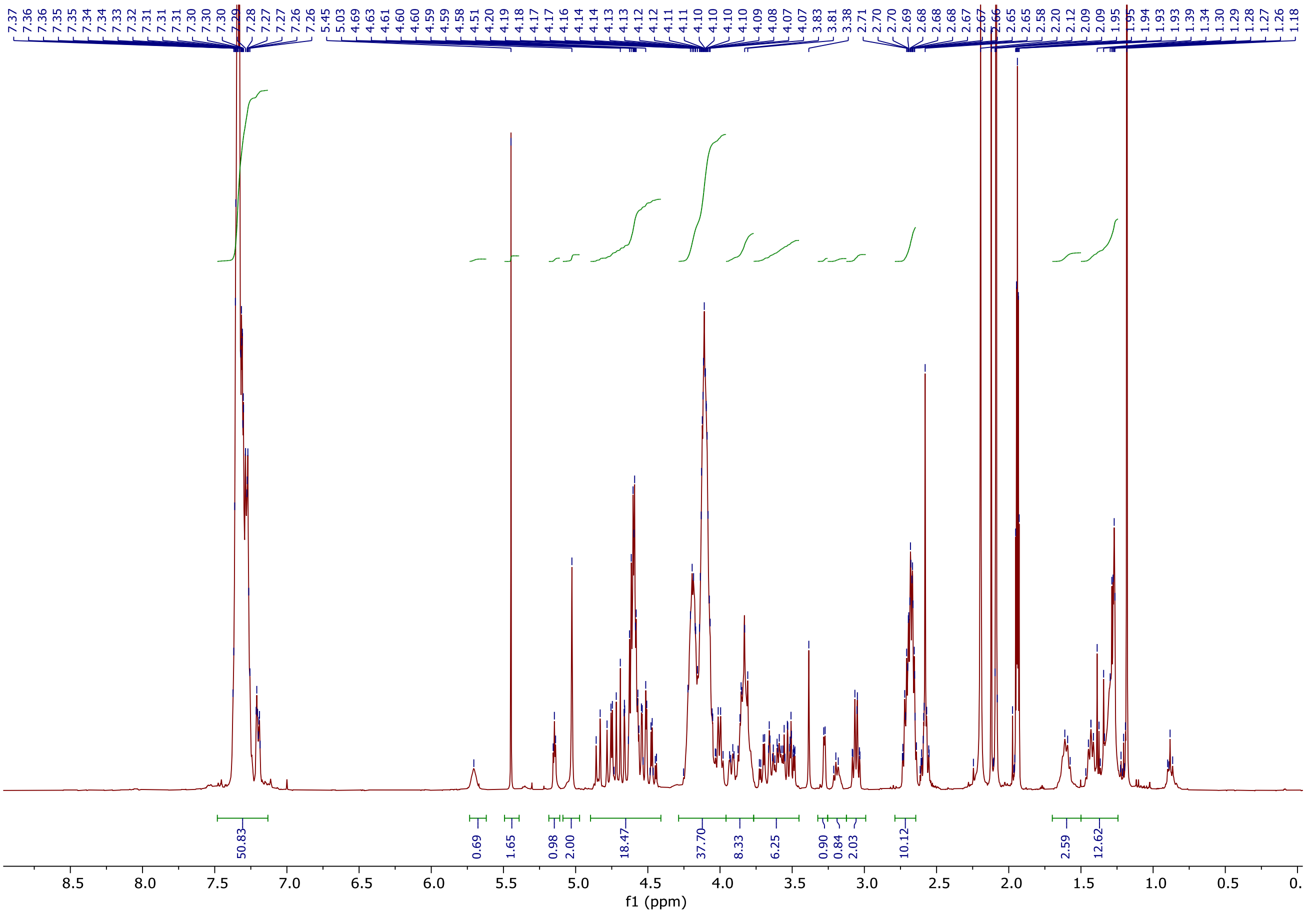

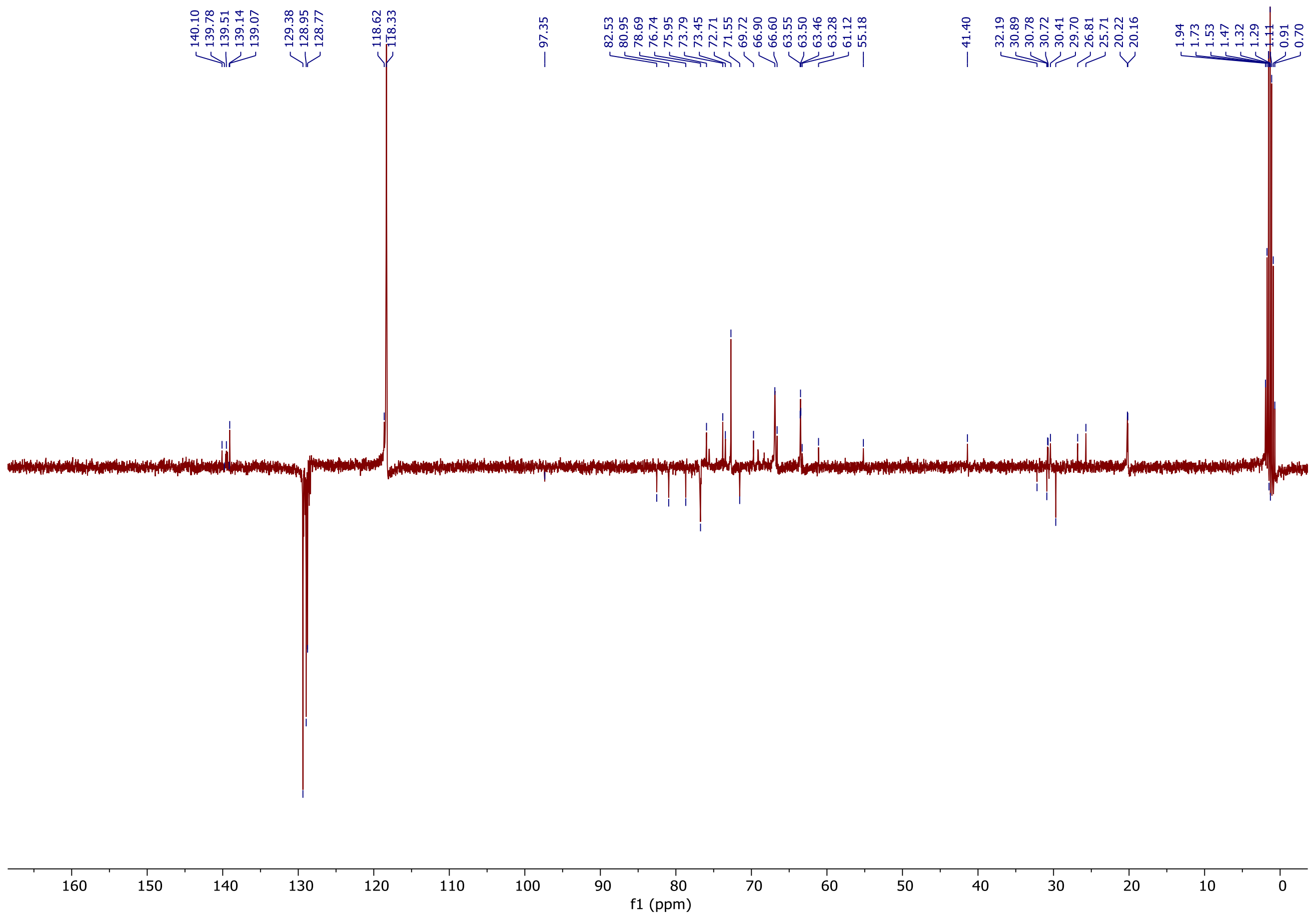
140.10
139.78
139.51
139.14
139.07
129.38
128.95
128.77
118.62
118.33
97.35
82.53
80.95
78.69
76.74
75.95
73.79
73.45
72.71
71.55
69.72
66.90
66.60
63.55
63.50
63.46
63.28
61.12
55.18
41.40
32.19
30.89
30.78
30.72
30.41
29.70
26.81
25.71
20.22
20.16
1.94
1.73
1.53
1.47
1.32
1.29
1.11
0.91
0.70
f1 (ppm)

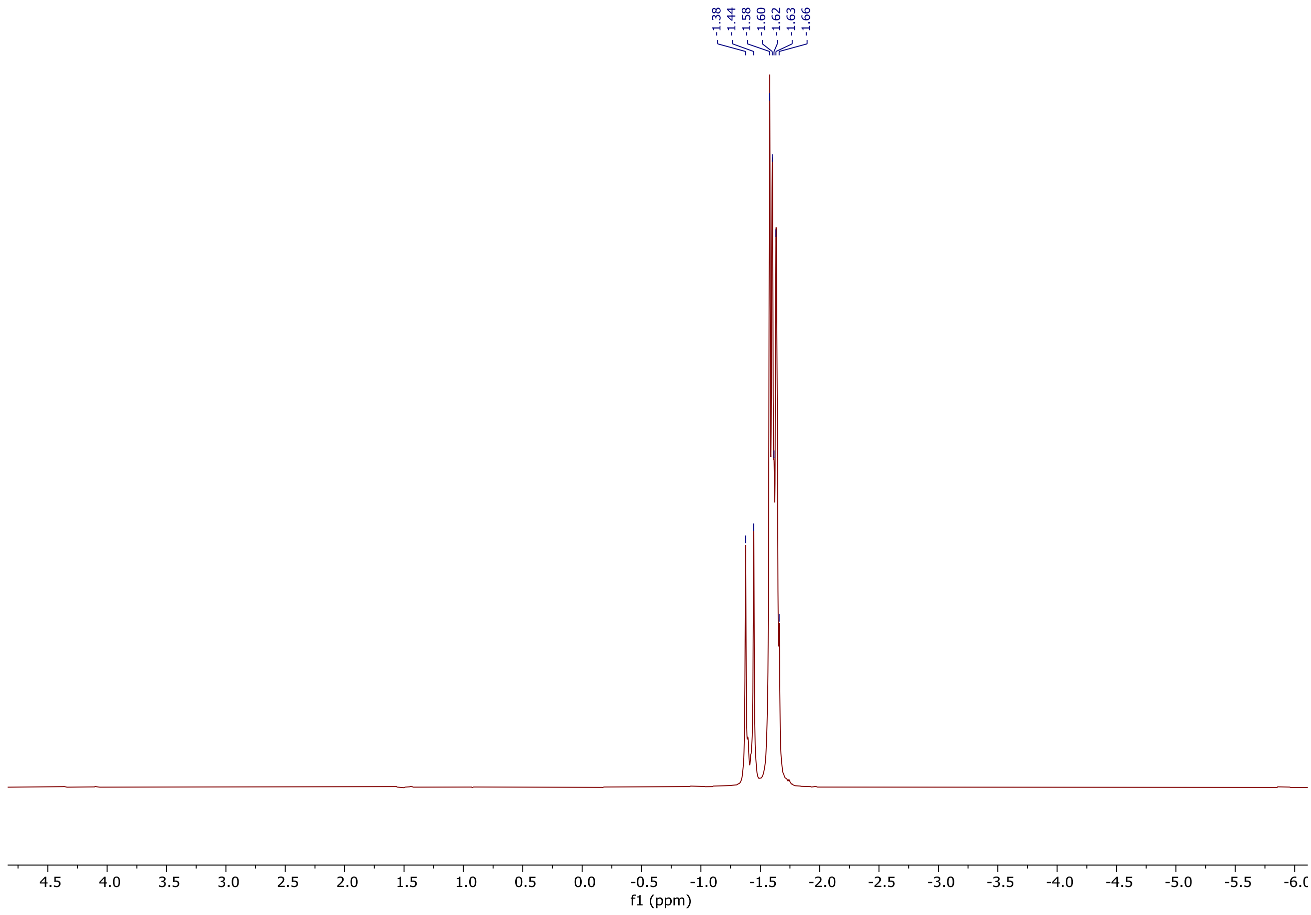
-1.38
-1.44
-1.58
-1.60
-1.62
-1.63
-1.66
f1 (ppm)

(Protected) (GlcGroP)(GroP)$_4$-Spacer or Pentamer S11

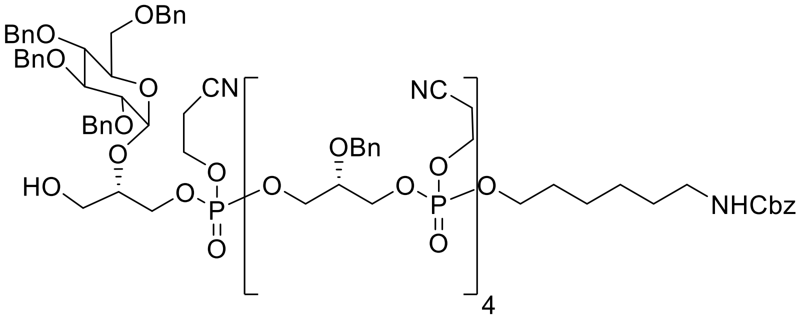

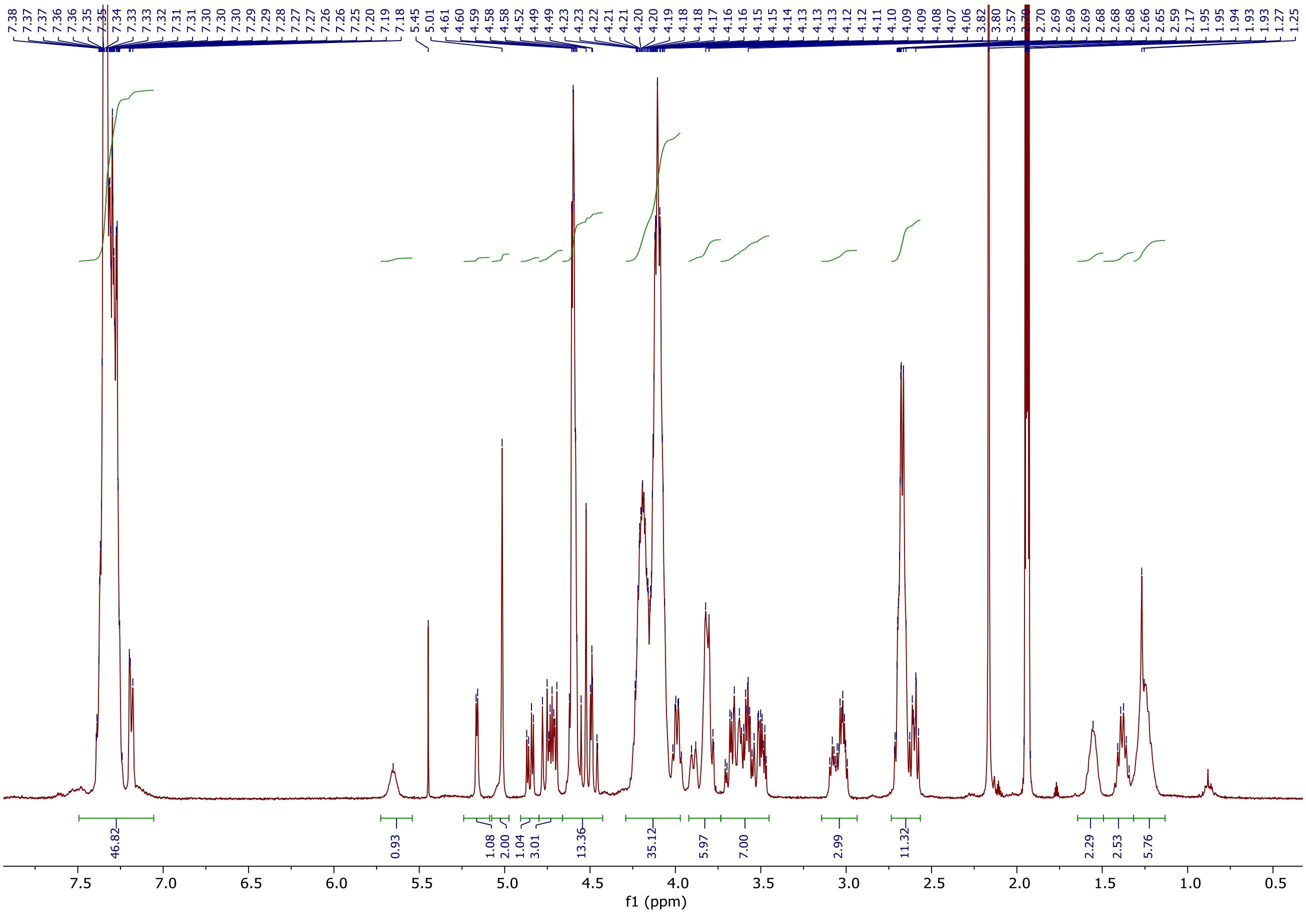

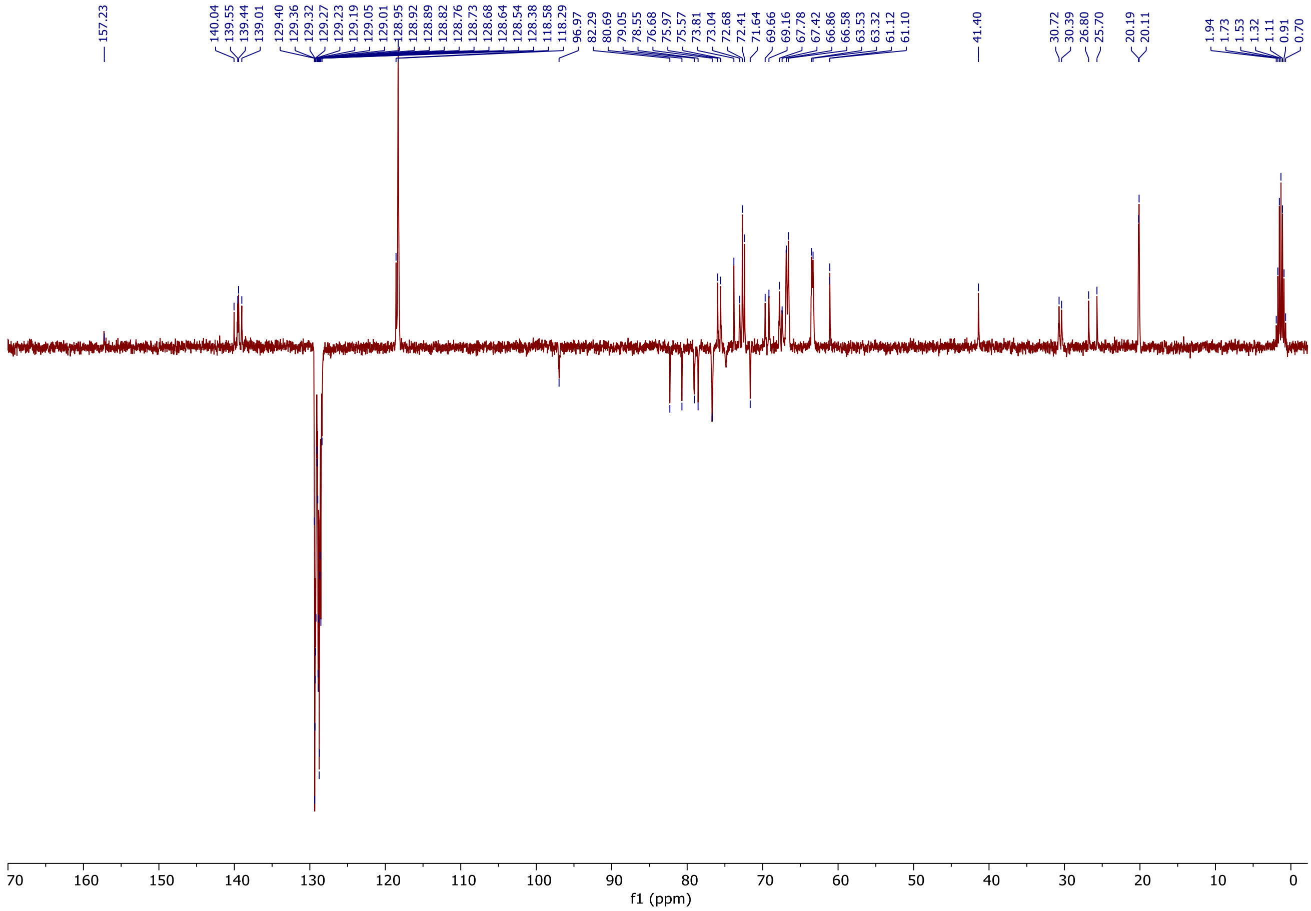
157.23
140.04
139.55
139.44
139.01
129.40
129.36
129.32
129.27
129.23
129.19
129.05
129.01
128.95
128.92
128.89
128.82
128.76
128.73
128.68
128.64
128.54
128.38
118.58
118.29
96.97
82.29
80.69
79.05
78.55
76.68
75.97
75.57
73.81
73.04
72.68
72.41
71.64
69.66
69.16
67.78
67.42
66.86
66.58
63.53
63.32
61.12
61.10
41.40
30.72
30.39
26.80
25.70
20.19
20.11
1.94
1.73
1.53
1.32
1.11
0.91
0.70
f1 (ppm)

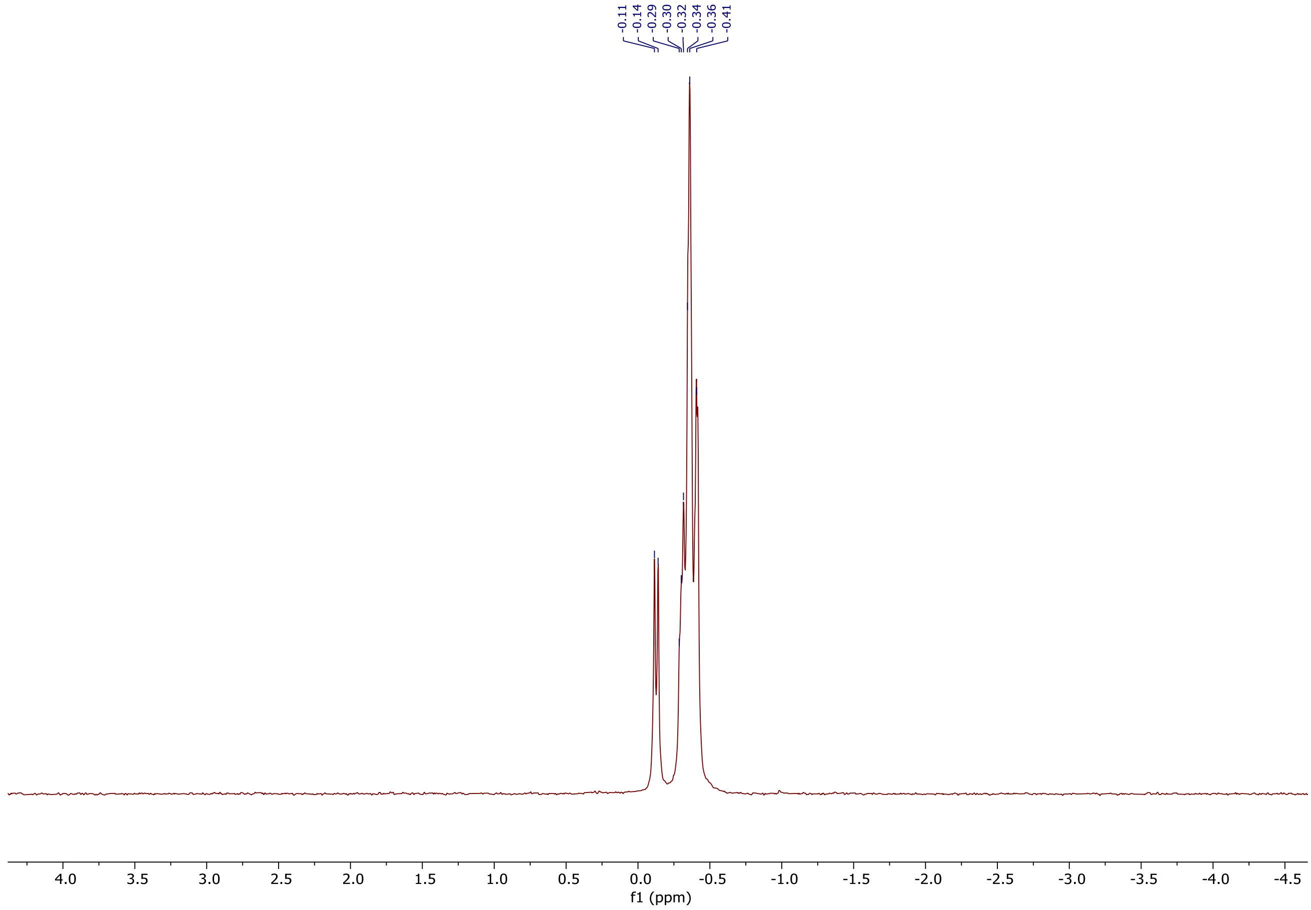
-0.11
-0.14
-0.29
-0.30
-0.32
-0.34
-0.36
-0.41
f1 (ppm)

(Protected) (GroP)(GlcGroP)(GroP)$_4$-Spacer or Hexamer 24

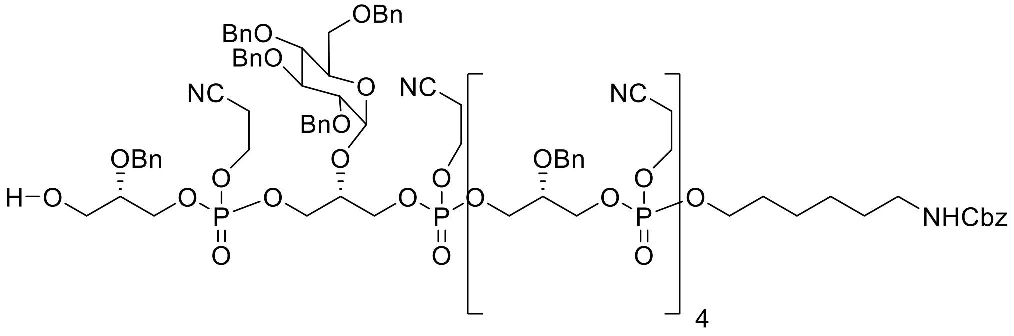

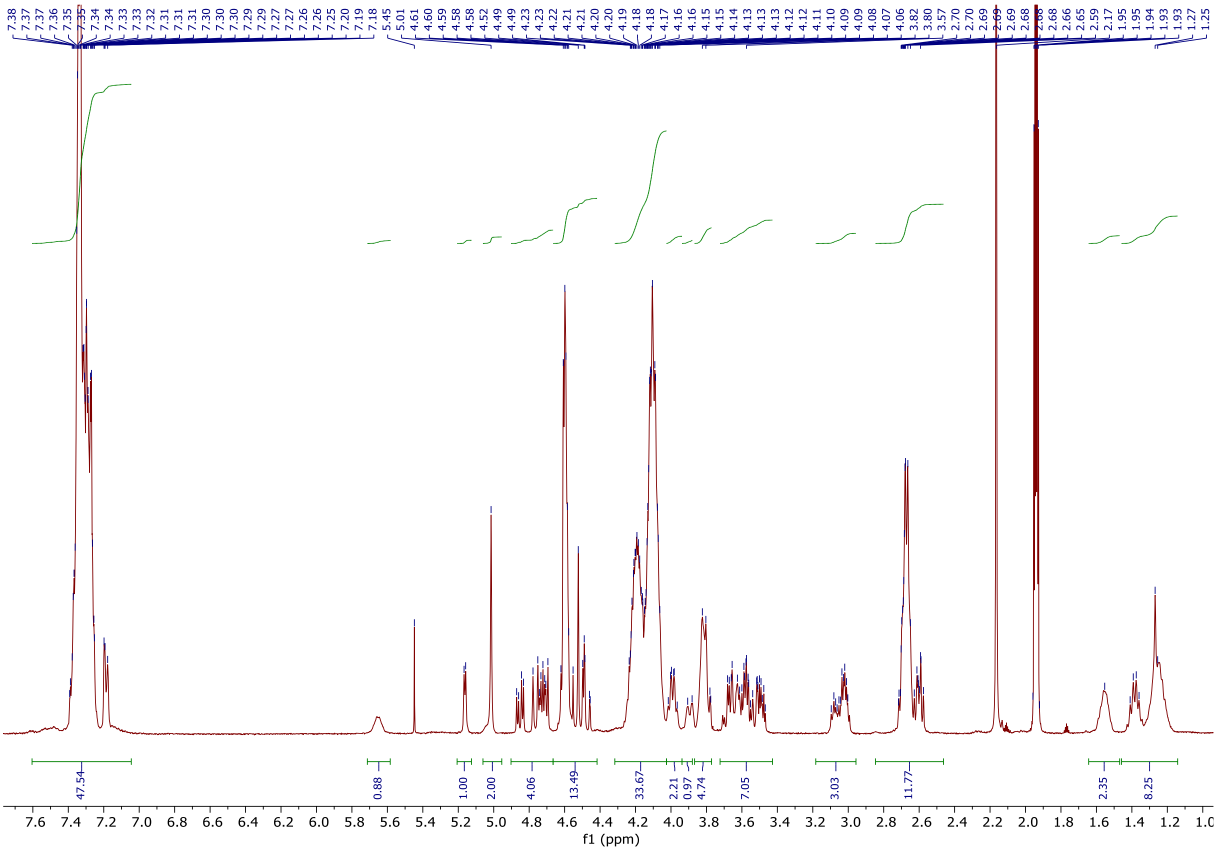

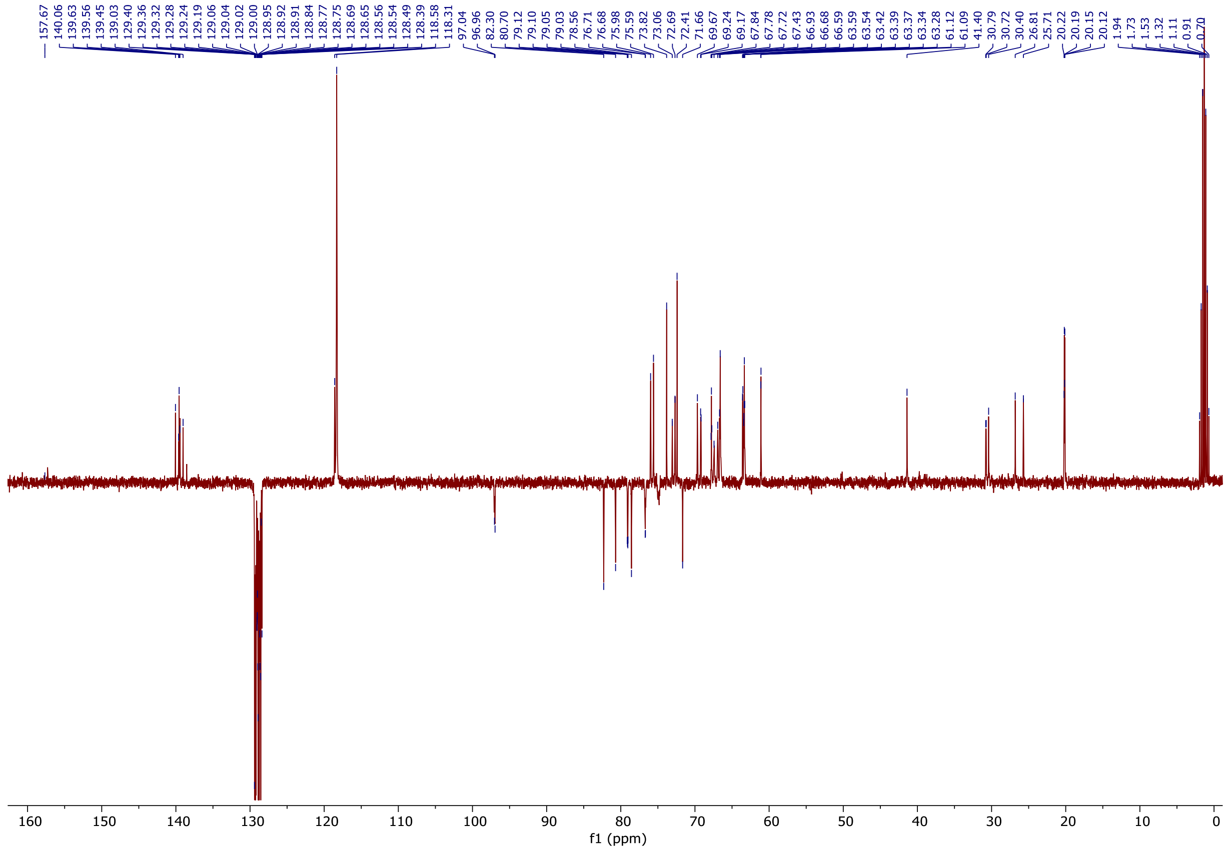
157.67
140.06
139.63
139.56
139.45
139.03
129.40
129.36
129.32
129.28
129.24
129.19
129.06
129.04
129.02
129.00
128.95
128.92
128.91
128.84
128.77
128.75
128.69
128.65
128.56
128.54
128.49
128.39
118.58
118.31
97.04
96.96
82.30
80.70
79.12
79.10
79.05
79.03
78.56
76.71
76.68
75.98
75.59
73.82
73.06
72.69
72.41
71.66
69.67
69.24
69.17
67.84
67.78
67.72
67.43
66.93
66.68
66.59
63.59
63.54
63.42
63.39
63.37
63.34
63.28
61.12
61.09
41.40
30.79
30.72
30.40
26.81
25.71
20.22
20.19
20.15
20.12
1.94
1.73
1.53
1.32
1.11
0.91
0.70
f1 (ppm)

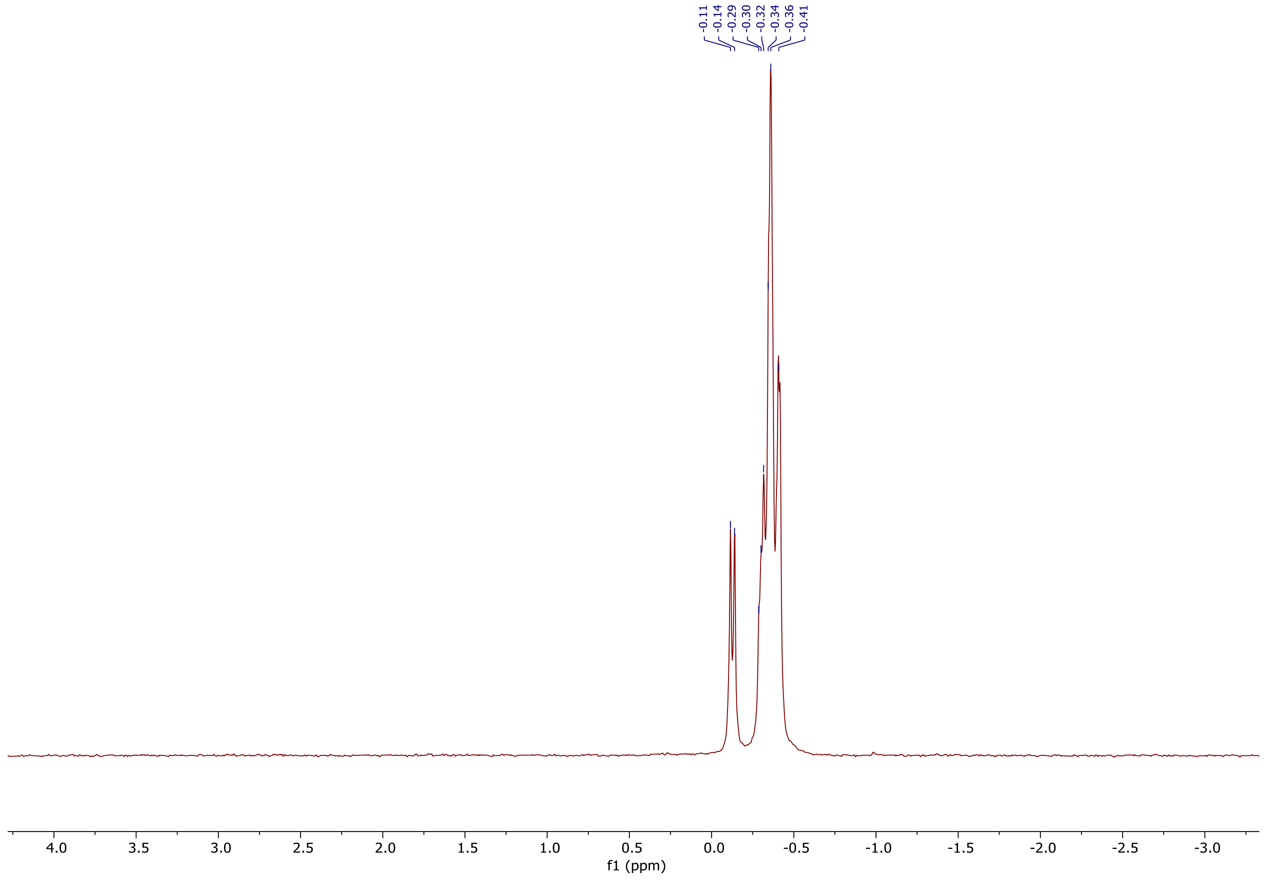
-0.11
-0.14
-0.29
-0.30
-0.32
-0.34
-0.36
-0.41
f1 (ppm)

(Protected) (GlcGroP)(GroP)$_3$-Spacer or Tetramer S12

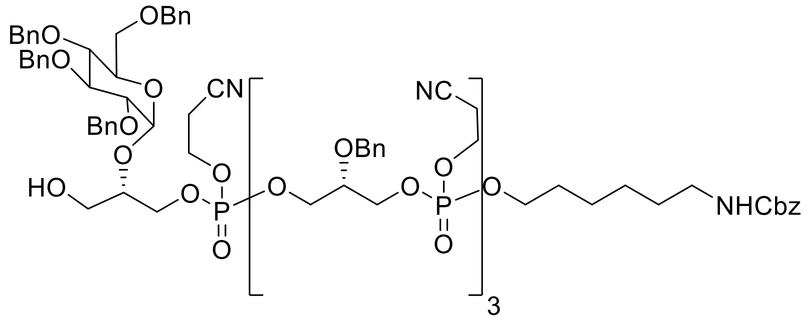

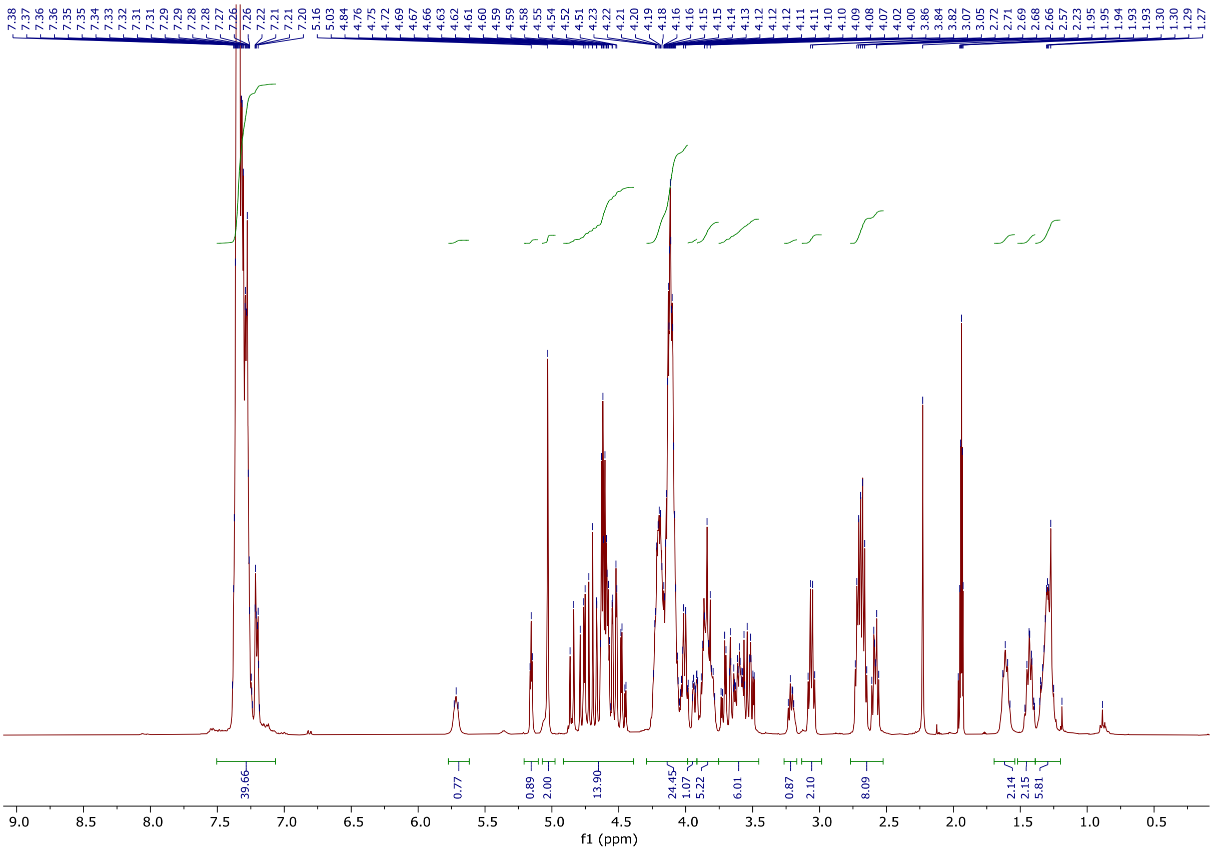

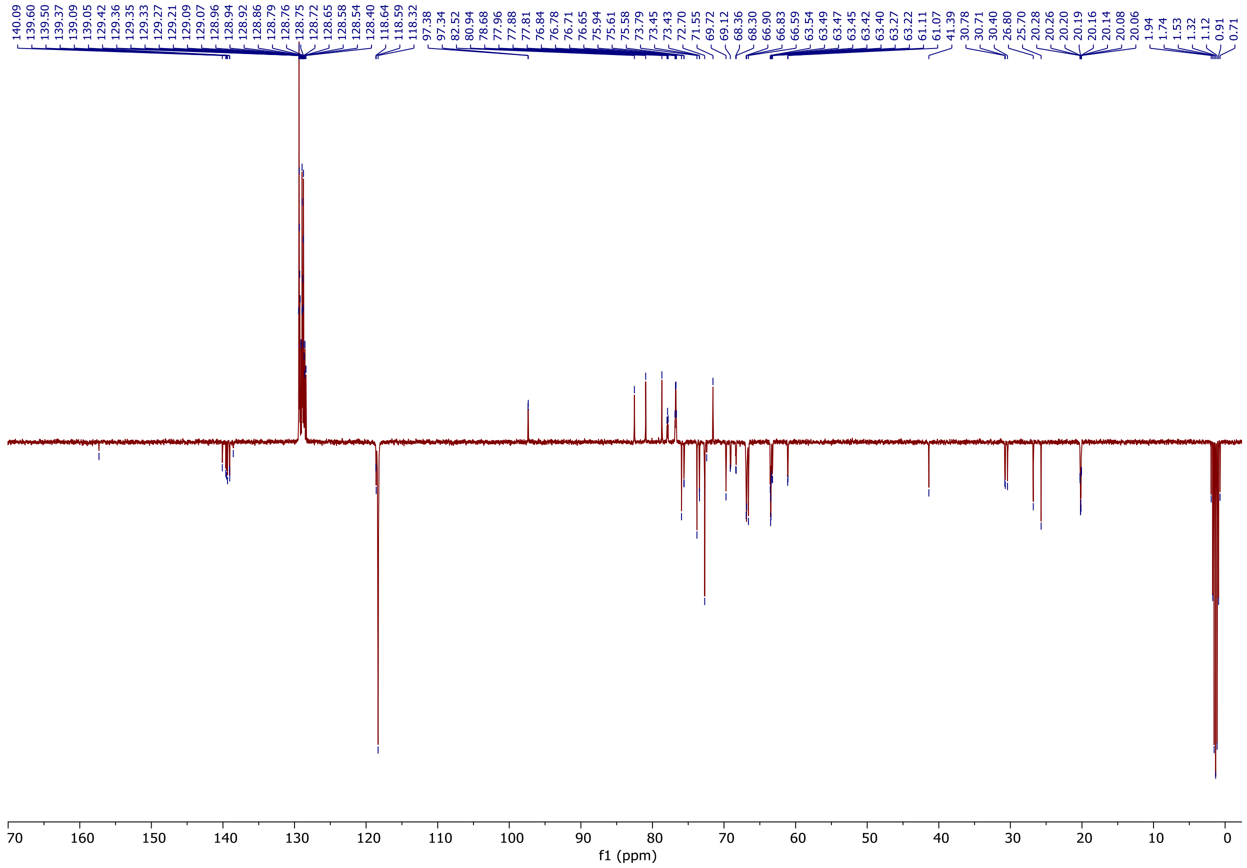
140.09
139.60
139.50
139.37
139.09
139.05
129.42
129.36
129.35
129.33
129.27
129.21
129.09
129.07
128.96
128.94
128.92
128.86
128.79
128.76
128.75
128.72
128.65
128.58
128.54
128.40
118.64
118.59
118.32
97.38
97.34
82.52
80.94
78.68
77.96
77.88
77.81
76.84
76.78
76.71
76.65
75.94
75.61
75.58
73.79
73.45
73.43
72.70
71.55
69.72
69.12
68.36
68.30
66.90
66.83
66.59
63.54
63.49
63.47
63.45
63.42
63.40
63.27
63.22
61.11
61.07
41.39
30.78
30.71
30.40
26.80
25.70
20.28
20.26
20.20
20.19
20.16
20.14
20.08
20.06
1.94
1.74
1.53
1.32
1.12
0.91
0.71
f1 (ppm)

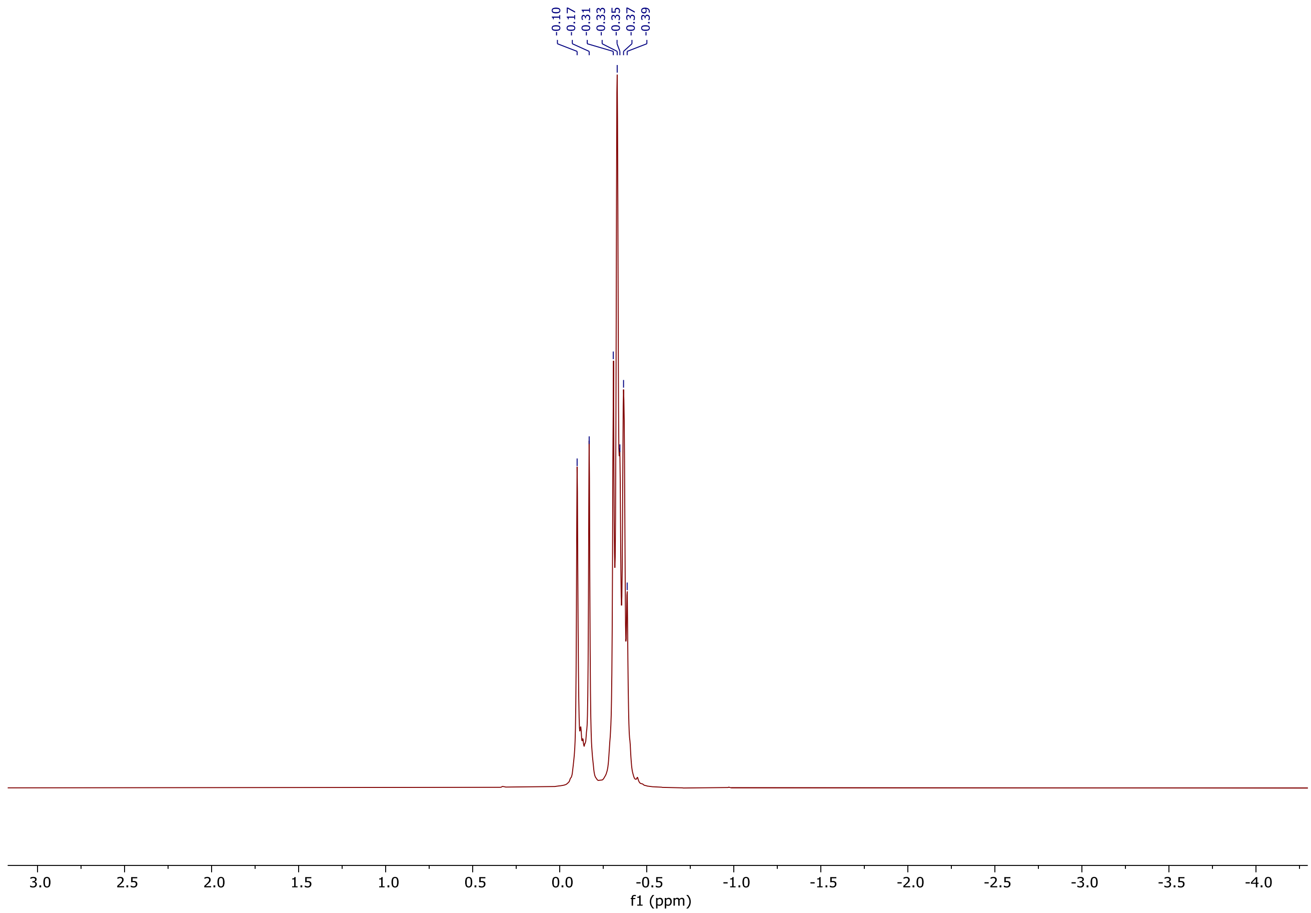
-0.10
-0.17
-0.31
-0.33
-0.35
-0.37
-0.39
f1 (ppm)

(Protected) (GroP)(GlcGroP)(GroP)$_3$-Spacer or Pentamer S13

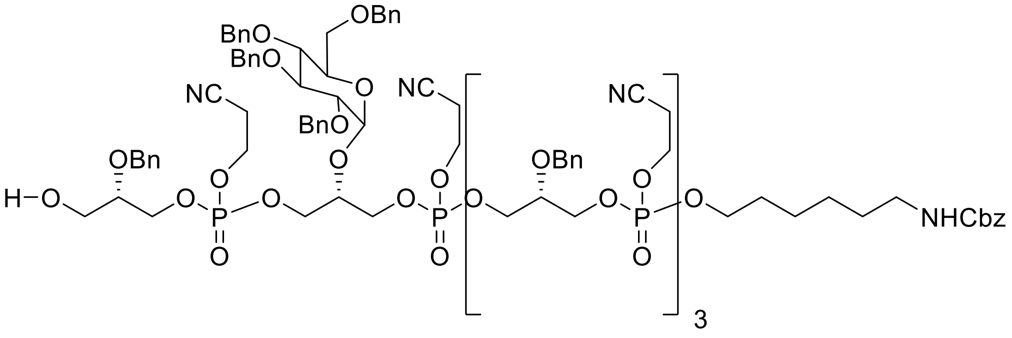

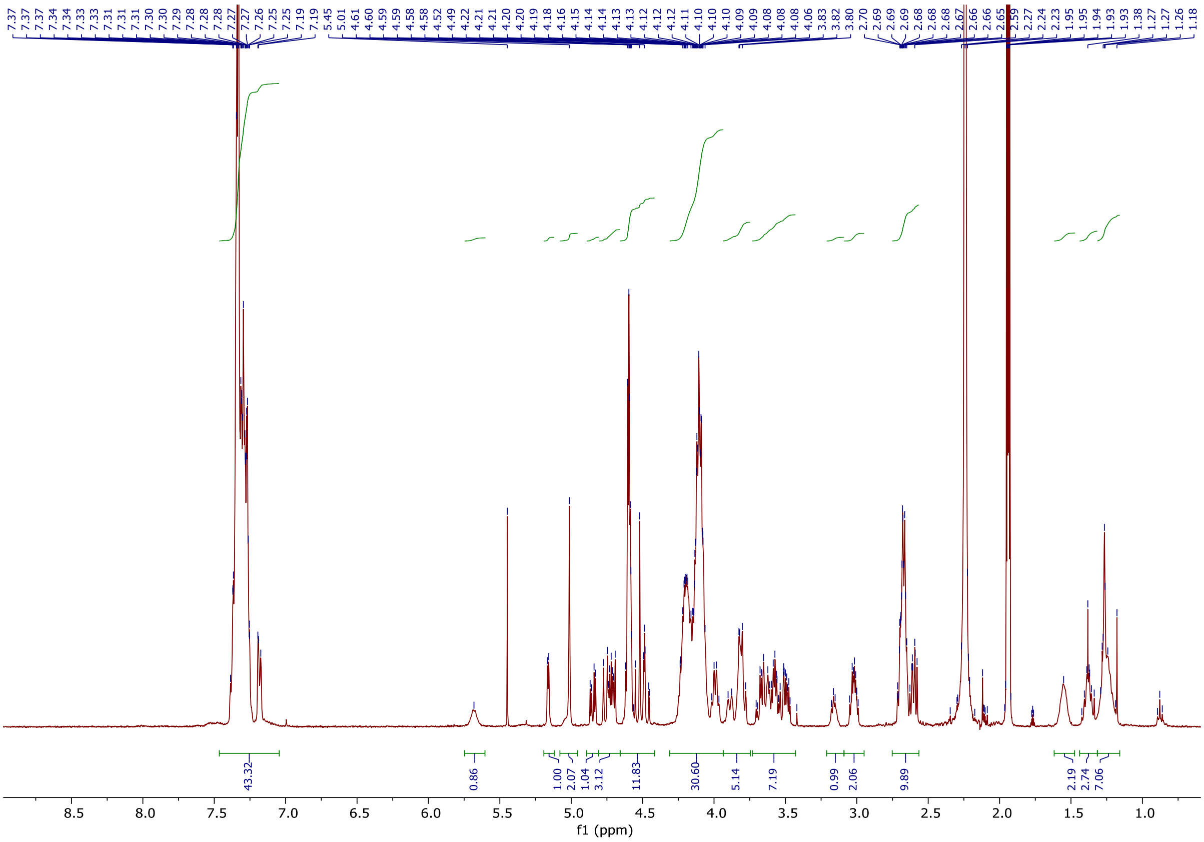

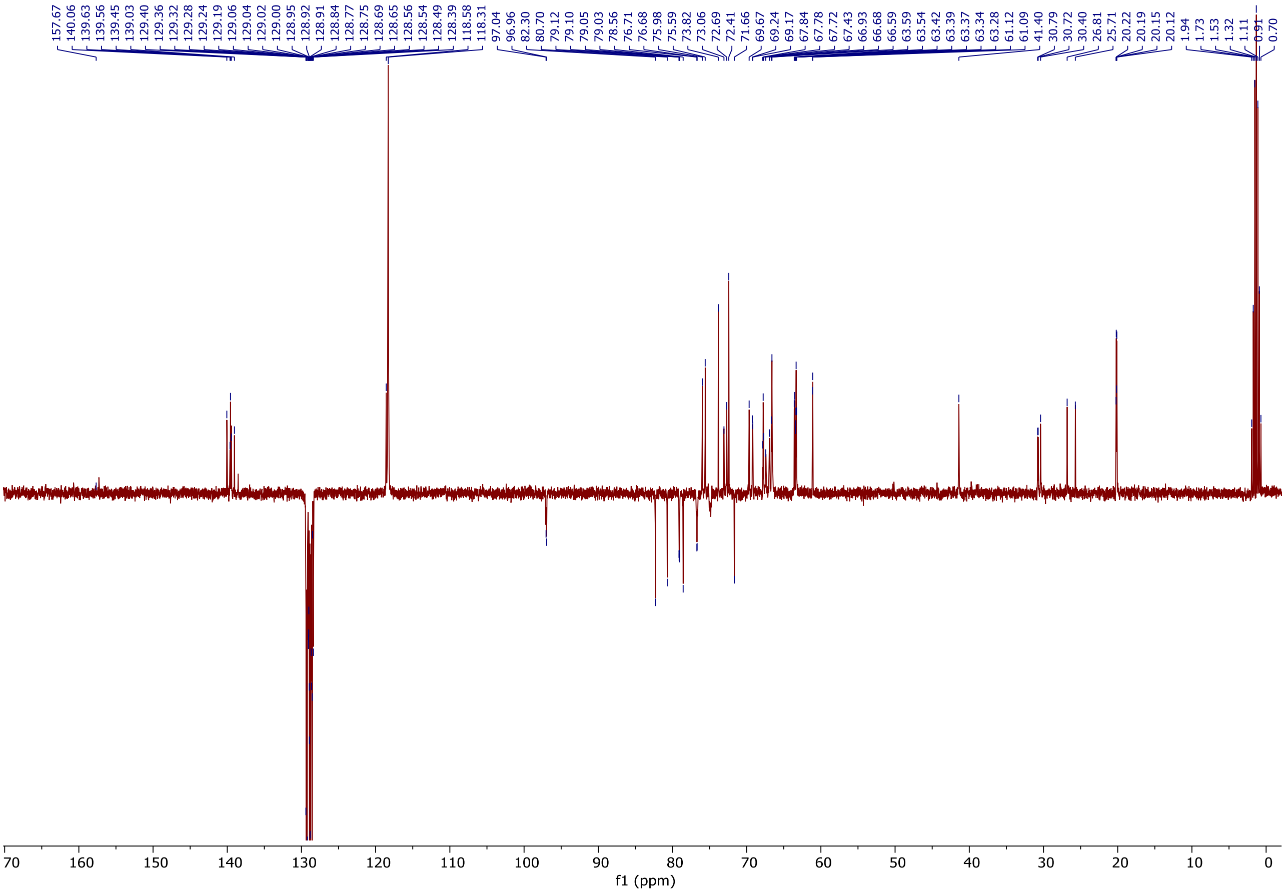
157.67
140.06
139.63
139.56
139.45
139.03
129.40
129.36
129.32
129.28
129.24
129.19
129.06
129.04
129.02
129.00
128.95
128.92
128.91
128.84
128.77
128.75
128.69
128.65
128.56
128.54
128.49
128.39
118.58
118.31
97.04
96.96
82.30
80.70
79.12
79.10
79.05
79.03
78.56
76.71
76.68
75.98
75.59
73.82
73.06
72.69
72.41
71.66
69.67
69.24
69.17
67.84
67.78
67.72
67.43
66.93
66.68
66.59
63.59
63.54
63.42
63.39
63.37
63.34
63.28
61.12
61.09
41.40
30.79
30.72
30.40
26.81
25.71
20.22
20.19
20.15
20.12
1.94
1.73
1.53
1.32
1.11
0.91
0.70
f1 (ppm)

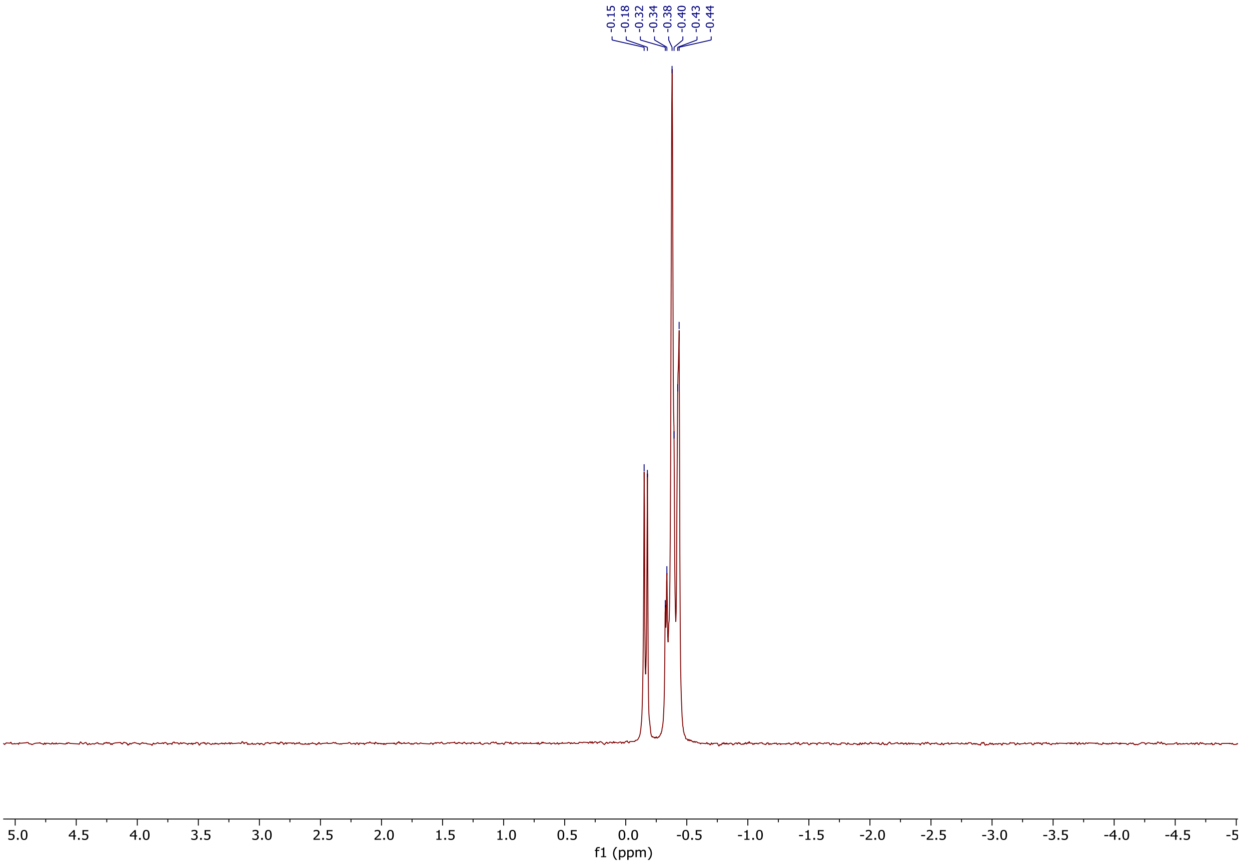
-0.15
-0.18
-0.32
-0.34
-0.38
-0.40
-0.43
-0.44
f1 (ppm)

(Protected) (GroP)$_2$(GlcGroP)(GroP)$_3$-Spacer or Hexamer 25

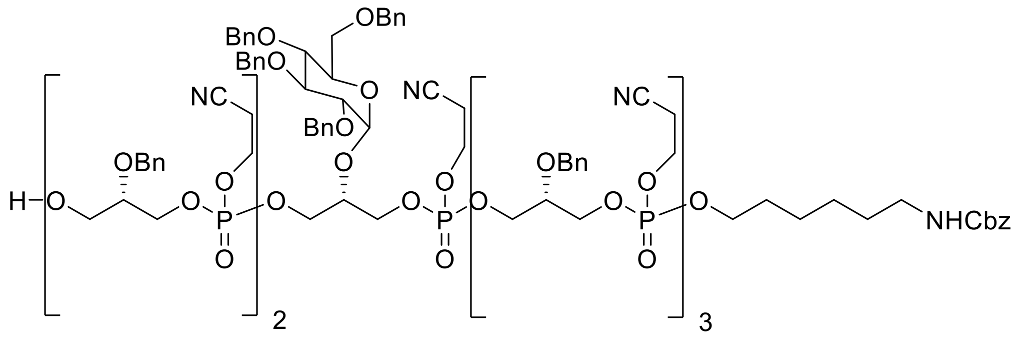

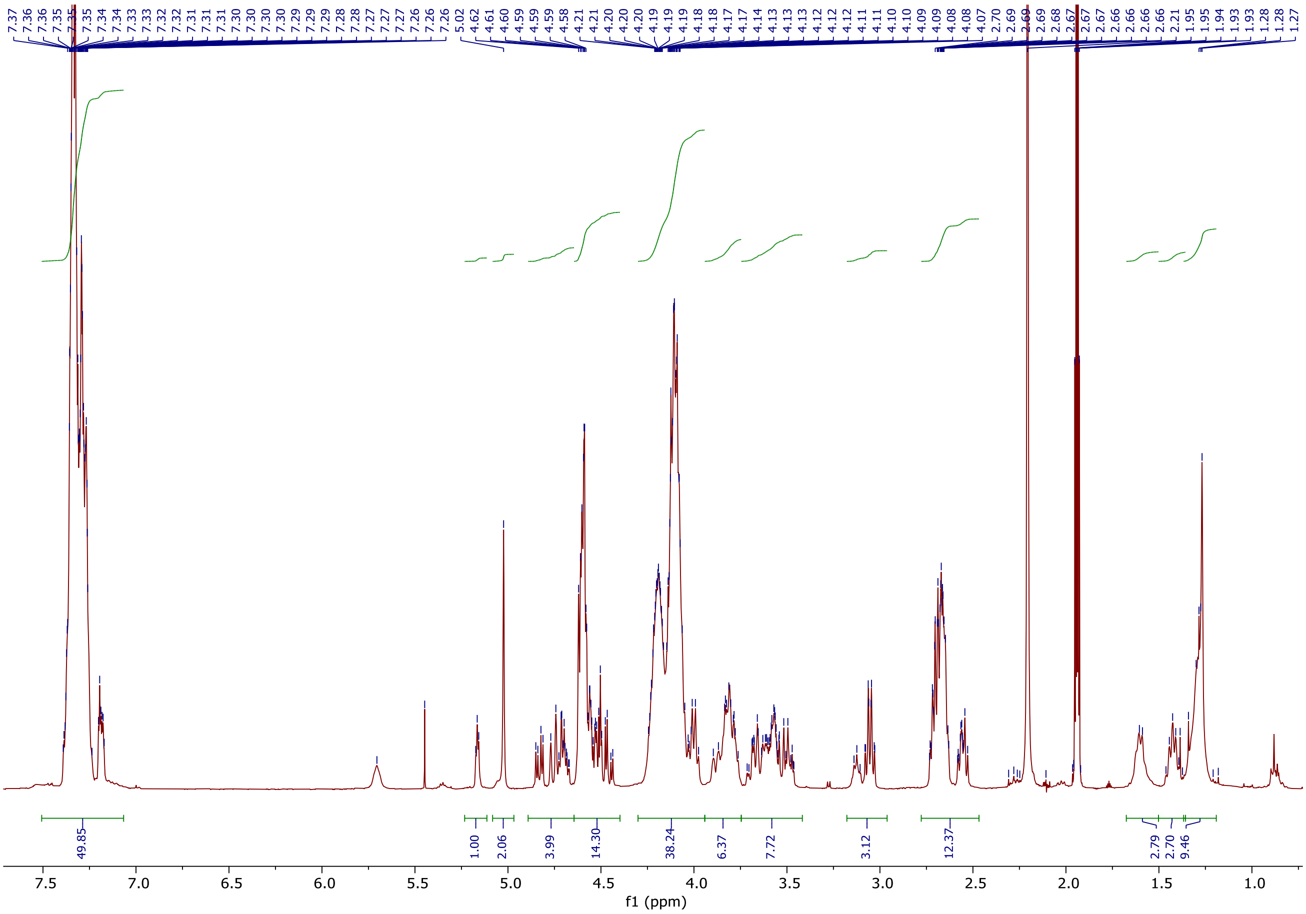

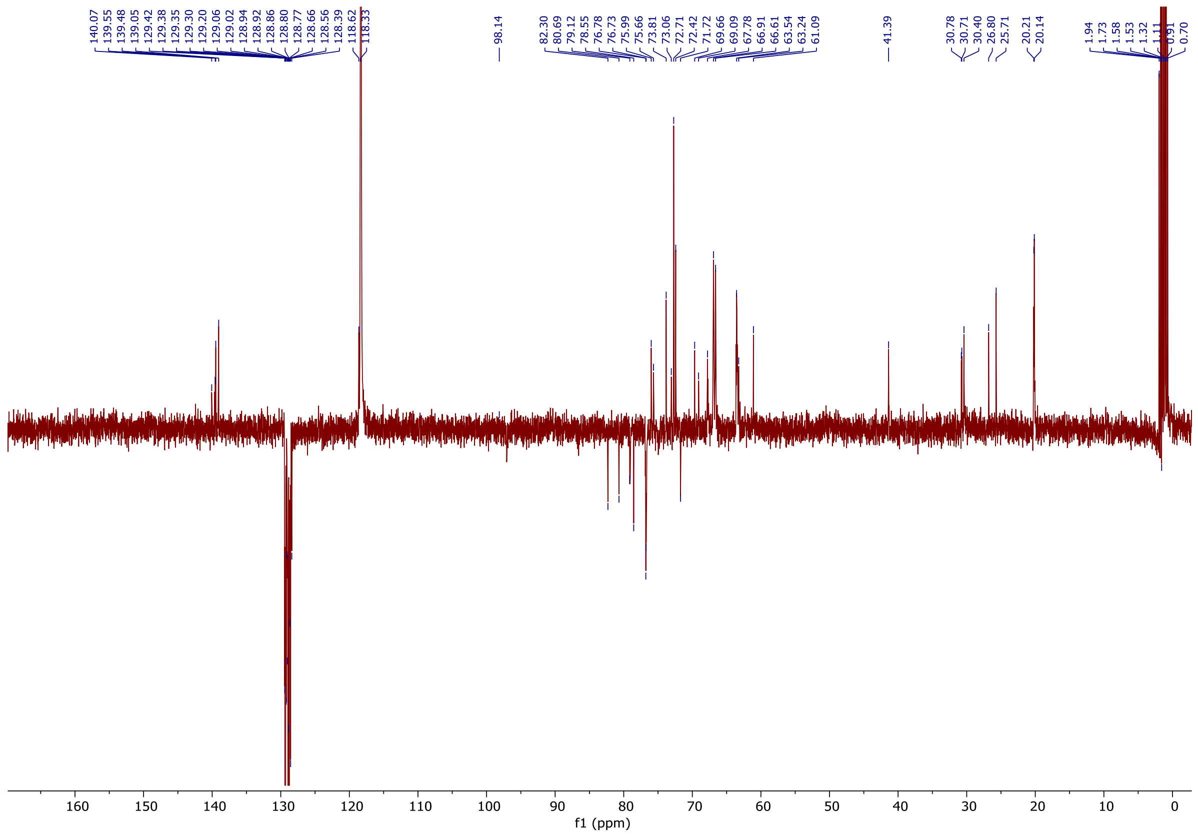
140.07
139.55
139.48
139.05
129.42
129.38
129.35
129.30
129.20
129.06
129.02
128.94
128.92
128.86
128.80
128.77
128.66
128.56
128.39
118.62
118.33
98.14
82.30
80.69
79.12
78.55
76.78
76.73
75.99
75.66
73.81
73.06
72.71
72.42
71.72
69.66
69.09
67.78
66.91
66.61
63.54
63.24
61.09
41.39
30.78
30.71
30.40
26.80
25.71
20.21
20.14
1.94
1.73
1.58
1.53
1.32
1.11
0.91
0.70
f1 (ppm)

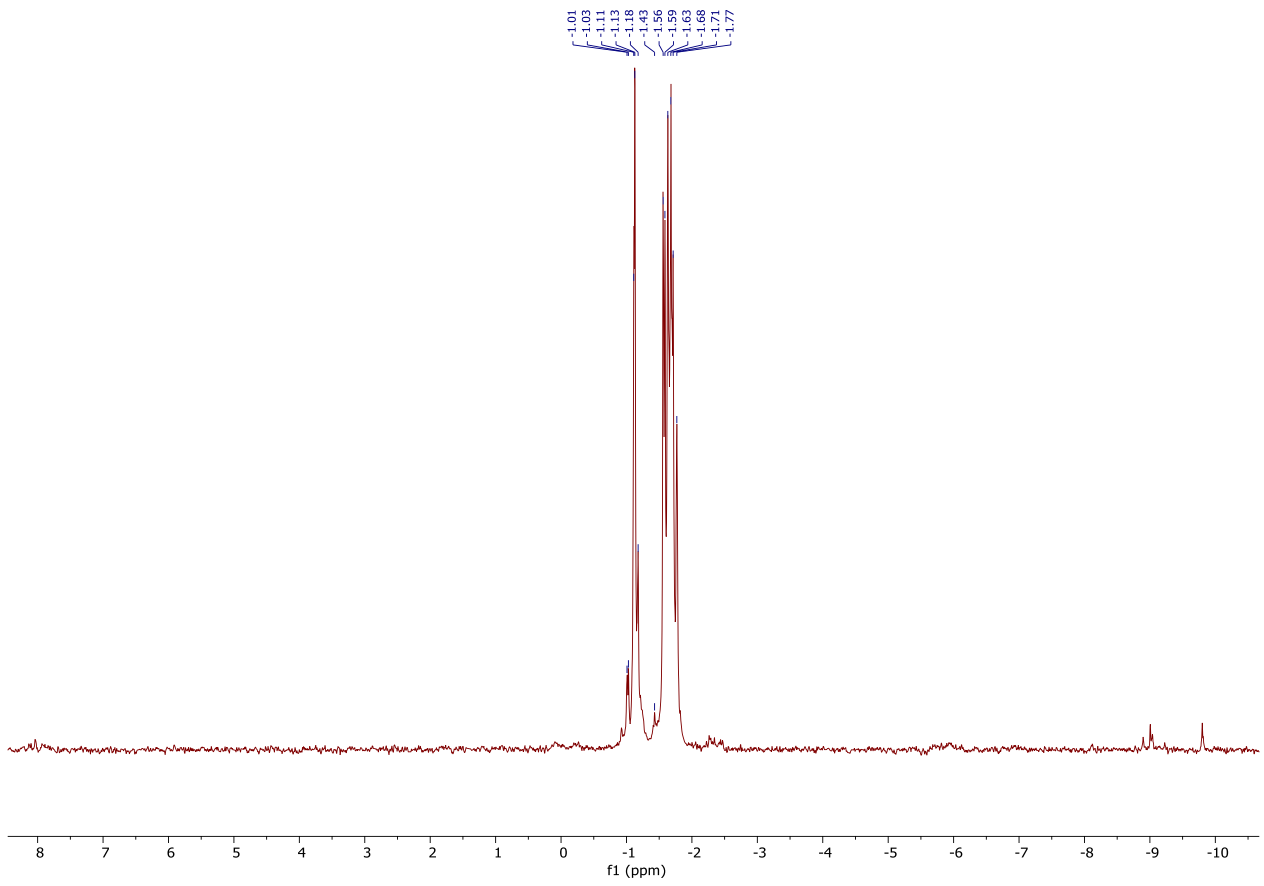
-1.01
-1.03
-1.11
-1.13
-1.18
-1.43
-1.56
-1.59
-1.63
-1.68
-1.71
-1.77
f1 (ppm)

(Protected) (GlcGroP)(GroP)$_2$-Spacer or Trimer S14

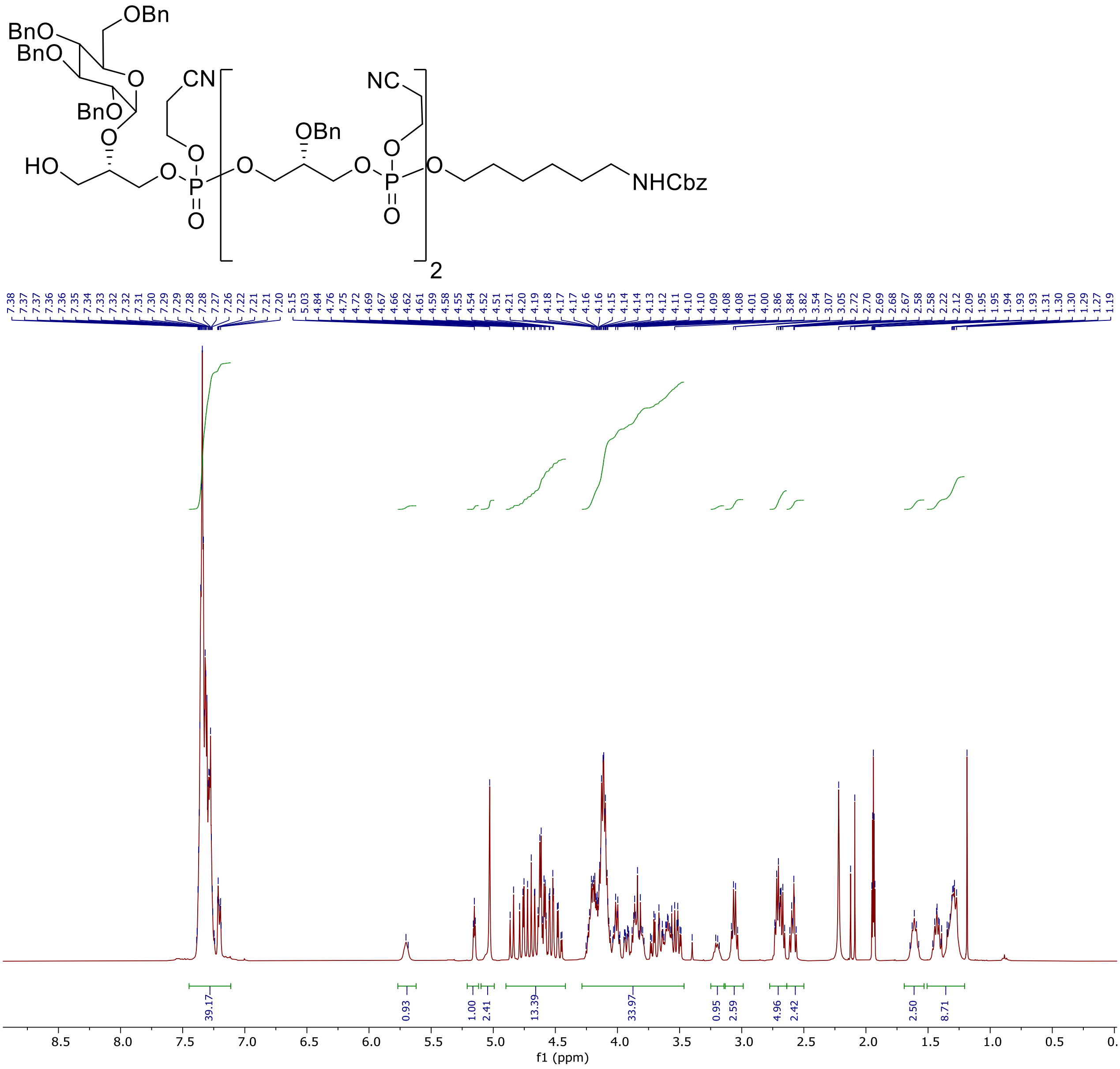

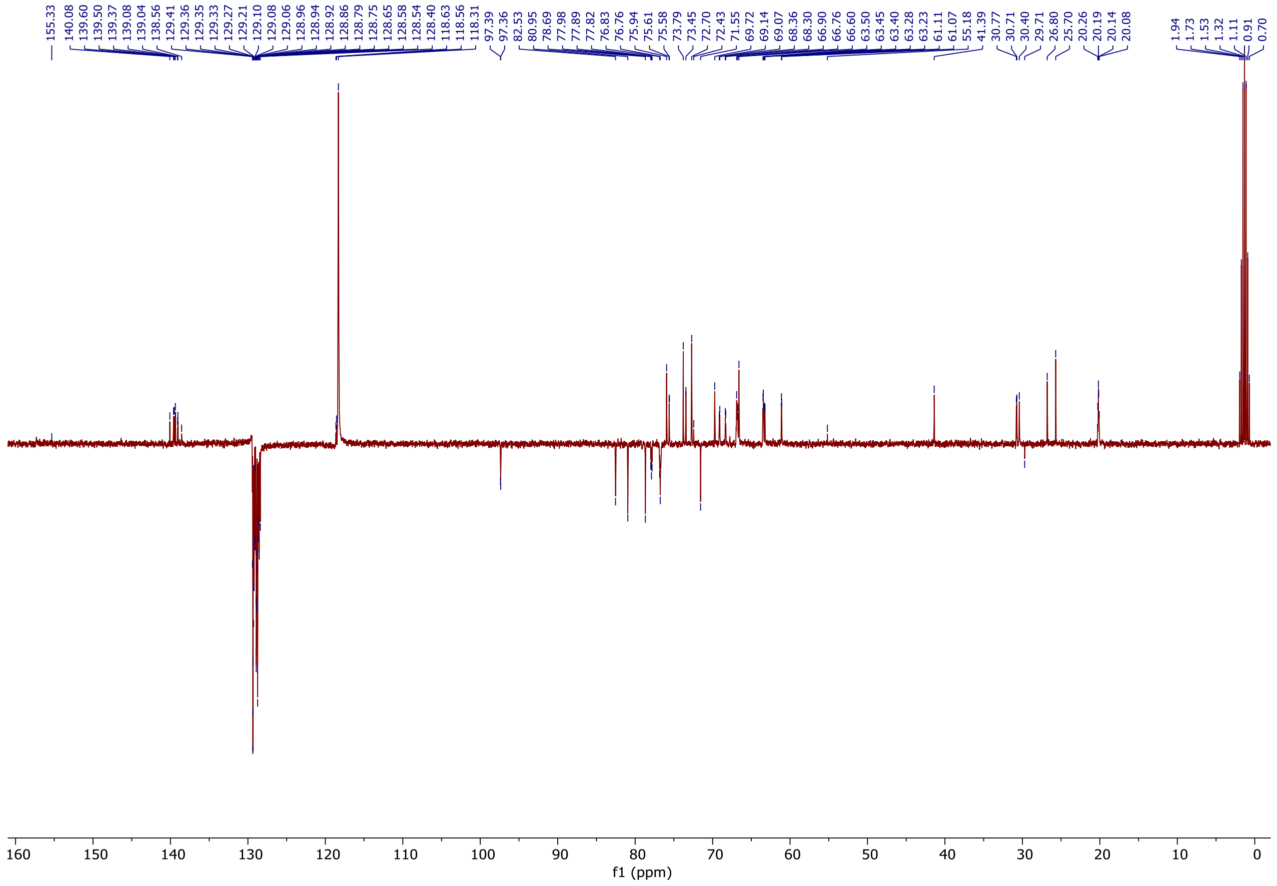
155.33
140.08
139.60
139.50
139.37
139.08
139.04
138.56
129.41
129.36
129.35
129.33
129.27
129.21
129.10
129.08
129.06
128.96
128.94
128.92
128.86
128.79
128.75
128.65
128.58
128.54
128.40
118.63
118.56
118.31
97.39
97.36
82.53
80.95
78.69
77.98
77.89
77.82
76.83
76.76
75.94
75.61
75.58
73.79
73.45
72.70
72.43
71.55
69.72
69.14
69.07
68.36
68.30
66.90
66.76
66.60
63.50
63.45
63.40
63.28
63.23
61.11
61.07
55.18
41.39
30.77
30.71
30.40
29.71
26.80
25.70
20.26
20.19
20.14
20.08
1.94
1.73
1.53
1.32
1.11
0.91
0.70
f1 (ppm)

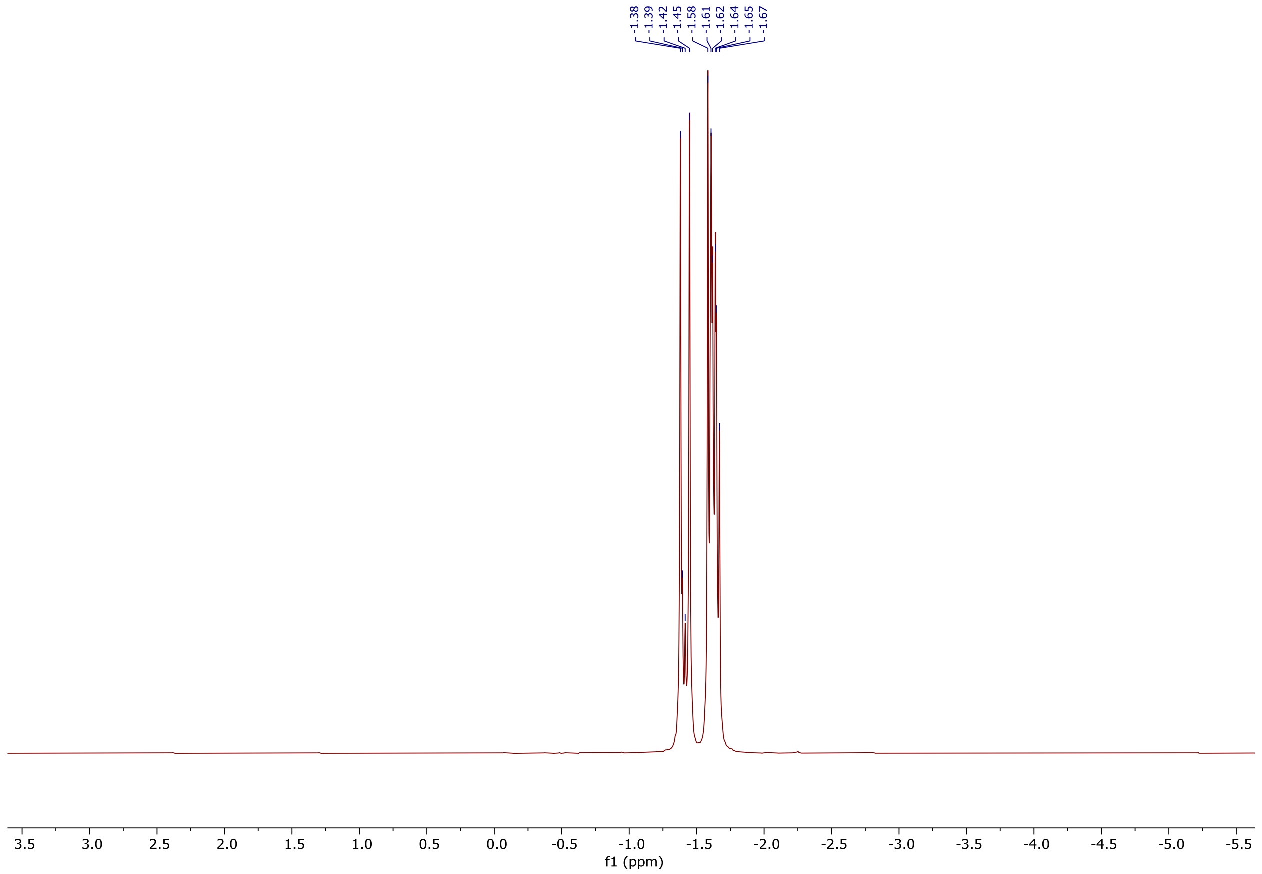
-1.38
-1.39
-1.42
-1.45
-1.58
-1.61
-1.62
-1.64
-1.65
-1.67
f1 (ppm)

(Protected) (GroP)(GlcGroP)(GroP)$_2$-Spacer or Tetramer S15

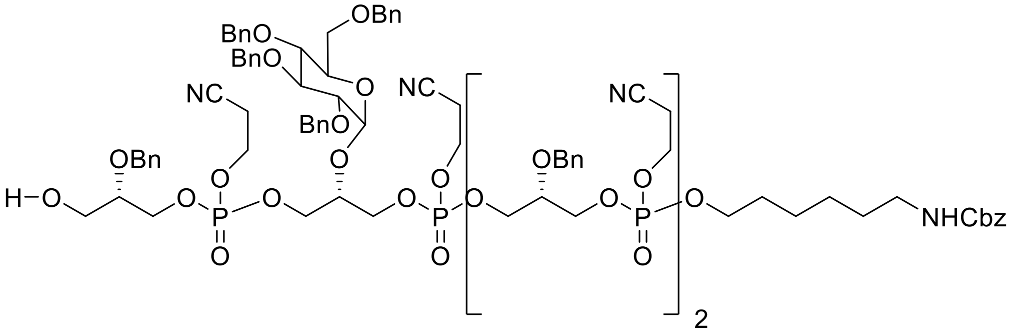

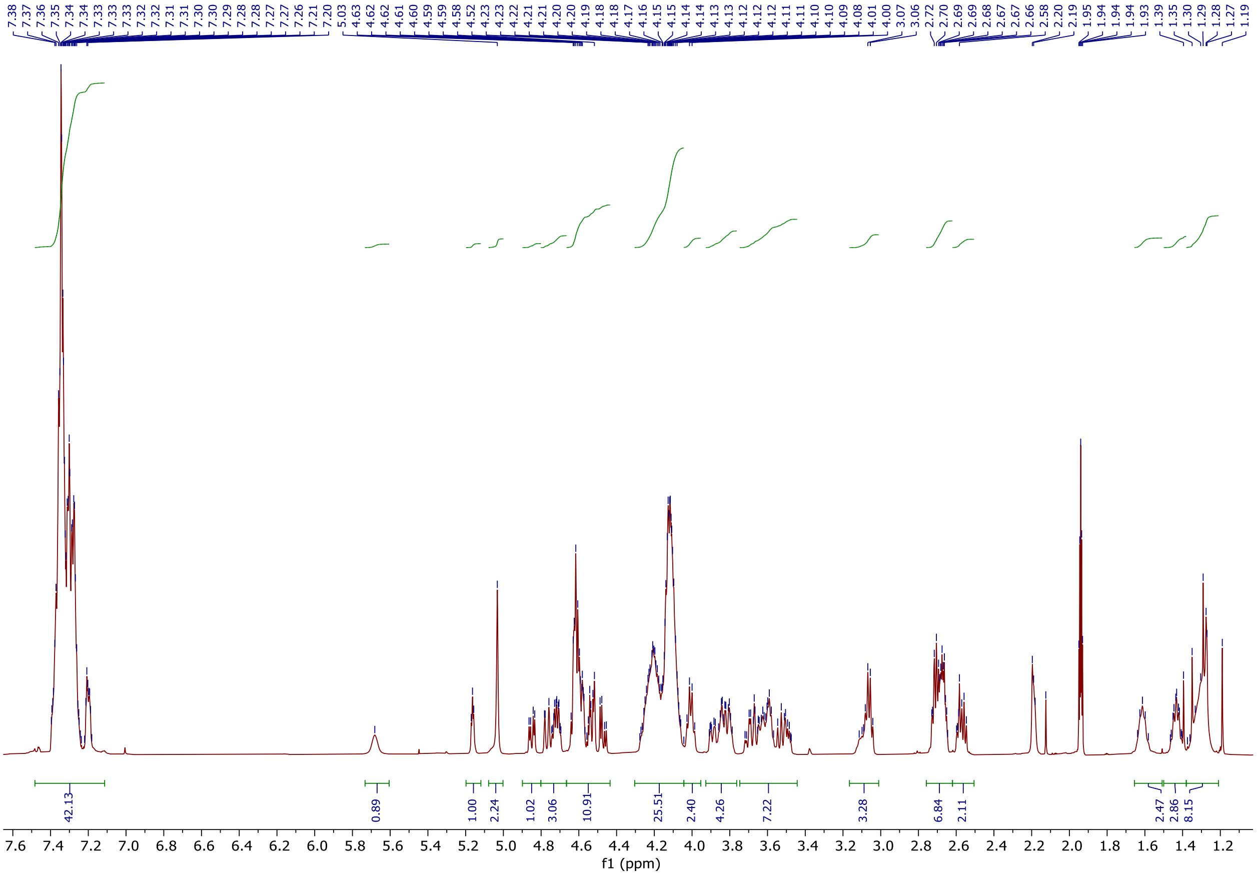

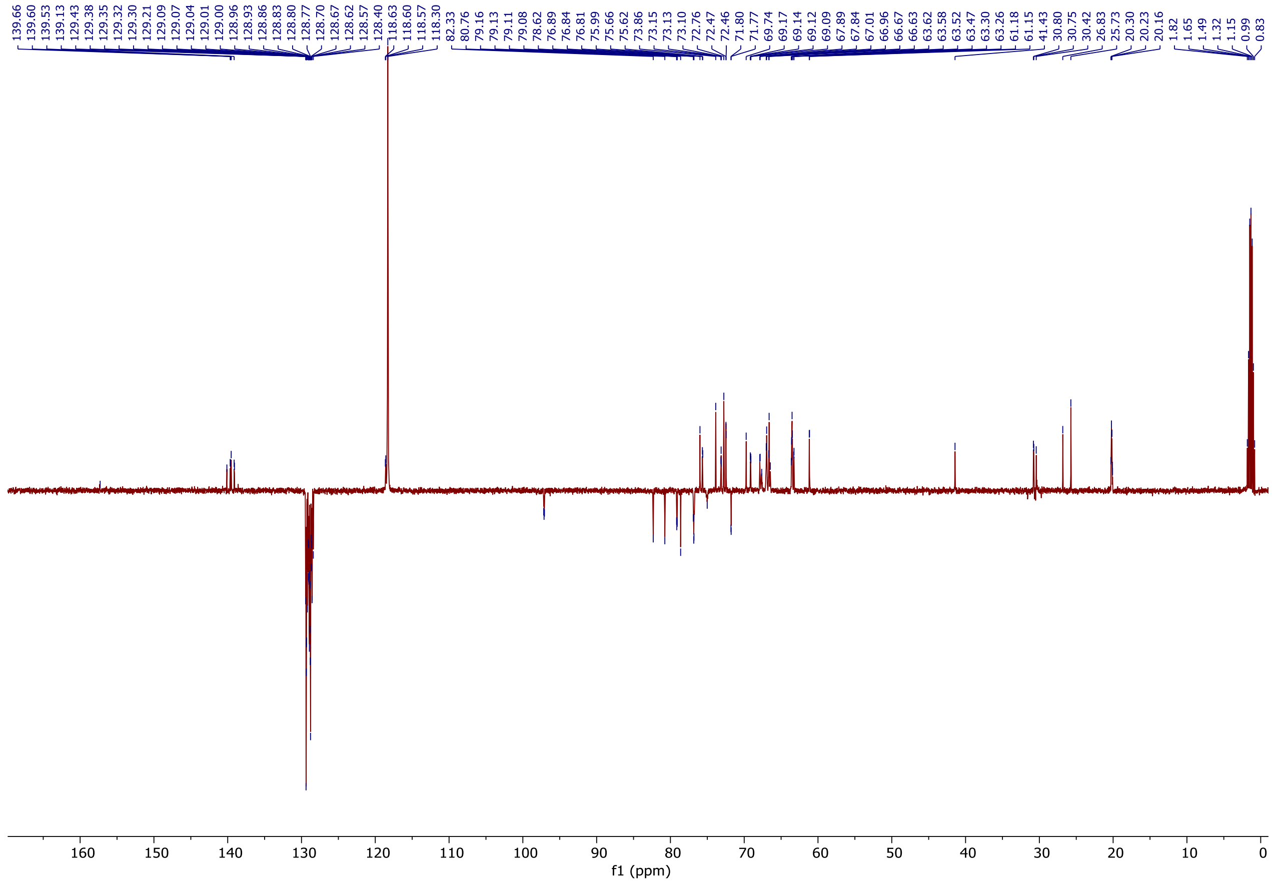
139.66
139.60
139.53
139.13
129.43
129.38
129.35
129.32
129.30
129.21
129.09
129.07
129.04
129.01
129.00
128.96
128.93
128.86
128.83
128.80
128.77
128.70
128.67
128.62
128.57
128.40
118.63
118.60
118.57
118.30
82.33
80.76
79.16
79.13
79.11
79.08
78.62
76.89
76.84
76.81
75.99
75.66
75.62
73.86
73.15
73.13
73.10
72.76
72.47
72.46
71.80
71.77
69.74
69.17
69.14
69.12
69.09
67.89
67.84
67.01
66.96
66.67
66.63
63.62
63.58
63.52
63.47
63.30
63.26
61.18
61.15
41.43
30.80
30.75
30.42
26.83
25.73
20.30
20.23
20.16
1.82
1.65
1.49
1.32
1.15
0.99
0.83
160 150 140 130 120 110 100 90 80 70 60 50 40 30 20 10 0
f1 (ppm)

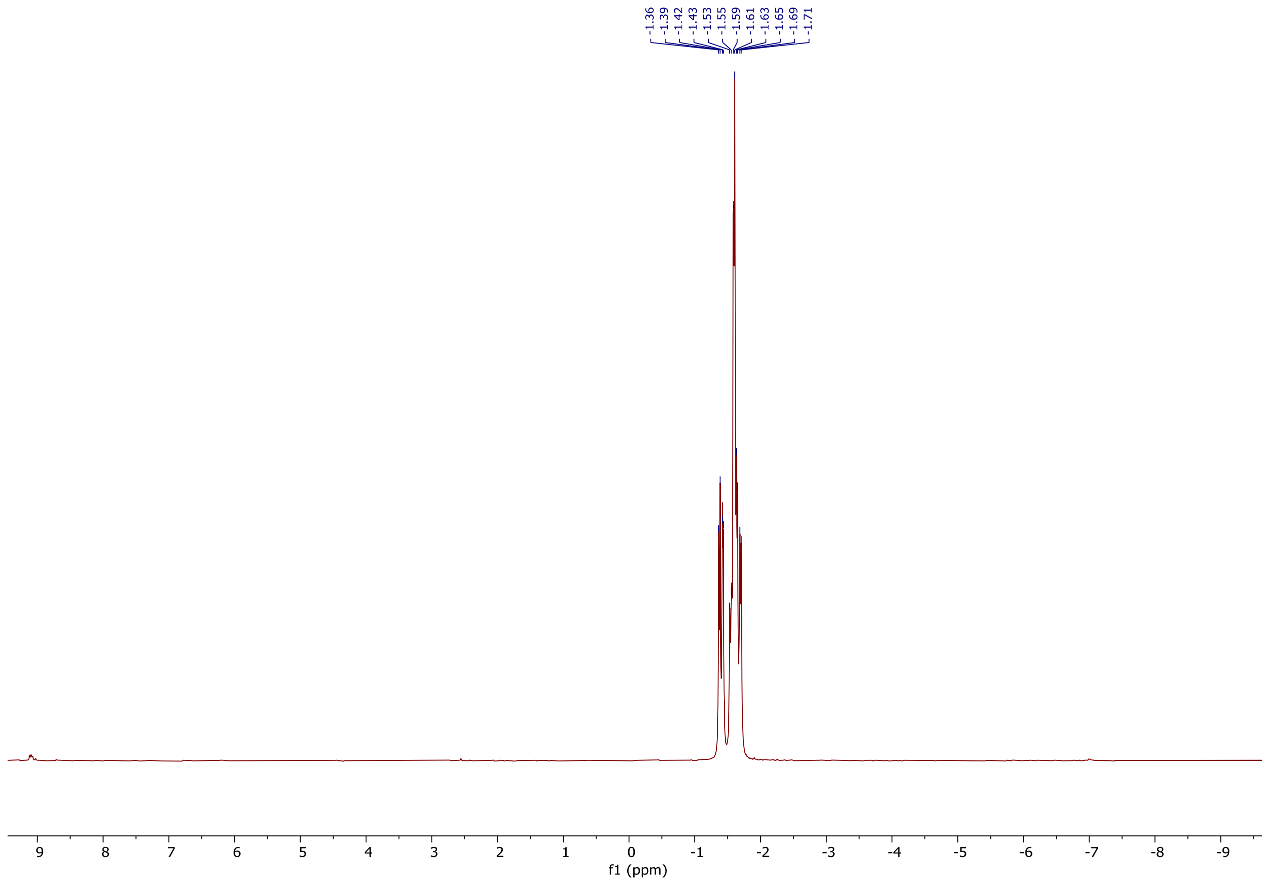
-1.36
-1.39
-1.42
-1.43
-1.53
-1.55
-1.59
-1.61
-1.63
-1.65
-1.69
-1.71
9 8 7 6 5 4 3 2 1 0 -1 -2 -3 -4 -5 -6 -7 -8 -9
f1 (ppm)

(Protected) $(GroP)_2(GlcGroP)(GroP)_2$-Spacer or Pentamer S16

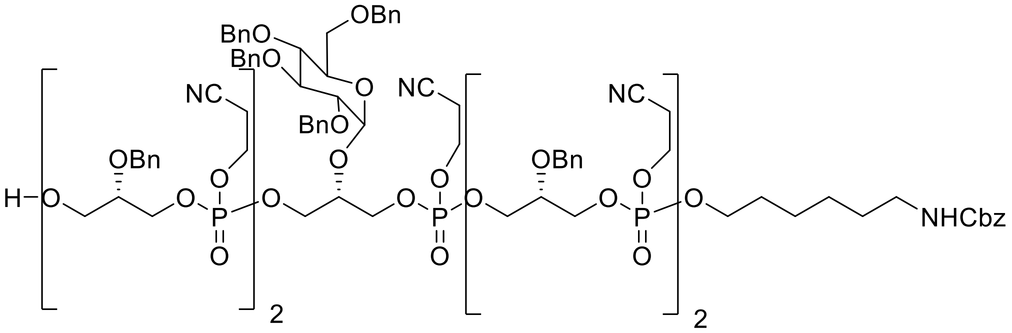

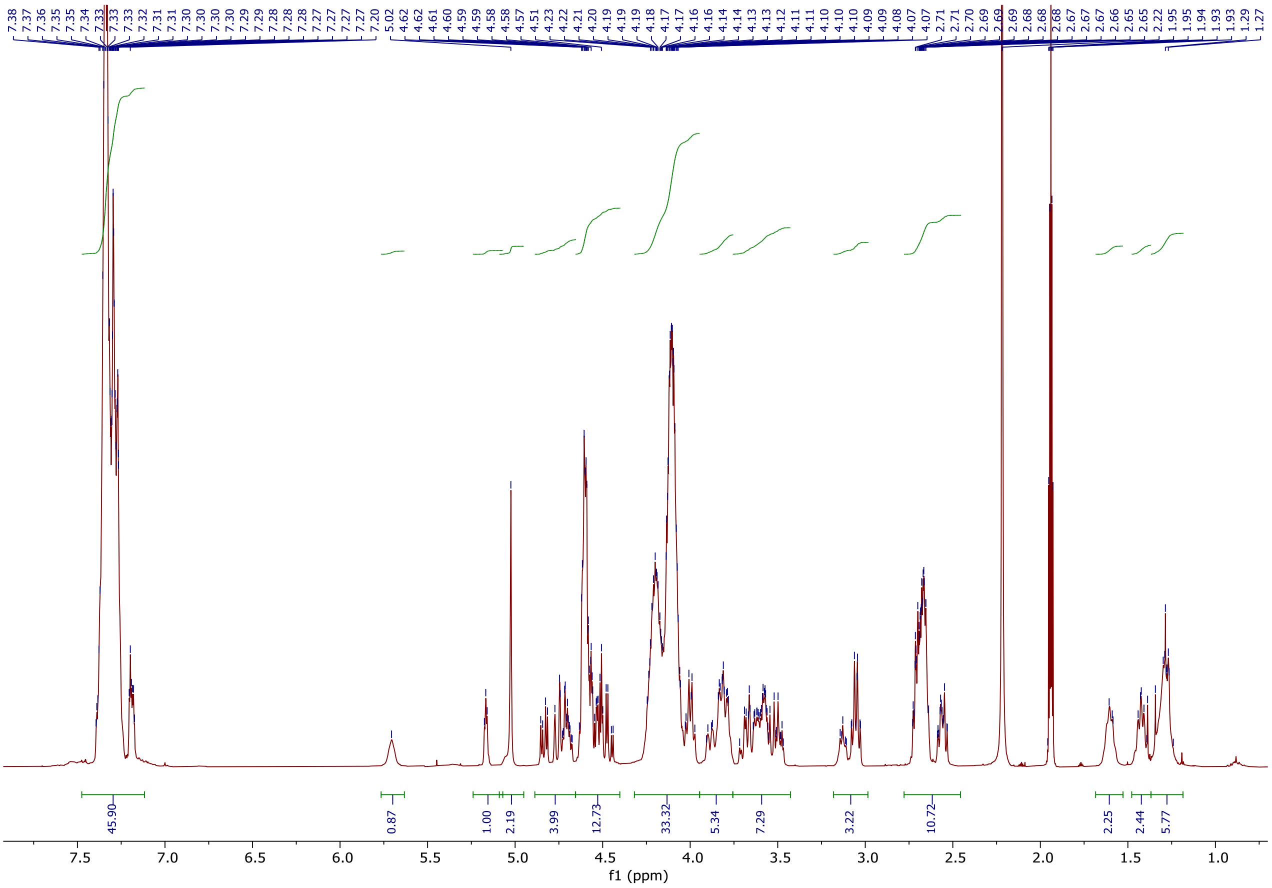

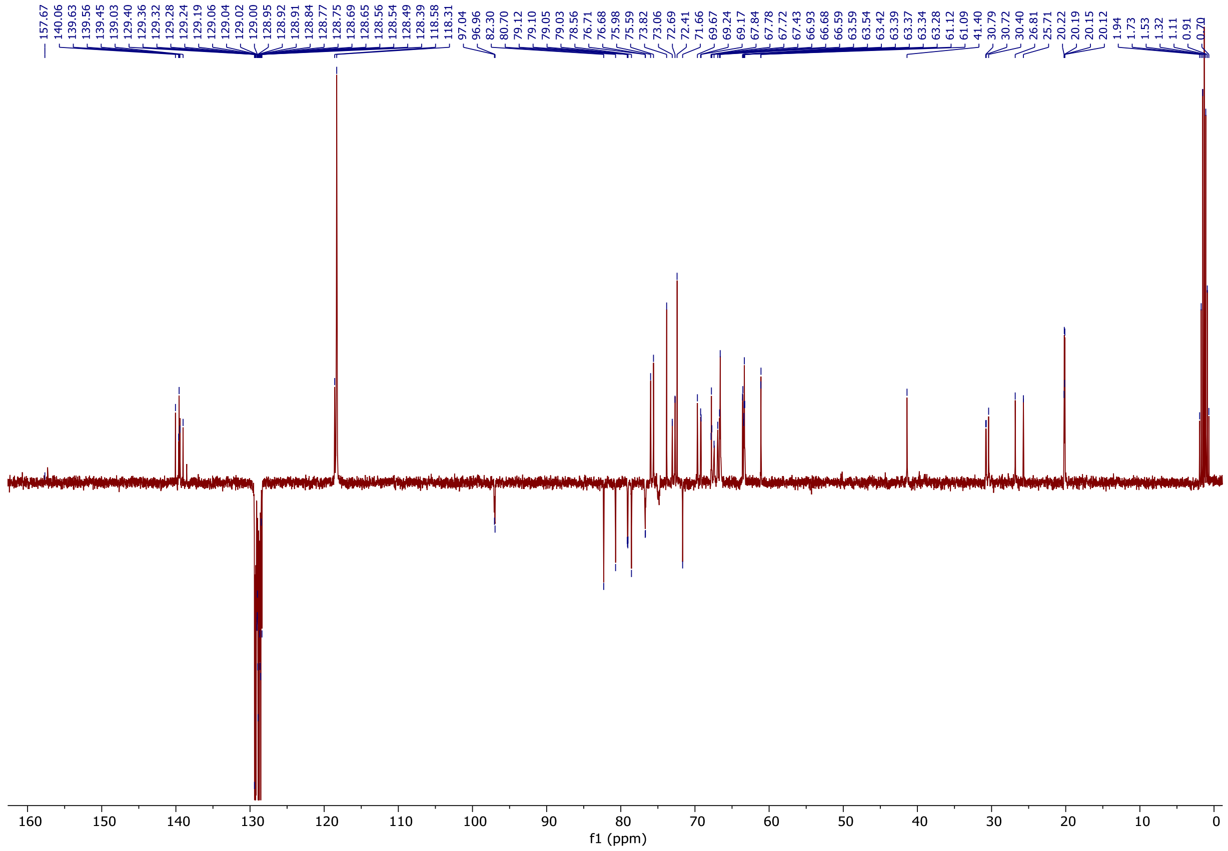
157.67
140.06
139.63
139.56
139.45
139.03
129.40
129.36
129.32
129.28
129.24
129.19
129.06
129.04
129.02
129.00
128.95
128.92
128.91
128.84
128.77
128.75
128.69
128.65
128.56
128.54
128.49
128.39
118.58
118.31
97.04
96.96
82.30
80.70
79.12
79.10
79.05
79.03
78.56
76.71
76.68
75.98
75.59
73.82
73.06
72.69
72.41
71.66
69.67
69.24
69.17
67.84
67.78
67.72
67.43
66.93
66.68
66.59
63.59
63.54
63.42
63.39
63.37
63.34
63.28
61.12
61.09
41.40
30.79
30.72
30.40
26.81
25.71
20.22
20.19
20.15
20.12
1.94
1.73
1.53
1.32
1.11
0.91
0.70
160
150
140
130
120
110
100
90
80
70
60
50
40
30
20
10
0
f1 (ppm)

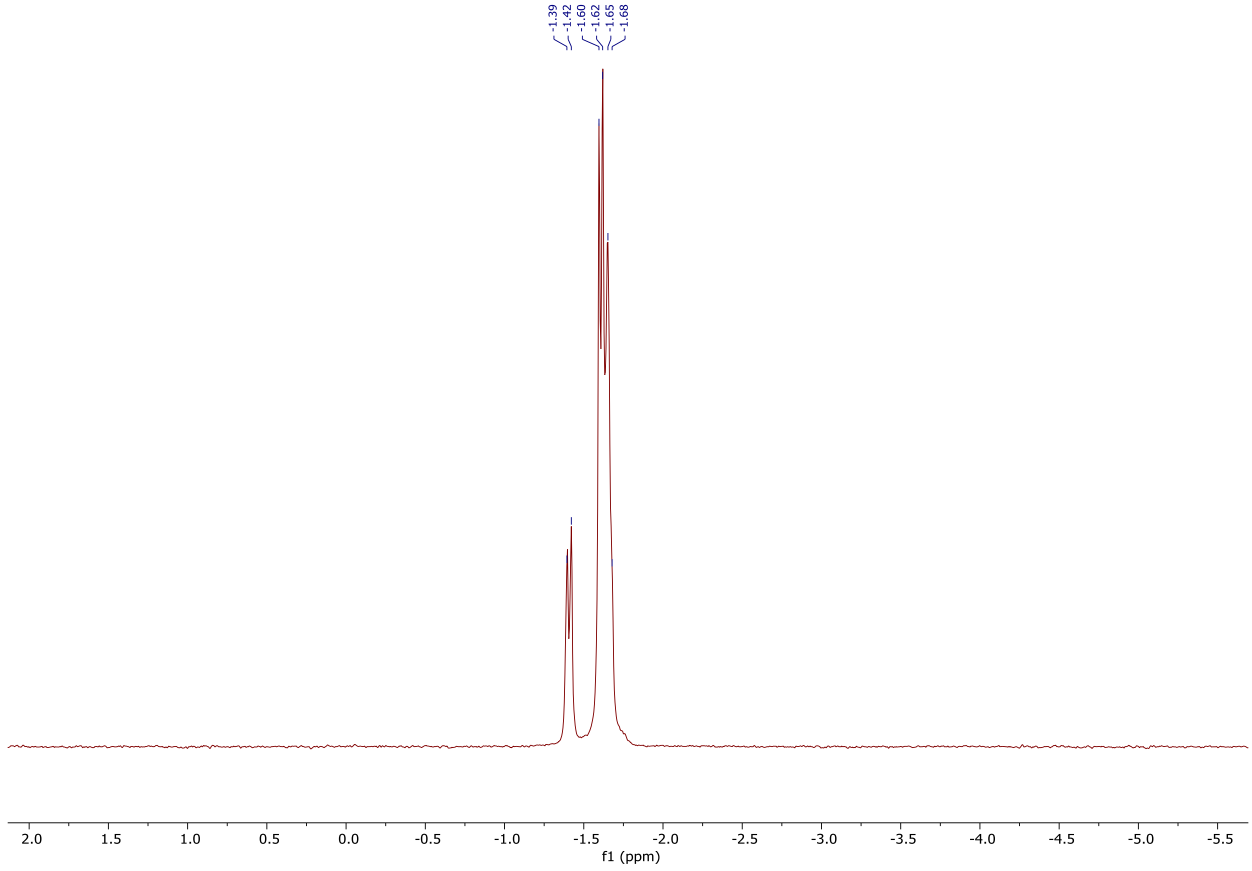
-1.39
-1.42
-1.60
-1.62
-1.65
-1.68
2.0
1.5
1.0
0.5
0.0
-0.5
-1.0
-1.5
-2.0
-2.5
-3.0
-3.5
-4.0
-4.5
-5.0
-5.5
f1 (ppm)

(Protected) (GroP)$_3$(GlcGroP)(GroP)$_2$-Spacer or Hexamer 26

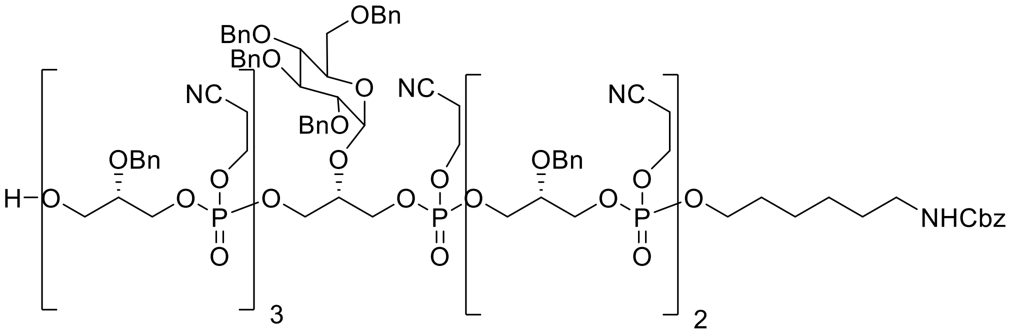

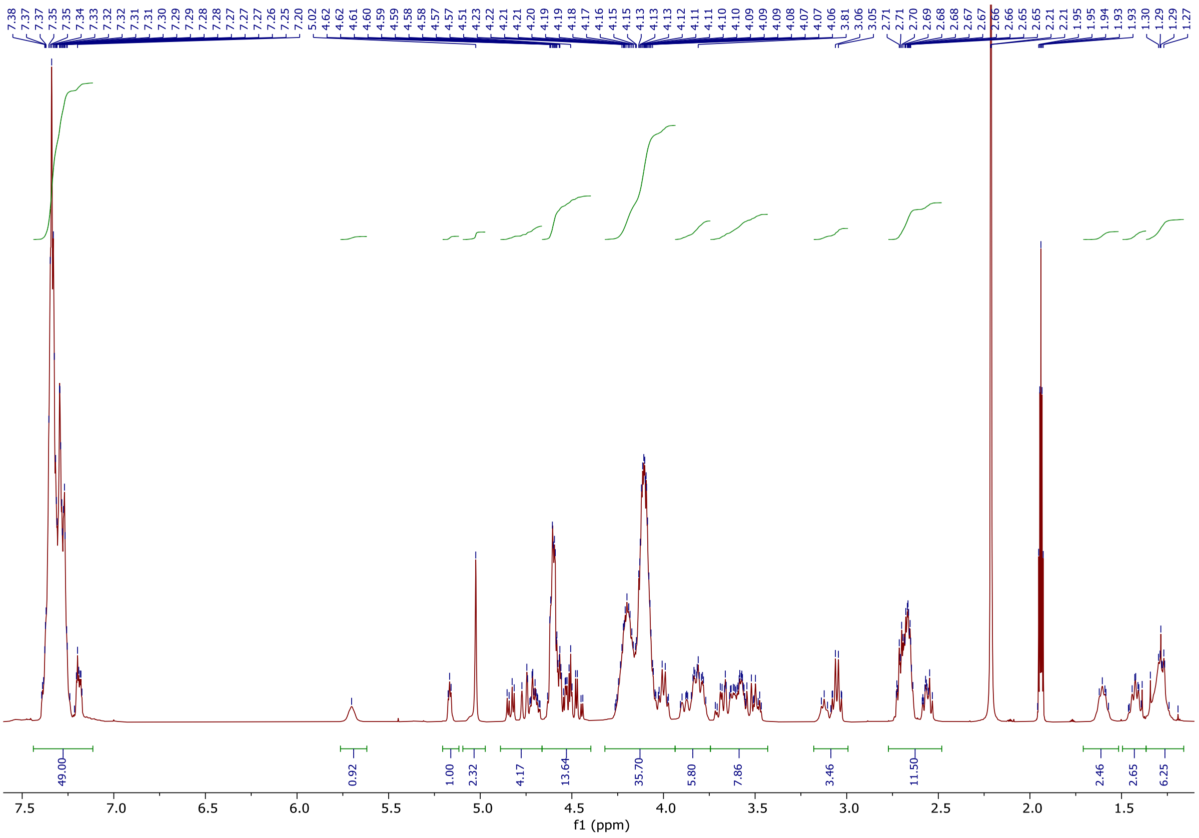

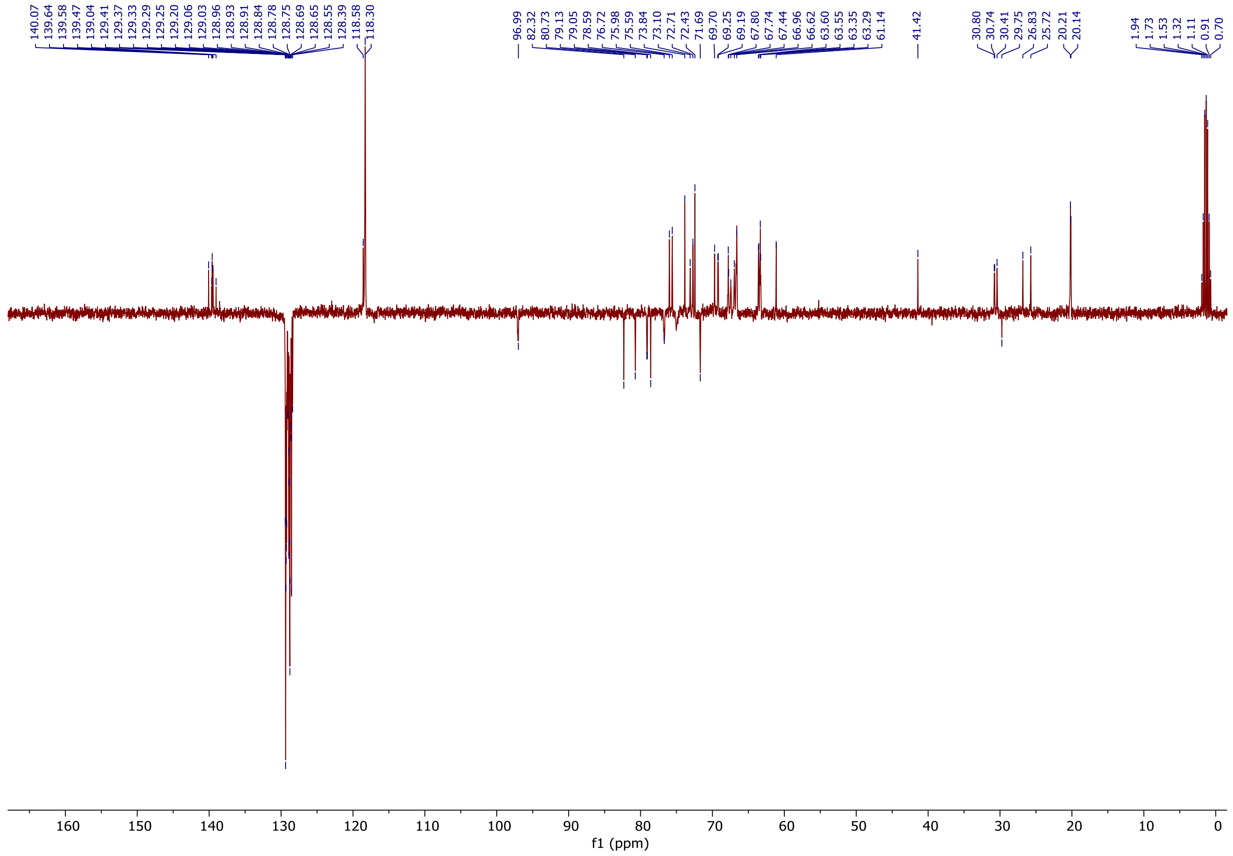
140.07
139.64
139.58
139.47
139.04
129.41
129.37
129.33
129.29
129.25
129.20
129.06
129.03
128.96
128.93
128.91
128.84
128.78
128.75
128.69
128.65
128.55
128.39
118.58
118.30
96.99
82.32
80.73
79.13
79.05
78.59
76.72
75.98
75.59
73.84
73.10
72.71
72.43
71.69
69.70
69.25
69.19
67.80
67.74
67.44
66.96
66.62
63.60
63.55
63.35
63.29
61.14
41.42
30.80
30.74
30.41
29.75
26.83
25.72
20.21
20.14
1.94
1.73
1.53
1.32
1.11
0.91
0.70
160
150
140
130
120
110
100
90
80
70
60
50
40
30
20
10
0
f1 (ppm)

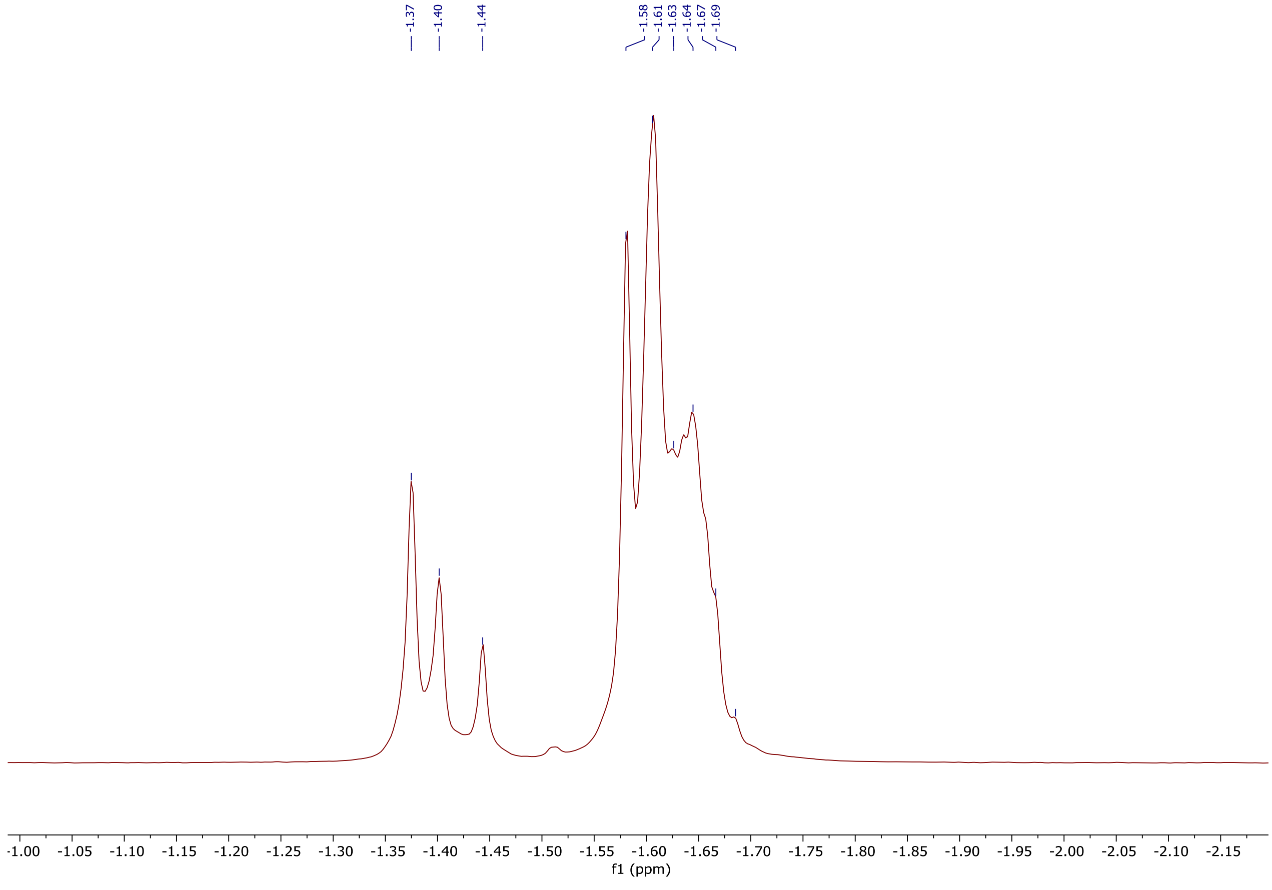
-1.37
-1.40
-1.44
-1.58
-1.61
-1.63
-1.64
-1.67
-1.69
-1.00
-1.05
-1.10
-1.15
-1.20
-1.25
-1.30
-1.35
-1.40
-1.45
-1.50
-1.55
-1.60
-1.65
-1.70
-1.75
-1.80
-1.85
-1.90
-1.95
-2.00
-2.05
-2.10
-2.15
f1 (ppm)

(Protected) (GlcGroP)(GroP)-Spacer or Dimer S17

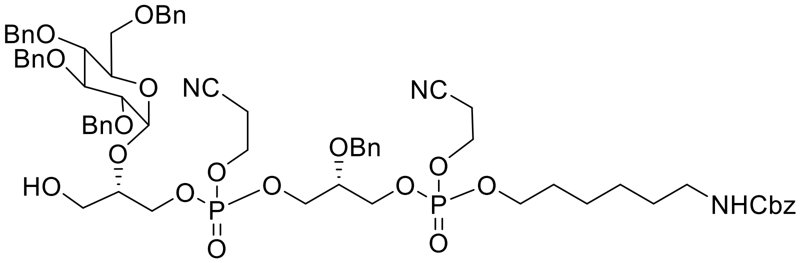

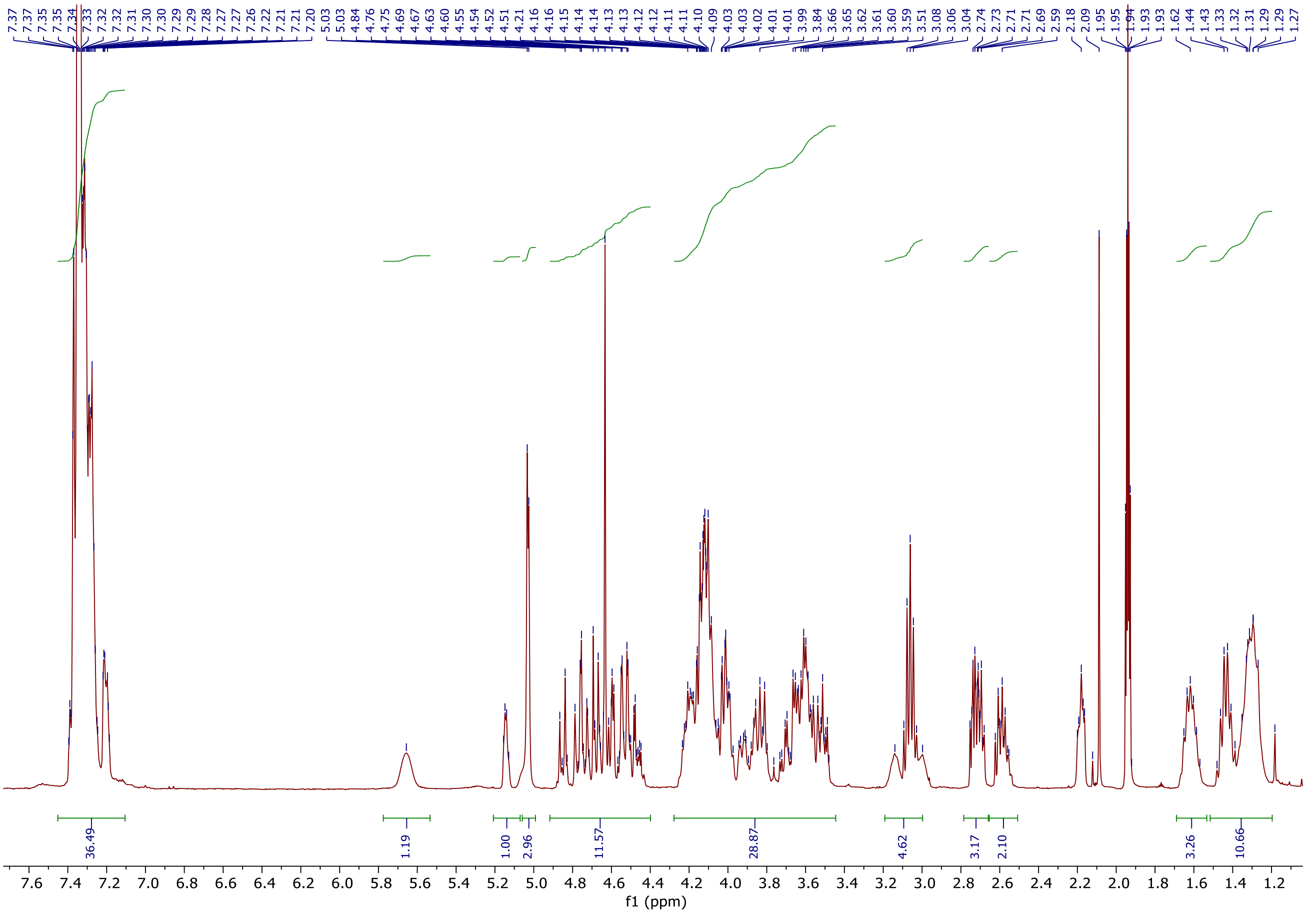

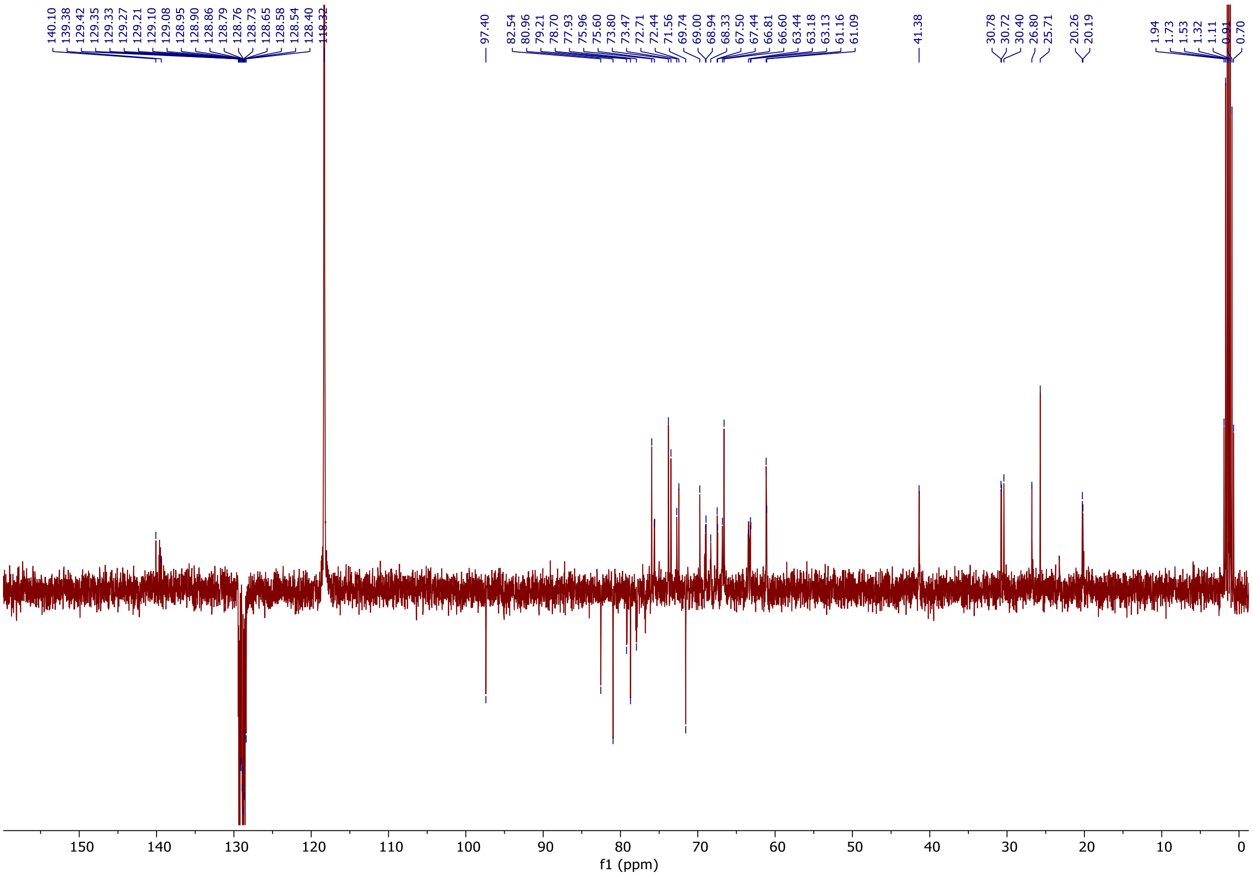
140.10
139.38
129.42
129.35
129.33
129.27
129.21
129.10
129.08
128.95
128.90
128.86
128.79
128.76
128.73
128.65
128.58
128.54
128.40
118.32
97.40
82.54
80.96
79.21
78.70
77.93
75.96
75.60
73.80
73.47
72.71
72.44
71.56
69.74
69.00
68.94
68.33
67.50
67.44
66.81
66.60
63.44
63.18
63.13
61.16
61.09
41.38
30.78
30.72
30.40
26.80
25.71
20.26
20.19
1.94
1.73
1.53
1.32
1.11
0.91
0.70
f1 (ppm)

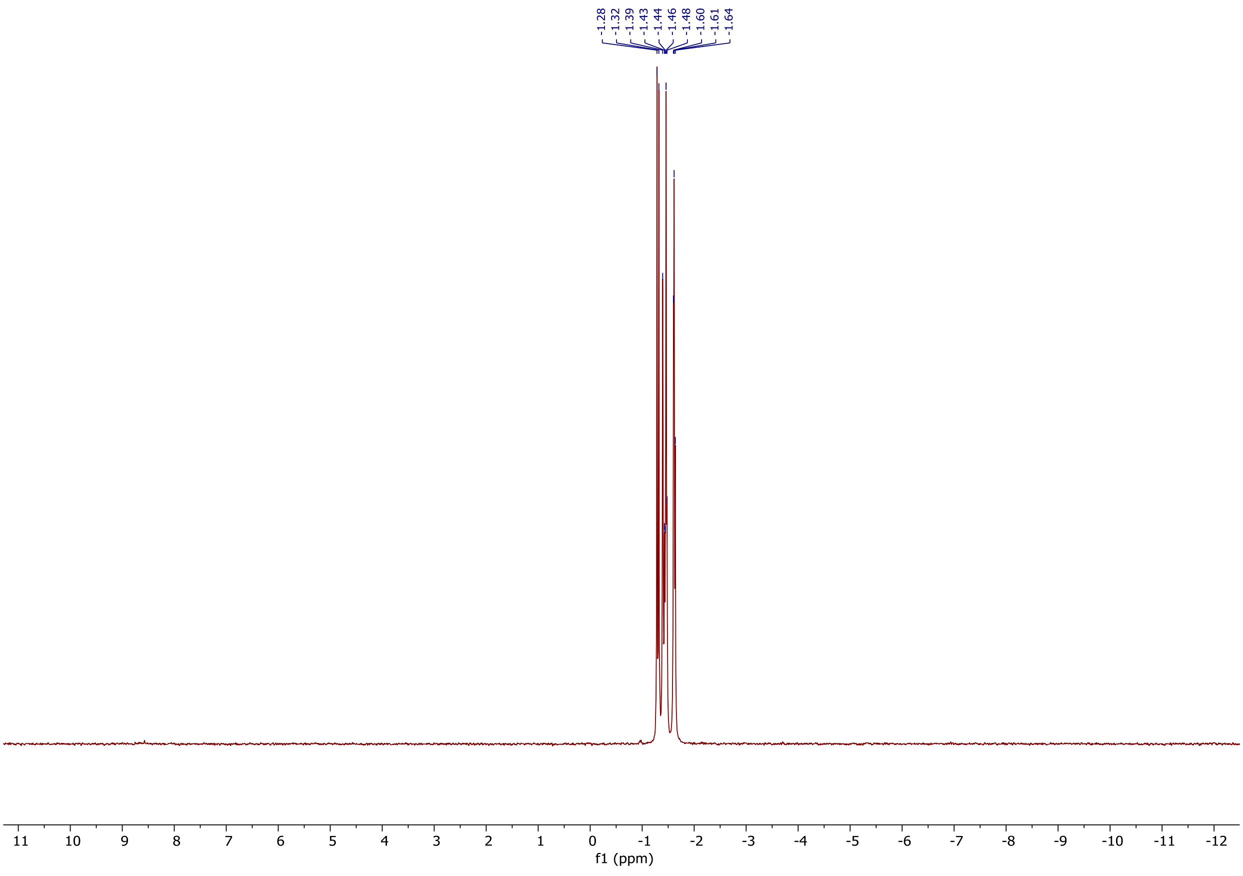
-1.28
-1.32
-1.39
-1.43
-1.44
-1.46
-1.48
-1.60
-1.61
-1.64
f1 (ppm)

(Protected) (GroP)(GlcGroP)(GroP) -Spacer or Trimer S18

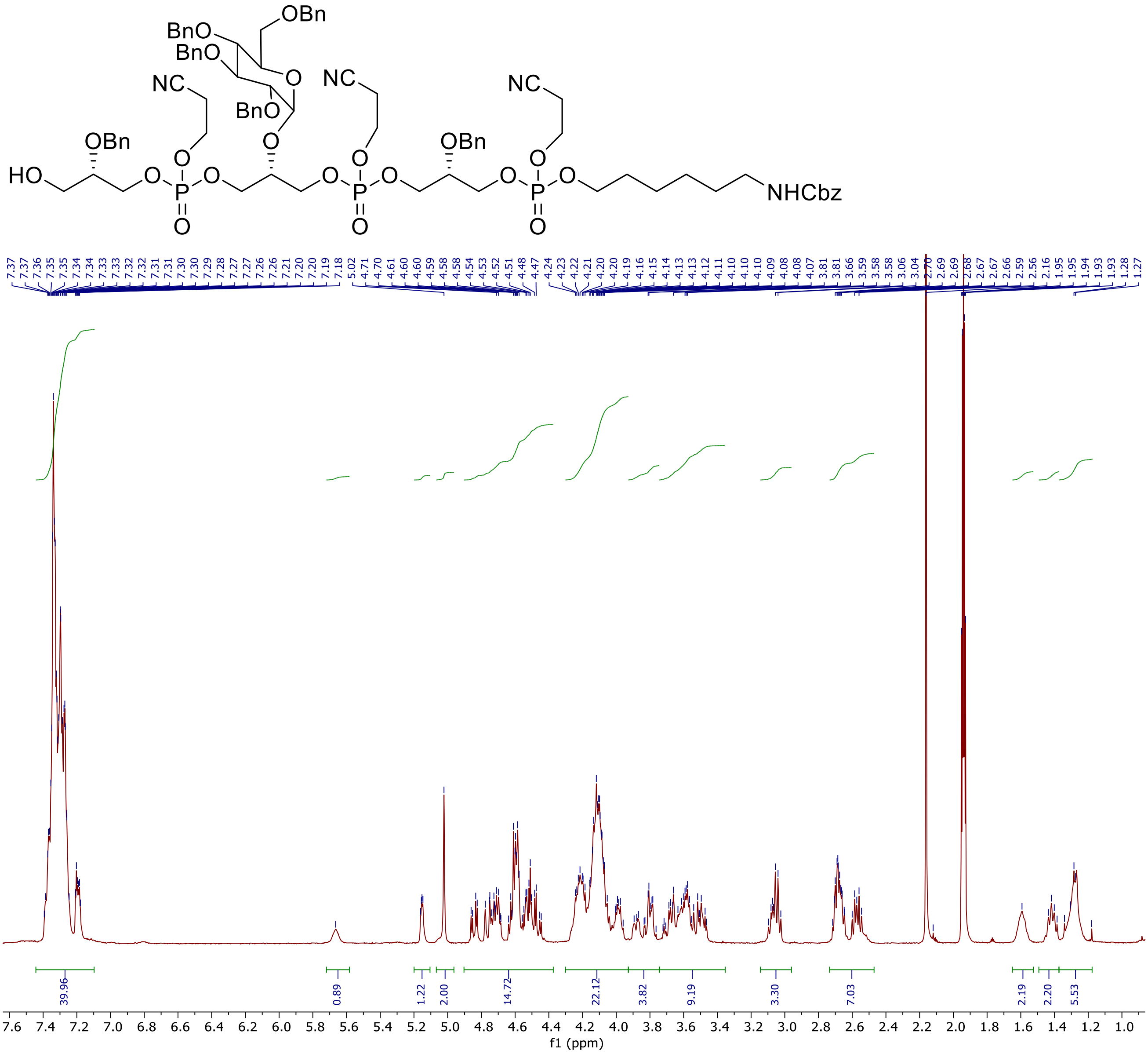

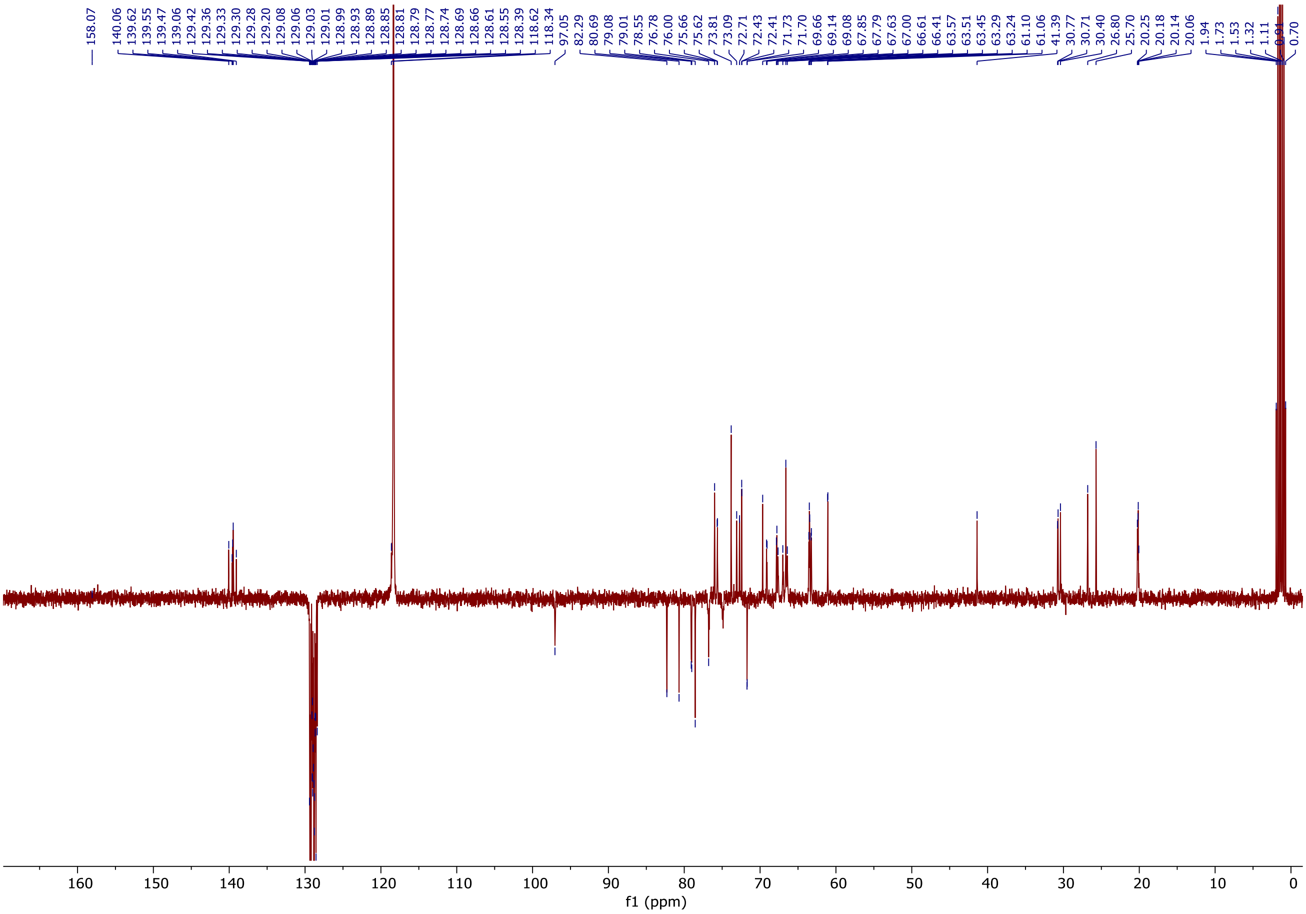
f1 (ppm)

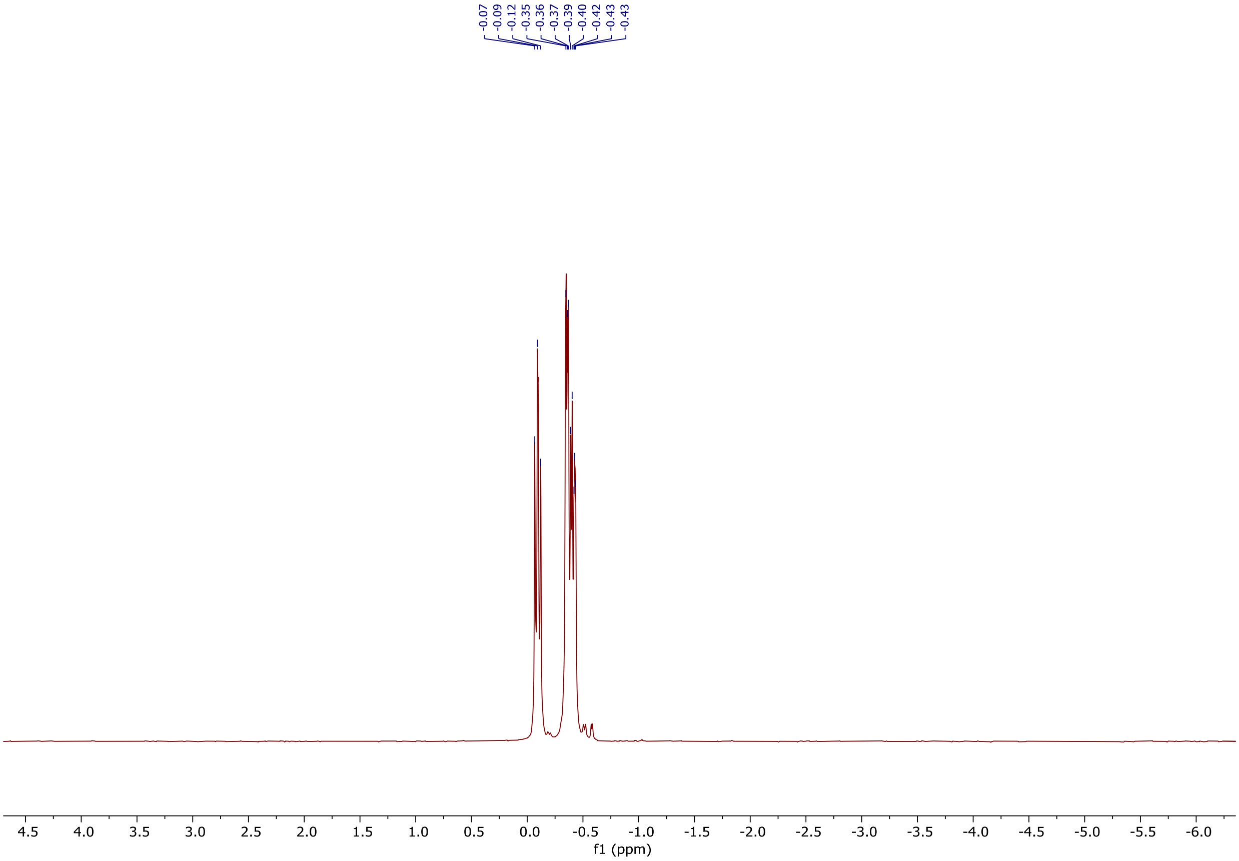
f1 (ppm)

(Protected) (GroP)$_2$(GlcGroP)(GroP) -Spacer or Tetramer S19

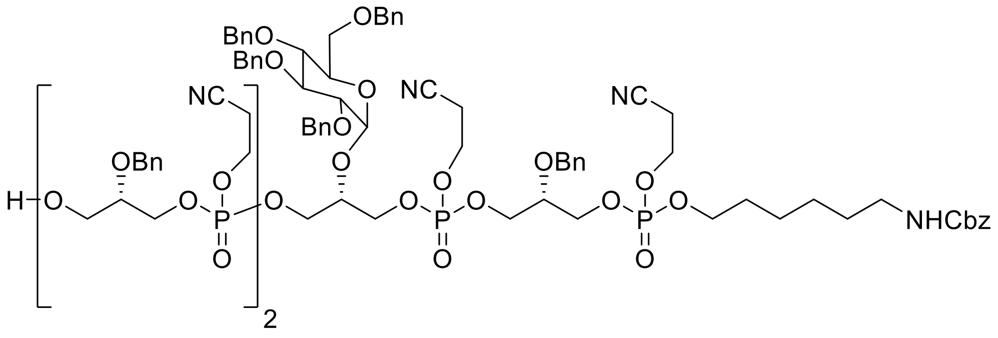

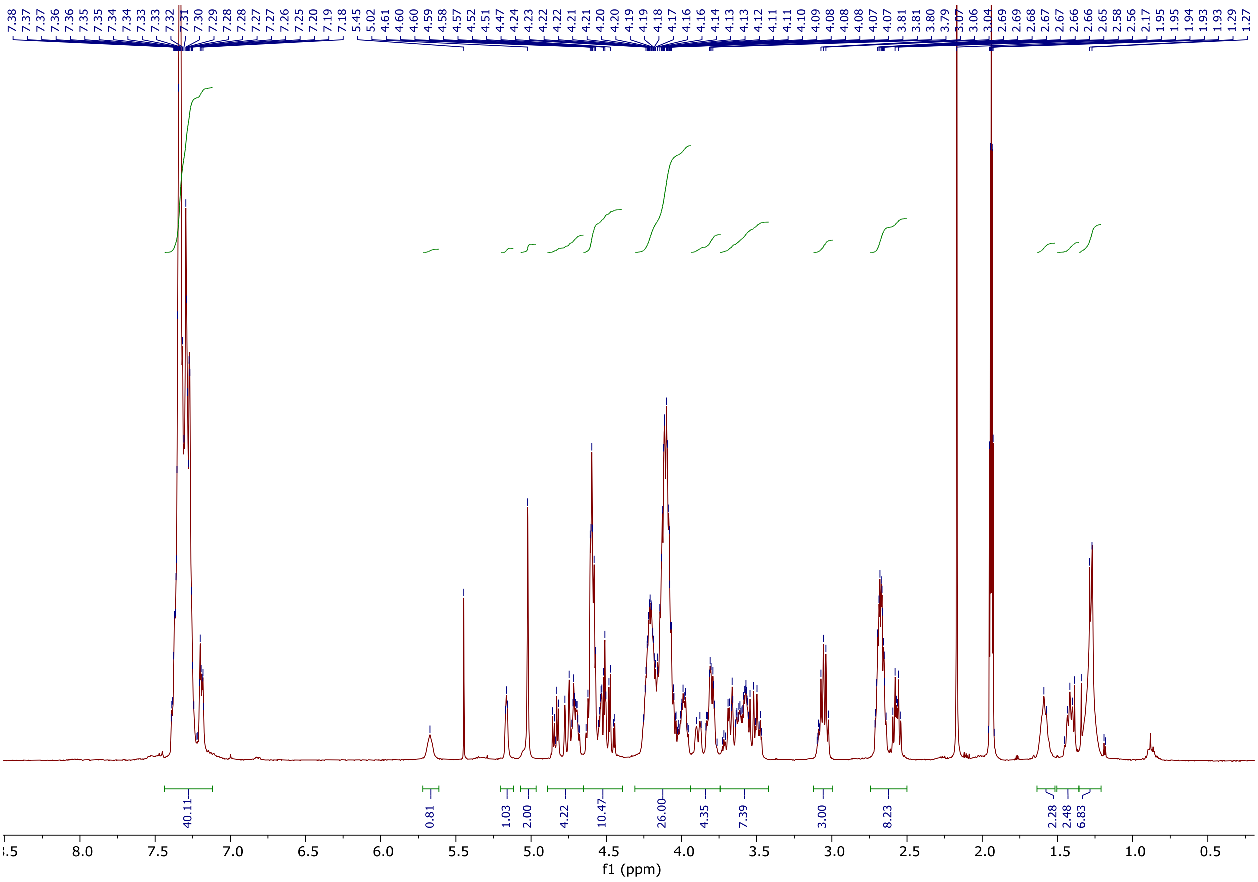

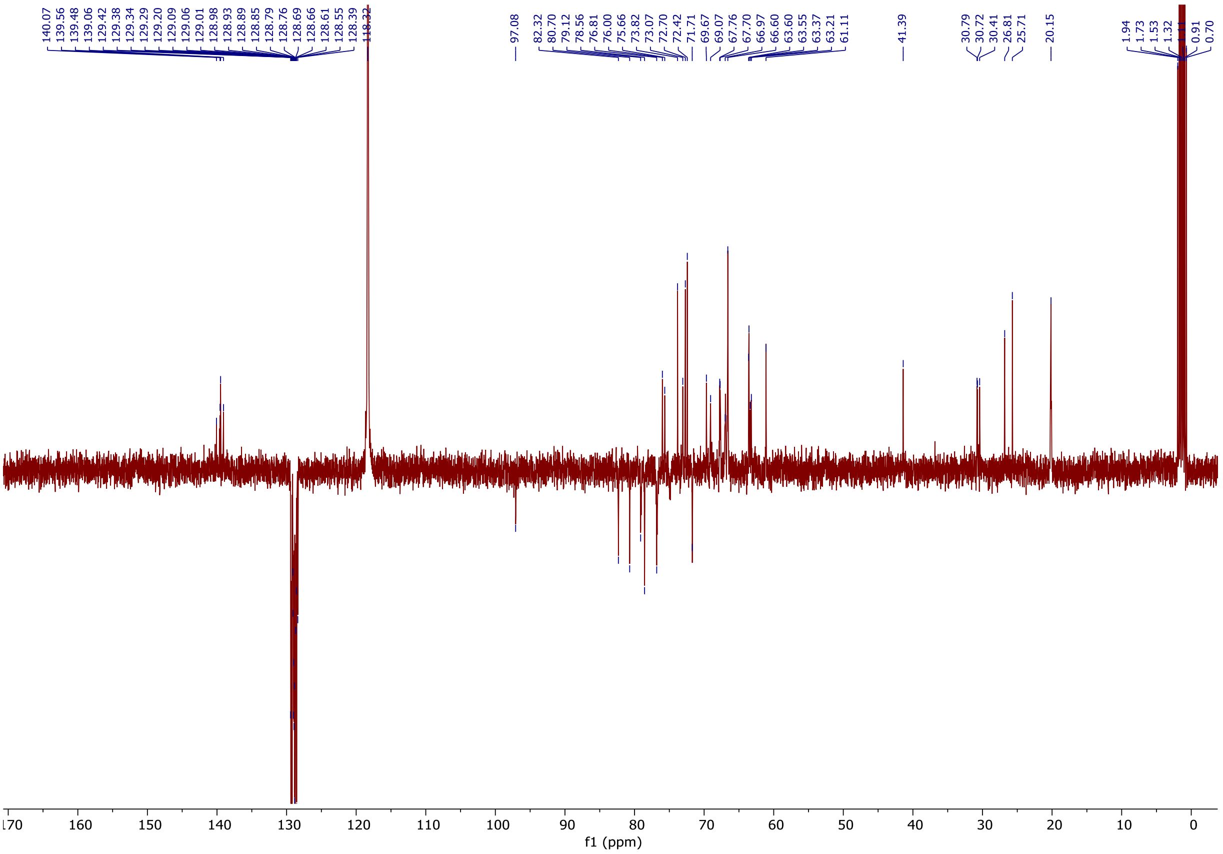
140.07
139.56
139.48
139.06
129.42
129.38
129.34
129.29
129.20
129.09
129.06
129.01
128.98
128.93
128.89
128.85
128.79
128.76
128.69
128.66
128.61
128.55
128.39
118.32
97.08
82.32
80.70
79.12
78.56
76.81
76.00
75.66
73.82
73.07
72.70
72.42
71.71
69.67
69.07
67.76
67.70
66.97
66.60
63.60
63.55
63.37
63.21
61.11
41.39
30.79
30.72
30.41
26.81
25.71
20.15
1.94
1.73
1.53
1.32
1.11
0.91
0.70
f1 (ppm)

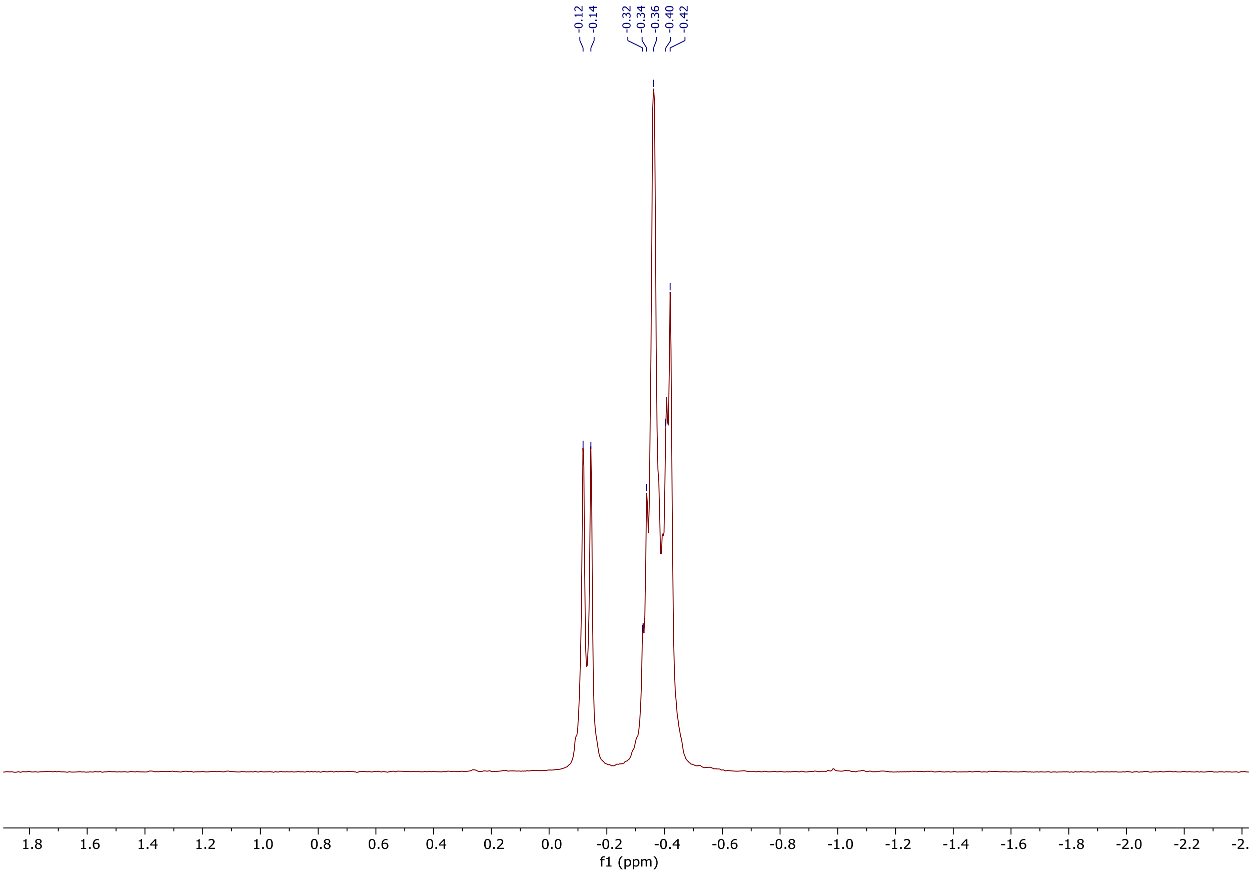
-0.12
-0.14
-0.32
-0.34
-0.36
-0.40
-0.42
f1 (ppm)

(Protected) (GroP)$_3$(GlcGroP)(GroP) -Spacer or Pentamer S20

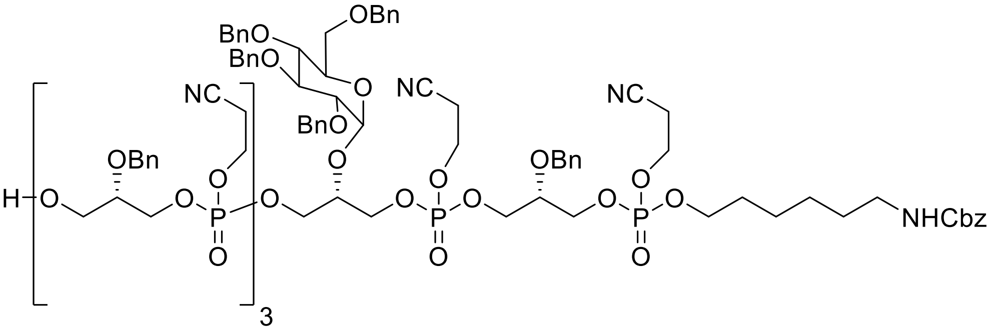

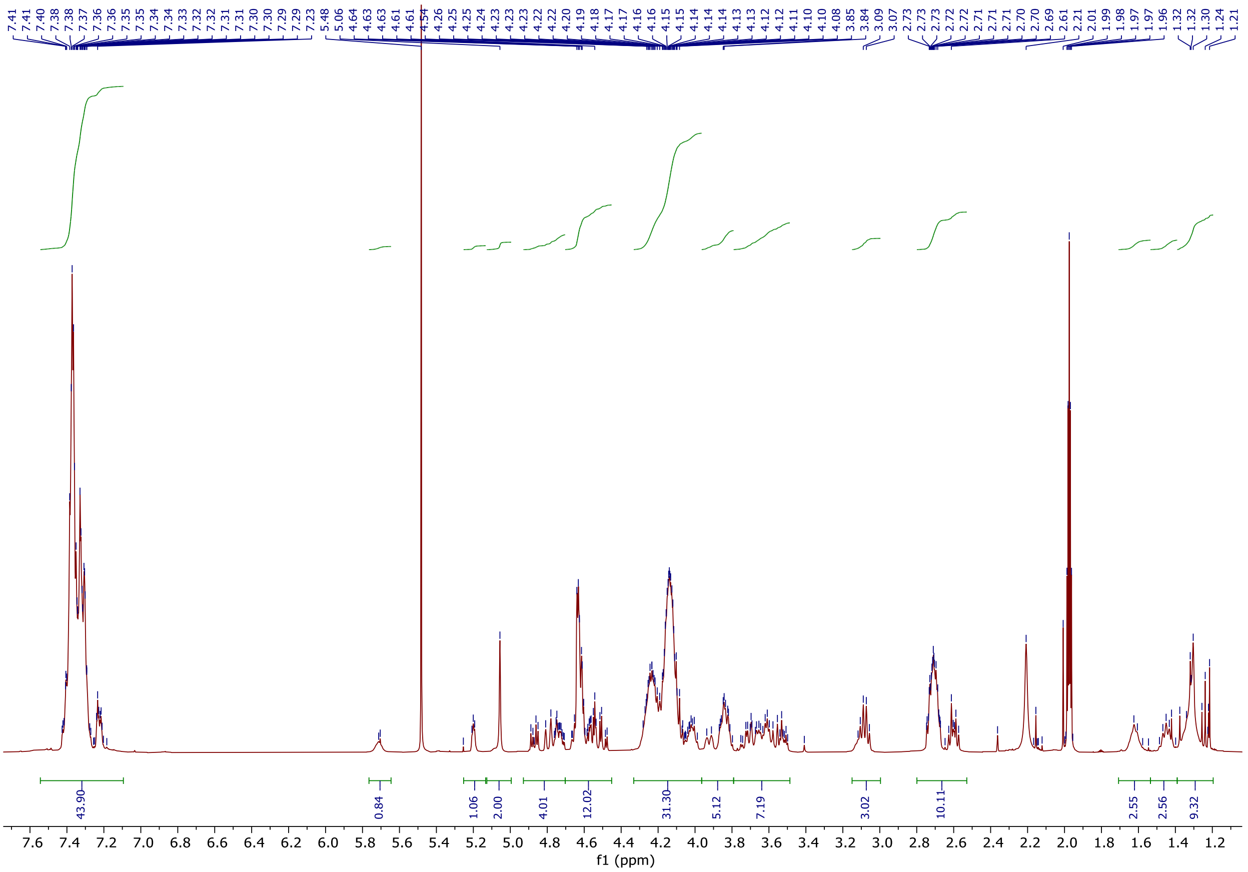

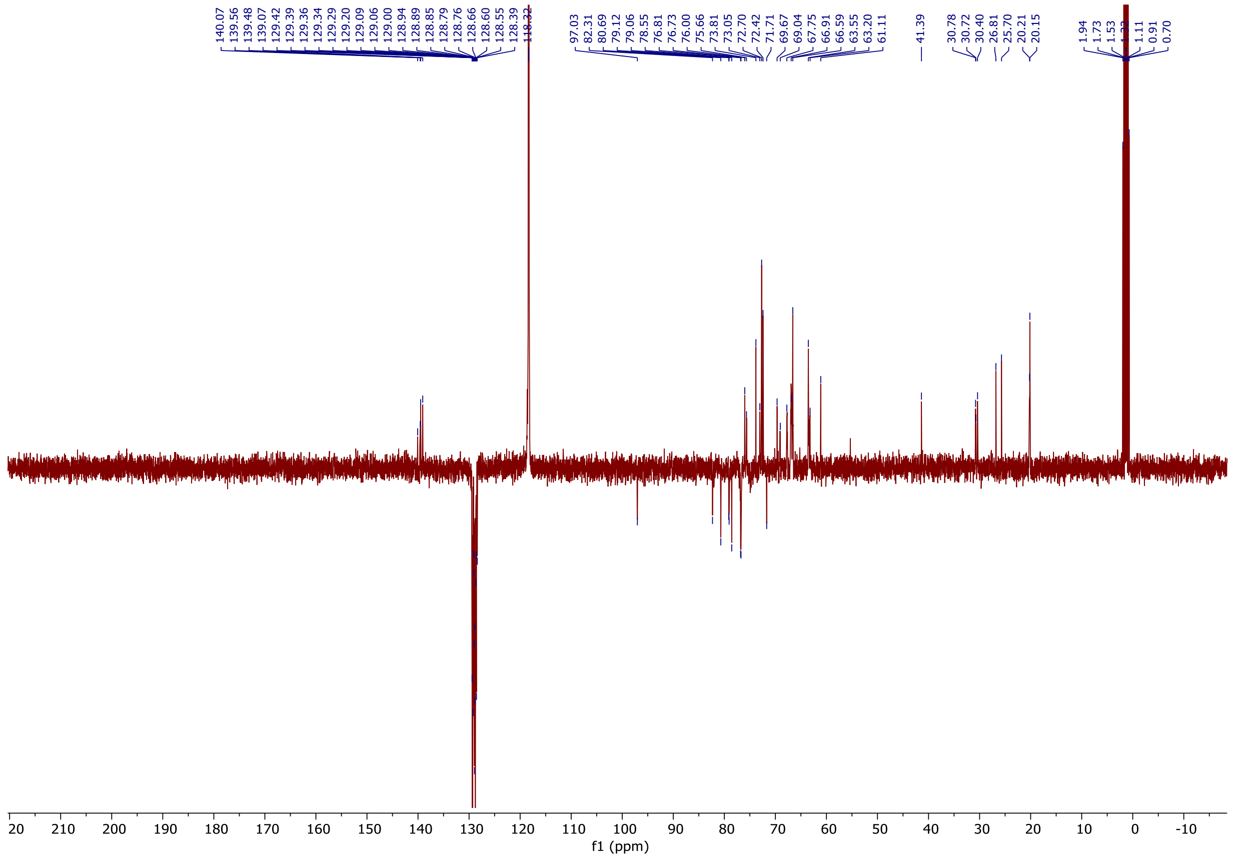
140.07 139.56 139.48 139.07 129.42 129.39 129.36 129.34 129.29 129.20 129.09 129.06 129.00 128.94 128.89 128.85 128.79 128.76 128.66 128.60 128.55 128.39 118.22
97.03 82.31 80.69 79.12 79.06 78.55 76.81 76.73 76.00 75.66 73.81 73.05 72.70 72.42 71.71 69.67 69.04 67.75 66.91 66.59 63.55 63.20 61.11
41.39
30.78 30.72 30.40 26.81 25.70 20.21 20.15
1.94 1.73 1.53 1.11 0.91 0.70
f1 (ppm)

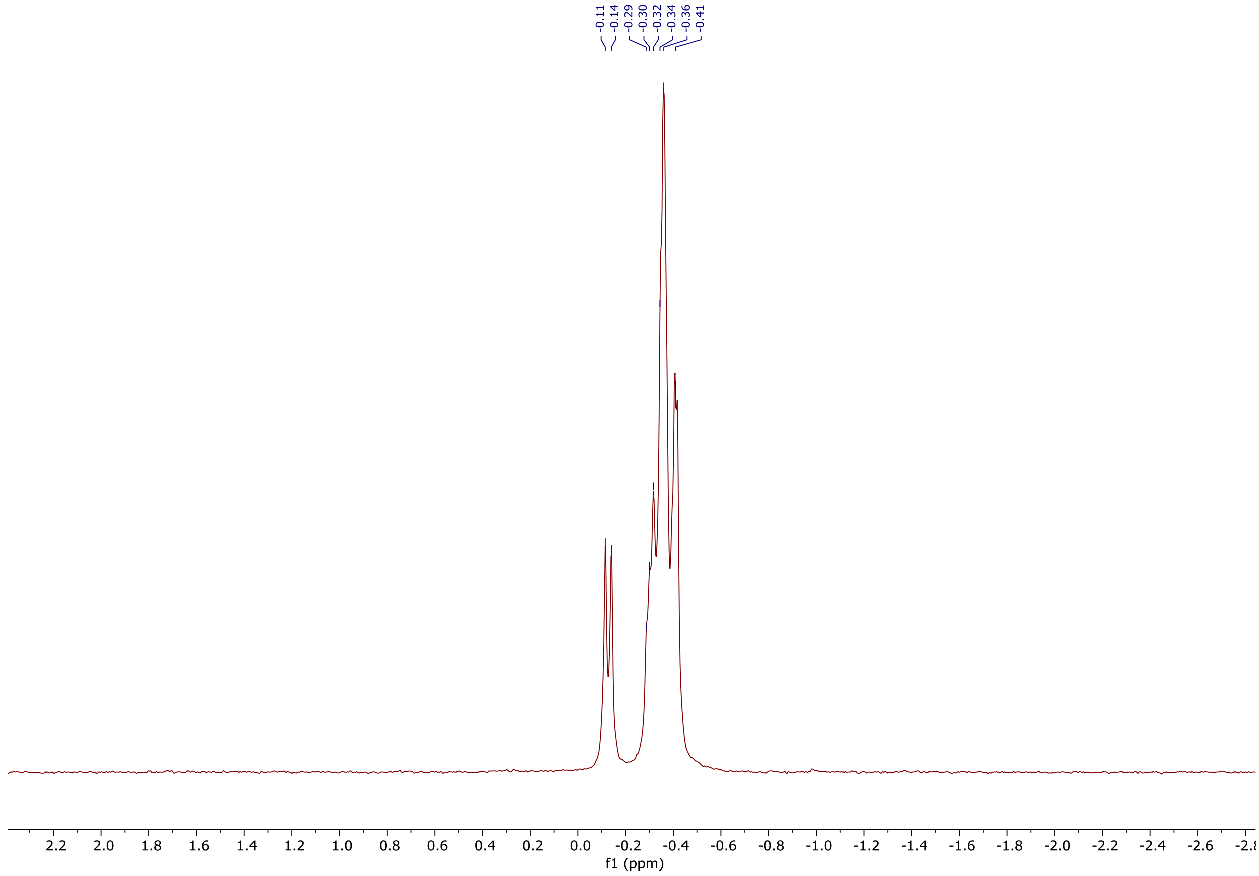
-0.11 -0.14 -0.29 -0.30 -0.32 -0.34 -0.36 -0.41
f1 (ppm)

(Protected) (GroP)$_4$(GlcGroP)(GroP) -Spacer or Hexamer 27

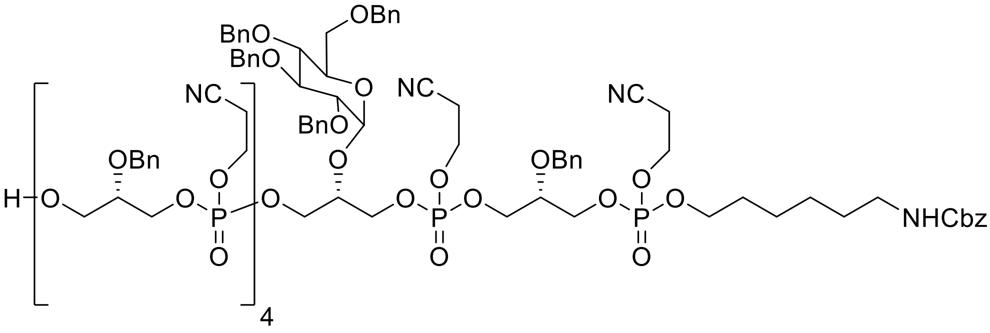

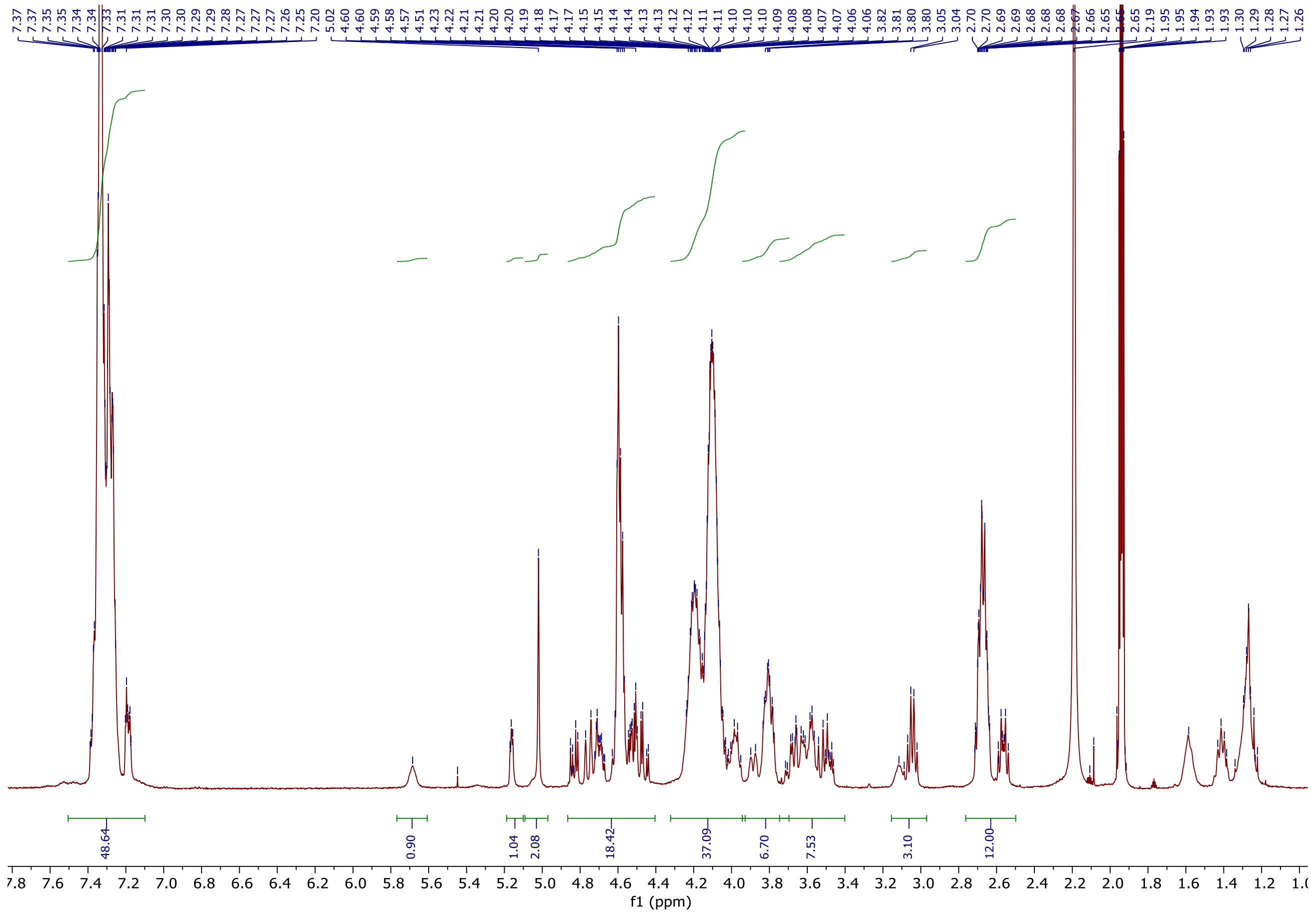

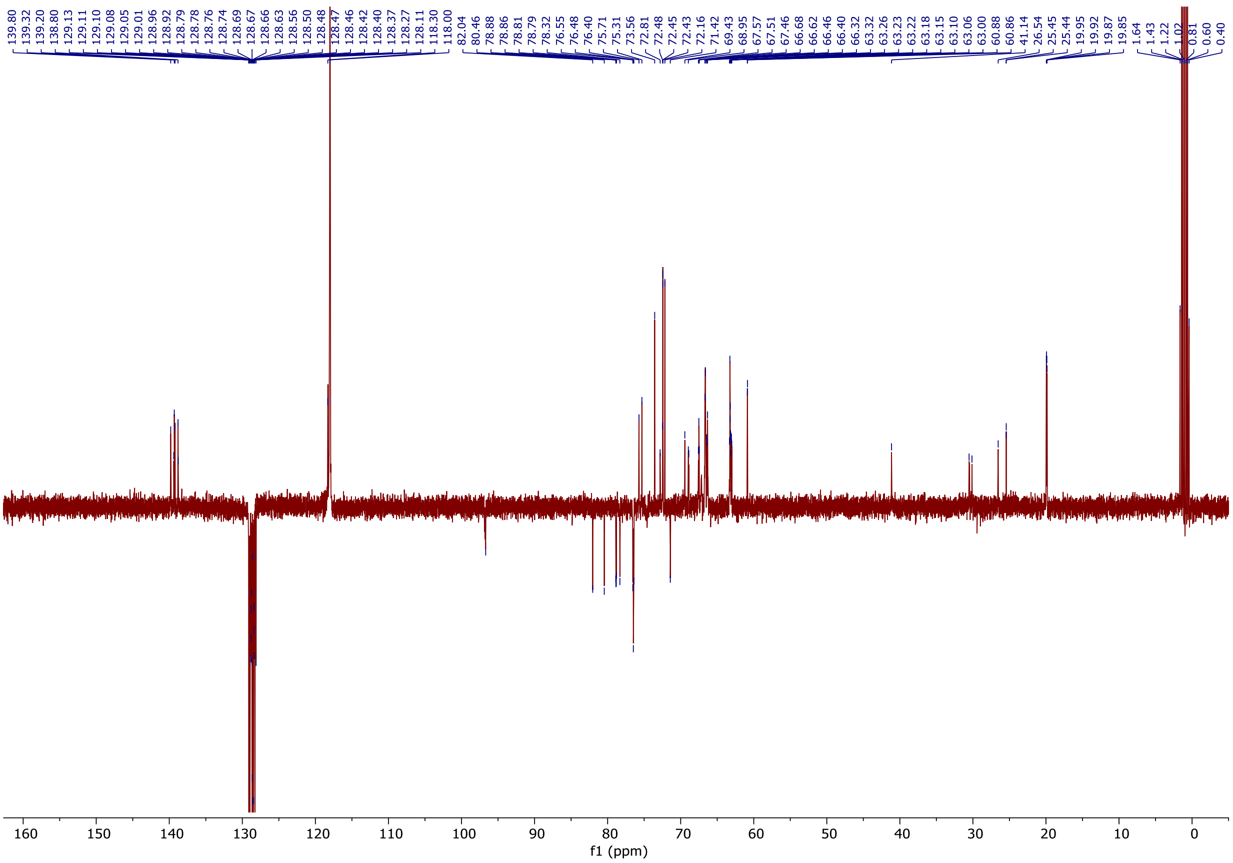
139.80 139.32 139.20 138.80 129.13 129.11 129.10 129.08 129.05 129.01 128.96 128.92 128.79 128.78 128.76 128.74 128.69 128.67 128.66 128.63 128.56 128.50 128.48 128.47 128.46 128.42 128.40 128.37 128.27 128.11 118.30 118.00
82.04 80.46 78.88 78.86 78.81 78.79 78.32 76.55 76.48 76.40 75.71 75.31 73.56 72.81 72.48 72.45 72.43 72.16 71.42 69.43 68.95 67.57 67.51 67.46 66.68 66.62 66.46 66.40 66.32 63.32 63.26 63.23 63.22 63.18 63.15 63.10 63.06 63.00 60.88 60.86 41.14 26.54 25.45 25.44 19.95 19.92 19.87 19.85 1.64 1.43 1.22 1.02 0.81 0.60 0.40
160 150 140 130 120 110 100 90 80 70 60 50 40 30 20 10 0
f1 (ppm)

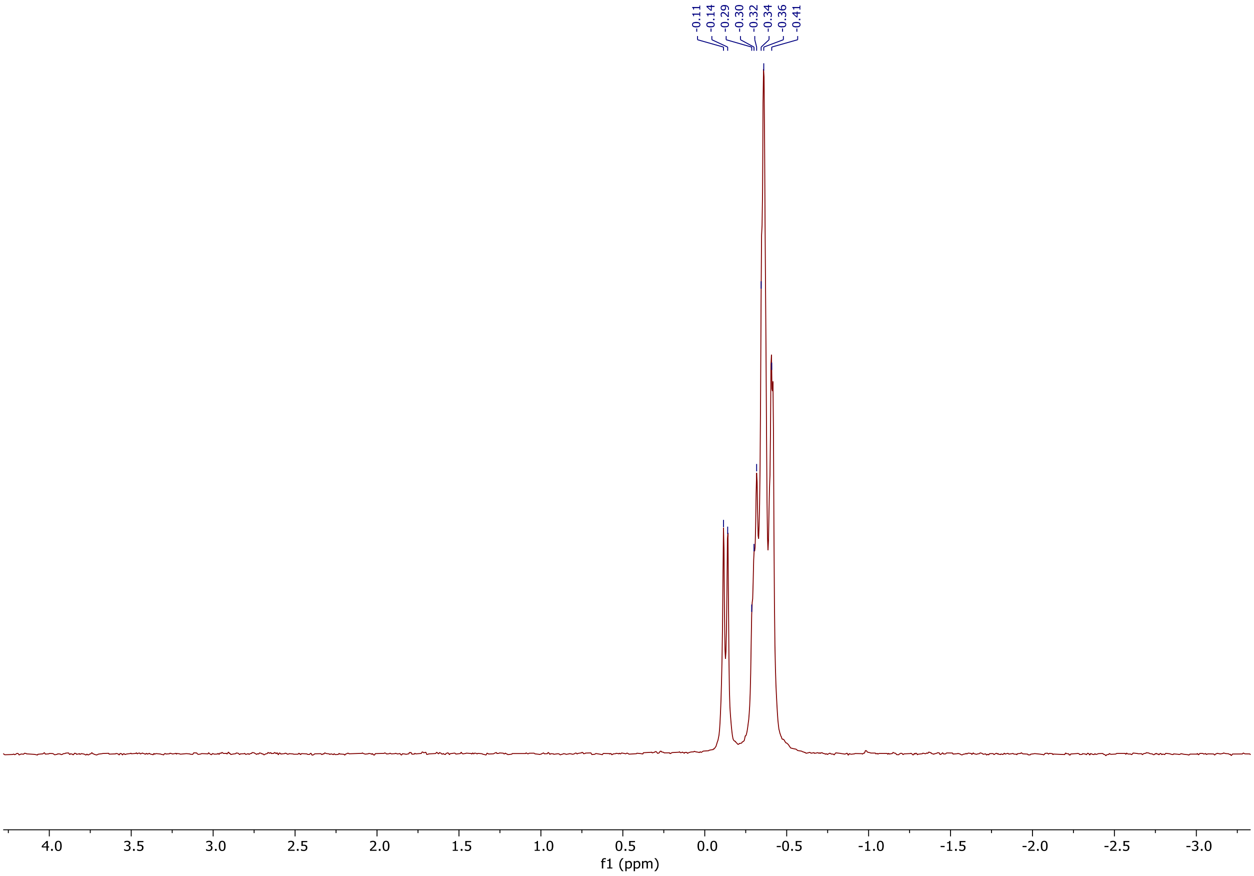
-0.11 -0.14 -0.29 -0.30 -0.32 -0.34 -0.36 -0.41
4.0 3.5 3.0 2.5 2.0 1.5 1.0 0.5 0.0 -0.5 -1.0 -1.5 -2.0 -2.5 -3.0
f1 (ppm)

(Protected) GlcGroP-Spacer or Monomer S21

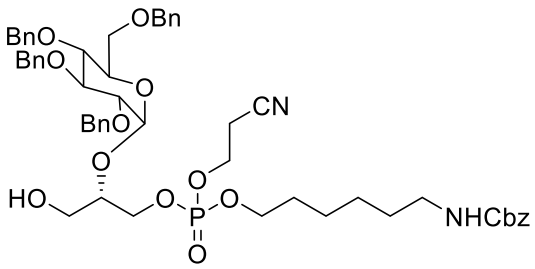

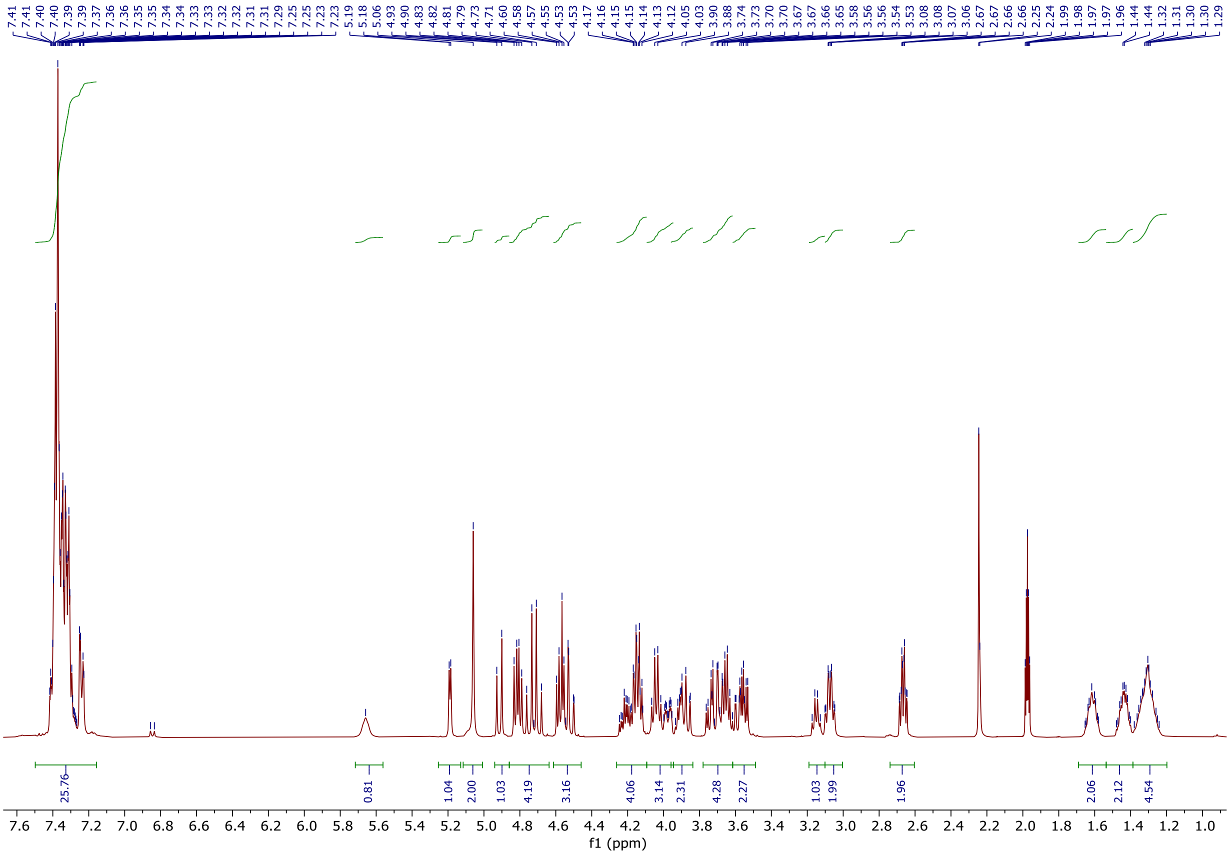

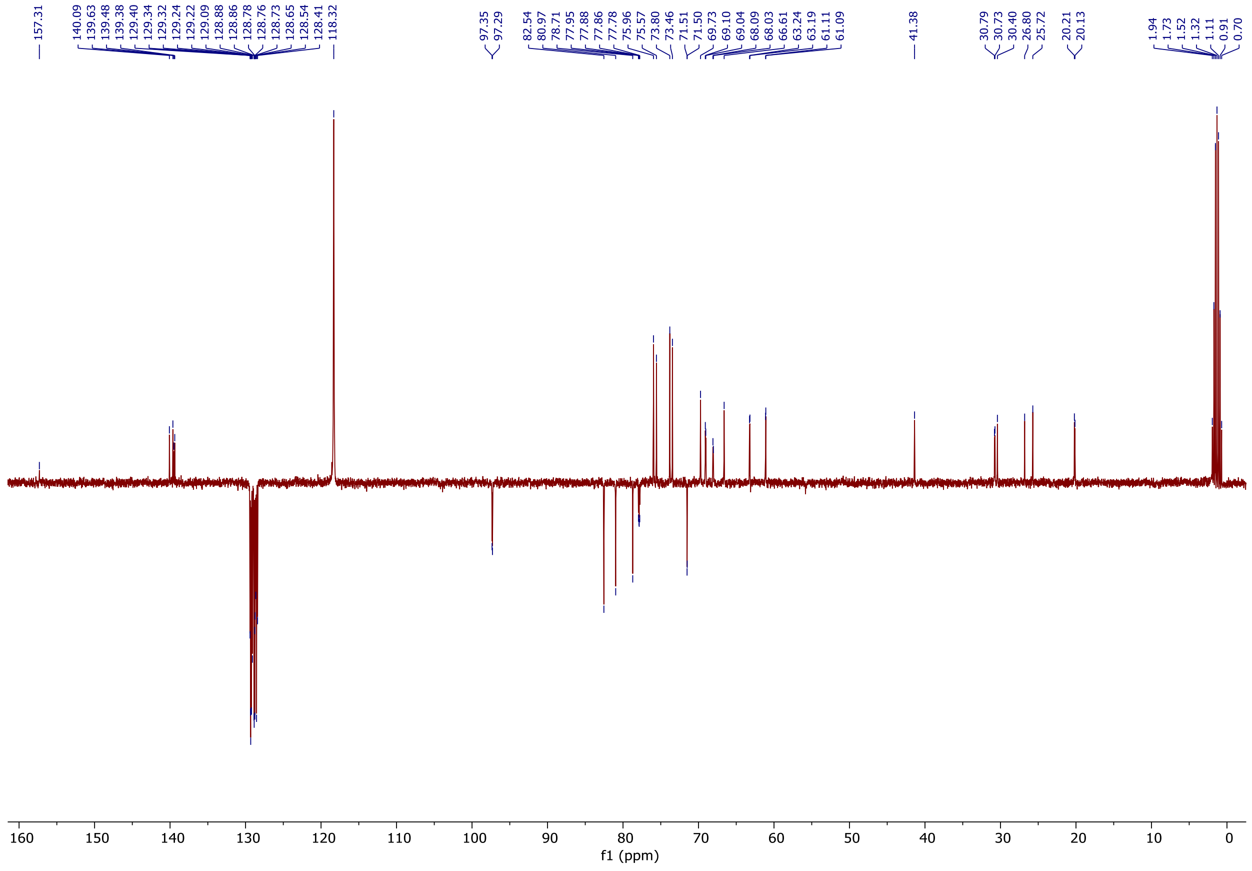
157.31
140.09
139.63
139.48
139.38
129.40
129.34
129.32
129.24
129.22
129.09
128.88
128.86
128.78
128.76
128.73
128.65
128.54
128.41
118.32
97.35
97.29
82.54
80.97
78.71
77.95
77.88
77.86
77.78
75.96
75.57
73.80
73.46
71.51
71.50
69.73
69.10
69.04
68.09
68.03
66.61
63.24
63.19
61.11
61.09
41.38
30.79
30.73
30.40
26.80
25.72
20.21
20.13
1.94
1.73
1.52
1.32
1.11
0.91
0.70
160
150
140
130
120
110
100
90
80
70
60
50
40
30
20
10
0
f1 (ppm)
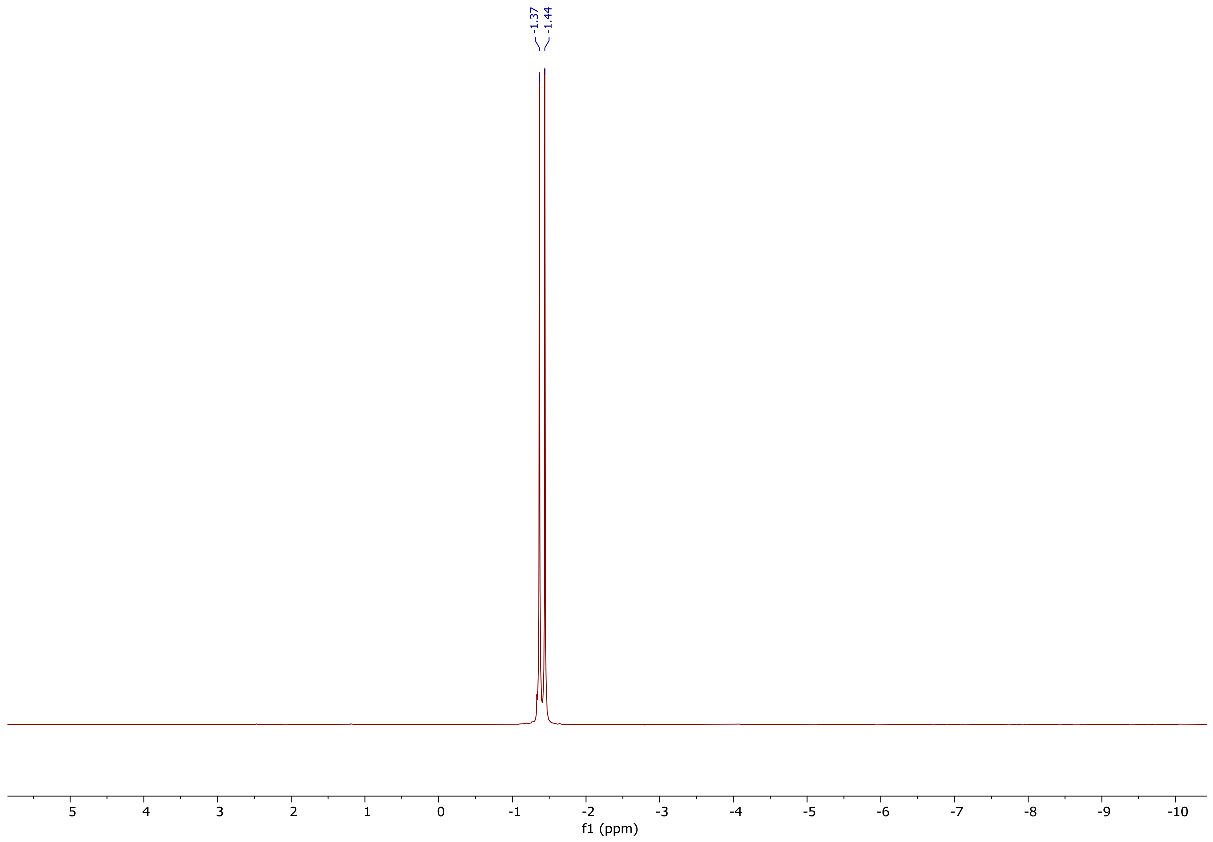
-1.37
-1.44
5
4
3
2
1
0
-1
-2
-3
-4
-5
-6
-7
-8
-9
-10
f1 (ppm)

(Protected) (GroP)(GlcGroP)-Spacer or Dimer S22

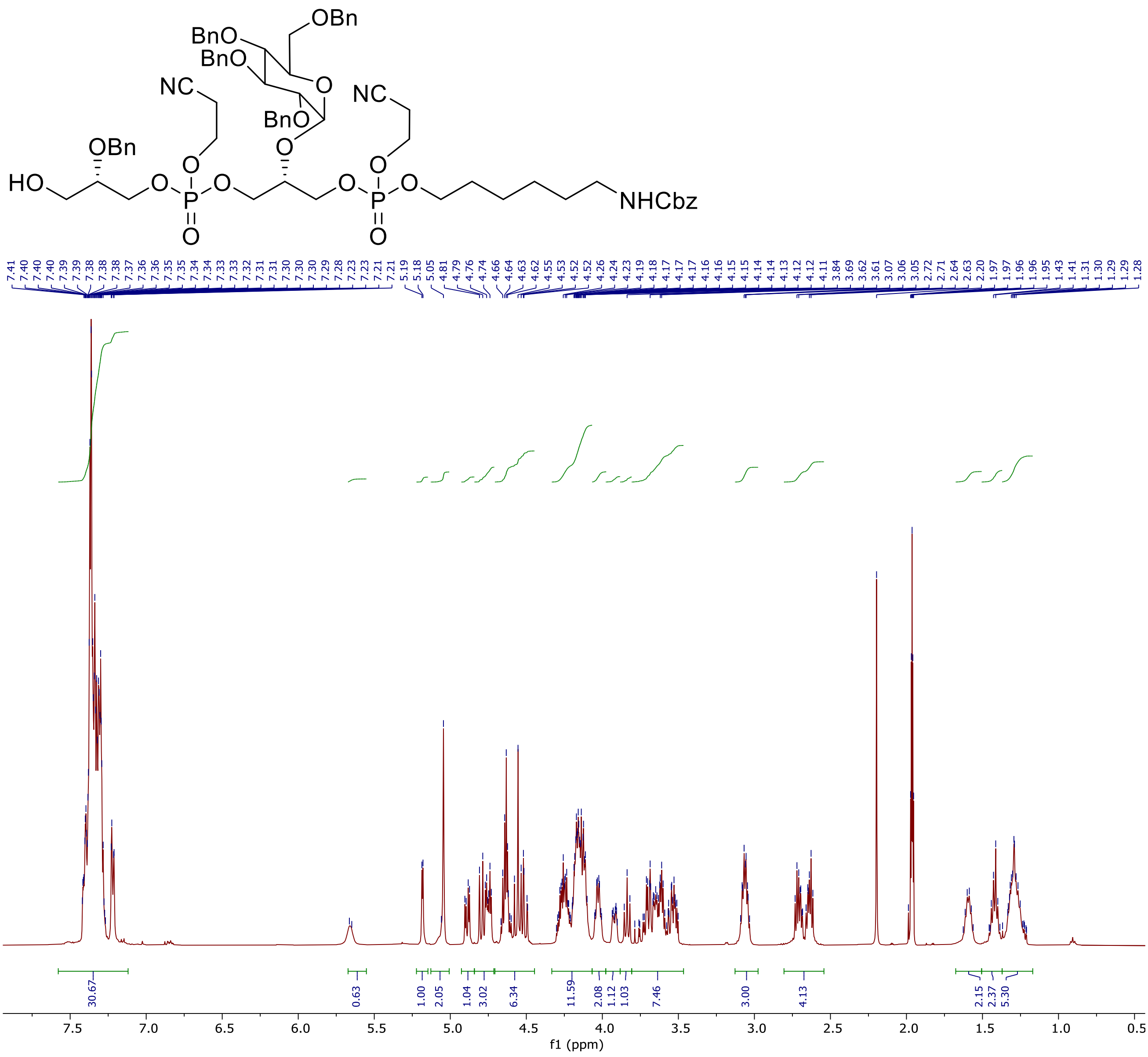

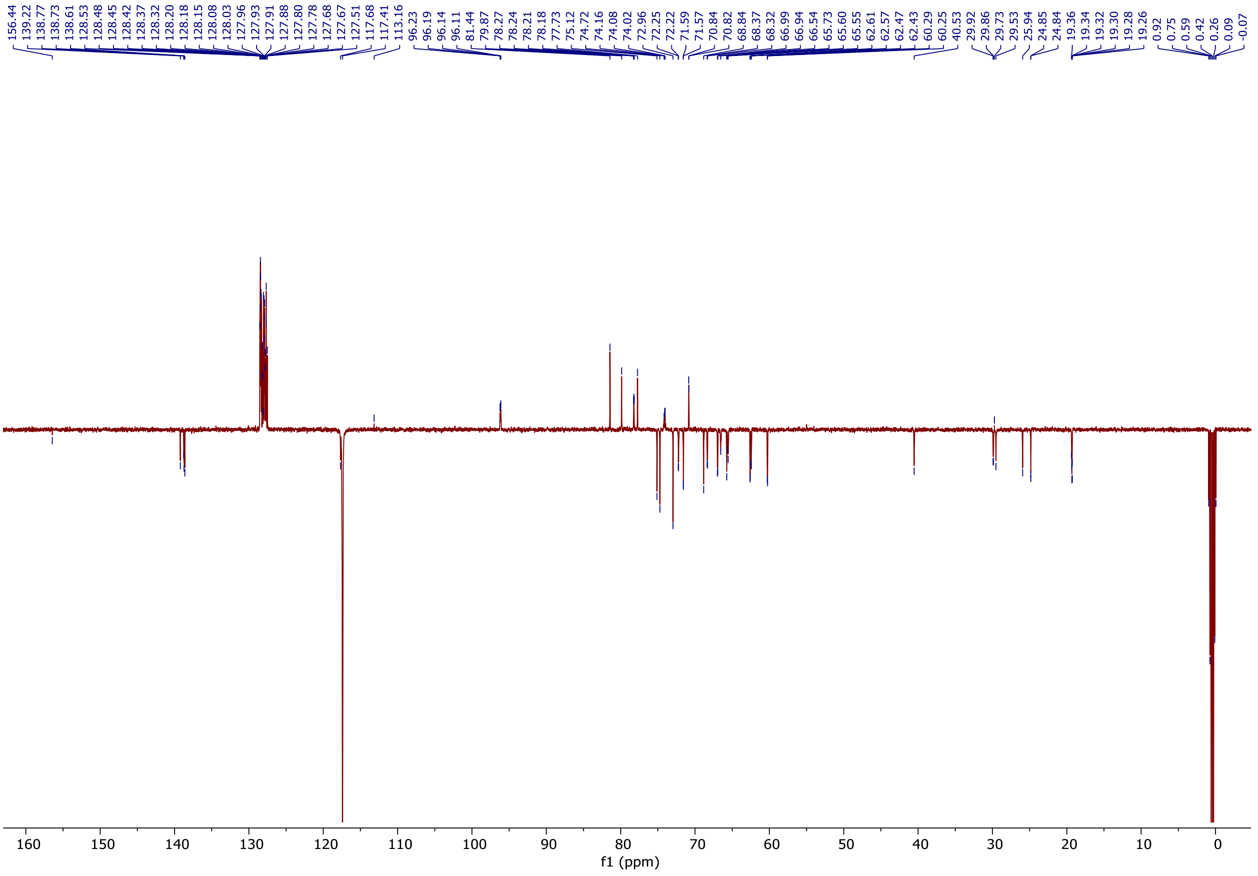
156.44
139.22
138.77
138.73
138.61
128.53
128.48
128.45
128.42
128.37
128.32
128.20
128.18
128.15
128.08
128.03
127.96
127.93
127.91
127.88
127.80
127.78
127.68
127.67
127.51
117.68
117.41
113.16
96.23
96.19
96.14
96.11
81.44
79.87
78.27
78.24
78.21
78.18
77.73
75.12
74.72
74.16
74.08
74.02
72.96
72.25
72.22
71.59
71.57
70.84
70.82
68.84
68.37
68.32
66.99
66.94
66.54
65.73
65.60
65.55
62.61
62.57
62.47
62.43
60.29
60.25
40.53
29.92
29.86
29.73
29.53
25.94
24.85
24.84
19.36
19.34
19.32
19.30
19.28
19.26
0.92
0.75
0.59
0.42
0.26
0.09
-0.07
f1 (ppm)

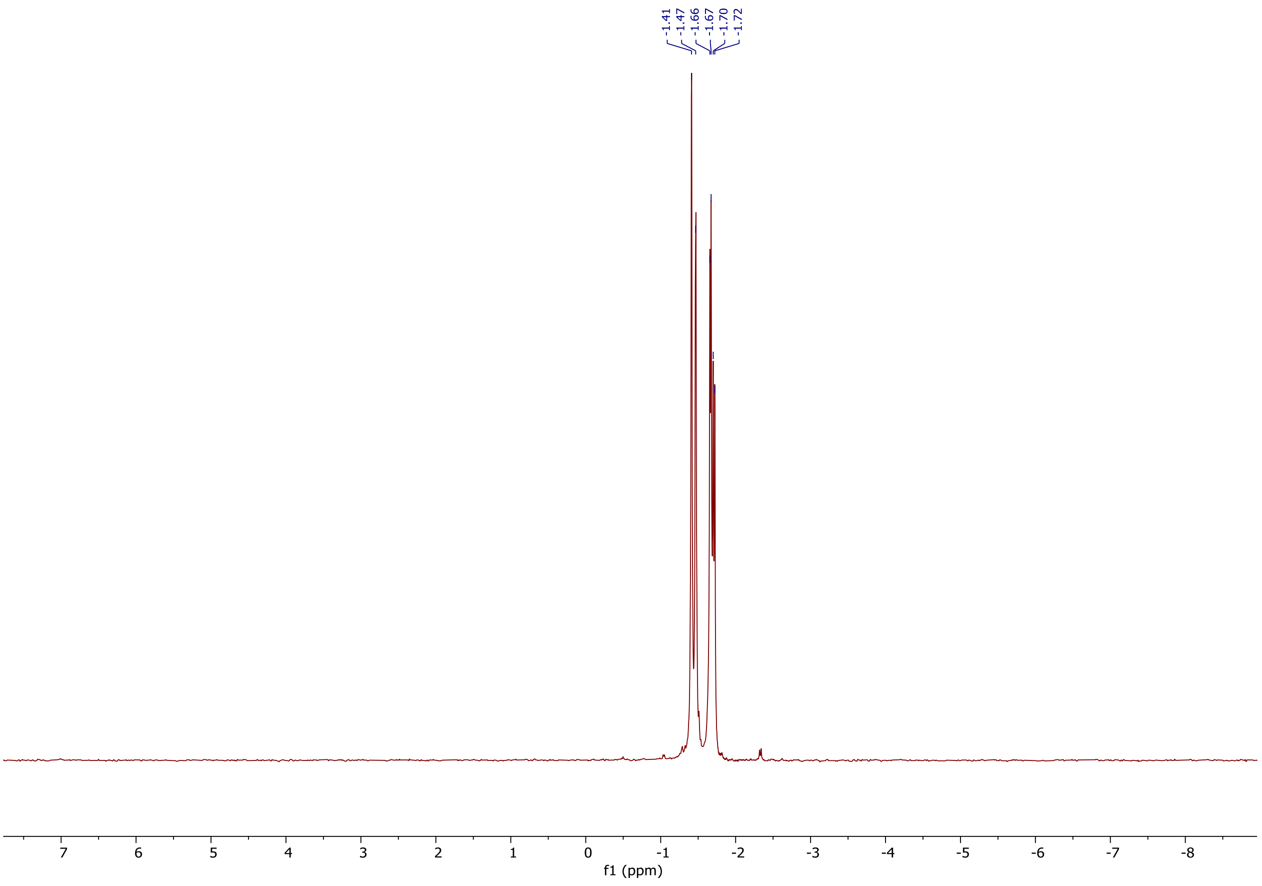
-1.41
-1.47
-1.66
-1.67
-1.70
-1.72
f1 (ppm)

(Protected) (GroP)$_2$(GlcGroP)-Spacer or Trimer S23

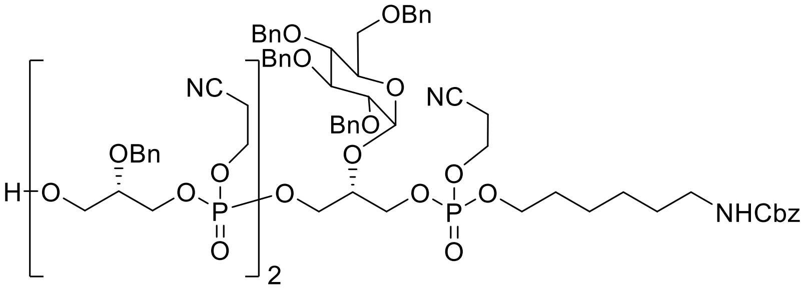

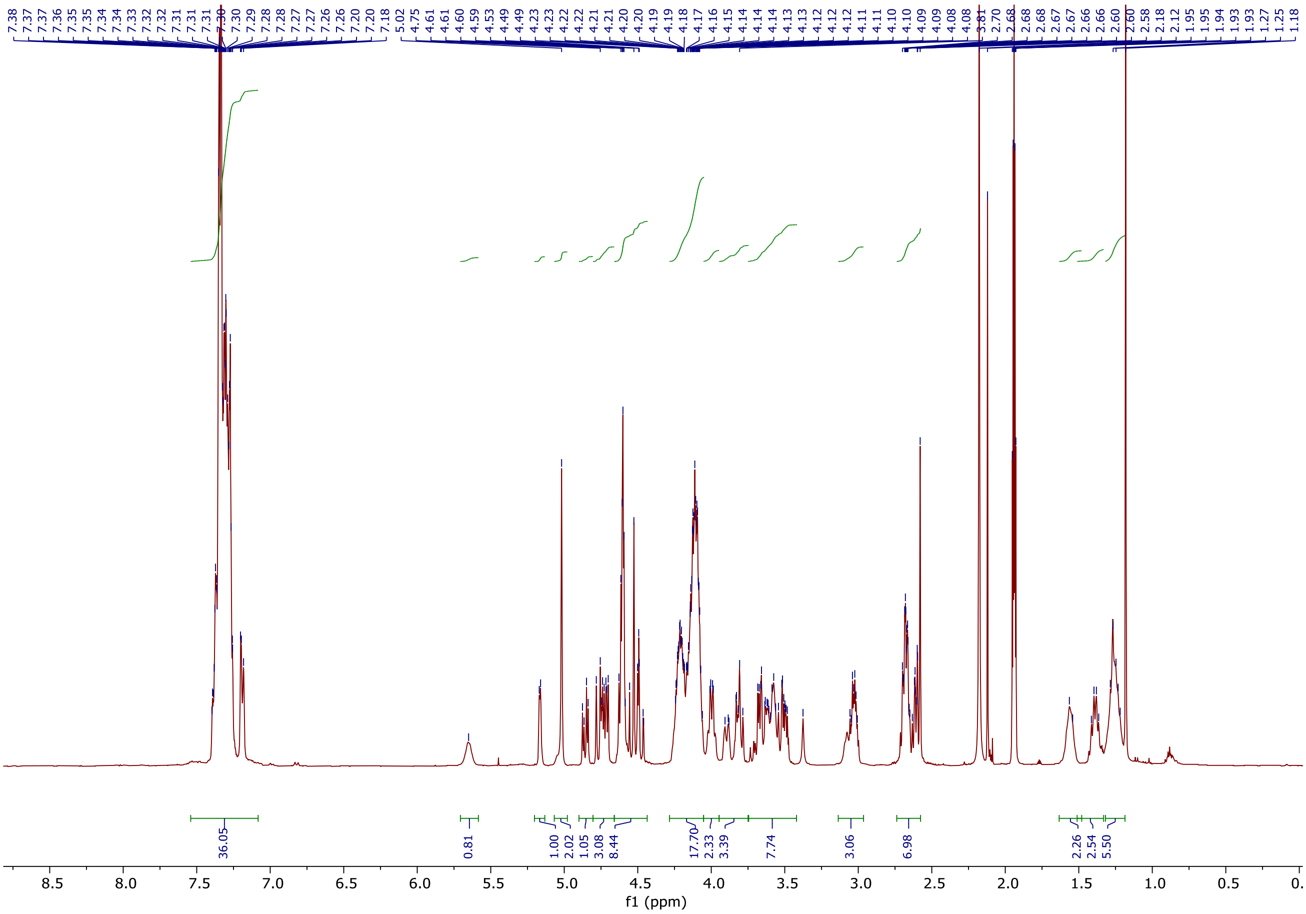

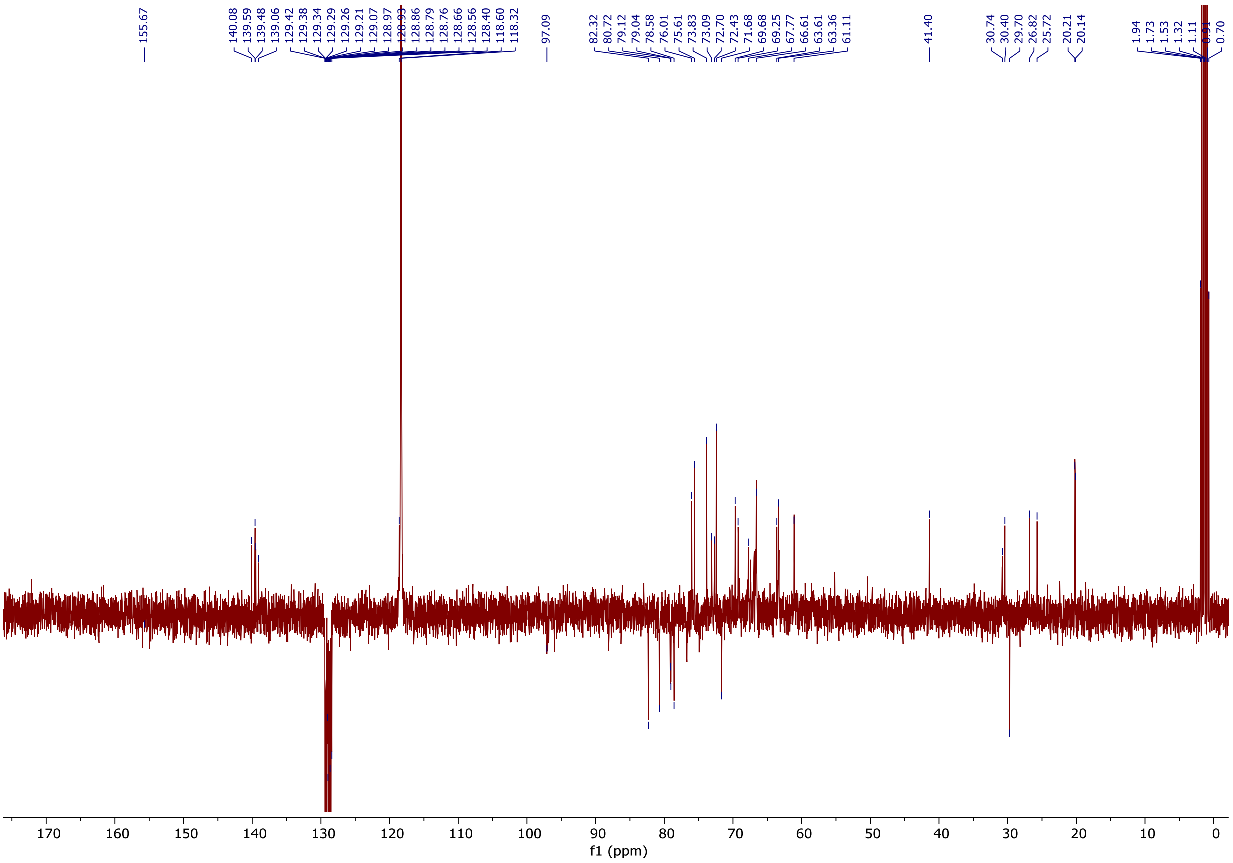
155.67
140.08
139.59
139.48
139.06
129.42
129.38
129.34
129.29
129.26
129.21
129.07
128.97
128.93
128.86
128.79
128.76
128.66
128.56
128.40
118.60
118.32
97.09
82.32
80.72
79.12
79.04
78.58
76.01
75.61
73.83
73.09
72.70
72.43
71.68
69.68
69.25
67.77
66.61
63.61
63.36
61.11
41.40
30.74
30.40
29.70
26.82
25.72
20.21
20.14
1.94
1.73
1.53
1.32
1.11
0.91
0.70
170 160 150 140 130 120 110 100 90 80 70 60 50 40 30 20 10 0
f1 (ppm)

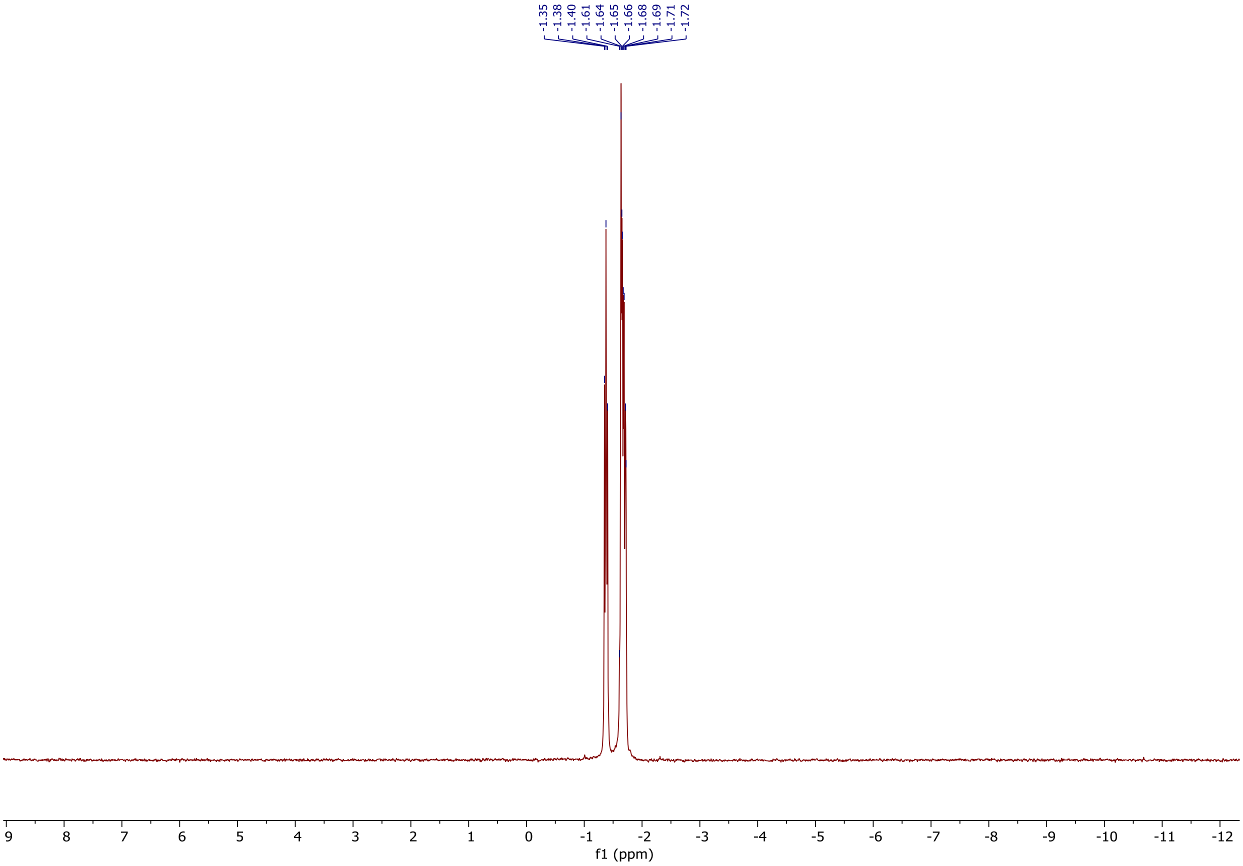
-1.35
-1.38
-1.40
-1.61
-1.64
-1.65
-1.66
-1.68
-1.69
-1.71
-1.72
9 8 7 6 5 4 3 2 1 0 -1 -2 -3 -4 -5 -6 -7 -8 -9 -10 -11 -12
f1 (ppm)

(Protected) (GroP)$_3$(GlcGroP)-Spacer or Tetramer S24

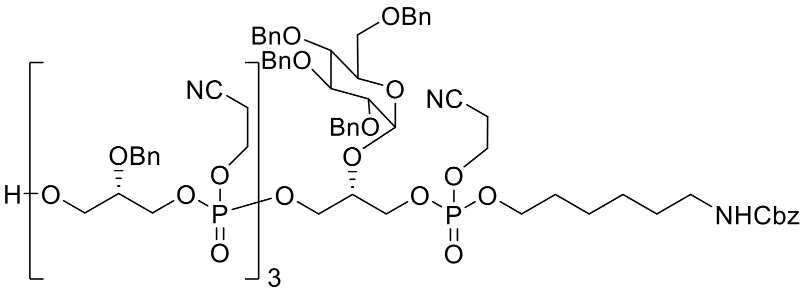

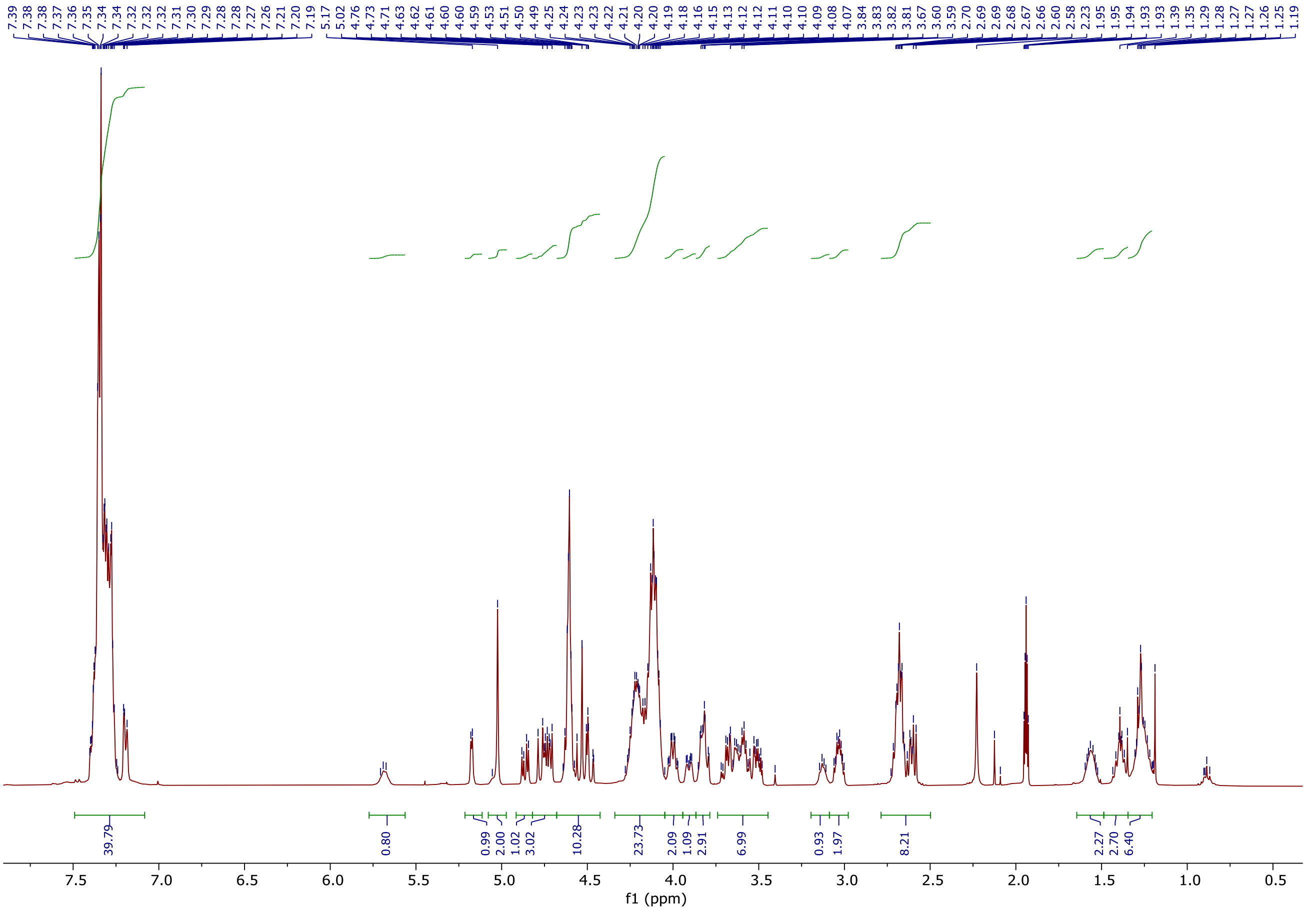

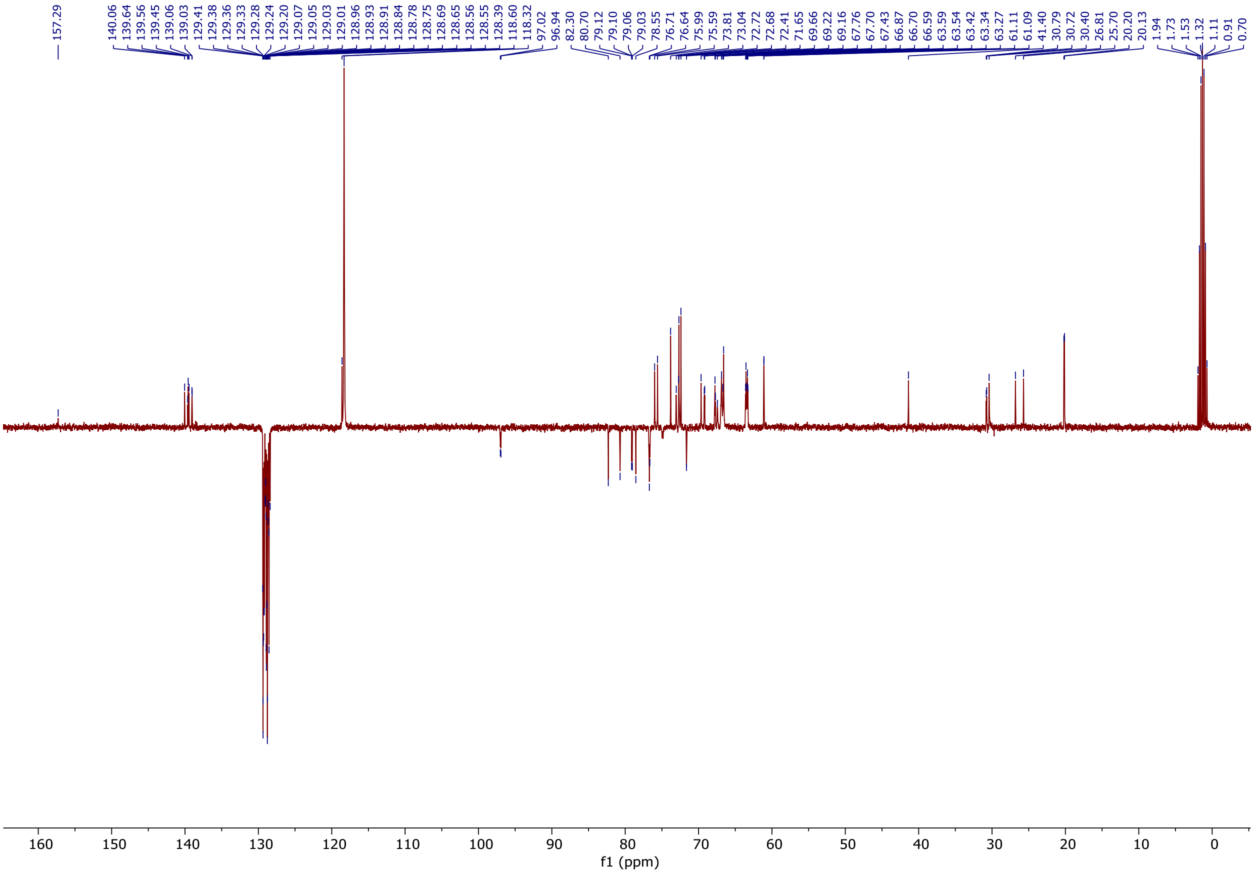
157.29
140.06 139.64 139.56 139.45 139.06 139.03 129.41 129.38 129.36 129.33 129.28 129.24 129.20 129.07 129.05 129.03 129.01 128.96 128.93 128.91 128.84 128.78 128.75 128.69 128.65 128.56 128.55 128.39 118.60 118.32 97.02 96.94 82.30 80.70 79.12 79.10 79.06 79.03 78.55 76.71 76.64 75.99 75.59 73.81 73.04 72.72 72.68 72.41 71.65 69.66 69.22 69.16 67.76 67.70 67.43 66.87 66.70 66.59 63.59 63.54 63.42 63.34 63.27 61.11 61.09 41.40 30.79 30.72 30.40 26.81 25.70 20.20 20.13 1.94 1.73 1.53 1.32 1.11 0.91 0.70
160 150 140 130 120 110 100 90 80 70 60 50 40 30 20 10 0
f1 (ppm)

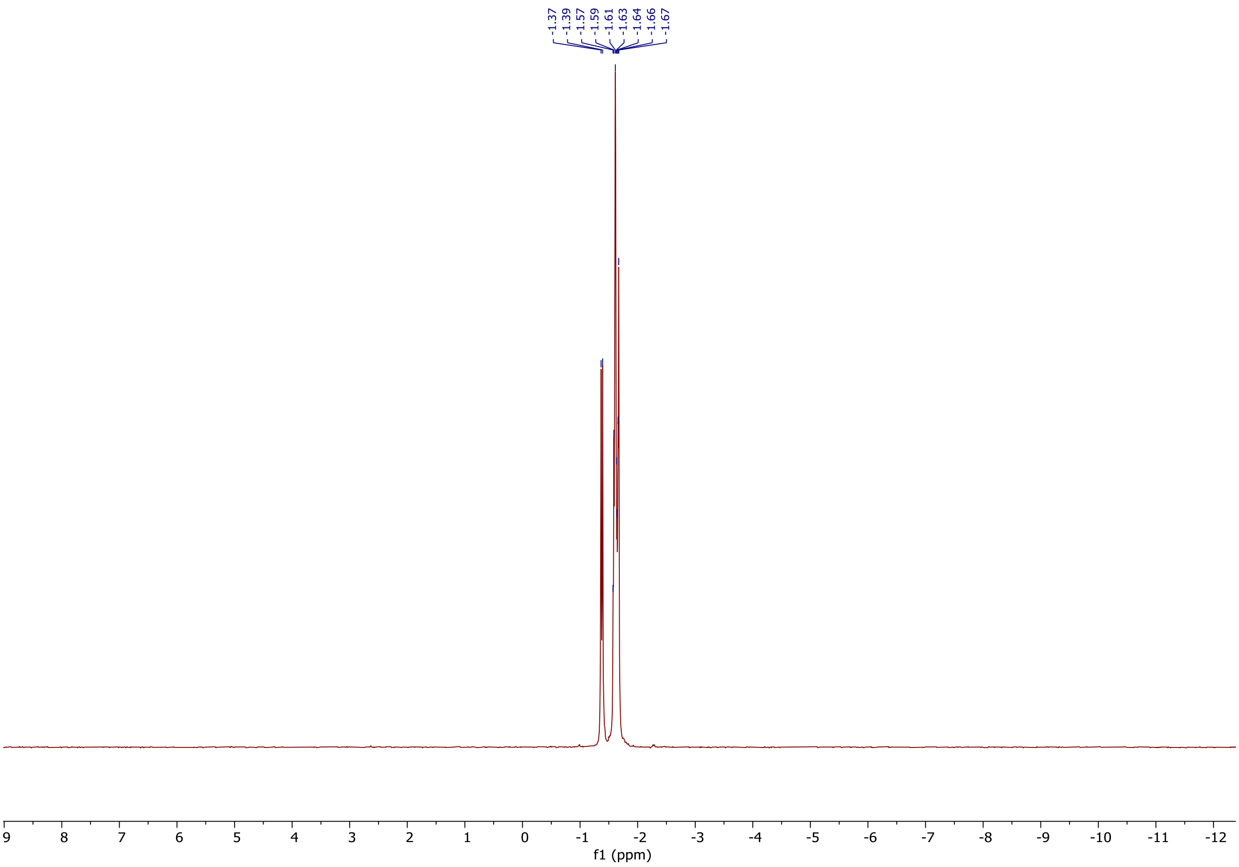
-1.37 -1.39 -1.57 -1.59 -1.61 -1.63 -1.64 -1.66 -1.67
9 8 7 6 5 4 3 2 1 0 -1 -2 -3 -4 -5 -6 -7 -8 -9 -10 -11 -12
f1 (ppm)

(Protected) (GroP)$_4$(GlcGroP)-Spacer or Pentamer S25

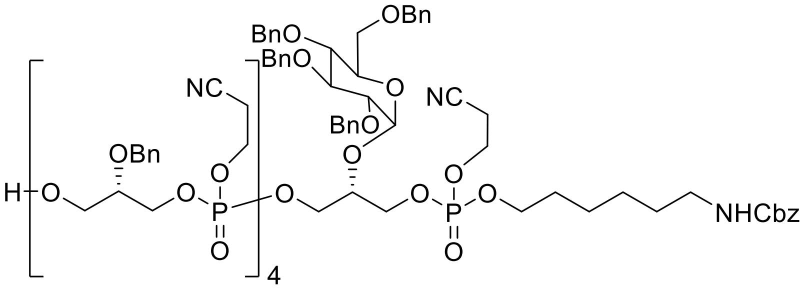

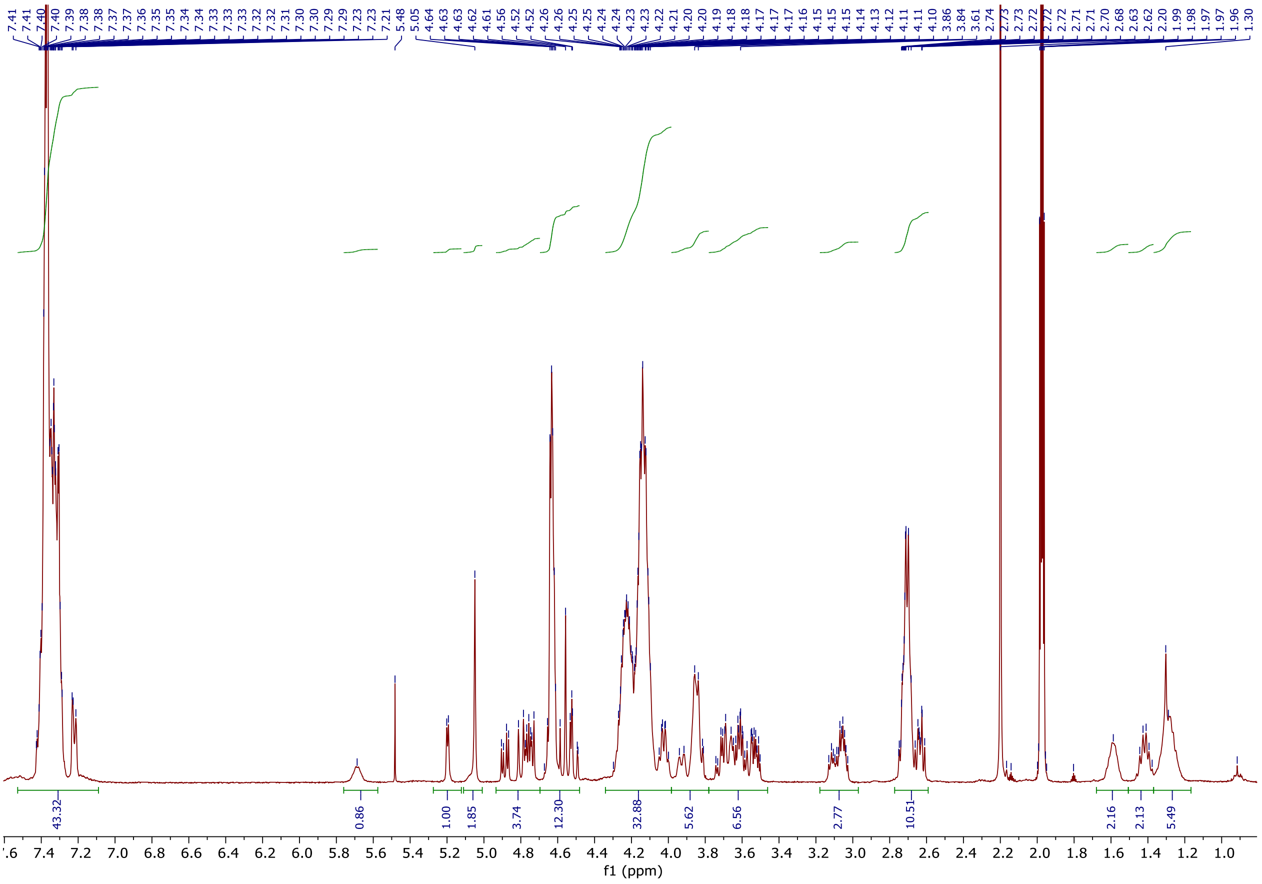

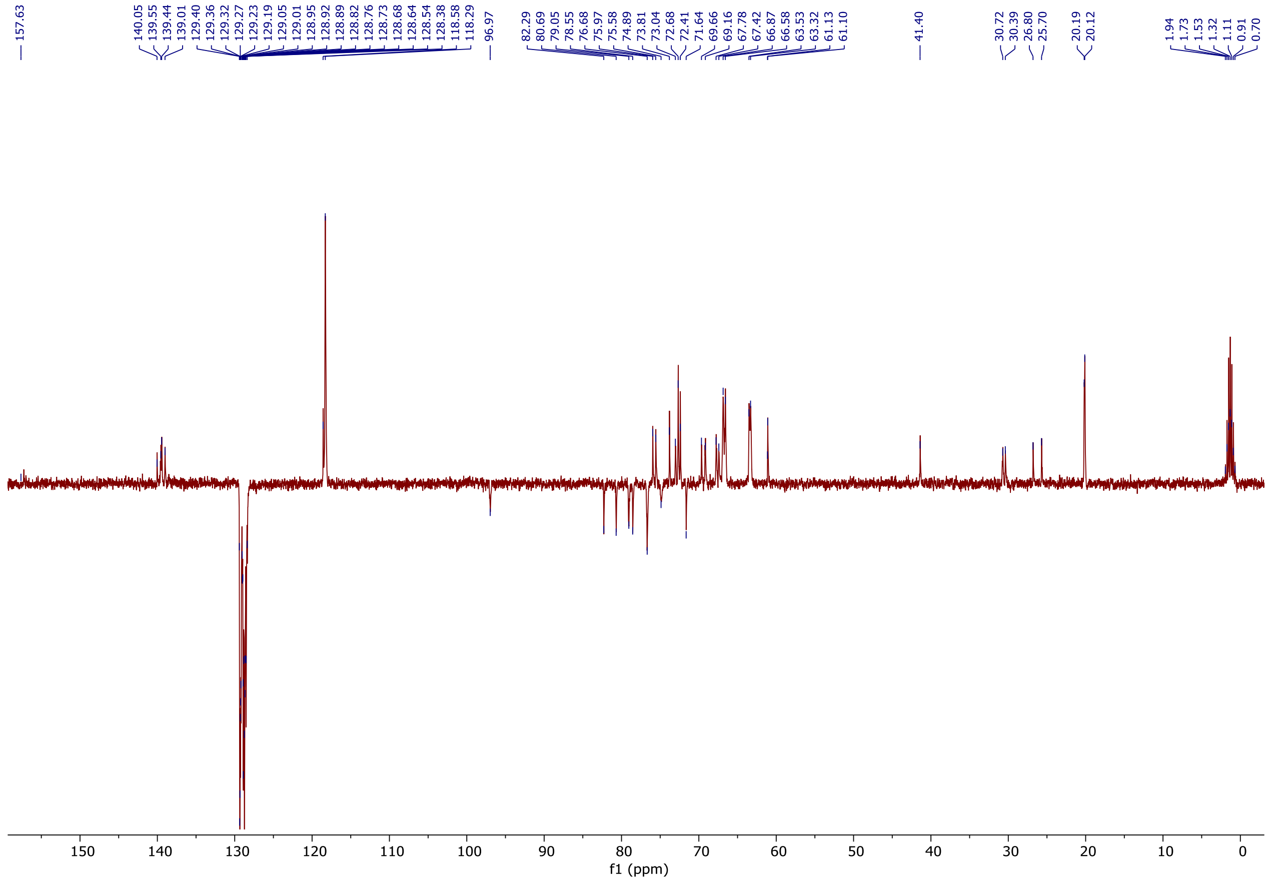
157.63
140.05
139.55
139.44
139.01
129.40
129.36
129.32
129.27
129.23
129.19
129.05
129.01
128.95
128.92
128.89
128.82
128.76
128.73
128.68
128.64
128.54
128.38
118.58
118.29
96.97
82.29
80.69
79.05
78.55
76.68
75.97
75.58
74.89
73.81
73.04
72.68
72.41
71.64
69.66
69.16
67.78
67.42
66.87
66.58
63.53
63.32
61.13
61.10
41.40
30.72
30.39
26.80
25.70
20.19
20.12
1.94
1.73
1.53
1.32
1.11
0.91
0.70
150
140
130
120
110
100
90
80
70
60
50
40
30
20
10
0
f1 (ppm)

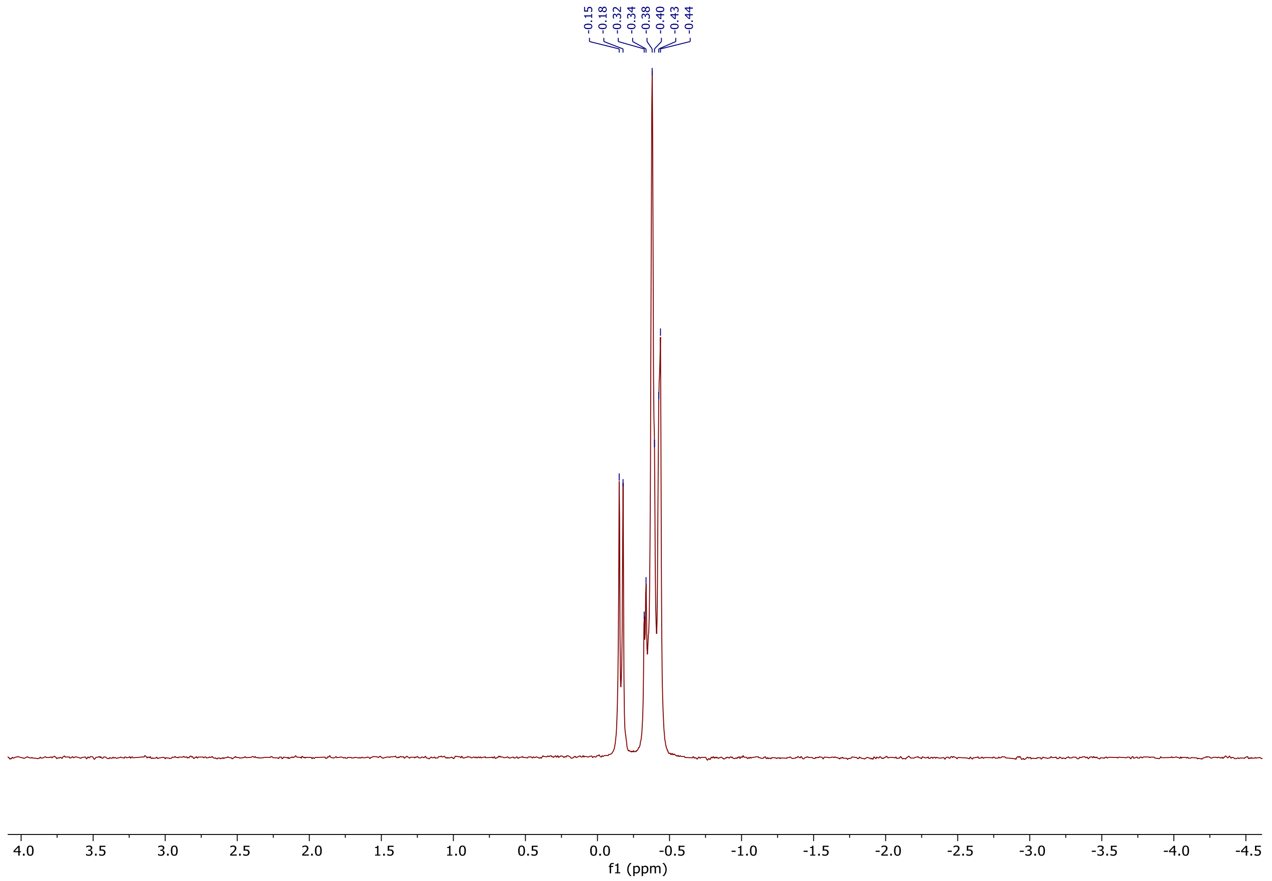
-0.15
-0.18
-0.32
-0.34
-0.38
-0.40
-0.43
-0.44
4.0
3.5
3.0
2.5
2.0
1.5
1.0
0.5
0.0
-0.5
-1.0
-1.5
-2.0
-2.5
-3.0
-3.5
-4.0
-4.5
f1 (ppm)

(Protected) (GroP)$_5$(GlcGroP)-Spacer or Hexamer 28

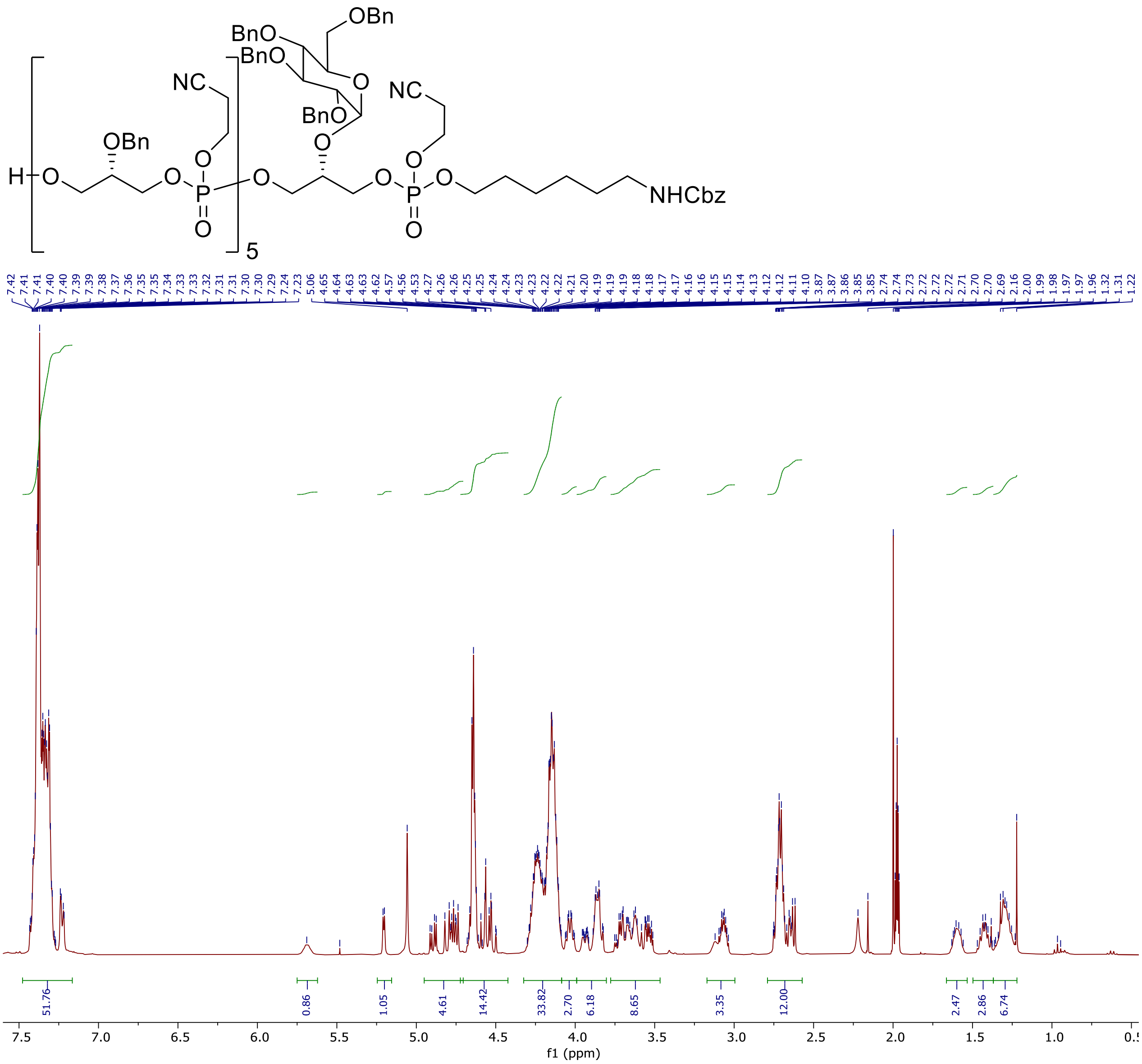

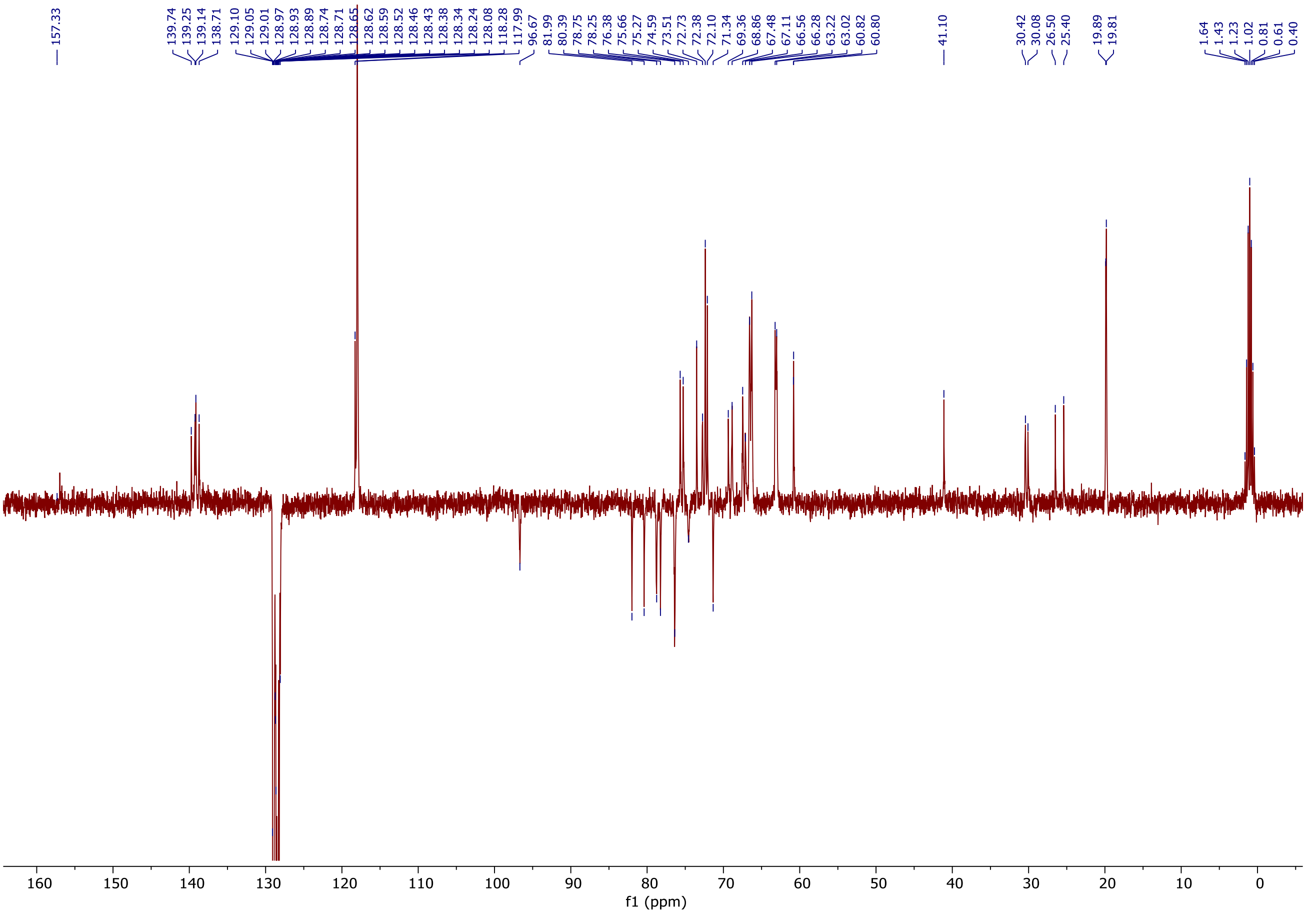
157.33
139.74
139.25
139.14
138.71
129.10
129.05
129.01
128.97
128.93
128.89
128.74
128.71
128.65
128.62
128.59
128.52
128.46
128.43
128.38
128.34
128.24
128.08
118.28
117.99
96.67
81.99
80.39
78.75
78.25
76.38
75.66
75.27
74.59
73.51
72.73
72.38
72.10
71.34
69.36
68.86
67.48
67.11
66.56
66.28
63.22
63.02
60.82
60.80
41.10
30.42
30.08
26.50
25.40
19.89
19.81
1.64
1.43
1.23
1.02
0.81
0.61
0.40
160
150
140
130
120
110
100
90
80
70
60
50
40
30
20
10
0
f1 (ppm)

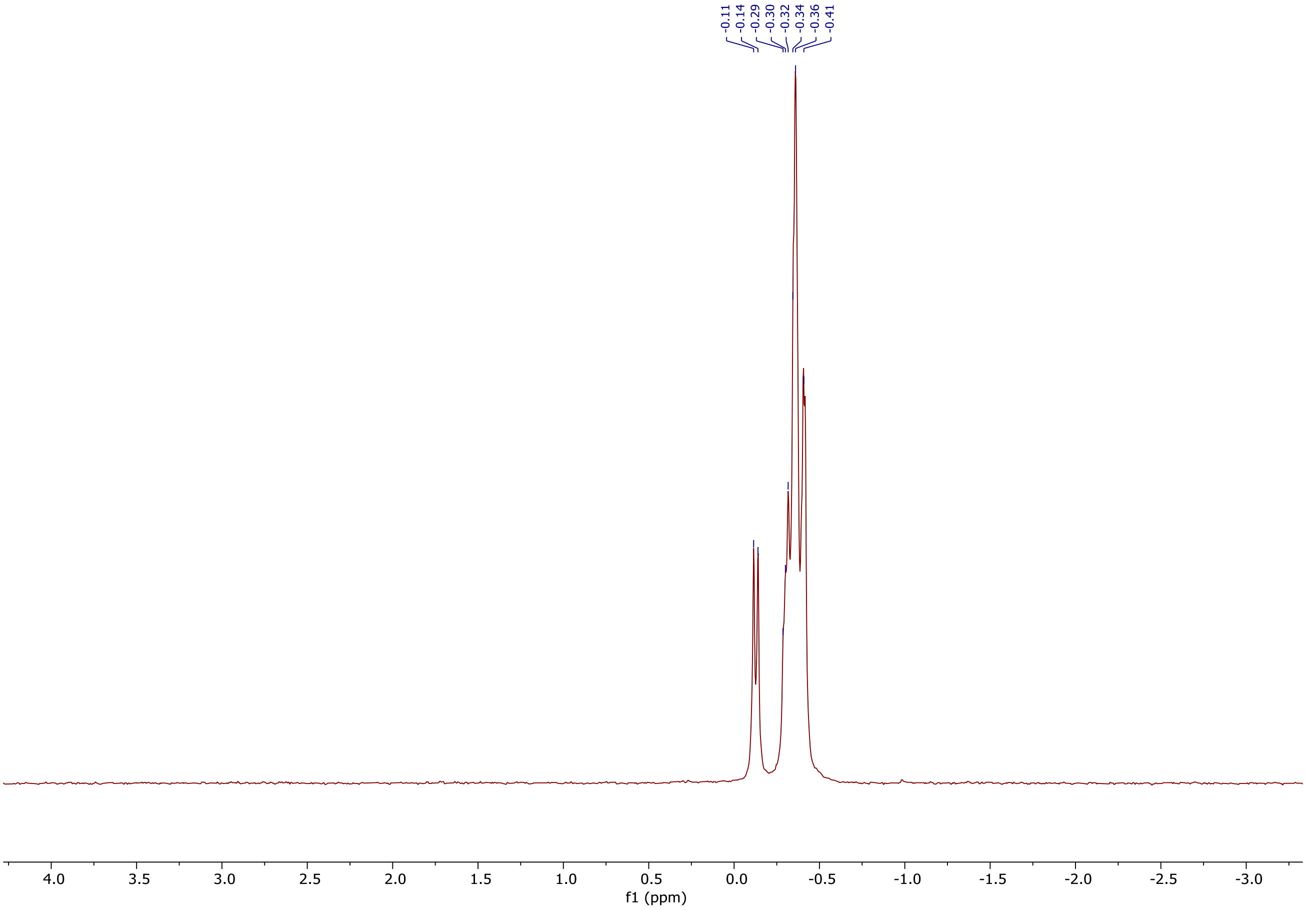
-0.11
-0.14
-0.29
-0.30
-0.32
-0.34
-0.36
-0.41
4.0
3.5
3.0
2.5
2.0
1.5
1.0
0.5
0.0
-0.5
-1.0
-1.5
-2.0
-2.5
-3.0
f1 (ppm)

(GlcGroP)(GroP)$_5$-Spacer or Hexamer (1)

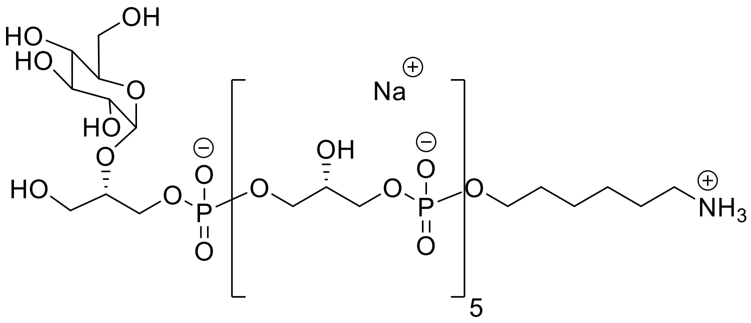

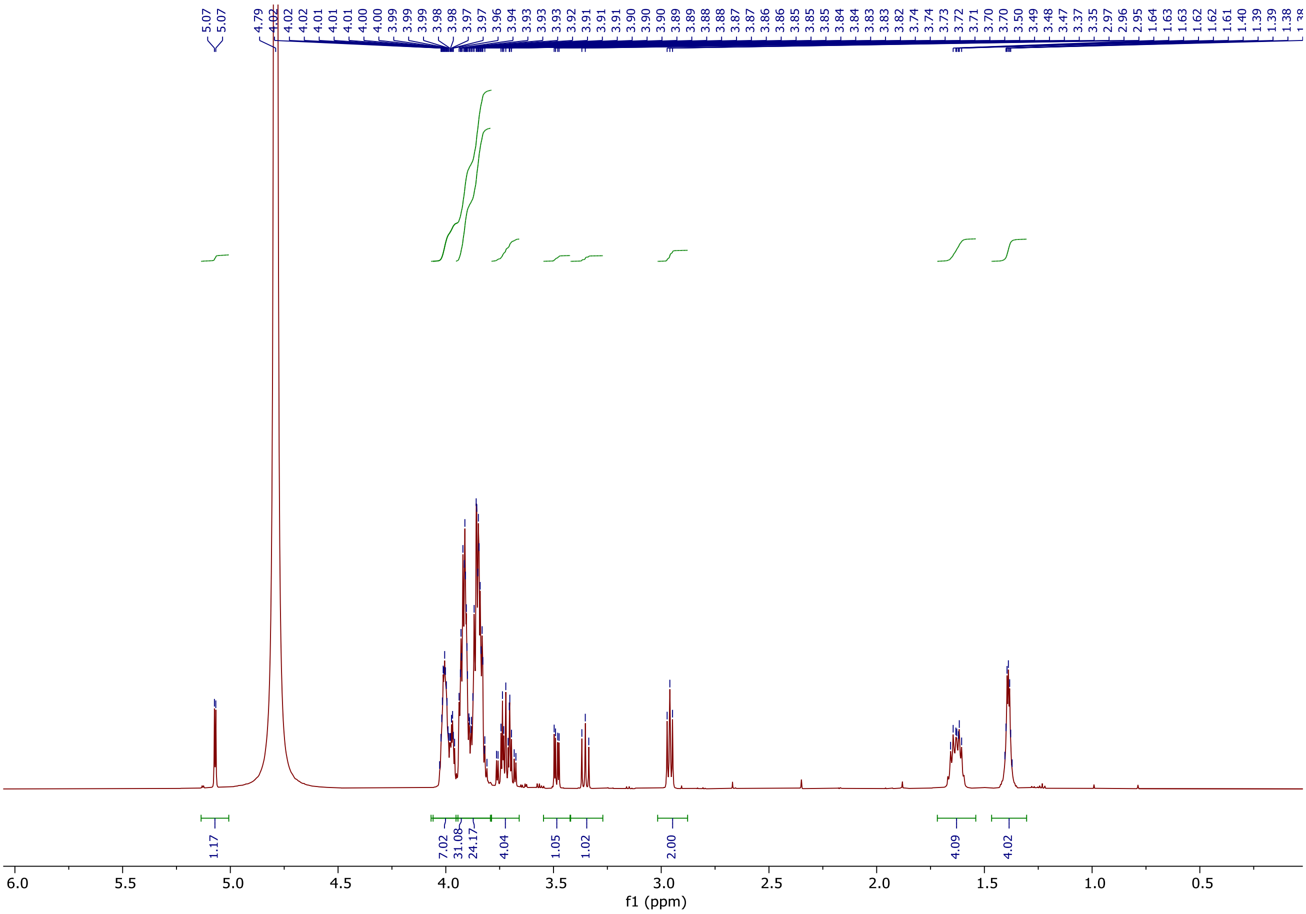

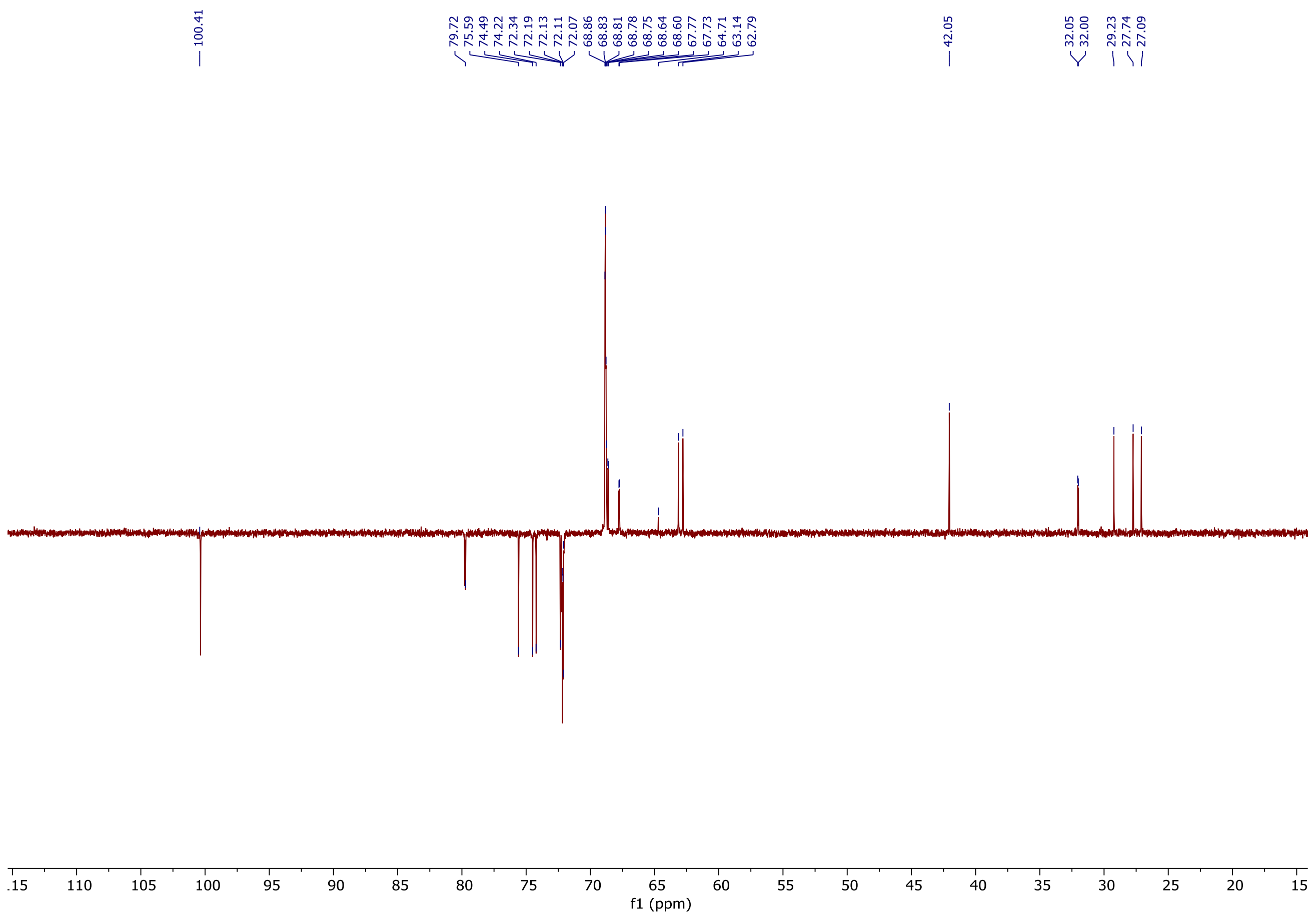
100.41
79.72
75.59
74.49
74.22
72.34
72.19
72.13
72.11
72.07
68.86
68.83
68.81
68.78
68.75
68.64
68.60
67.77
67.73
64.71
63.14
62.79
42.05
32.05
32.00
29.23
27.74
27.09
f1 (ppm)

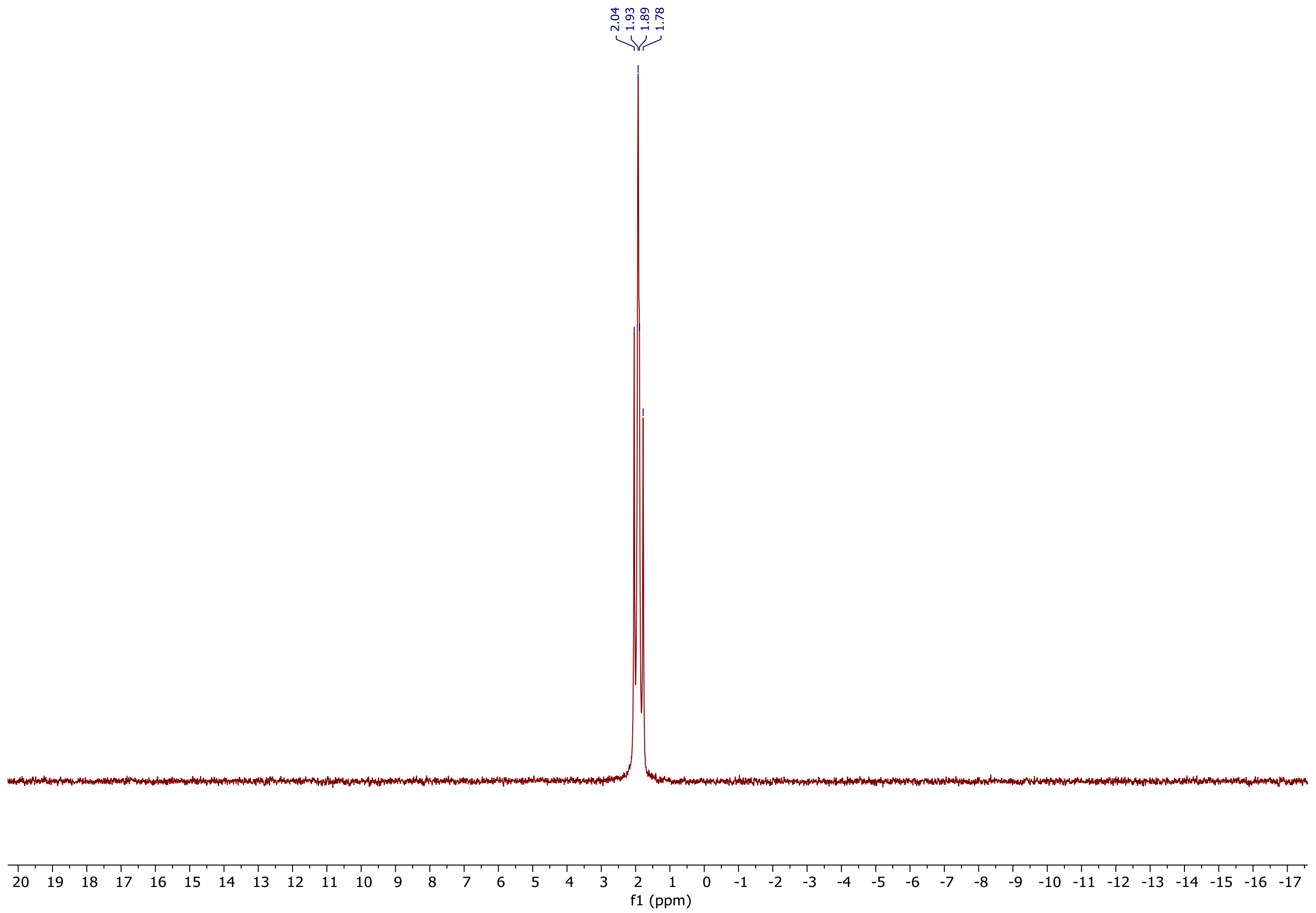
2.04
1.93
1.89
1.78
f1 (ppm)

(GroP)(GlcGroP)(GroP)$_4$-Spacer or Hexamer (2)

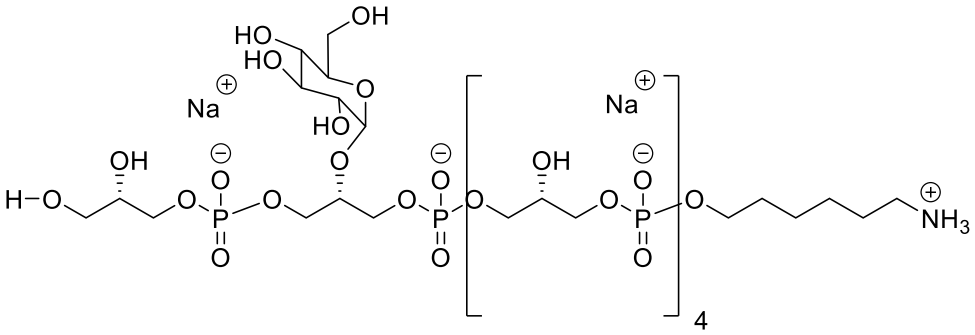

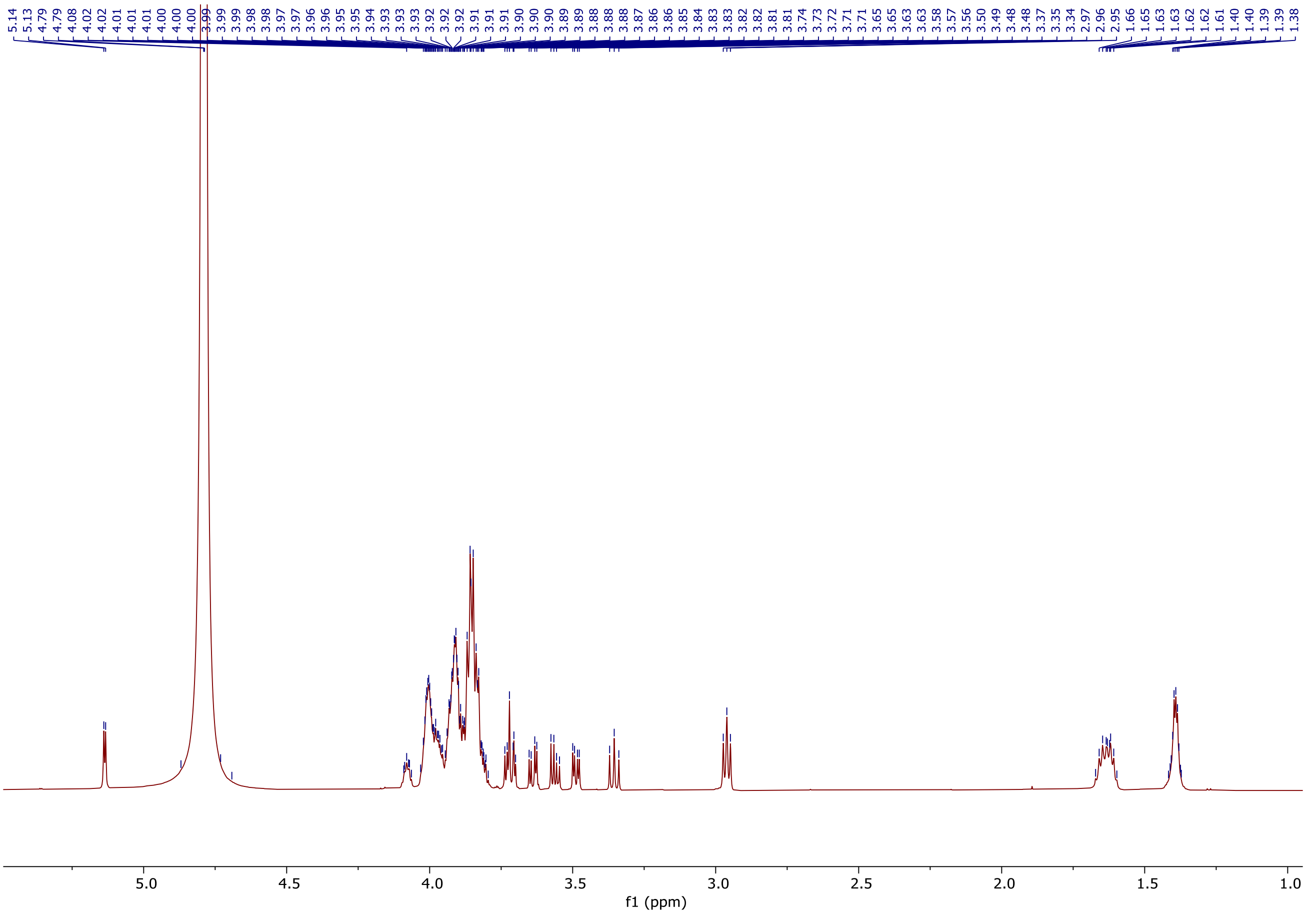

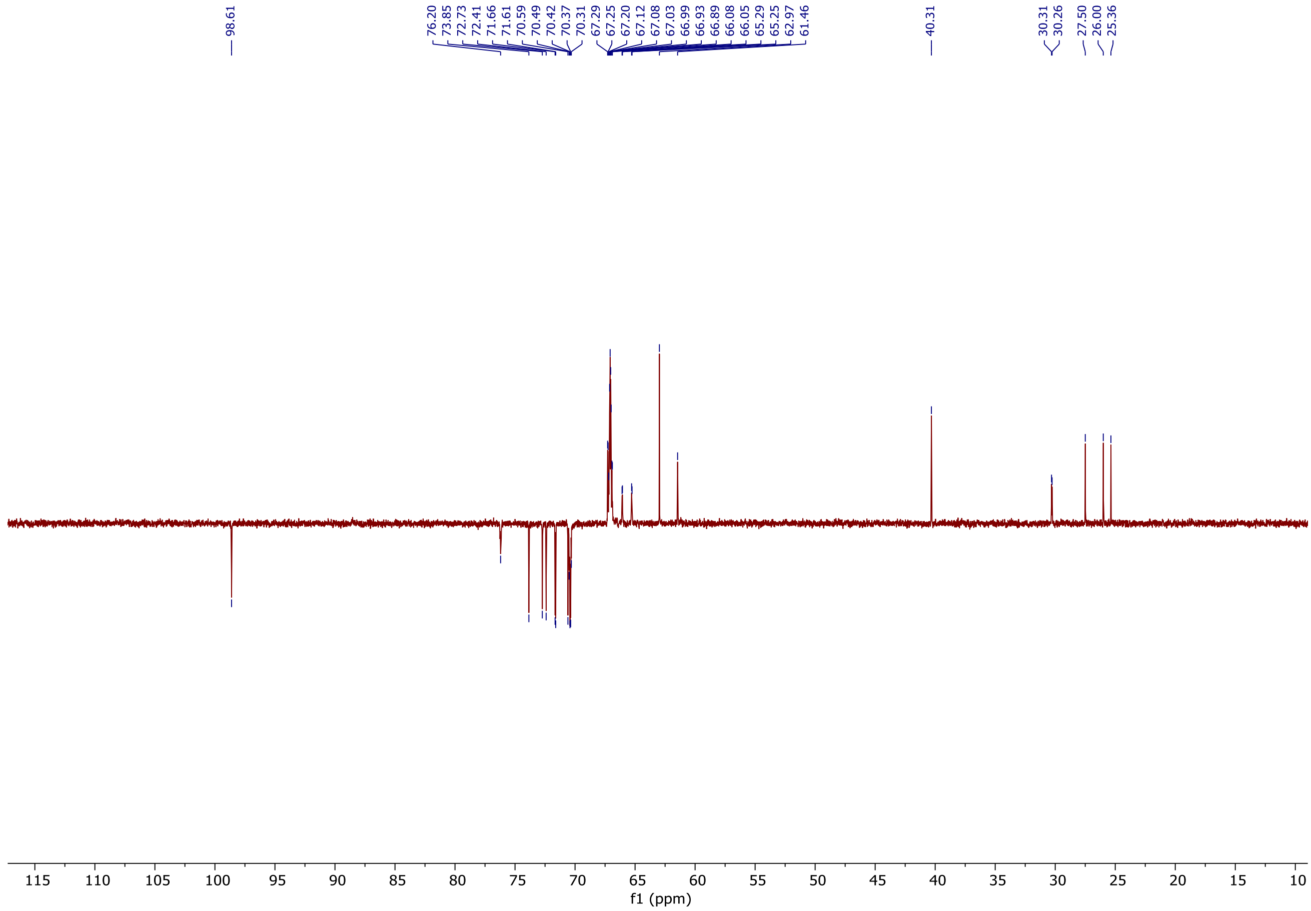
98.61
76.20
73.85
72.73
72.41
71.66
71.61
70.59
70.49
70.42
70.37
70.31
67.29
67.25
67.20
67.12
67.08
67.03
66.99
66.93
66.89
66.08
66.05
65.29
65.25
62.97
61.46
40.31
30.31
30.26
27.50
26.00
25.36
f1 (ppm)

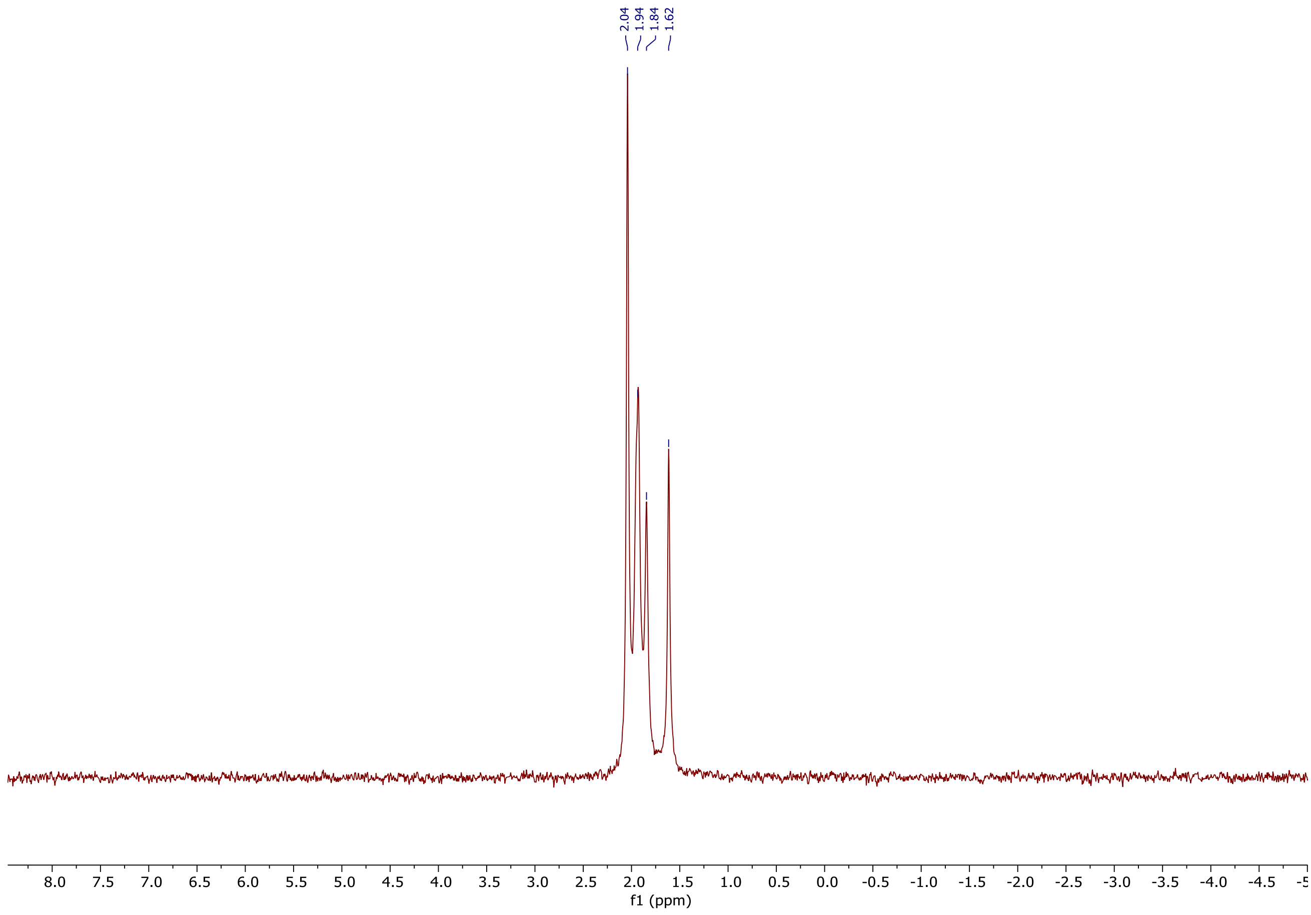
2.04
1.94
1.84
1.62
f1 (ppm)

(GroP)$_2$(GlcGroP)(GroP)$_3$-Spacer or Hexamer (3)

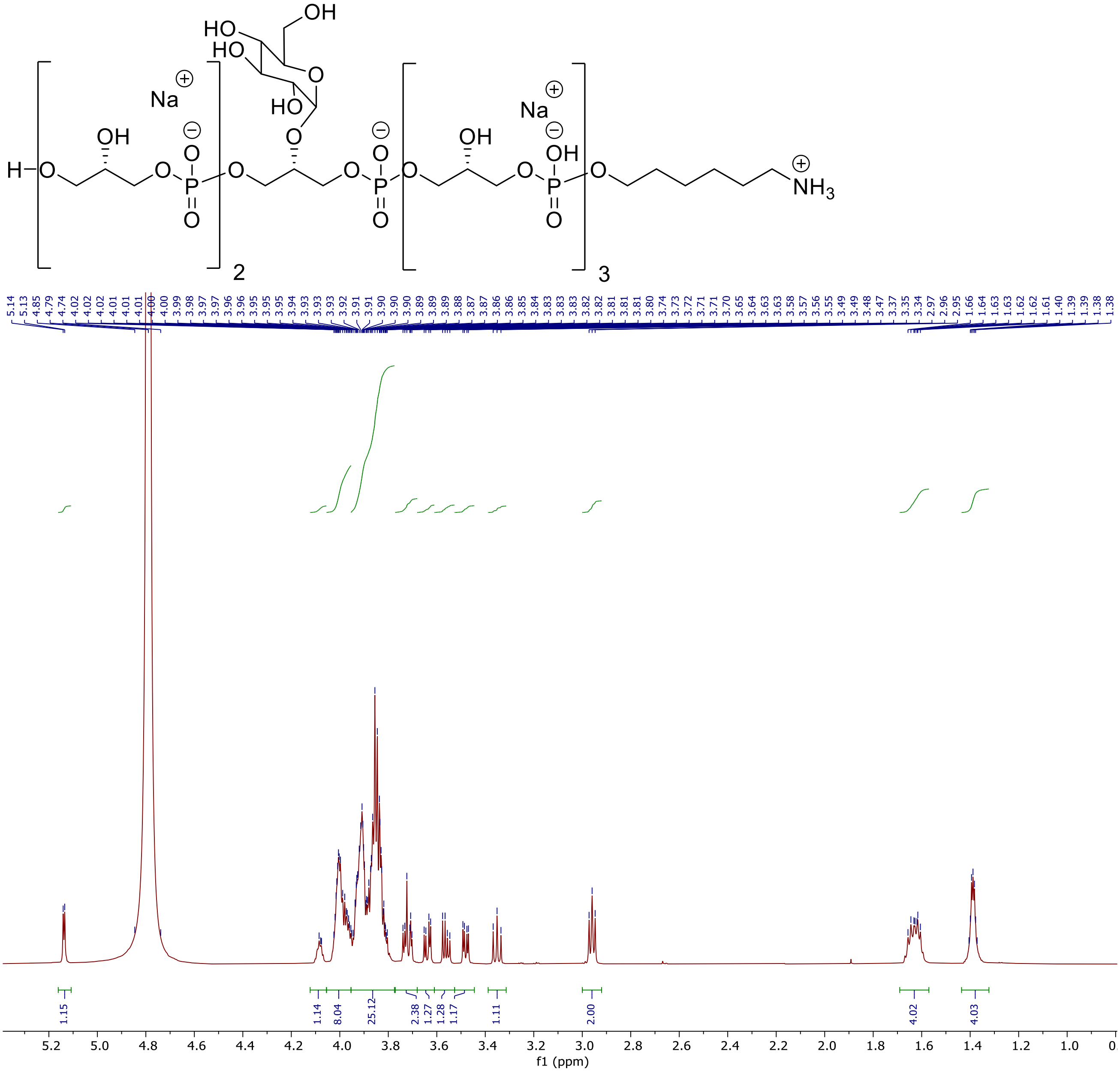

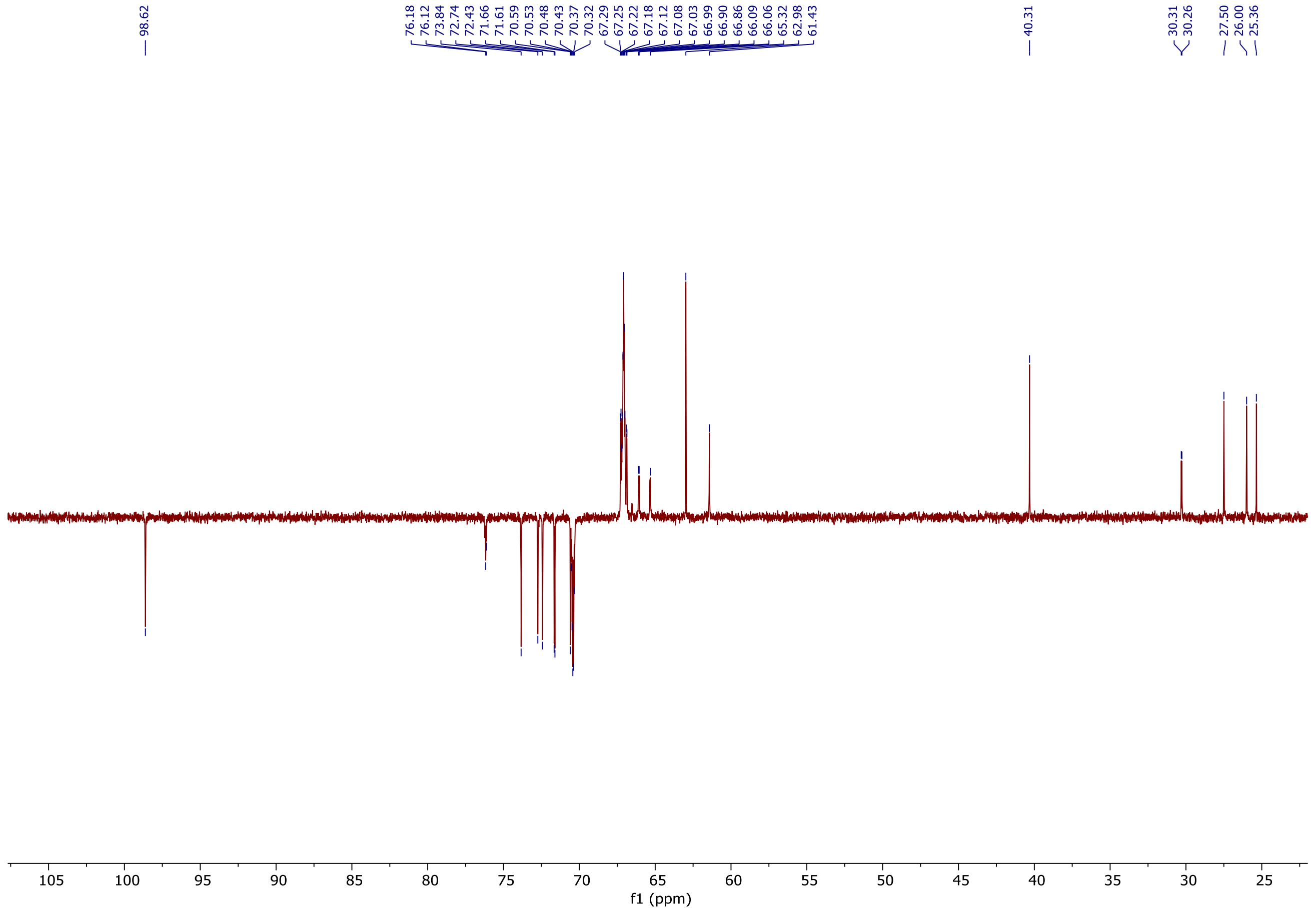
98.62
76.18
76.12
73.84
72.74
72.43
71.66
71.61
70.59
70.53
70.48
70.43
70.37
70.32
67.29
67.25
67.22
67.18
67.12
67.08
67.03
66.99
66.90
66.86
66.09
66.06
65.32
62.98
61.43
40.31
30.31
30.26
27.50
26.00
25.36
105 100 95 90 85 80 75 70 65 60 55 50 45 40 35 30 25
f1 (ppm)

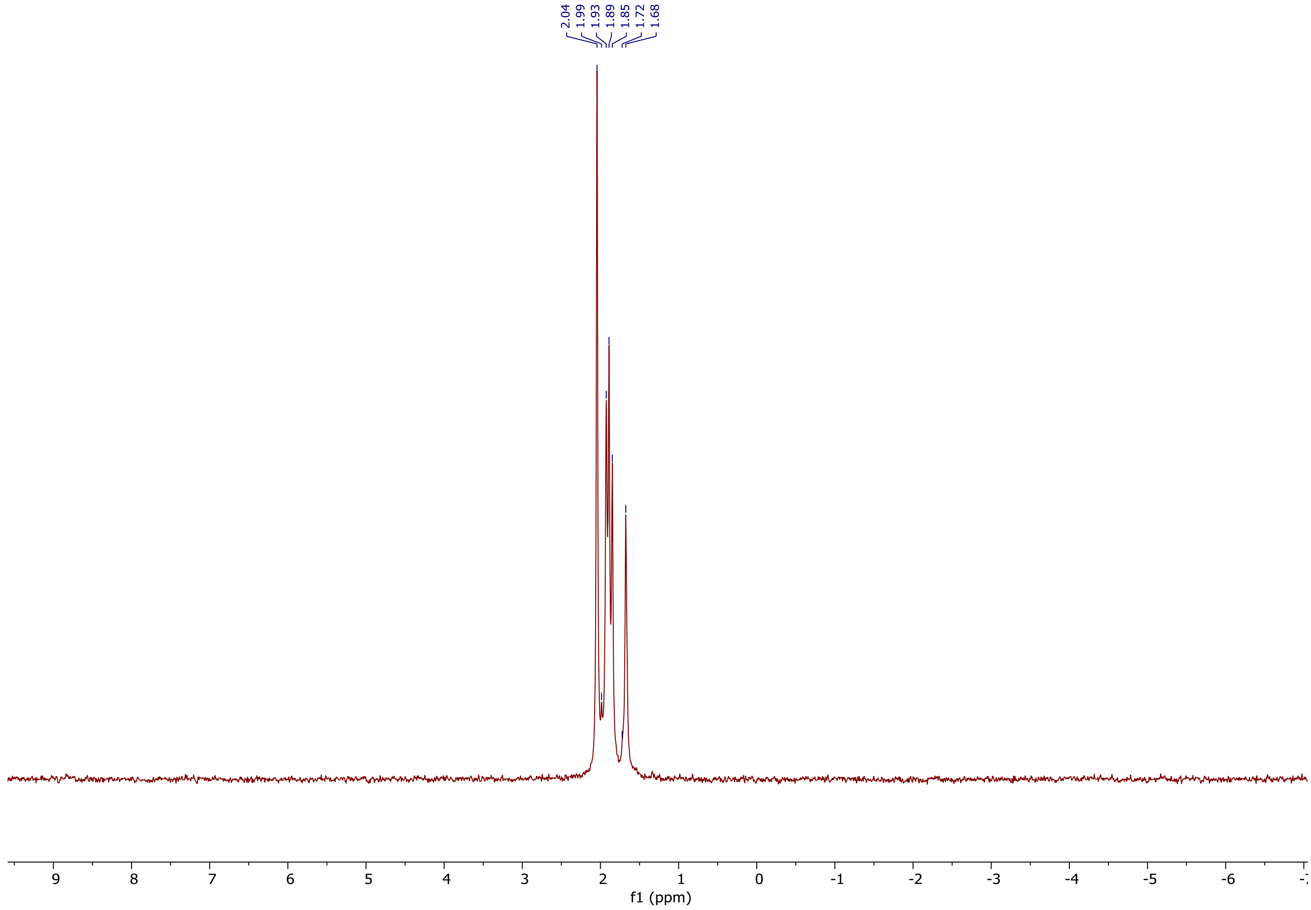
2.04
1.99
1.93
1.89
1.85
1.72
1.68
9 8 7 6 5 4 3 2 1 0 -1 -2 -3 -4 -5 -6
f1 (ppm)

(GroP)$_3$(GlcGroP)(GroP)$_2$-Spacer or Hexamer (4)

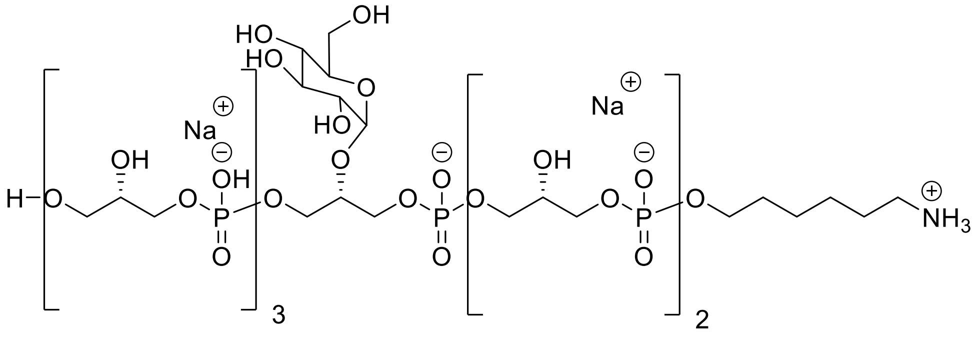

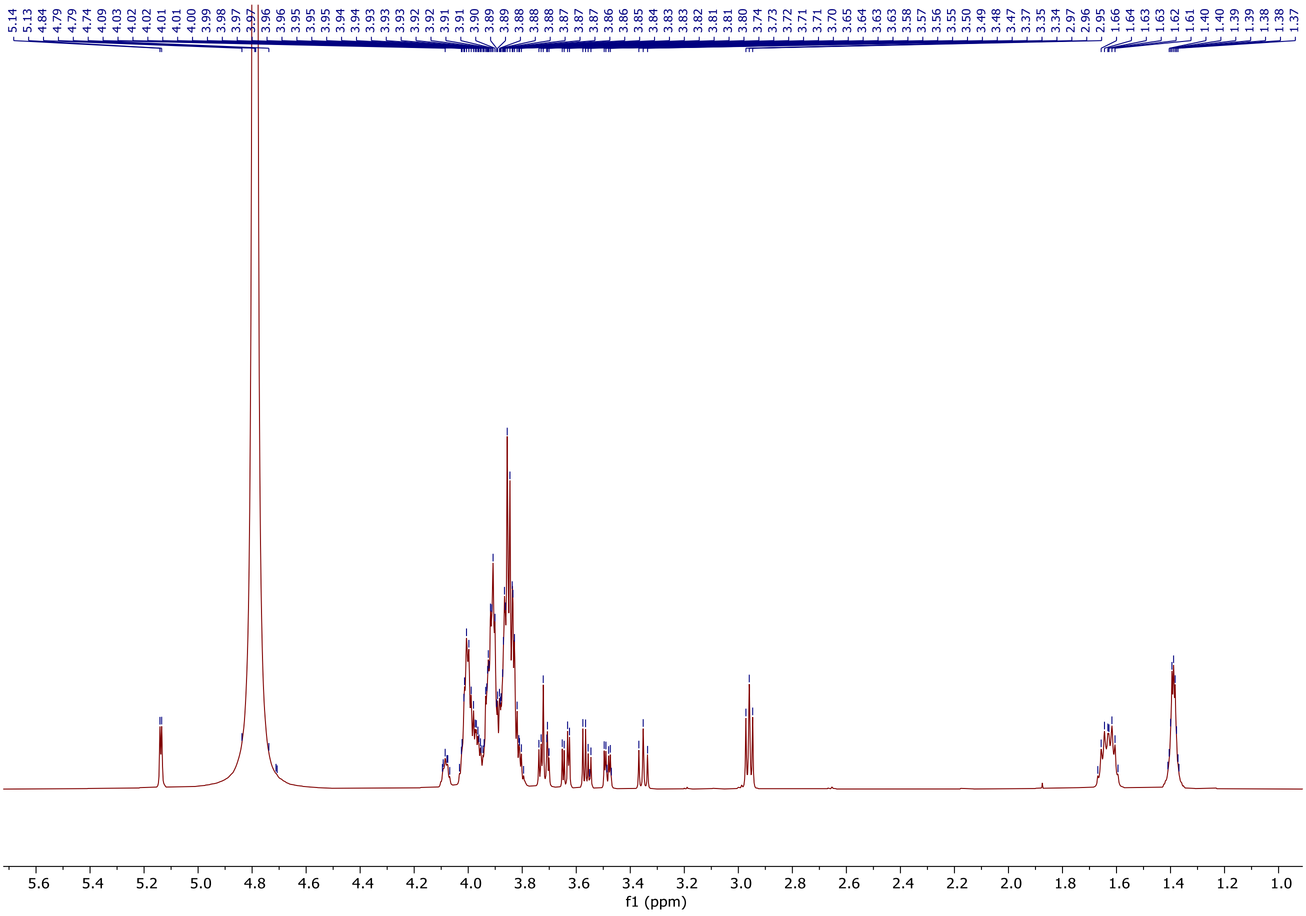

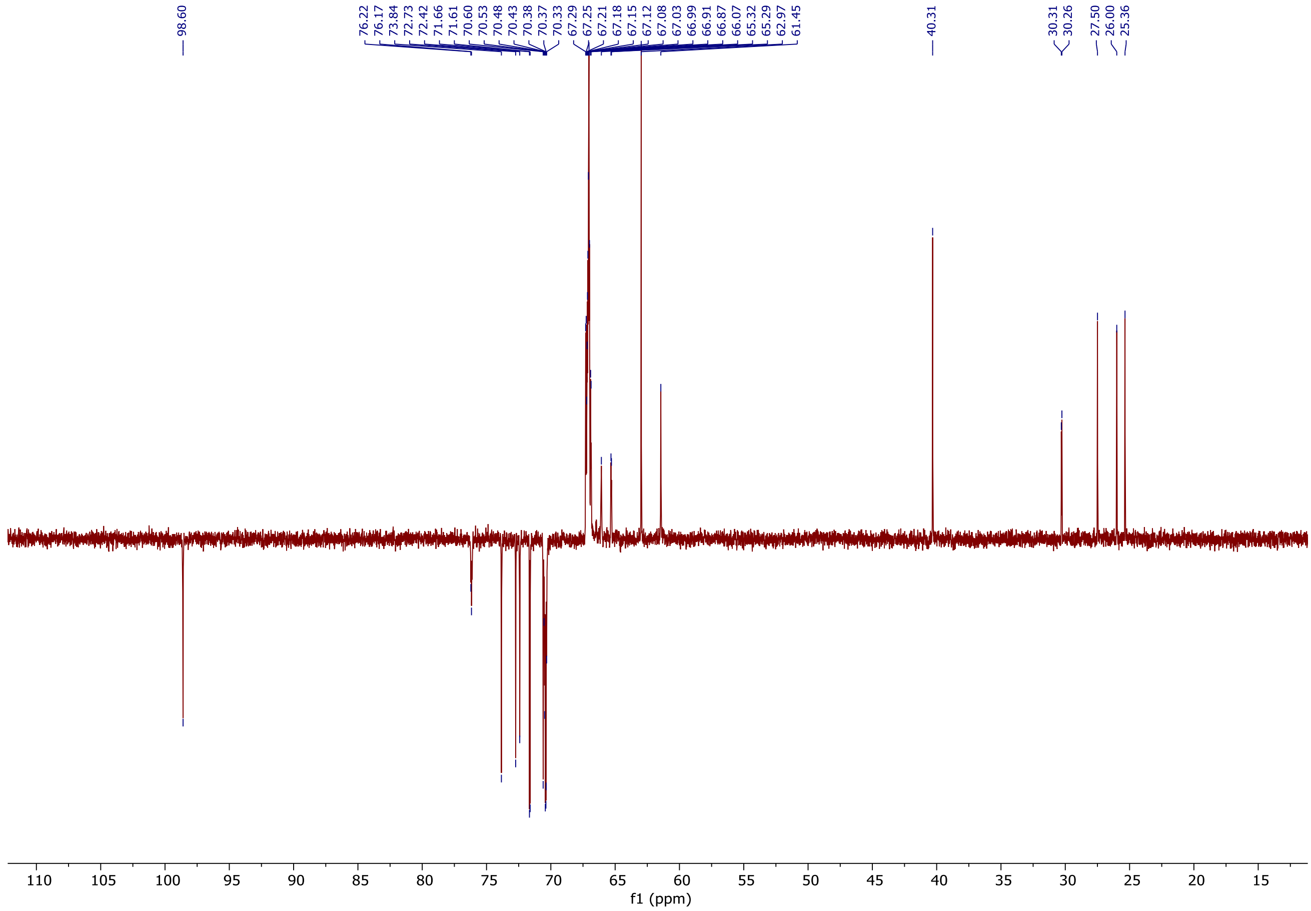
98.60
76.22
76.17
73.84
72.73
72.42
71.66
71.61
70.60
70.53
70.48
70.43
70.38
70.37
70.33
67.29
67.25
67.21
67.18
67.15
67.12
67.08
67.03
66.99
66.91
66.87
66.07
65.32
65.29
62.97
61.45
40.31
30.31
30.26
27.50
26.00
25.36
f1 (ppm)

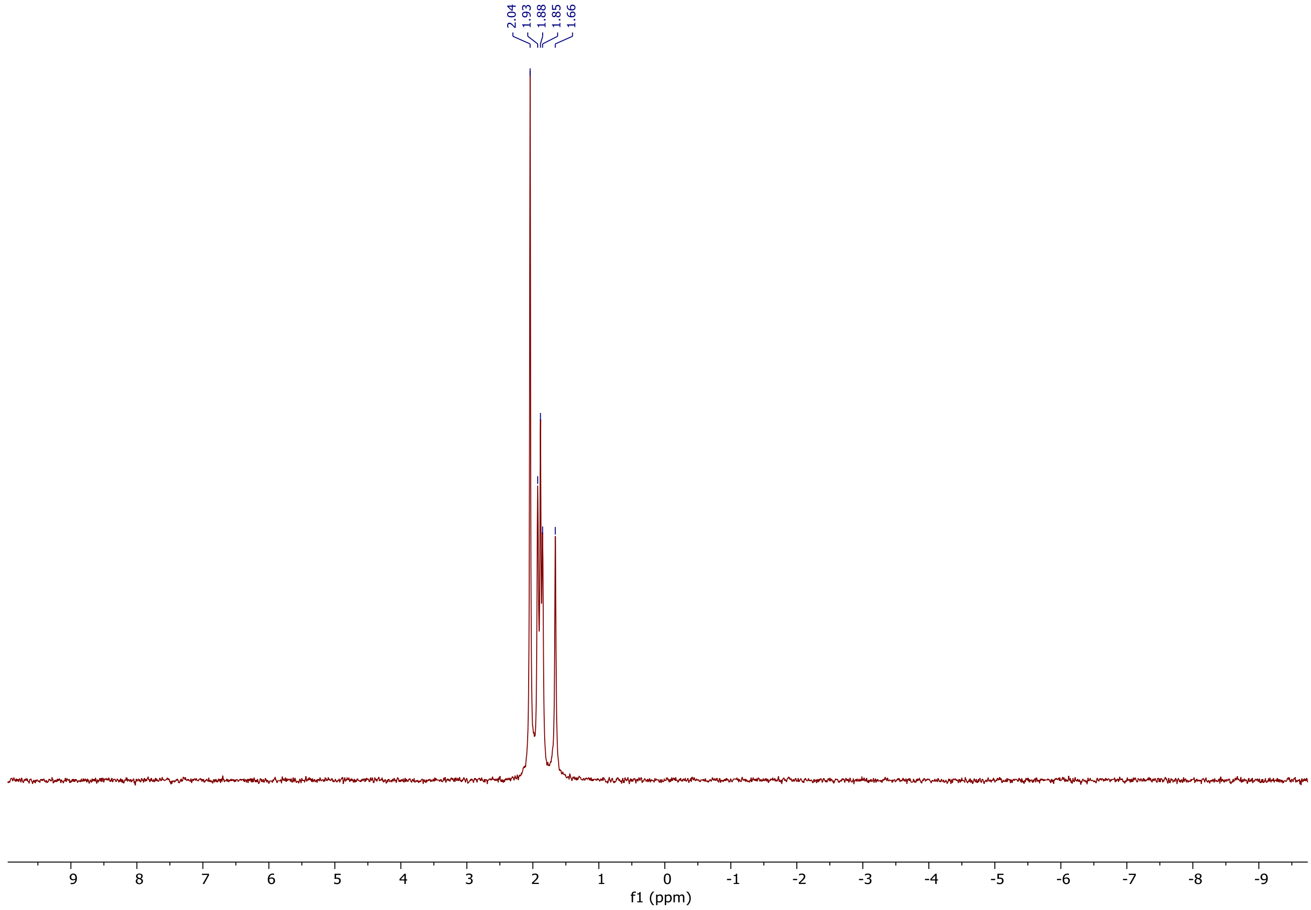
2.04
1.93
1.88
1.85
1.66
f1 (ppm)

$(GroP)_4(GlcGroP)(GroP)$ -Spacer or Hexamer (5)

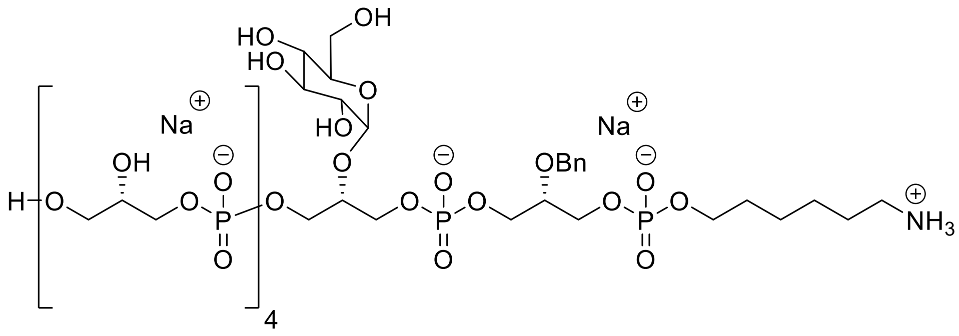

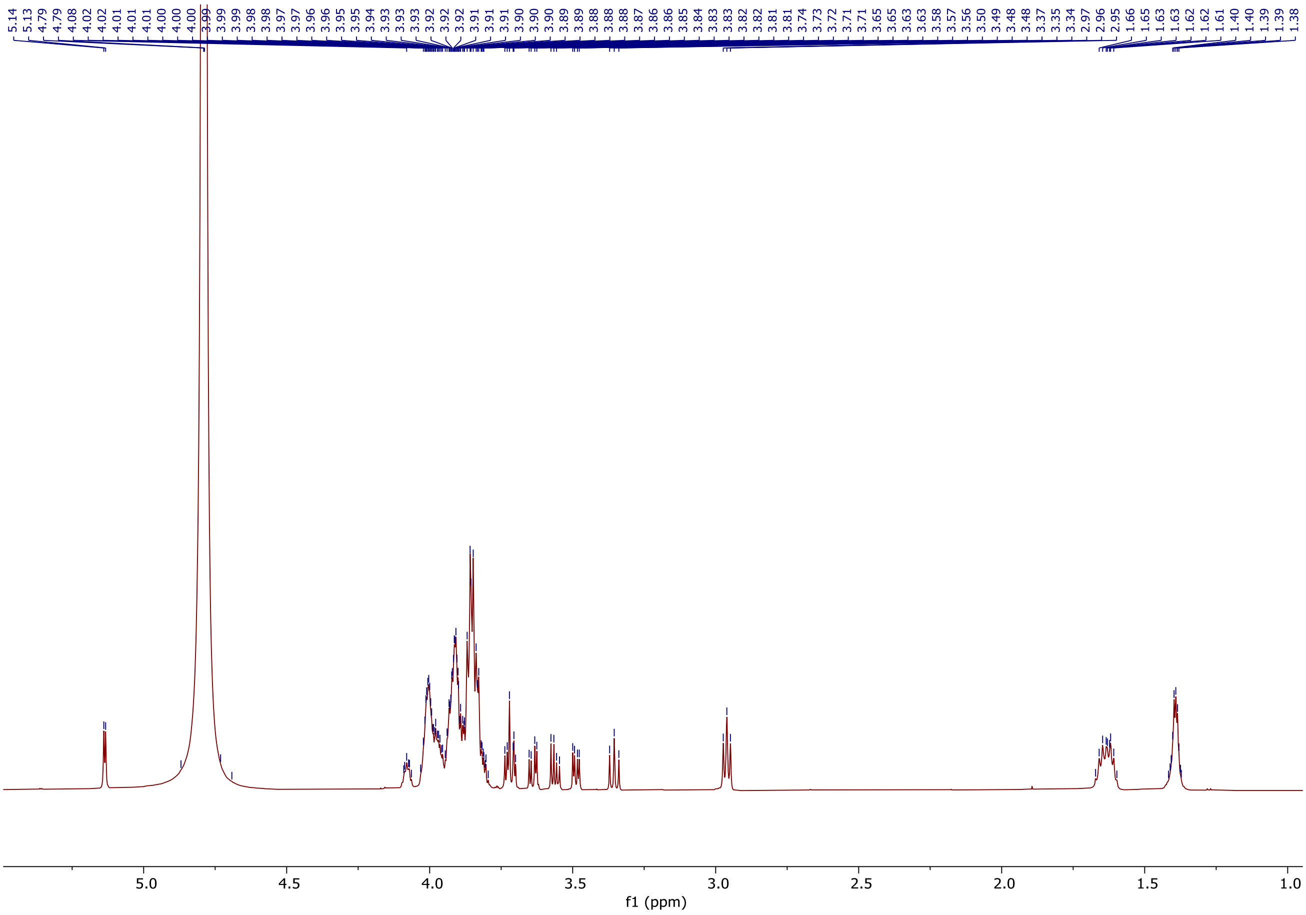

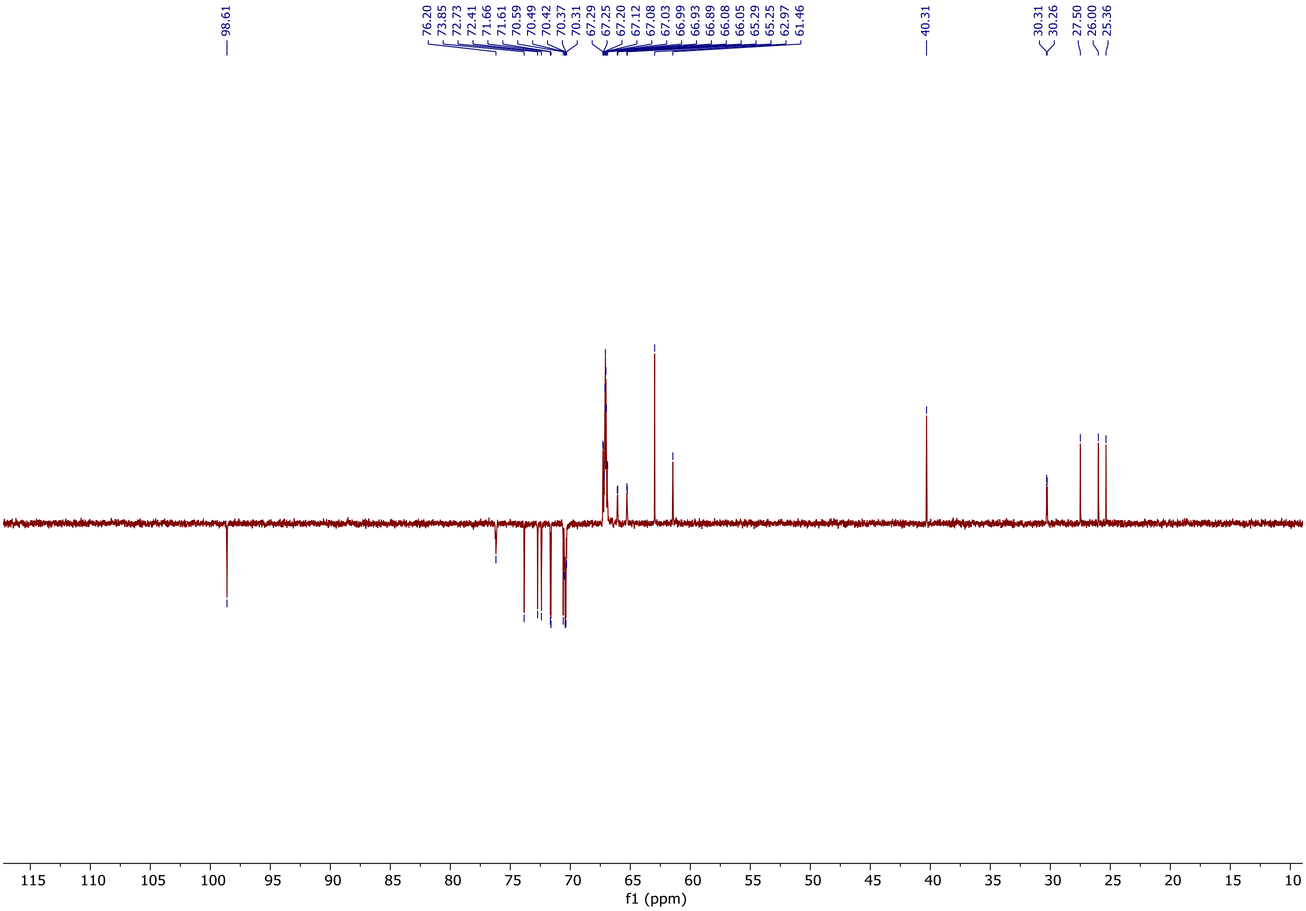
98.61
76.20
73.85
72.73
72.41
71.66
71.61
70.59
70.49
70.42
70.37
70.31
67.29
67.25
67.20
67.12
67.08
67.03
66.99
66.93
66.89
66.08
66.05
65.29
65.25
62.97
61.46
40.31
30.31
30.26
27.50
26.00
25.36
115 110 105 100 95 90 85 80 75 70 65 60 55 50 45 40 35 30 25 20 15 10
f1 (ppm)

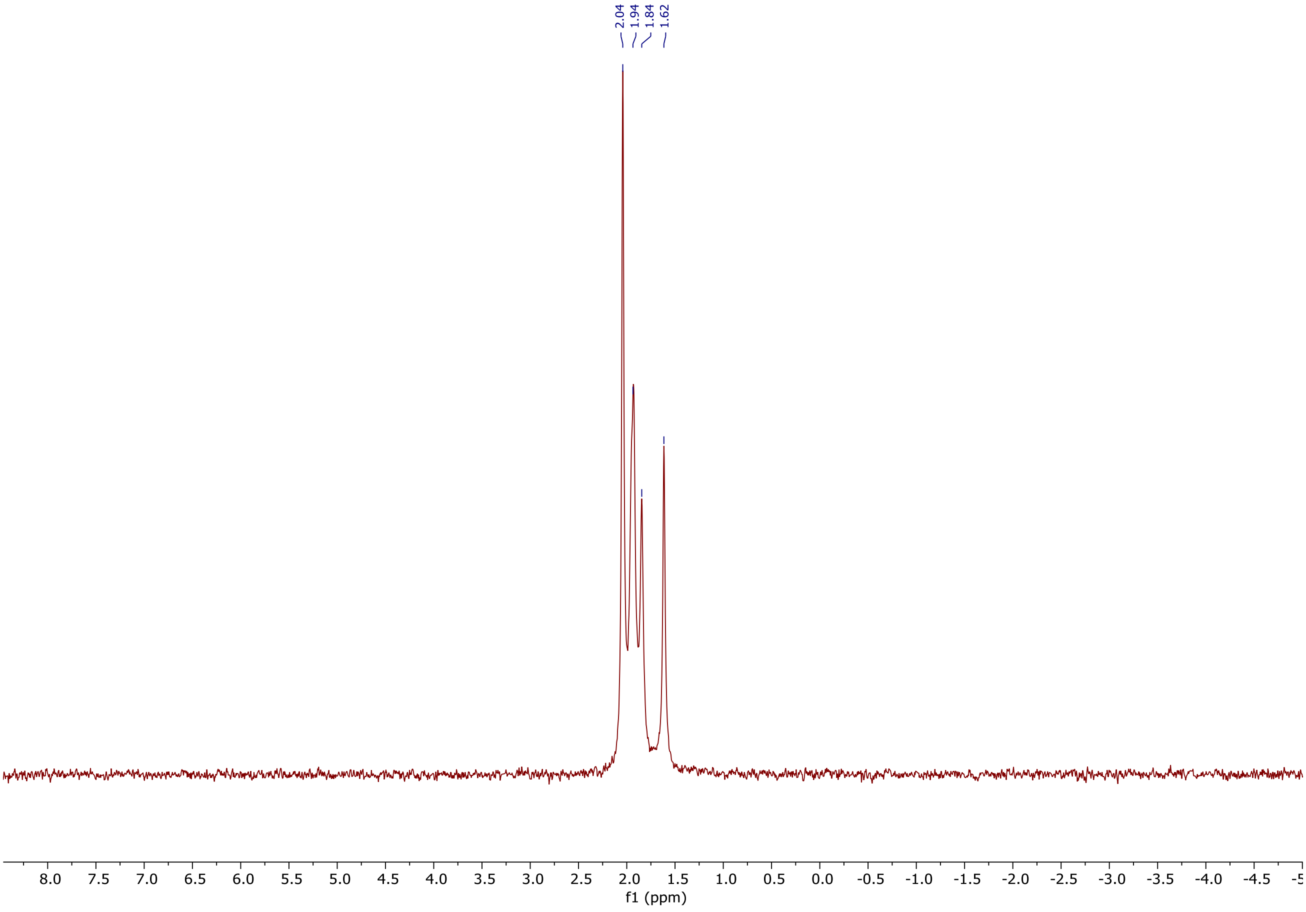
2.04
1.94
1.84
1.62
8.0 7.5 7.0 6.5 6.0 5.5 5.0 4.5 4.0 3.5 3.0 2.5 2.0 1.5 1.0 0.5 0.0 -0.5 -1.0 -1.5 -2.0 -2.5 -3.0 -3.5 -4.0 -4.5
f1 (ppm)

(GroP)$_5$(GlcGroP)-Spacer or Hexamer (6)

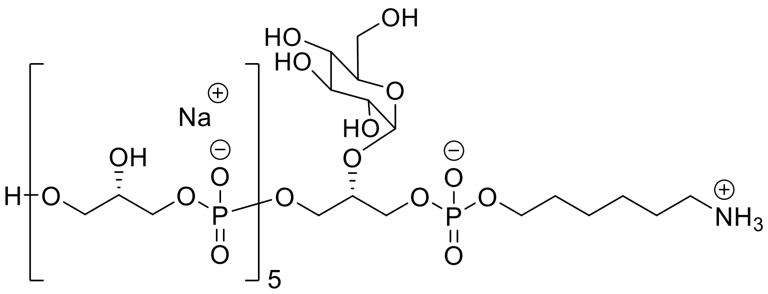

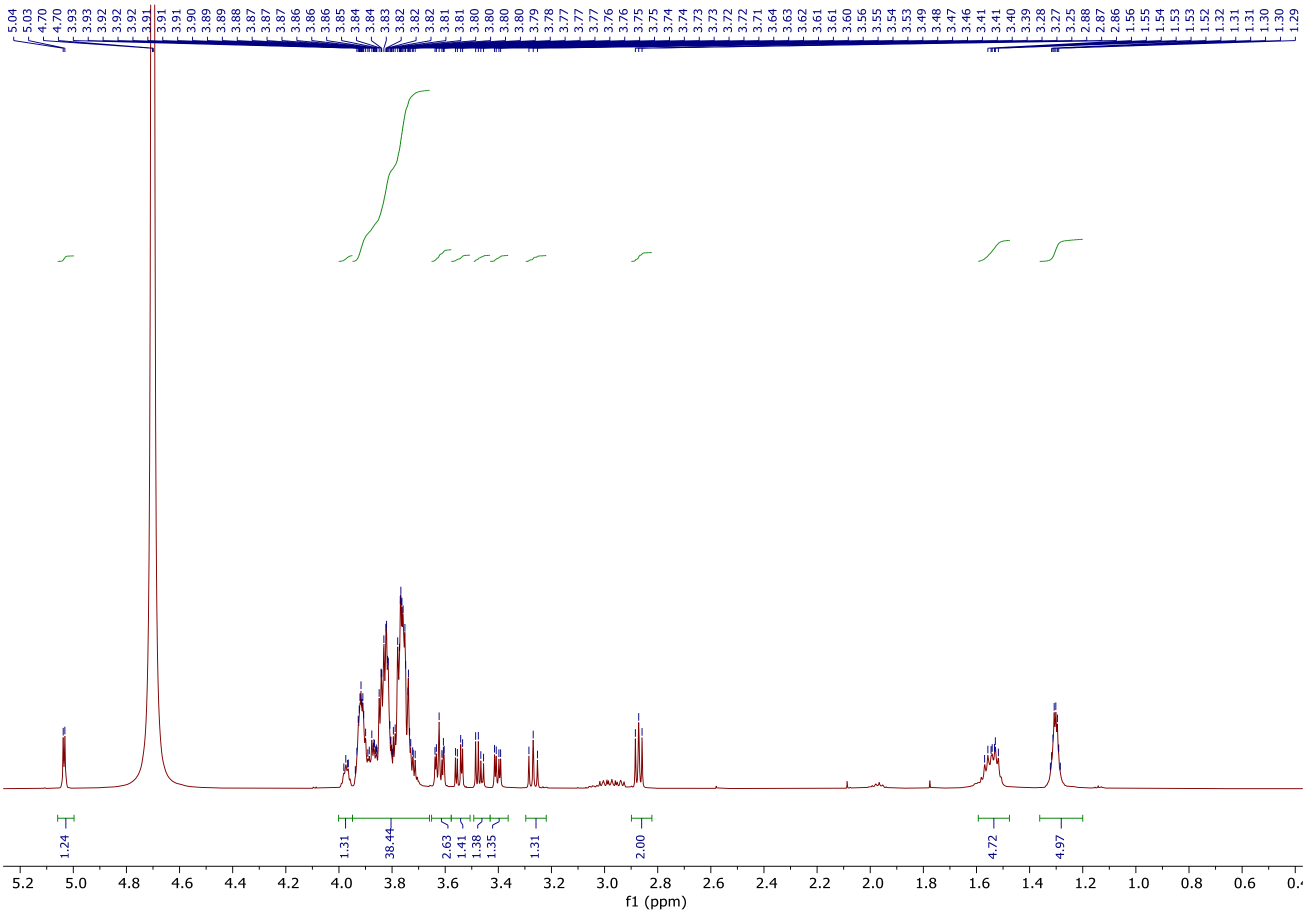

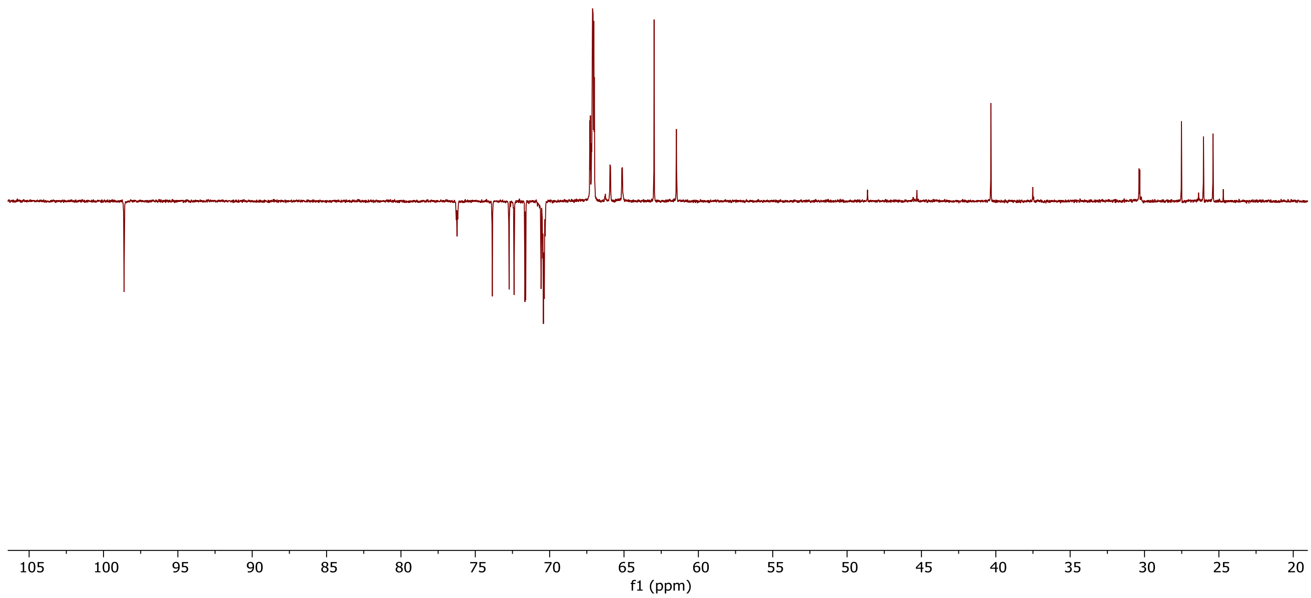
105 100 95 90 85 80 75 70 65 60 55 50 45 40 35 30 25 20
f1 (ppm)

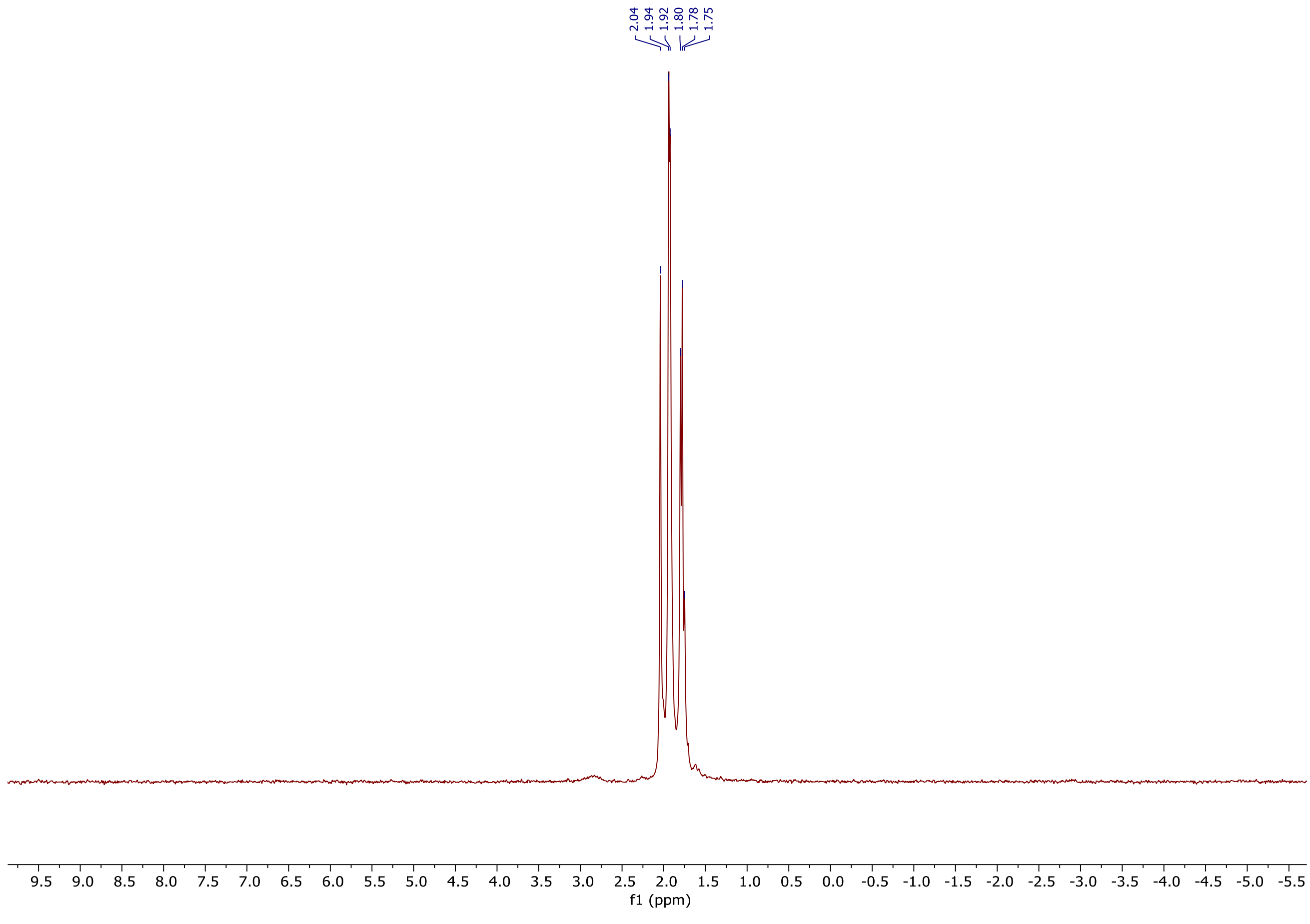
2.04
1.94
1.92
1.80
1.78
1.75
9.5 9.0 8.5 8.0 7.5 7.0 6.5 6.0 5.5 5.0 4.5 4.0 3.5 3.0 2.5 2.0 1.5 1.0 0.5 0.0 -0.5 -1.0 -1.5 -2.0 -2.5 -3.0 -3.5 -4.0 -4.5 -5.0 -5.5
f1 (ppm)
